# Evolutionary Analysis of the B56 Gene Family of PP2A Regulatory Subunits

**DOI:** 10.3390/ijms160510134

**Published:** 2015-05-05

**Authors:** Lauren M. Sommer, Hyuk Cho, Madhusudan Choudhary, Joni M. Seeling

**Affiliations:** 1 Department of Biological Sciences, Sam Houston State University, Huntsville, TX 77341, USA; E-Mails: lms047@shsu.edu (L.M.S.); mxc017@shsu.edu (M.C.); 2 Department of Computer Science, Sam Houston State University, Huntsville, TX 77341, USA; E-Mail: hxc005@shsu.edu

**Keywords:** protein phosphatase 2A, B56 regulatory subunit, molecular phylogeny

## Abstract

Protein phosphatase 2A (PP2A) is an abundant serine/threonine phosphatase that functions as a tumor suppressor in numerous cell-cell signaling pathways, including Wnt, myc, and ras. The B56 subunit of PP2A regulates its activity, and is encoded by five genes in humans. B56 proteins share a central core domain, but have divergent amino- and carboxy-termini, which are thought to provide isoform specificity. We performed phylogenetic analyses to better understand the evolution of the B56 gene family. We found that B56 was present as a single gene in eukaryotes prior to the divergence of animals, fungi, protists, and plants, and that B56 gene duplication prior to the divergence of protostomes and deuterostomes led to the origin of two B56 subfamilies, B56αβε and B56γδ. Further duplications led to three B56αβε genes and two B56γδ in vertebrates. Several nonvertebrate B56 gene names are based on distinct vertebrate isoform names, and would best be renamed. B56 subfamily genes lack significant divergence within primitive chordates, but each became distinct in complex vertebrates. Two vertebrate lineages have undergone B56 gene loss, *Xenopus* and *Aves*. In *Xenopus*, B56δ function may be compensated for by an alternatively spliced transcript, B56δ/γ, encoding a B56δ-like amino-terminal region and a B56γ core.

## 1. Introduction

Although signal transduction cascades are intensely studied, relatively little is known about the role that serine/threonine phosphatases play in them. While there are over 400 serine/threonine kinase genes in the human genome, there are only around 40 serine/threonine phosphatase catalytic subunits to counter them. This was initially interpreted to mean that phosphatases have broad, constitutive activities, however, it was later found that phosphatases are highly specific and that the majority of phosphatases achieve diversity by forming numerous distinct multimeric protein complexes. In the case of PP2A, there are at least three different B regulatory subunit gene families (B55/PR55/B, B56/PR56/B', and B72/PR72/B'') that bind to the structural A subunit and the catalytic C subunits, each of which is encoded by two genes in humans. Therefore, through the combinatorial effects of the association of multiple subunits, and with the inclusion of alternative splicing, PP2A could form as many as 200 different heterotrimers. As the B subunits are more diverse than the A and C subunits, they are the major contributors to substrate specificity and subcellular localization of the PP2A holoenzyme [1,2].

PP2A carries out essential cellular functions, and therefore its subunits are encoded by one of the most highly conserved sets of genes. The C subunit is the most conserved, with 75% identity between human and yeast proteins; human and yeast A subunit proteins share 44% identity. In humans, B56 isoforms are encoded by five widely expressed genes. B56 proteins are highly conserved between species, sharing approximately 60% identity between human and yeast. Even though individual B56 isoforms have distinct functions, the five human B56 proteins share 66% to 81% identity. B56 genes encode proteins with a highly conserved core of about 400 amino acids and variable amino- and carboxy-termini ranging from approximately ten to one hundred amino acids in length in humans. The divergent amino- and carboxy-termini are thought to provide specificity to the different isoforms. Alternative splicing occurs at the B56γ locus to produce a transcript with either a B56γ amino-terminal extension (B56γ/γ) or a mixed-isoform transcript containing a B56δ-like amino-terminal extension (B56δ/γ) [3]. As the B56 amino- and carboxy-termini are proposed to determine substrate specificity, these alternative splice products are likely to have distinct roles in the cell.

B56 isoforms have roles in numerous cell-cell signaling pathways. B56 isoforms modulate canonical Wnt signaling; most B56 isoforms are inhibitory to Wnt signaling, however, B56ε is required for Wnt signaling [4,5,6]. B56 isoforms also have a role in ras signaling, as transgenic mice with an A subunit mutation unable to bind B56 and an activating ras mutation have a reduced lifespan when compared to those solely possessing activated ras [7]. B56α inhibits Myc signaling by promoting Myc’s proteasomal-mediated degradation [8]. B56γ inhibits cell spreading and metastasis by dephosphorylating paxillin [9]. B56ε also has a role in hedgehog signaling [10].

Here we explored the evolution of the B56 gene family of PP2A regulatory subunits to provide us with a deeper understanding of B56 and how its evolution has resulted in five vertebrate genes that differentially regulate cell-cell signaling pathways. This characterization is especially important, as it will aid the integration of B56 studies in diverse organisms, especially when comparing functional analyses between species containing different complements of B56 genes. In addition, B56 isoforms can have antagonistic effects on signaling pathways, resulting in either growth inhibition or growth promotion. Understanding the origin of the antagonistic isoforms may be useful in understanding their disparate roles in signaling pathways. We performed a hierarchical clustering and a phylogenetic analysis to examine the highly conserved B56 isoforms. We traced the expansion of the B56 gene family from simple to complex organisms, and also found interesting patterns of gene duplication and deletion throughout the evolution of the B56 gene family.

## 2. Results and Discussion

### 2.1. Identification of B56 Gene Family Homologs

We analyzed B56 sequences from thirty-three species, each possessing between one and nine B56 genes, for a total analysis of 105 B56 sequences (Table 1). The best match of each vertebrate B56 protein sequence to the corresponding human B56 ortholog is shown in Table 2. We examined B56 genes from sixteen diverse species of mammals, birds, reptiles, amphibians, and fish. Each of the vertebrate B56 isoforms matched the corresponding human ortholog, as can be seen from their low expected values, and their high maximum scores, percent query coverages, percent identities, and percent similarities. Their amino acid identities ranged from 75% to 100%, while their similarities ranged from 80% to 100%. The B56ε gene is highly conserved, as the B56ε protein from *Homo sapiens* is identical to that in six species*: Macaca mulatta*, *Bos taurus*, *Ovis aries*, *Canis lupus familiaris*, *Gallus gallus*, and *Falco peregrinus*; and 97.2%–99.8% identical to that in seven species: *Mus musculus*, *Rattus norvegicus*, *Felis catus*, *Ambystoma mexicanum, Chrysemys picta bellii*, *Xenopus laevis*, and *Xenopus tropicalis.* This suggests that B56ε orthologs experienced a strong selective pressure to maintain their function.

**Table 1:** Summary of B56 sequences analyzed in comprehensive phylogenetic tree.

Group	Species	Isoforms/Species	Total Sequences
Plants	3	1–9	17
Protists	4	1	4
Fungi	5	1–2	6
Diploblasts	2	2	4
Protostomes	2	2	4
Echinoderm	1	2	2
Gnathostomes	16	4–5	68
Total	33	1–9	105

**Table 2:** Blast summary of vertebrate B56 sequence alignment. *Homo sapiens* B56α, B56β, B56γ, B56δ, and B56ε were used as queries in Blastp searches against the NCBI database. The highest-ranking chordate B56 isoform hits from fifteen species are provided along with their protein accession number (Accession #), E-value (E), maximum score (M), percent query coverage (Q), percent identity (% I), and percent similarity (% S). The superscript ^ denotes sequences that were not used in the phylogenetic analysis due to the short length of the sequence. The superscript ° denotes a sequence retrieved from *Xenopus* Database 3.2 (XB3.2) [11]. The superscript * denotes a sequence that came from Sal-Site [12], whereas the superscript ^2^ denotes a sequence that came from Uniprot [13]. The high level of conservation of the vertebrate B56 isoforms can be seen through the low E-values, high maximum scores, and high query coverages.

Query	Species	Class	Isoform	Accession #	E	M	Q	% I	% S
α	*Mus musculus*	*Mammalia*	α	NP_659129.2	0	976	100	97.7	98.8
β			β	NP_937811.1	0	1006	100	98.8	99.2
γ			γ	XP_006515983.1	0	1014	100	92.2	93.1
δ			δ	BAB62015.1	0	1178	100	96.0	97.3
γ			δ/γ	XP_006515987.1	0	978	96	91.9	93.1
ε			ε	NP_036154.1	0	955	100	99.8	100.0
α	*Felis catus*	*Mammalia*	α	XP_003999369.2 ^	0	820	100	99.5	99.5
β			β	XP_003993624.1	0	1012	100	99.2	99.6
γ			γ	XP_006933193.1 ^	0	905	90	88.2	89.6
δ			δ	XP_003986198.1	0	1217	88	98.7	99.0
ε			ε	XP_003987771.1	0	954	100	99.8	99.8
α	*Macaca mulatta*	*Mammalia*	α	NP_001244568.1	0	998	100	99.8	100.0
β			β	XP_001118226.1	0	692	88	83.6	86.1
γ			γ	XP_002805259.1	0	1052	100	99.2	99.4
δ			δ	XP_001087636.2	0	1177	100	95.3	95.8
γ			δ/γ	XP_001112240.1 ^	0	1020	96	98.6	99.2
ε			ε	NP_001253672.1	0	956	100	100.0	100.0
α	*Ovis aries*	*Mammalia*	α	XP_004013951.1	0	978	99	98.4	98.8
β			β	XP_004019707.1	0	953	100	94.6	95.6
γ			γ	XP_004018041.1 ^	0	888	96	99.5	99.5
δ			δ	XP_004019269.1	0	1164	98	96.5	96.8
ε			ε	XP_004010759.1	0	956	100	100.0	100.0
α	*Bos taurus*	*Mammalia*	α	NP_001075197.1	0	978	99	98.4	98.8
β			β	NP_001068700.1	0	1006	100	99.0	99.4
γ			γ	NP_001076845.1	0	1024	98	96.9	97.9
δ			δ	NP_001193287.1	0	1214	100	98.8	99.2
γ			δ/γ	XP_005222201.1	0	962	99	94.7	96.5
ε			ε	NP_001076937.1	0	956	100	100.0	100.0
α	*Canis lupus*	*Mammalia*	α	XP_005622406.1 ^	0	820	100	99.5	99.5
β	*familiaris*		β	XP_540876.1	0	1013	100	99.2	99.6
γ			γ	XP_854560.1	0	1031	100	97.5	97.7
δ			δ	XP_005627428.1	0	1195	100	97.5	98.0
γ			δ/γ	XP_005623875.1	0	996	96	97.2	97.8
ε			ε	XP_537472.2	0	956	100	100.0	100.0
α	*Rattus*	*Mammalia*	α	NP_001101361.1	0	980	100	97.9	98.8
β	*norvegicus*		β	NP_852044.1	0	1008	100	99.0	99.4
γ			γ	NP_001178041.1	0	1028	100	96.9	97.5
δ			N/A	AAH99800.1	0	1195	95	97.5	98.2
γ			δ/γ	XP_001077680.1	0	994	96	96.4	97.2
ε			ε	XP_006240284.1	0	955	100	99.8	100.0
α	*Falco*	*Aves*	α	XP_005238767.1	0	874	97	95.4	97.9
γ	*peregrinus*		γ	XP_005243013.1	0	980	100	94.7	96.7
δ			δ	XP_005242599.1	0	984	85	94.8	97.8
γ			δ/γ	XP_005243015.1 ^	0	848	87	94.2	96.4
ε			ε	XP_005241867 ^	0	956	100	100.0	100.0
α	*Gallus gallus*	*Aves*	α	XP_419432.2	0	872	97	95.0	97.7
γ			γ	NP_001072950.1	0	977	99	94.9	96.9
δ			δ	XP_419321.3	0	1086	98	90.9	93.9
ε			ε	XP_421412.2	0	956	100	100.0	100.0
α	*Alligator*	*Reptilia*	α	XP_006268191.1	0	858	96	94.9	97.7
β	*mississippiensis*		β	XP_006265023.1	0	806	92	90.5	94.8
γ			γ	XP_006270473.1	0	978	99	95.1	97.1
δ			δ	XP_006268832.1	0	1082	91	94.7	98.2
γ			δ/γ	XP_006270474.1 ^	0	847	87	94.6	96.9
ε			ε	XP_006264307.1 ^	0	739	100	78.3	80.2
α	*Chrysemys picta*	*Reptilia*	α	XP_005306377.1	0	874	97	95.2	97.7
β	*bellii*		β	XP_005307965.1	0	876	100	88.0	94.4
γ			γ	XP_005285849.1	0	977	99	94.3	96.7
δ			δ	XP_005293566.1	0	1088	100	91.1	94.4
γ			δ/γ	XP_005285851.1 ^	0	845	87	94.0	96.4
ε			ε	XP_008166448.1	0	937	100	98.7	98.7
α	*Xenopus laevis*	*Amphibia*	α	NP_001108316.1	0	838	88	92.3	97.2
β			β	rXL259o17ex °^	3 × 10^−20^	70.5	9	88.9	100.0
γ			γ	[3] ^	0	875	89	97.7	99.3
γ			δ/γ	NP_001087638.1 ^	0	840	85	97.6	99.5
ε			ε	NP_001088245.1	0	937	100	97.2	98.9
α	*Xenopus*	*Amphibia*	α	NP_001072157.1	0	844	97	90.3	95.6
β	*tropicalis*		β	NP_001093749.1	0	830	96	85.8	93.5
γ			δ/γ	Q6P3P7 ^2^	0	942	96	93.3	97.0
ε			ε	NP_989253.1	0	929	100	97.4	98.5
α	*Ambystoma*	*Amphibia*	α	contig314980 *^	0	743	82	92.6	96.8
β	*mexicanum*		β	contig328022 *^	0	655	72	87.2	94.4
γ			γ	contig314598 *^	0	950	99	91.6	94.9
δ			δ	contig133764 *^	0	739	64	95.9	99.2
ε			ε	contig190869 *^	0	865	92	97.7	99.3
α	*Danio rerio*	*Actino-pterygii*	α	XP_690932.3	0	791	97	82.1	91.4
β			β	XP_690770.2	0	787	96	81.2	89.7
γ			γ	XP_005160957.1	0	950	99	89.3	94.4
δ			δ	NP_998483.1	0	1043	97	84.9	91.0
δ			δ/γ	A4QP33 ^2^	0	914	98	75.3	84.4
ε			ε	NP_919396.1	0	871	100	90.2	95.3

B56 is also well conserved in simple chordates, nonchordate animals, fungi, protists, and plants. The amino acid identities between both simple chordate and nonchordate animals *versus* human B56 proteins were 59% to 84%, while their similarities were 77% to 94% (Table 3). The identities and similarities between fungi and protists *versus* human B56 proteins ranged from 51% to 62% and 69% to 80%, respectively (Table 3). The identities and similarities between plant and human B56 proteins were slightly less than those observed with fungi and protists, and ranged from 47% to 57% and 61% to 77%, respectively (Table 4). The high conservation of B56 proteins between animals, fungi, protists, and plants suggest that B56 plays a key role in basic cellular functions. The details of the protein similarities of vertebrates; simple animals, fungi, and protists; and plants, including data from all B56 pair-wise comparisons with human B56 isoforms, are listed in supplementary Tables S1–S3, respectively. An alignment of all analyzed B56 sequences is shown in supplementary Figure S1.

**Table 3:** Blast summary of simple chordate/nonchordate animal/fungi/protist B56 sequence alignment. *Homo sapiens* B56 isoforms were used as queries in Blastp searches against the NCBI database. Each of the five *H. sapiens* B56 isoforms was similar in its identity and similarity to each of the hits, and therefore no specific B56 isoform orthologs could be identified. However, the NCBI hits are listed with the *H. sapiens* query with which they had the lowest E-value. The NCBI hits are provided along with their protein accession number (Accession #), E-value (E), maximum score (M), percent query coverage (Q), percent identity (% I), and percent similarity (% S). The superscript ^1^ denotes sequences retrieved from JGI [14], while the superscript ^2^ denotes sequences from Uniprot [13]. The high level of conservation of the B56 isoforms in distant species can be seen through the low E-values, high maximum scores, and high query coverages. Identities range from 51% to 84% and similarities range from 69% to 94%.

Query	Species	Class	Isoform	Accession #	E	M	Q	% I	% S
α	*Petromyzon*	*Cephalaspidomorphi*	N/A	S4RHV1 ^2^	0	706	93	74.6	89.0
γ	*marinus*		N/A	S4RN43 ^2^	0	830	95	81.3	88.6
ε			N/A	S4RGA7 ^2^	9 × 10^−138^	388	56	81.9	94.4
γ	*Branchiostoma*	*Leptocardii*	N/A	BF237525 ^1^	0	775	94	78.6	87.4
γ	*floridae*		N/A	BF252487 ^1^	0	691	95	70.1	81.1
ε			N/A	BF112597 ^1^	1 × 10^−55^	171	36	59.1	77.3
ε			N/A	BF284583 ^1^	0	645	88	77.3	89.0
ε			N/A	BF237518 ^1^	0	685	81	84.4	93.9
δ	*Strongylocentrotus*	*Echinoidea*	δ	XP_003730246.1	0	797	90	72.2	81.9
ε	*purpuratus*		α	XP_780697.2	0	713	97	75.3	87.7
α	*Hydra vulgaris*	*Hydrozoa*	α	XP_002154794.2	0	725	94	74.0	88.1
γ			δ	XP_004208357.1	0	687	78	83.0	92.7
γ	*Amphimedon*	*Demospongiae*	β	XP_003386538.1	0	558	87	65.5	79.0
γ	*queenslandica*		δ	XP_003384582.1	0	678	93	67.0	79.0
δ	*Caenorhabditis*	*Chromadorea*	PPTR-2	NP_505808.1	0	741	88	65.6	79.4
ε	*elegans*		PPTR-1	NP_507133.4	0	641	95	69.0	82.7
α	*Drosophila*	*Insecta*	wdb	NP_733219.1	0	689	86	78.1	87.7
γ	*melanogaster*		B56-1	CAB86364.1	0	701	79	79.5	92.2
γ	*Aspergillus niger*	*Eurotiomycetes*	N/A	EHA23297.1	0	599	90	61.4	77.8
γ	*Aspergillus nidulans*	*Eurotiomycetes*	parA	XP_868849.1	0	608	94	62.1	79.5
γ	*Ashbya gossypii*	*Saccharomycetes*	RTS1	NP_984527	0	521	83	56.5	72.2
γ	*Saccharomyces cerevisiae*	*Saccharomycetes*	RTS1	AAB38372.1	3 × 10^−176^	508	80	56.0	72.4
γ	*Schizosaccharomyces*	*Schizosaccharomycetes*	par1	NP_588206.1	1 × 10^−178^	506	79	60.6	78.2
γ	*pombe*	*mycetes*	par2	NP_593298.1	1 × 10^−167^	481	79	54.8	76.3
δ	*Dictyostelium discoideum*	*Dictyostelia*	psrA	XP_641193.1	9 × 10^−147^	431	75	51.3	68.9
γ	*Dictyostelium purpureum*	*Dictyostelia*	N/A	XP_003290675.1	8 × 10^−149^	430	75	53.1	72.9
γ	*Dictyostelium fasciculatum*	*Dictyostelia*	N/A	XP_004360558.1	2 × 10^−149^	433	77	53.1	72.1
γ	*Polysphondylium pallidum*	*Dictyostelia*	N/A	EFA76858.1	1 × 10^−149^	431	76	53.9	74.0

**Table 4:** Blast summary of plant B56 sequence alignment. *Homo sapiens* B56 isoforms were used as queries in Blastp searches against the NCBI database. Each *H. sapiens* B56 isoform was similar in its identity and similarity to each of the hits, and therefore no specific B56 isoform orthologs could be identified. However, the NCBI hits are listed with the *H. sapiens* query with which they had the lowest E-value. The NCBI hits are provided along with their protein accession number (Accession #), E-value (E), maximum score (M), percent query coverage (Q), percent identity (% I), and percent similarity (% S). The relatively high level of conservation of the B56 isoforms in plant species can be seen through the low E-values, high maximum scores, and high query coverage.

Query	Species	Class	Isoform	Accession #	E	M	Q	% I	% S
γ	*Chlamydomonas reinhardtii*	*Chlorophyceae*	wdb	XP_001693445.1	0	775	94	57.1	76.5
γ	*Arabidopsis thaliana*	*Dicot*	θ	NP_973816.1	1 × 10^−170^	484	82	54.8	72.2
γ			ζ	NP_188802.1	2 × 10^−165^	473	83	53.2	70.3
γ			N/A	NP_197933.1	7 × 10^−154^	441	80	50.2	70.7
δ			α	NP_195967.1	2 × 10^−161^	464	71	54.5	74.9
δ			β	NP_187599.1	5 × 10^−166^	475	68	55.4	75.3
δ			δ	AAD02810.1	2 × 10^−165^	473	72	51.6	70.2
δ			γ	NP_849390.1	2 × 10^−168^	483	70	54.0	72.4
ε			ε	NP_191053.1	1 × 10^−155^	444	96	50.7	67.0
ε			η	NP_001154648.1	1 × 10^−140^	407	88	47.0	60.6
α	*Oryza sativa*	*Monocot*	N/A	NP_001059361.1	9 × 10^−161^	459	81	52.0	71.6
γ			N/A	AAP68376.1	3 × 10^−160^	459	79	52.1	71.7
γ			κ	CAC85920.1	5 × 10^−149^	429	80	49.8	68.8
δ			ζ	CAC85921.1	1 × 10^−171^	491	68	56.4	73.8
δ			θ	CAC85922.1	1 × 10^−166^	478	68	53.8	73.8
δ			N/A	NP_001054799.1	8 × 10^−136^	398	67	49.3	69.9
ε			η	NP_001053130.1	5 × 10^−171^	484	88	55.3	72.8

### 2.2. Hierarchical Clustering

**Figure 1:**
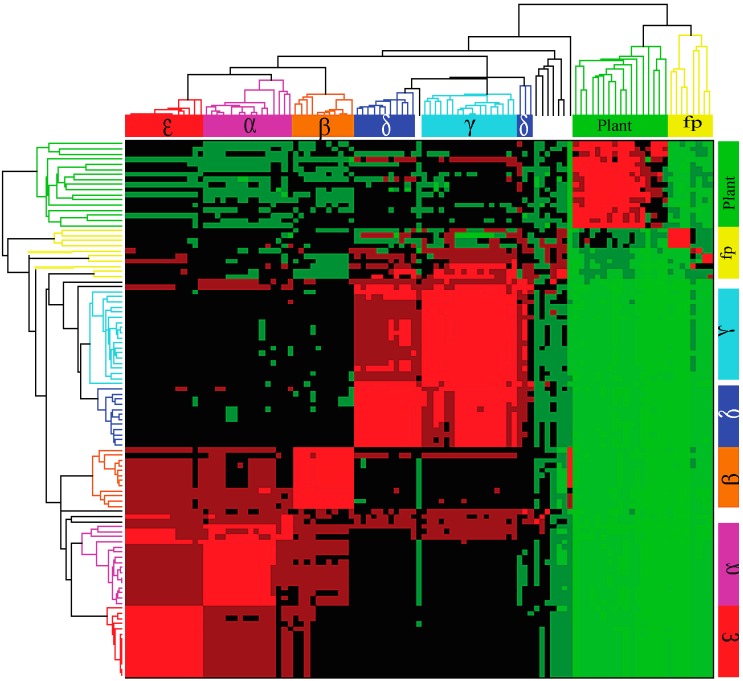
B56 hierarchical cluster based on percent identity. Each B56 protein sequence was chosen in turn as the query sequence in Blastp search. The resultant pair-wise percent identities were plotted. The identity is indicated by color, ranging from the highest to lowest identity, progressively colored light red, red, maroon, black, dark green, medium green, and light green. The B56 isoform designation refers to the vertebrate isoforms; fp refers to fungal and protist B56 genes; plant refers to plant B56 genes.

A hierarchical clustering was undertaken to gain insight into the relationship among the 105 B56 genes from animal, fungal, protist, and plant species. This analysis is based on sequence identity obtained through BLAST hits. The identity matrix was populated with the percent identity values, where rows correspond to the queries of the 105 genes, and columns correspond to the target database of the 105 genes. The identity matrix was then visualized using hierarchical clustering (Figure 1). The dendrograms and heat maps clearly delineate separate gene clusters for animal and plant B56 genes, with the animal cluster further subdivided into two clusters, B56αβε and B56γδ. Within the animal B56 genes, the B56αβε cluster has clearly grouped into its three isoforms and the B56γδ cluster has segregated into its two isoforms. The increased heterogeneity in the B56αβε cluster may suggest that the duplicate copies were retained because they acquired novel functions. The plant B56 genes do not segregate into distinct families, suggesting that plant B56 family genes underwent duplication later than in animal lineages. However, we only examined three plant species, and a broader analysis may reveal additional information. Species possessing a single B56 gene of each B56 subfamily (*Amphimedon queenslandica*, *Hydra vulgaris*, *Drosophila melanogaster*, *Caenorhabditis elegans*, and *Strongylocentrotus purpuratus*) generally fall in line with either the B56αβε or B56γδ subfamilies. Although this data visualization clearly delineates B56 subfamilies and suggests relationships between the B56 genes, it provides only an overview of B56 gene family divergence and evolution. A phylogenetic analysis was performed to trace the diversification of the B56 family.

### 2.3. B56 Gene Family Phylogeny

**Figure 2:**
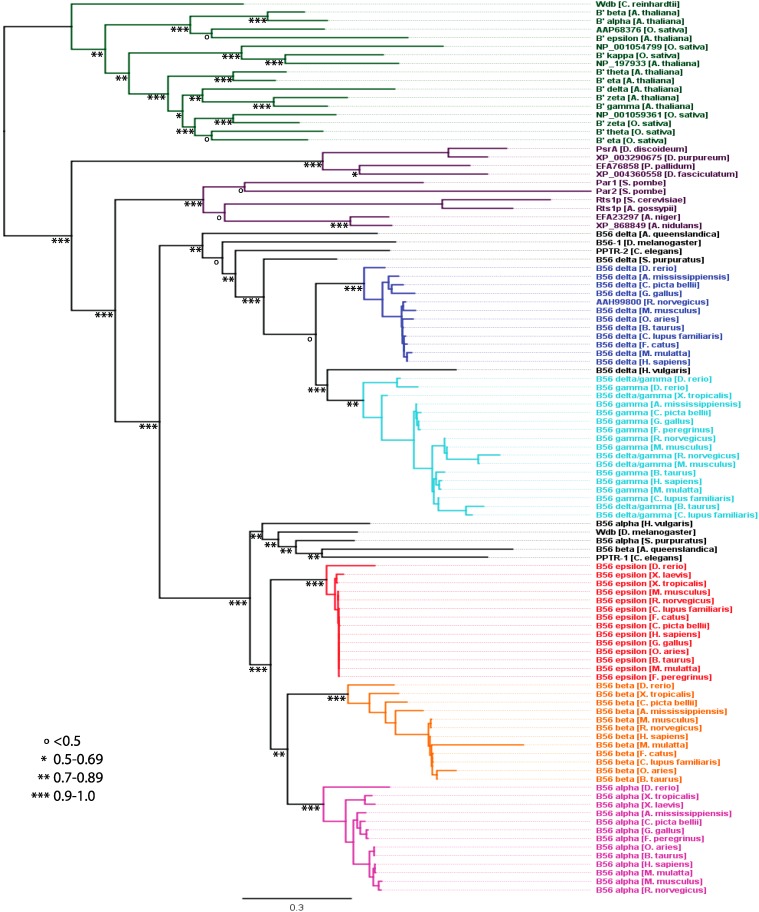
B56 phylogenetic tree. A B56 phylogenetic tree was built using FastTree 2. A B56 sequence was used in this analysis only if it contained 90% or more of the conserved core domain. Horizontal lines are proportional to the substitution rate. The bar represents 0.3 changes per amino acid. Local support values are marked with ***, **, *, and ^o^ for 0.9–1.0, 0.7–0.89, 0.5–0.69, and <0.5, respectively.

B56 was present as a single gene in eukaryotes prior to the divergence of animals, fungi, protists, and plants (Figure 2). Subsequently, four separate B56 clades evolved, mirroring species divergence. The plant B56 clade displayed the deepest division, followed by the protist B56 clade, with a local support value of 1.0, and then the fungal and animal B56 clades, with a local support value of 0.91. The B56αβε and B56γδ clades separated with a local support value of 0.93. Because of the structure of the B56 gene products, which are comprised of an approximately 400 amino acid conserved core domain and variable amino- and carboxy-termini, this global analysis included the core domain but the termini were excluded. This was a consequence of the lack of significant identity between the amino- and carboxy-termini of distant B56 isoforms, as the algorithm used for these analyses eliminates any region in the alignment displaying a gap in any sequence in the phylogenetic tree construction. To study the evolution of B56 genes in more detail, we examined individual B56 gene clades separately from the remaining B56 gene family, thereby reducing the exclusion of the less-conserved termini.

#### 2.3.1. Plants

**Figure 3:**
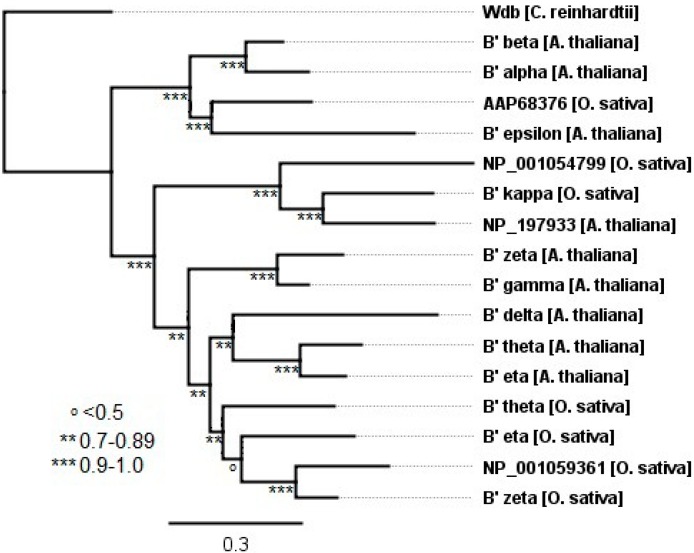
The evolution of B56 genes in plants. A plant B' phylogenetic tree was built using FastTree 2. The tree was rooted with *C*. *reinhardtii* wdb. Horizontal lines are proportional to the substitution rate. The bar represents 0.3 changes per amino acid. Local support values are marked with ***, **, and ^o^ for 0.9–1.0, 0.7–0.89, and <0.5, respectively.

Two different sets of nomenclature were initially used to describe the B56 genes, B56 and B', as several laboratories concurrently isolated the genes; the B' designations have been retained to describe the plant B56 genes [15]. The separate analysis of B56 plant genes yielded a phylogenetic tree with more sequence coverage than the global B56 analysis, as fewer sequence gaps reduced the extent of the sequences excluded in the FastTree phylogenetic tree construction. As *Chlamydomonas reinhardtii*, a unicellular green algae, is believed to be a representative of a terrestrial plant progenitor, the single B56 gene present in *C. reinhardtii* likely represents the B56 progenitor of multicellular plants [16]. The *C. reinhardtii* B56 gene is named wdb, which is a misnomer. It is not more highly related to its namesake, which was initially identified in *D. melanogaster*, than to the other B56 isoforms, and would more appropriately be renamed B56, without an isoform designation [17]. The B56 gene was duplicated numerous times within multicellular plant species, as *Arabidopsis thaliana* has nine B56 genes while *Oryza sativa* (Japanese rice) has seven (Figure 3). A previous report proposed a B56 family tree composed of eight *A. thaliana* and five *O. sativa* genes based on a neighbor-joining algorithm UPGMA (Unweighted Pair Group Method with Arithmetic Mean). The tree consisted of three B56 subfamilies named B'α, B'η, and B'κ, with two *A. thaliana* genes, B'γ and B'δ, placed outside of the defined subfamilies [18]. Our analysis employed several multiple sequence alignment algorithms and maximum likelihood methods for phylogenetic tree construction, and differs from that previously proposed (Figure 3 and data not shown). Three distinct clades were resolved. Each of these clades was present in both *A. thaliana* and *O. sativa*, and therefore likely present prior to the divergence of monocots (*O. sativa*) and dicots (*A. thaliana*). One clade consists of B'β, B'α, and B'ε from *A. thaliana* and an unannotated gene from *O. sativa*, AAP68376, and was supported with a local support value of 1.0. The other two clades diverged with a local support value of 0.96. One of these clades consists of *O. sativa* (B'κ and NP_001054799) and *A. thaliana* (NP_197933) genes. The other clade consists of three subgroups: *A. thaliana* B'ζ and B'γ; *A. thaliana* B'δ, B'θ, and B'η; and *O. sativa* B'θ, B'η, B'ζ, and NP_001059361. As each of these subfamilies was either *A. thaliana* or *O. sativa* specific, they likely resulted from duplications occurring within each species. In summary, plants express a unique set of B56 gene orthologs and paralogs that have undergone both pre-speciation and post-speciation duplications.

#### 2.3.2. Protists and Fungi

**Figure 4:**
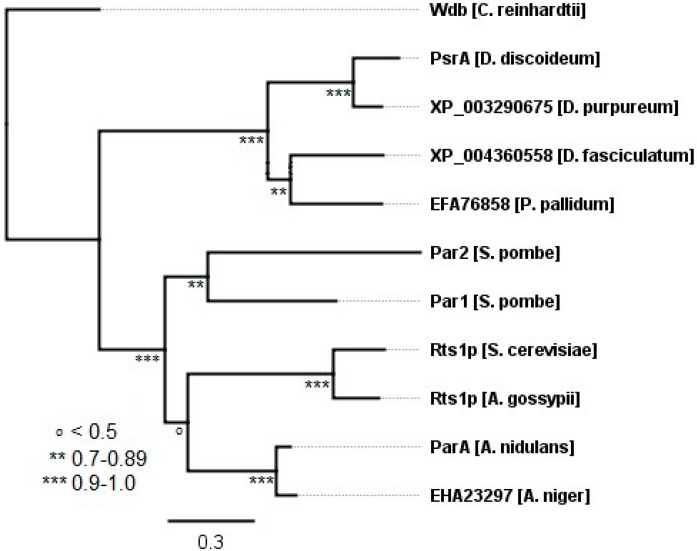
The evolution of B56 genes in fungi and protists. A B56 protist and fungal phylogenetic tree was built using FastTree 2. Each tree was rooted with *C. reinhardtii* wdb. Horizontal lines are proportional to the substitution rate. The bar represents 0.3 changes per amino acid. Local support values are marked with ***, **, and ^o^ for 0.9–1.0, 0.7–0.89, and <0.5, respectively.

The protists, *Dictyostelium discoideum*, *Dictyostelium purpureum*, *Dictyostelium fasciculatum*, and *Polyspondylium pallidum*, each contain a single B56 gene, as do the fungi *Saccharomyces cerevisiae*, *Ashbya gossypii*, *Aspergillus nidulans*, and *Aspergillus niger* (Figure 4). In contrast, the fungus *Schizosaccharomyces pombe* possesses two B56 genes, likely resulting from a gene duplication occurring after the divergence of *Aspergillus* and *S. pombe*. The lineage of the B56 gene does not precisely follow that of the fungal species. With regard to species divergence, *S. cerevisiae* and *A. gossypii* form a clade separate from *S. pombe* and *Aspergillus* species, whereas with the B56 gene, *S. cerevisiae*, *A. gossypii*, and *Aspergillus* form a clade separate from *S. pombe*. This is not uncommon, as many fungal species have acquired genes by horizontal gene transfer from not only distantly related fungal species, but also from bacteria and plants [19,20].

#### 2.3.3. Animals

A duplication of the B56 gene prior to the divergence of diploblastic and triploblastic species, animals with two or three germ layers, respectively, led to the formation of two animal B56 clades, B56αβε (B56-1) and B56γδ (B56-2), with a local support value of 0.93 (Figure 2). The diploblasts *Amphimedon queenslandica* (sponge) and *Hydra vulgaris* (fresh water polyp) maintained one representative from each B56 subfamily (*A. queenslandica:* B56β and B56δ; *H. vulgaris*: B56α and B56δ). Within the triploblasts, protostomes *D. melanogaster* and *Caenorhabditis* *elegans* retained a single B56 gene from each subfamily: wdb and PPTR-1 from B56αβε, and B56-1 and PPTR-2 from B56γδ, respectively. In deuterostomes, *Strongylocentrotus purpuratus* (sea urchin) possesses a B56 gene from each subfamily, named B56α and B56δ. Although current nomenclature suggests that these genes may be more closely related to an individual isoform within the subfamilies, *A. queenslandica*, *H. vulgaris*, *D. melanogaster*, *C. elegans*, and *S. purpuratus*, B56αβε and B56γδ subfamily genes are derived from branches that diverged prior to divergence within the B56αβε and B56γδ subfamily clades. Consequently, *A. queenslandica* B56β, *H. vulgaris* B56α, *D. melanogaster* wdb, *C. elegans* PPTR-1, and *S. purpuratus* B56α should be more appropriately named; we suggest B56-1. In addition, *A. queenslandica* B56δ, *H. vulgaris* B56δ, *D. melanogaster* B56-1, *C. elegans* PPTR-2, and *S. purpuratus* B56δ should be more appropriately named to signify that they diverged prior to divergence of the B56γδ subfamily clade, perhaps with the name B56-2. In congruence with this nomenclature, the B56αβε subfamily would become the B56-1 subfamily and the B56γδ subfamily would become the B56-2 subfamily.

**Figure 5:**
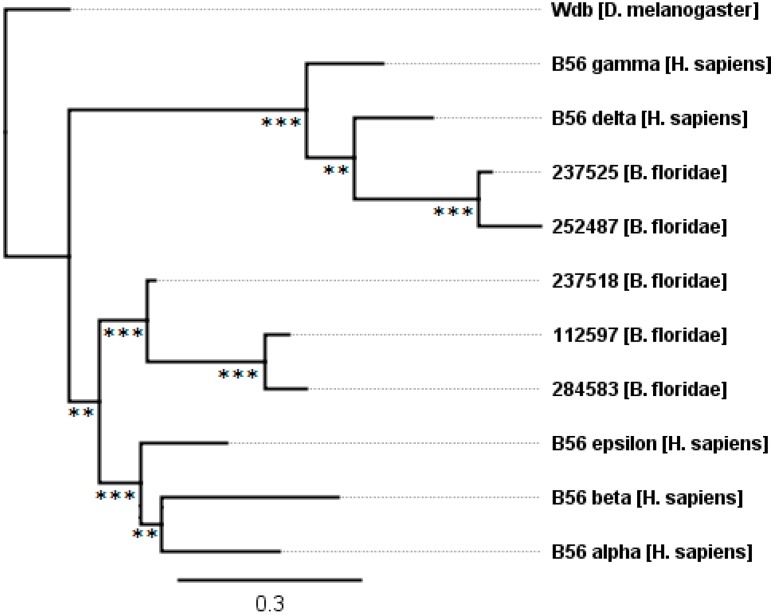
The evolution of B56 genes in the simple chordate *B. floridae*. *B. floridae* B56 isoforms have diverged into B56αβε and B56γδ subfamilies. The tree was rooted with *D. melanogaster* wdb. Horizontal lines are proportional to the substitution rate. The bar represents 0.3 changes per amino acid. Local support values are marked with *** and ** for 0.9–1.0 and 0.7–0.89.

Two rounds of whole-genome duplications occurred after the divergence of urochordates (e.g., sea squirt) and cephalochordates (e.g., lancelets) but prior to the divergence of cyclostomes (e.g., lamprey) and gnathostomes (jawed vertebrates) [21]. Many paralogous genes present on duplicated genomes were lost, but some remain. Not surprisingly then, chordates contain higher copy numbers of B56 genes than simpler organisms. *B. floridae* (lancelet, a chordate containing a neural cord and notochord but lacking vertebrae), whose genome sequence was first reported in 2008, has a full complement of five B56 genes [22]. Three of these genes share 70%–90% identity and 82%–96% similarity with one another and fall into the B56αβε subfamily, but have not separated into distinct B56α, B56β, and B56ε isoforms; the other two B56 genes share 88% identity and 90% similarity and are within the B56γδ subfamily (Figure 5). This suggests that *B. floridae* branched off from vertebrate progenitors after two rounds of whole genome duplication, but prior to the time at which the B56αβε or B56γδ subfamilies evolved into the five vertebrate isoforms. In addition, the presence of three B56αβε genes and two B56γδ genes suggests that one B56αβε gene and two B56γδ genes were lost after the whole-genome duplications (or one B56γδ gene was lost after the first genome-wide duplication).

**Figure 6:**
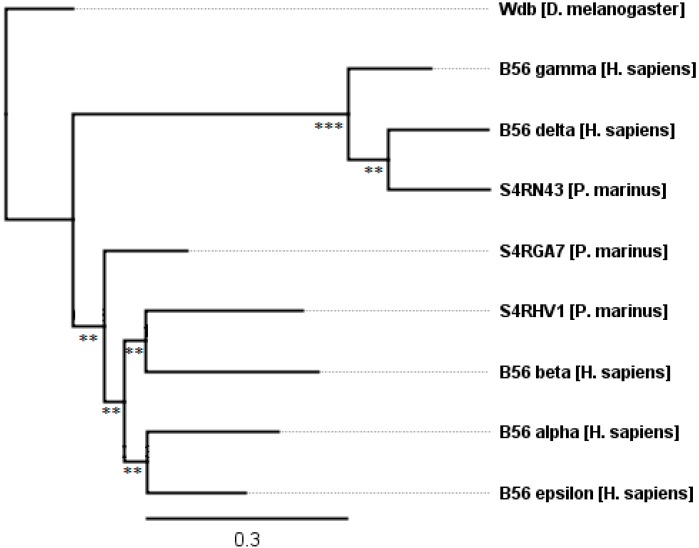
The evolution of B56 genes in the simple chordate *P. marinus*. One *P. marinus* B56 family member remains undifferentiated in the B56αβε subfamily while two correspond to the vertebrate isoforms B56α and B56δ. The tree was rooted with *D. melanogaster* wdb. Horizontal lines are proportional to the substitution rate. The bar represents 0.3 changes per amino acid. Local support values are marked with *** and ** for 0.9–1.0 and 0.7–0.89.

The genome sequence of *P. marinus* (sea lamprey, a primitive vertebrate) was first reported in 2013, and is available at 5.0X whole genome coverage [23,24]. We identified three *P. marinus* B56 genes: two B56αβε subfamily members and one B56γδ subfamily member (Table 2). Similar to *B. floridae*, one B56αβε subfamily member diverged from the B56αβε clade prior to isoform specialization (S4RGA7, Figure 6). However, S4RHV1 forms a clade with B56β, while S4RN43 forms a clade with B56δ. This suggests that *P. marinus* branched off from vertebrates after isoform specialization had started, but before it had been completed. *P. marinus*’ phylogenetic position suggests that it will possess a full complement of B56 genes; these genes will likely be revealed once a more complete coverage of the *P. marinus* genome is obtained. The five chordate B56 genes present in *B. floridae* are maintained in all chordates examined, with two exceptions, as described below.

The B56γδ clade has a two-fold lower substitution rate than the B56αβε clade before they first branch (leading to 12% and 25% divergence, respectively) (Figure 2). This finding correlates with the heat map (Figure 1), suggesting that the B56γδ clade is either newer than the B56αβε clade, or that it is under stronger selection to maintain its sequence. Our data suggest that the B56γδ clade has fewer substitutions because it resulted from the second genome-wide duplication, with the paralogs from the first genome-wide duplication being lost. However, our data does not rule out the possibility that the B56γδ clade may be more constrained. Future studies of synonymous/non-synonymous changes may determine the mechanism behind the conservation of the B56γδ clade, as well as the mechanism behind the limited B56 subfamily divergence in *B. floridae* and *P. marinus.*

### 2.4. The B56αβε Subfamily

**Figure 7:**
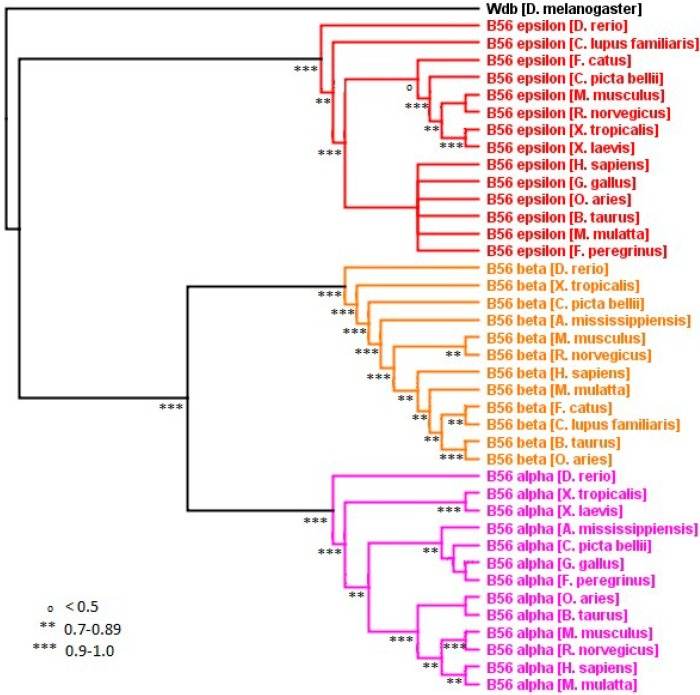
Vertebrate B56αβε phylogenetic tree. A B56αβε phylogenetic tree was built using FastTree 2. The tree was rooted with *D. melanogaster* wdb. Horizontal lines are not proportional to the substitution rate in order to display the clade topology. Local support values are marked with ***, **, and ^o^ for 0.9–1.0, 0.7–0.89, and <0.5, respectively.

Within the B56αβε subfamily, individual B56 isoforms exhibited distinct levels of evolutionary change. *D. rerio* B56 αβε genes were most divergent from the rest of the species examined (Figure 2 and Figure 7). This was not unexpected, as *D. rerio* (zebrafish) is the outlier of the vertebrate species examined. B56ε displayed the most stringent conservation, as it underwent 4% amino acid changes excluding *D. rerio*, and 13% amino acid changes including *D. rerio.* B56α displayed an intermediate level of conservation, as it underwent 8% amino acid changes excluding *D. rerio*, and 18% amino acid changes including *D. rerio.* B56β was the least conserved, as it underwent 23% amino acid changes excluding *D. rerio*, and 29% amino acid changes including *D. rerio* (each also excluding *M. mulatta* (rhesus macaque))*. M. mulatta’s* B56β gene displayed an exceptionally high amino acid substitution rate, 25% since its divergence from other mammals. This was due in large part to a 63 amino acid region in the amino half of its core that lacks significant conservation with other B56 sequences. In addition, unlike B56α and B56ε, reptilian and amphibian B56β displayed a relatively high amino acid substitution rate, 14% *versus* 8% and 4% in B56α and B56ε, respectively, again suggesting reduced constraint on B56β sequence in these species (Figure 2). In summary, B56ε was under the strongest selective pressure to maintain its sequence, whereas B56α was under moderate selective pressure. B56β’s selective pressure was similar to B56α in mammalian genes (excluding *M. mulatta*), but much looser in reptiles and amphibians. Alternatively, B56α and B56β may have been under positive selection.

The evolution of the B56αβε subfamily is of particular interest, as isoforms within this subfamily have antagonistic effects on the canonical Wnt signaling pathway [4,6]. B56ε is required for canonical Wnt signaling, whereas B56α inhibits Wnt signaling. There is also evidence suggesting that B56β has an inhibitory role [4]. An earlier report used UPGMA to suggest that B56α and B56ε are more highly related to one another than to B56β [1]. We carried out several analyses to sort out the relationships within the B56αβε subfamily, using FastTree 2, Bayesian, and neighbor joining programs (Figure 7 and data not shown). The majority of our analyses showed that B56ε diverged prior to B56α and B56β. However, there were also instances where B56α and B56ε appeared more closely related. Therefore, our data is suggestive of B56ε being more distantly related to B56α and B56β, correlating with the functional data, but this conclusion is not robust. This ambiguity was likely due to the fact that there were few informational differences within the B56αβε clade.

### 2.5. The B56γδ Subfamily

**Figure 8:**
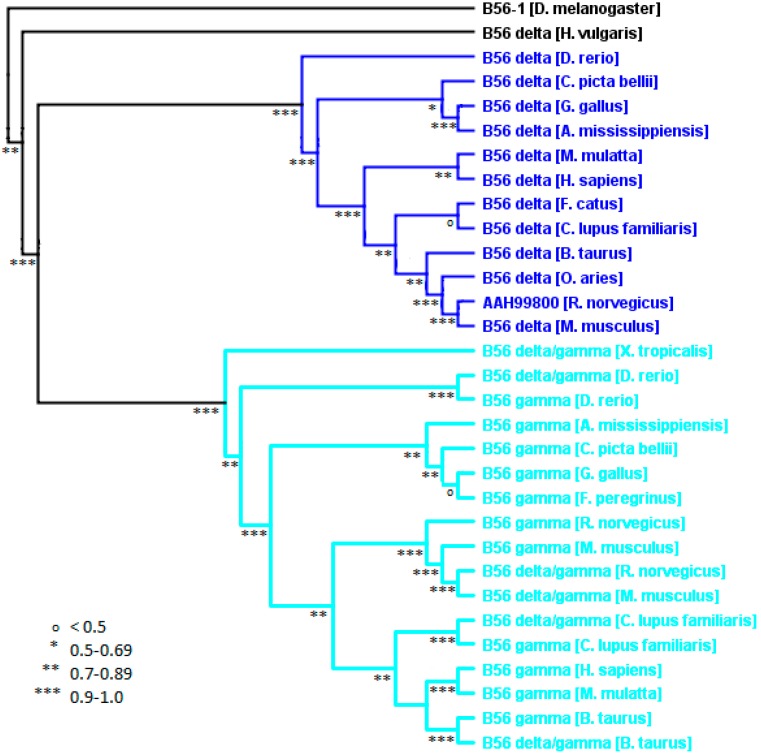
Vertebrate B56γδ phylogenetic tree. A B56γδ phylogenetic tree was built using FastTree 2. The tree was rooted with *D. melanogaster* B56-1. Horizontal lines are not proportional to the substitution rate in order to display the clade topology. Local support values are marked with ***, **, * and ^o^ for 0.9–1.0, 0.7–0.89, 0.5–0.69, and <0.5, respectively.

A distinct analysis of the B56γδ subfamily was carried out to construct a B56γδ phylogenetic tree based on sequences specific for the B56γδ subfamily to gain insight that was not obtained from the global B56 analysis, which was based on the core domain. Both B56γ and B56δ vertebrate isoforms differed by approximately 12% when the B56δ/γ splice variants were not included in the analysis (Figure 2 and Figure 8, and data not shown). With the inclusion of B56δ/γ, B56γ differed by 29%. This is due to the fact that B56δ/γ has an 82 amino acid amino-terminal region that is not related to the 19 amino acid amino-terminal region of B56γ/γ.

*H. vulgaris* contains one B56 gene from each subfamily. The B56γδ family member of *H. vulgaris* segregated within the B56δ clade in the larger phylogenetic analysis of B56 (Figure 2). However, all other B56 proteins that were examined from nonchordate animal species did not segregate into distinct isoforms within the B56 subfamily clades. We therefore included the *H. vulgaris* B56γδ protein in our analysis of the vertebrate B56γδ subfamily to more accurately place *H. vulgaris* B56γδ within the B56 tree. This B56γδ-specific analysis placed *H. vulgaris* B56γδ within the B56γδ subfamily but outside of the B56γ and B56δ isoform clades. Therefore, the *H. vulgaris* B56γδ protein now falls in line with other diploblasts (*A. queenslandica*), protostomes (*D. melanogaster* and *C. elegans*), and primitive deuterostomes (*S. purpuratus*) in which the B56 genes have not evolved into distinct isoforms.

### 2.6. The Loss of Vertebrate B56 Genes

**Figure 9:**
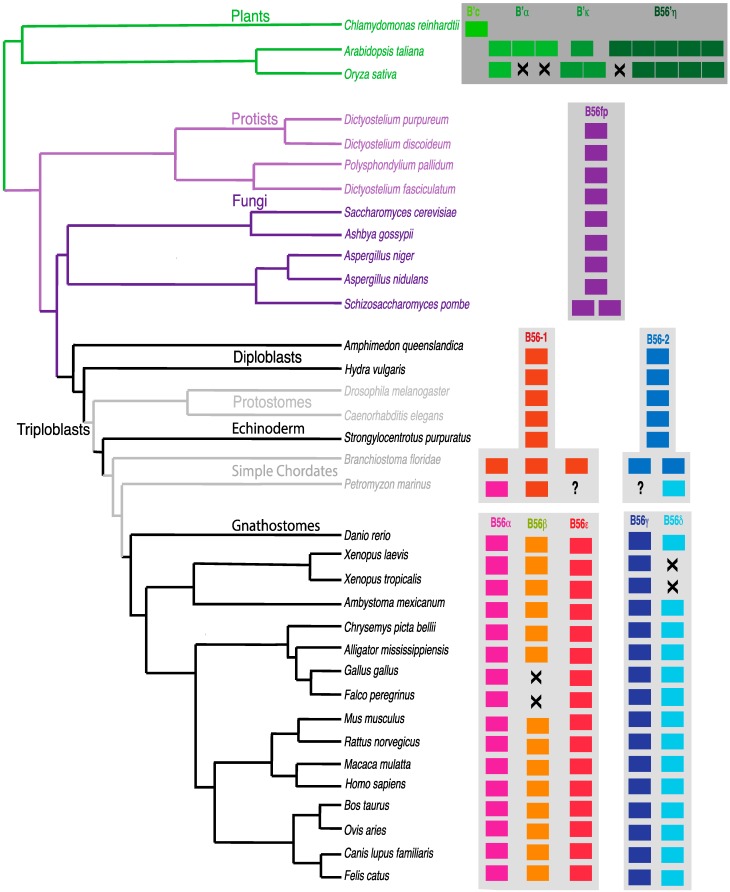
Distribution of B56 genes in plants, protists, fungi, and animals. A species tree was constructed based on the Tree of Life [25]. B56 genes are represented by rectangles; the absence of a B56 gene is signified with an X; uncertainty in the presence of a B56 isoform is signified by a question mark.

B56δ was not found in *X. laevis* or *X. tropicalis* but was present in *A. mexicanum*, a closely related amphibian (Figure 9). As *X. tropicalis’s* genome has been completely sequenced, this strongly suggests that the B56δ gene was lost in these two *Xenopus* species. Within *archosaurs*, B56β was not found in *G. gallus* and *F. peregrinus* but was present in *Alligator mississippiensis*. As *G. gallus*’, *F. peregrinus*’, and *A. mississippiensis’s* genomes have all been completely sequenced, this strongly suggests that the B56β gene was lost in the *Aves* lineage. These two separate B56 gene losses suggest that B56 isoforms may share some overlapping functions. Since the amino- and carboxy-terminal variable domains of the protein are likely to be key in carrying out isoform-specific functions, similarities in these regions may be important in understanding the potential for functional overlap between B56 isosforms. Overlapping functions would be more likely to occur within a B56 subfamily. For example, the function of B56δ in *Xenopus* is more likely to have been maintained by B56γ rather than by a B56 αβε family member, whereas the function of B56β in *Aves* would more likely be carried by B56α or B56ε. Indeed, the amino-terminal variable regions of human B56α, B56β, and B56ε are approximately 50% identical and 60% similar, while their carboxy-termini lack significant similarity. Therefore, the similarity of the amino-terminal domains in the B56αβε subfamily may provide sufficient functional overlap to allow the loss of one family member. The amino-terminal variable region of human B56γ and B56δ lack significant similarity, but their carboxy-termini possess approximately 50% identity and 56% similarity, therefore their carboxy-termini, but not their amino-termini, may provide some overlapping functions. Alternatively, we previously identified an evolutionarily conserved alternative splice form of B56γ that contains a B56δ-like amino-terminal variable region [3]. This B56δ/γ isoform may be sufficient to carry out B56δ-specific functions in *Xenopus*. Indeed, as B56δ/γ and B56γ share their B56γ core and carboxy-termini, they are somewhat intermingled on the phylogenetic tree, with B56δ/γ and B56γ from the same species, such as *B. taurus*, *C. lupus familiaris*, and *D. rerio*, often segregating together (Figure 8).

## 3. Experimental Section

### 3.1. Identification of B56 Gene Homologs

All members of the B56 gene family (B56α, B56β, B56γ, B56δ, and B56ε) were identified from diverse species of animals, fungi, protists, and plants from the NCBI. Amino acid sequences of *Homo sapiens* B56 isoform proteins were used as queries to identify the corresponding target homologs of different species using Blastp [26]. A symmetrical similarity search scheme was employed to perform all pair-wise comparisons to confirm the homologs, and their accession numbers were then retrieved. The following stringency criteria were used for the identification of the best matches: percent query coverage ≥ 50, maximum score ≥ 100, percent identity ≥ 40, and E-value ≤ 10^−3^.

### 3.2. Hierarchical Clustering

In this analysis, each of the B56 protein sequences was chosen in turn as the query sequence in a Blastp search. We collected the pair-wise amino acid identity values for all possible pairs of total 105 members of the B56 protein family, and used the resulting protein percent identity matrix for data visualization. We used agglomerative hierarchical clustering to visualize similarities within and between B56 isoforms. Hierarchical clustering constructs a hierarchical structure of input data and it has become a standard visualization method since its seminal application to microarray data [27,28]. Particularly, agglomerative clustering method creates a hierarchical structure through a bottom-up approach, in which a pair of closest clusters is merged at each step. Agglomerative clustering takes an input of pair-wise similarities (or distances) among data items, from which cluster similarities (or distances) are inferred for grouping data items. We utilized the *clustergram* function in the Bioinformatics Toolbox of a commercial software package MATLAB 7.11 (R2010b) (MathWorks, Natick, MA, USA) and generated the heat map with dendrograms as shown in Figure 1. Each row of the identity matrix was transformed so that its mean is 0 and the standard deviation is 1 for better visualization. Also, average linkage (*i.e.*, UPGMA) was used to compute Euclidean distance between a data point and a cluster.

### 3.3. Phylogenetic Analysis

Phylogenetic analysis was run on the Geneious version 7.1.5 platform [29]. B56 protein sequences from selected species were input into Geneious, and sequences were aligned using MUSCLE (MUltiple Sequence Comparison by Log-Expectation) [30]. A phylogenetic tree was inferred for these aligned protein sequences with FastTree version 2.1.5 with default settings [31]. The resulting phylogeny was rooted by using the plant B56 genes as an out-group. FastTree 2 is an approximately maximum-likelihood phylogenetic method which efficiently uses alignment with a large number of genes or protein sequences [31]. It is openly available software and it produces phylogenetic trees in a short amount of time that are as accurate as trees constructed by other maximum-likelihood methods such as PhyML 3.0 or RAxML 7.0. FastTree2 uses the CAT (category) approximation [32] to account for variation in rates across sites and also implements the Shimodaira-Hasegawa (SH) test [33] to estimate the reliability of each split in the phylogeny, which is the same as PhyML3’s SH-like local support values [34]. A species phylogenetic tree was constructed based on the Tree of Life [25].

## 4. Conclusions

The B56 gene family is highly conserved. B56 was present as a single gene in simple eukaryotes, but was duplicated prior to the divergence of protostomes and deuterostomes. Further duplications occurred in chordates, resulting in three B56αβε and two B56γδ genes. These genes remained similar to one another in simple chordates, but diverged into five distinct isoforms in vertebrates. B56ε was most highly conserved, followed by B56α, B56γ, and B56δ, which displayed an intermediate level of conservation; B56β was the least conserved. This divergence in vertebrates likely led to the ability of B56 family members to regulate numerous signal transduction pathways.

The deletion of B56δ in *Xenopus* species and B56β in *Aves* suggests that some B56 isoforms may have overlapping functions. However, in the case of B56δ, there exists an evolutionarily conserved mixed-isoform alternative splice form that contains a B56δ-like amino-terminal variable domain upstream of the B56γ core region [3]. This strengthens the argument that the variable regions largely determine isoform specificity, as the presence of a B56δ amino-terminal variable domain appears to compensate for loss of the B56δ.

## Supplementary Materials

Supplementary materials can be found at http://www.mdpi.com/1422-0067/16/05/10134/s1.
