# Supplementary material for: Evolutionary Analysis of the B56 Gene Family of PP2A Regulatory Subunits
Source: Int J Mol Sci. 2015 May 5;16(5):10134–57. doi: 10.3390/ijms160510134 (PMC4463637; doi:10.3390/ijms160510134)
Supplement: Supplementary file 1 [file ijms-16-10134-s001.zip › ijms-80778-Supplementary Information/ijms-80778-Supplementary Figure.pdf]

Supplementary Information

```
CLUSTAL 2.1 multiple sequence alignment


gi|Aspergillus_niger_N/A           --------------------------------------------------
gi|Aspergillus_nidulans_parA       --------------------------------------------------
gi|Mus_musculus_alpha              --------------------------------------------------
gi|Rattus_norvegicus_alpha         --------------------------------------------------
gi|Macaca_mulatta_alpha            --------------------------------------------------
gi|Homo_sapiens_alpha              --------------------------------------------------
gi|Ovis_aries_alpha                --------------------------------------------------
gi|Bos_taurus_alpha                --------------------------------------------------
gi|Felis_catus_alpha               --------------------------------------------------
gi|Canis_lupus_familiaris_alph     --------------------------------------------------
gi|Falco_peregrinus_alpha          --------------------------------------------------
gi|Gallus_gallus_alpha             --------------------------------------------------
gi|Alligator_mississippiensis_     --------------------------------------------------
gi|Chrysemys_picta_bellii_alph     --------------------------------------------------
gi|Xenopus_laevis_alpha            --------------------------------------------------
gi|Xenopus_tropicalis_alpha        --------------------------------------------------
gi|Ambystoma_mexicanum_alpha       --------------------------------------------------
gi|Danio_rerio_alpha               --------------------------------------------------
gi|Petromyzon_marinus_S4RHV1       --------------------------------------------------
gi|Mus_musculus_epsilon            --------------------------------------------------
gi|Rattus_norvegicus_epsilon       --------------------------------------------------
gi|Macaca_mulatta_epsilon          --------------------------------------------------
gi|Homo_sapiens_epsilon            --------------------------------------------------
gi|Ovis_arie_epsilon               --------------------------------------------------
gi|Bos_taurus_epsilon              --------------------------------------------------
gi|Canis_lupus_familiaris_epsi     --------------------------------------------------
gi|Falco_peregrinus_epsilon        --------------------------------------------------
gi|Gallus_gallus_epsilon           --------------------------------------------------
gi|Felis_catus_epsilon             --------------------------------------------------
gi|Chrysemys_picta_bellii_epsi     --------------------------------------------------
gi|Xenopus_laevis_epsilon          --------------------------------------------------
gi|Xenopus_tropicalis_epsilon      --------------------------------------------------
gi|Alligator_mississippiensis_     --------------------------------------------------
gi|Ambystoma_mexicanum_epsilon     --------------------------------------------------
gi|Danio_rerio_epsilon             --------------------------------------------------
gi|Mus_musculus_beta               --------------------------------------------------
gi|Rattus_norvegicus_beta          --------------------------------------------------
gi|Felis_catus_beta                --------------------------------------------------
gi|Canis_lupus_familiaris_beta     --------------------------------------------------
gi|Homo_sapiens_beta               --------------------------------------------------
gi|Bos_taurus_beta                 --------------------------------------------------
gi|Ovis_aries_beta                 --------------------------------------------------
gi|Macaca_mulatta_beta             --------------------------------------------------
gi|Xenopus_laevis_beta             --------------------------------------------------
gi|Alligator_mississippiensis_     --------------------------------------------------
gi|Chrysemys_picta_bellii_beta     --------------------------------------------------
gi|Xenopus_tropicalis_beta         --------------------------------------------------
gi|Danio_rerio_beta                --------------------------------------------------
gi|Ambystoma_mexicanum_beta        --------------------------------------------------
gi|Petromyzon_marinus_S4RGA7       --------------------------------------------------
gi|Strongylocentrotus_purpurat     --------------------------------------------------
gi|Drosophila_melanogaster_wdb     --------------------------------------------------
jgi|Branchiostoma_floridae_237     --------------------------------------------------
gi|Hydra_vulgaris_alpha            --------------------------------------------------
jgi|Branchiostoma_floridae_112     --------------------------------------------------
jgi|Branchiostoma_floridae_284     --------------------------------------------------
gi|Caenorhabditis_elegans_PPTR     --------------------------------------------------
```

Figure S1. *Cont.*

```
gi|Amphimedon_queenslandica_be      --------------------------------------------------
gi|Chrysemys_picta_bellii_gamm      --------------------------------------------------
gi|Chrysemys_picta_bellii_delt      --------------------------------------------------
gi|Falco_peregrinus_gamma           --------------------------------------------------
gi|Falco_peregrinus_delta/gamm      --------------------------------------------------
gi|Gallus_gallus_gamma              --------------------------------------------------
gi|Alligator_mississippiensis_      --------------------------------------------------
gi|Alligator_mississippiensis_      --------------------------------------------------
gi|Ambystoma_mexicanum_gamma        --------------------------------------------------
gi|Mus_musculus_gamma               --------------------------------------------------
gi|Rattus_norvegicus_gamma          --------------------------------------------------
gi|Ovis_aries_gamma                 --------------------------------------------------
gi|Bos_taurus_gamma                 --------------------------------------------------
gi|Canis_lupus_familiaris_gamm      --------------------------------------------------
gi|Macaca_mulatta_gamma             --------------------------------------------------
gi|Homo_sapiens_gamma               --------------------------------------------------
gi|Felis_catus_gamma                --------------------------------------------------
gi|Canis_lupus_familiaris_delt      --------------------------------------------------
gi|Macaca_mulatta_delta/gamma       MAQGAATGRGIPLQATVAEAGPVDFPDTSQNSVSPEGRGGKGQAVHAERQ 50
gi|Bos_taurus_delta/gamma           --------------------------------------------------
gi|Mus_musculus_delta/gamma         --------------------------------------------------
gi|Rattus_norvegicus_delta/gam      --------------------------------------------------
gi|Danio_rerio_gamma                --------------------------------------------------
gi|Danio_rerio_delta/gamma          --------------------------------------------------
gi|Xenopus_laevis_delta/gamma       --------------------------------------------------
gi|Xenopus_laevis_gamma             --------------------------------------------------
gi|Xenopus_tropicalis_delta/ga      --------------------------------------------------
gi|Mus_musculus_delta               --------------------------------------------------
gi|Rattus_norvegicus_delta          --------------------------------------------------
gi|Felis_catus_delta                --------------------------------------------------
gi|Bos_taurus_delta                 --------------------------------------------------
gi|Canis_lupus_familiaris_delt      --------------------------------------------------
gi|Macaca_mulatta_delta             --------------------------------------------------
gi|Homo_sapiens_delta               --------------------------------------------------
gi|Ovis_aries_delta                 --------------------------------------------------
gi|Falco_peregrinus_delta           --------------------------------------------------
gi|Gallus_gallus_delta              --------------------------------------------------
gi|Alligator_mississippiensis_      --------------------------------------------------
gi|Chrysemys_picta_bellii_delt      --------------------------------------------------
gi|Danio_rerio_delta                --------------------------------------------------
gi|Ambystoma_mexicanum_delta        --------------------------------------------------
gi|Petromyzon_marinus_S4RN43        --------------------------------------------------
jgi|Branchiostoma_floridae_237      --------------------------------------------------
jgi|Branchiostoma_floridae_252      --------------------------------------------------
gi|Strongylocentrotus_purpurat      --------------------------------------------------
gi|Caenorhabditis_elegans_PPTR      --------------------------------------------------
gi|Hydra_vulgaris_delta             --------------------------------------------------
gi|Amphimedon_queenslandica_de      --------------------------------------------------
gi|Drosophila_melanogaster_B56      --------------------------------------------------
gi|Dictyostelium_discoideum_ps      --------------------------------------------------
gi|Dictyostelium_purpureum_N/A      --------------------------------------------------
gi|Dictyostelium_fasciculatum_      --------------------------------------------------
gi|Polysphondylium_pallidum_N/      --------------------------------------------------
gi|Arabidopsis_thaliana_theta       --------------------------------------------------
gi|Arabidopsis_thaliana_eta         --------------------------------------------------
gi|Arabidopsis_thaliana_zeta        --------------------------------------------------
gi|Arabidopsis_thaliana_gamma       --------------------------------------------------
gi|Oryza                            --------------------------------------------------
gi|Oryza_sativa_zeta                --------------------------------------------------
gi|Oryza_sativa_theta               --------------------------------------------------
gi|Oryza_sativa_eta                 --------------------------------------------------
```

Figure S1. *Cont.*

```
gi|Arabidopsis_thaliana_delta      --------------------------------------------------
gi|Arabidopsis_thaliana_N/A        --------------------------------------------------
gi|Oryza_sativa_kappa              --------------------------------------------------
gi|Oryza_sativa_N/A                --------------------------------------------------
gi|Arabidopsis_thaliana_alpha      --------------------------------------------------
gi|Arabidopsis_thaliana_beta       --------------------------------------------------
gi|Oryza_sativa_N/A_2              --------------------------------------------------
gi|Arabidopsis_thaliana_epsilo     --------------------------------------------------
gi|Chlamydomonas_reinhardtii_w     --------------------------------------------------
gi|Schizosaccharomyces_pombe_P     --------------------------------------------------
gi|Schizosaccharomyces_pombe_P     --------------------------------------------------
gi|Ashbya_gossypii_RTS1            --------------------------------------------------
gi|Saccharomyces_cerevisiae_RT     --------------------------------------------------

gi|Aspergillus_niger_N/A           --------------------------------------------------
gi|Aspergillus_nidulans_parA       --------------------------------------------------
gi|Mus_musculus_alpha              --------------------------------------------------
gi|Rattus_norvegicus_alpha         --------------------------------------------------
gi|Macaca_mulatta_alpha            --------------------------------------------------
gi|Homo_sapiens_alpha              --------------------------------------------------
gi|Ovis_aries_alpha                --------------------------------------------------
gi|Bos_taurus_alpha                --------------------------------------------------
gi|Felis_catus_alpha               --------------------------------------------------
gi|Canis_lupus_familiaris_alph     --------------------------------------------------
gi|Falco_peregrinus_alpha          --------------------------------------------------
gi|Gallus_gallus_alpha             --------------------------------------------------
gi|Alligator_mississippiensis_     --------------------------------------------------
gi|Chrysemys_picta_bellii_alph     --------------------------------------------------
gi|Xenopus_laevis_alpha            --------------------------------------------------
gi|Xenopus_tropicalis_alpha        --------------------------------------------------
gi|Ambystoma_mexicanum_alpha       --------------------------------------------------
gi|Danio_rerio_alpha               --------------------------------------------------
gi|Petromyzon_marinus_S4RHV1       --------------------------------------------------
gi|Mus_musculus_epsilon            --------------------------------------------------
gi|Rattus_norvegicus_epsilon       --------------------------------------------------
gi|Macaca_mulatta_epsilon          --------------------------------------------------
gi|Homo_sapiens_epsilon            --------------------------------------------------
gi|Ovis_arie_epsilon               --------------------------------------------------
gi|Bos_taurus_epsilon              --------------------------------------------------
gi|Canis_lupus_familiaris_epsi     --------------------------------------------------
gi|Falco_peregrinus_epsilon        --------------------------------------------------
gi|Gallus_gallus_epsilon           --------------------------------------------------
gi|Felis_catus_epsilon             --------------------------------------------------
gi|Chrysemys_picta_bellii_epsi     --------------------------------------------------
gi|Xenopus_laevis_epsilon          --------------------------------------------------
gi|Xenopus_tropicalis_epsilon      --------------------------------------------------
gi|Alligator_mississippiensis_     --------------------------------------------------
gi|Ambystoma_mexicanum_epsilon     --------------------------------------------------
gi|Danio_rerio_epsilon             --------------------------------------------------
gi|Mus_musculus_beta               --------------------------------------------------
gi|Rattus_norvegicus_beta          --------------------------------------------------
gi|Felis_catus_beta                --------------------------------------------------
gi|Canis_lupus_familiaris_beta     --------------------------------------------------
gi|Homo_sapiens_beta               --------------------------------------------------
gi|Bos_taurus_beta                 --------------------------------------------------
gi|Ovis_aries_beta                 --------------------------------------------------
gi|Macaca_mulatta_beta             --------------------------------------------------
gi|Xenopus_laevis_beta             --------------------------------------------------
gi|Alligator_mississippiensis_     --------------------------------------------------
gi|Chrysemys_picta_bellii_beta     --------------------------------------------------
```

Figure S1. *Cont.*

```
gi|Xenopus_tropicalis_beta          --------------------------------------------------
gi|Danio_rerio_beta                 --------------------------------------------------
gi|Ambystoma_mexicanum_beta         --------------------------------------------------
gi|Petromyzon_marinus_S4RGA7        --------------------------------------------------
gi|Strongylocentrotus_purpurat      --------------------------------------------------
gi|Drosophila_melanogaster_wdb      --------------------------------------------------
jgi|Branchiostoma_floridae_237      --------------------------------------------------
gi|Hydra_vulgaris_alpha             --------------------------------------------------
jgi|Branchiostoma_floridae_112      --------------------------------------------------
jgi|Branchiostoma_floridae_284      --------------------------------------------------
gi|Caenorhabditis_elegans_PPTR      --------------------------------------------------
gi|Amphimedon_queenslandica_be      --------------------------------------------------
gi|Chrysemys_picta_bellii_gamm      --------------------------------------------------
gi|Chrysemys_picta_bellii_delt      --------------------------------------------------
gi|Falco_peregrinus_gamma           --------------------------------------------------
gi|Falco_peregrinus_delta/gamm      --------------------------------------------------
gi|Gallus_gallus_gamma              --------------------------------------------------
gi|Alligator_mississippiensis_      --------------------------------------------------
gi|Alligator_mississippiensis_      --------------------------------------------------
gi|Ambystoma_mexicanum_gamma        --------------------------------------------------
gi|Mus_musculus_gamma               --------------------------------------------------
gi|Rattus_norvegicus_gamma          --------------------------------------------------
gi|Ovis_aries_gamma                 --------------------------------------------------
gi|Bos_taurus_gamma                 --------------------------------------------------
gi|Canis_lupus_familiaris_gamm      --------------------------------------------------
gi|Macaca_mulatta_gamma             --------------------------------------------------
gi|Homo_sapiens_gamma               --------------------------------------------------
gi|Felis_catus_gamma                --------------------------------------------------
gi|Canis_lupus_familiaris_delt      --------------------------------------------------
gi|Macaca_mulatta_delta/gamma       SLQVCQDGVVTWKRILFTGAGGCSLSKMQWRFSLSPHEGEAPMPLAREIR 100
gi|Bos_taurus_delta/gamma           --------------------------------------------------
gi|Mus_musculus_delta/gamma         --------------------------------------------------
gi|Rattus_norvegicus_delta/gam      --------------------------------------------------
gi|Danio_rerio_gamma                --------------------------------------------------
gi|Danio_rerio_delta/gamma          --------------------------------------------------
gi|Xenopus_laevis_delta/gamma       --------------------------------------------------
gi|Xenopus_laevis_gamma             --------------------------------------------------
gi|Xenopus_tropicalis_delta/ga      --------------------------------------------------
gi|Mus_musculus_delta               --------------------------------------------------
gi|Rattus_norvegicus_delta          --------------------------------------------------
gi|Felis_catus_delta                --------------------------------------------------
gi|Bos_taurus_delta                 --------------------------------------------------
gi|Canis_lupus_familiaris_delt      --------------------------------------------------
gi|Macaca_mulatta_delta             --------------------------------------------------
gi|Homo_sapiens_delta               --------------------------------------------------
gi|Ovis_aries_delta                 --------------------------------------------------
gi|Falco_peregrinus_delta           --------------------------------------------------
gi|Gallus_gallus_delta              --------------------------------------------------
gi|Alligator_mississippiensis_      --------------------------------------------------
gi|Chrysemys_picta_bellii_delt      --------------------------------------------------
gi|Danio_rerio_delta                --------------------------------------------------
gi|Ambystoma_mexicanum_delta        --------------------------------------------------
gi|Petromyzon_marinus_S4RN43        --------------------------------------------------
jgi|Branchiostoma_floridae_237      --------------------------------------------------
jgi|Branchiostoma_floridae_252      --------------------------------------------------
gi|Strongylocentrotus_purpurat      --------------------------------------------------
gi|Caenorhabditis_elegans_PPTR      --------------------------------------------------
gi|Hydra_vulgaris_delta             --------------------------------------------------
gi|Amphimedon_queenslandica_de      --------------------------------------------------
gi|Drosophila_melanogaster_B56      --------------------------------------------------
gi|Dictyostelium_discoideum_ps      --------------------------------------------------
```

Figure S1. *Cont.*

```
gi|Dictyostelium_purpureum_N/A      --------------------------------------------------
gi|Dictyostelium_fasciculatum_      --------------------------------------------------
gi|Polysphondylium_pallidum_N/      --------------------------------------------------
gi|Arabidopsis_thaliana_theta       --------------------------------------------------
gi|Arabidopsis_thaliana_eta         --------------------------------------------------
gi|Arabidopsis_thaliana_zeta        --------------------------------------------------
gi|Arabidopsis_thaliana_gamma       --------------------------------------------------
gi|Oryza                            --------------------------------------------------
gi|Oryza_sativa_zeta                --------------------------------------------------
gi|Oryza_sativa_theta               --------------------------------------------------
gi|Oryza_sativa_eta                 --------------------------------------------------
gi|Arabidopsis_thaliana_delta       --------------------------------------------------
gi|Arabidopsis_thaliana_N/A         --------------------------------------------------
gi|Oryza_sativa_kappa               --------------------------------------------------
gi|Oryza_sativa_N/A                 --------------------------------------------------
gi|Arabidopsis_thaliana_alpha       --------------------------------------------------
gi|Arabidopsis_thaliana_beta        --------------------------------------------------
gi|Oryza_sativa_N/A_2               --------------------------------------------------
gi|Arabidopsis_thaliana_epsilo      --------------------------------------------------
gi|Chlamydomonas_reinhardtii_w      --------------------------------------------------
gi|Schizosaccharomyces_pombe_P      --------------------------------------------------
gi|Schizosaccharomyces_pombe_P      --------------------------------------------------
gi|Ashbya_gossypii_RTS1             --------------------------------------------------
gi|Saccharomyces_cerevisiae_RT      --------------------------------------------------


gi|Aspergillus_niger_N/A            --------------------------------------------------
gi|Aspergillus_nidulans_parA        --------------------------------------------------
gi|Mus_musculus_alpha               --------------------------------------------------
gi|Rattus_norvegicus_alpha          --------------------------------------------------
gi|Macaca_mulatta_alpha             --------------------------------------------------
gi|Homo_sapiens_alpha               --------------------------------------------------
gi|Ovis_aries_alpha                 --------------------------------------------------
gi|Bos_taurus_alpha                 --------------------------------------------------
gi|Felis_catus_alpha                --------------------------------------------------
gi|Canis_lupus_familiaris_alph      --------------------------------------------------
gi|Falco_peregrinus_alpha           --------------------------------------------------
gi|Gallus_gallus_alpha              --------------------------------------------------
gi|Alligator_mississippiensis_      --------------------------------------------------
gi|Chrysemys_picta_bellii_alph      --------------------------------------------------
gi|Xenopus_laevis_alpha             --------------------------------------------------
gi|Xenopus_tropicalis_alpha         --------------------------------------------------
gi|Ambystoma_mexicanum_alpha        --------------------------------------------------
gi|Danio_rerio_alpha                --------------------------------------------------
gi|Petromyzon_marinus_S4RHV1        --------------------------------------------------
gi|Mus_musculus_epsilon             --------------------------------------------------
gi|Rattus_norvegicus_epsilon        --------------------------------------------------
gi|Macaca_mulatta_epsilon           --------------------------------------------------
gi|Homo_sapiens_epsilon             --------------------------------------------------
gi|Ovis_arie_epsilon                --------------------------------------------------
gi|Bos_taurus_epsilon               --------------------------------------------------
gi|Canis_lupus_familiaris_epsi      --------------------------------------------------
gi|Falco_peregrinus_epsilon         --------------------------------------------------
gi|Gallus_gallus_epsilon            --------------------------------------------------
gi|Felis_catus_epsilon              --------------------------------------------------
gi|Chrysemys_picta_bellii_epsi      --------------------------------------------------
gi|Xenopus_laevis_epsilon           --------------------------------------------------
gi|Xenopus_tropicalis_epsilon       --------------------------------------------------
gi|Alligator_mississippiensis_      --------------------------------------------------
gi|Ambystoma_mexicanum_epsilon      --------------------------------------------------
gi|Danio_rerio_epsilon              --------------------------------------------------
```

Figure S1. *Cont.*

```
gi|Mus_musculus_beta                 --------------------------------------------------
gi|Rattus_norvegicus_beta            --------------------------------------------------
gi|Felis_catus_beta                  --------------------------------------------------
gi|Canis_lupus_familiaris_beta       --------------------------------------------------
gi|Homo_sapiens_beta                 --------------------------------------------------
gi|Bos_taurus_beta                   --------------------------------------------------
gi|Ovis_aries_beta                   --------------------------------------------------
gi|Macaca_mulatta_beta               --------------------------------------------------
gi|Xenopus_laevis_beta               --------------------------------------------------
gi|Alligator_mississippiensis_       --------------------------------------------------
gi|Chrysemys_picta_bellii_beta       --------------------------------------------------
gi|Xenopus_tropicalis_beta           --------------------------------------------------
gi|Danio_rerio_beta                  --------------------------------------------------
gi|Ambystoma_mexicanum_beta          --------------------------------------------------
gi|Petromyzon_marinus_S4RGA7         --------------------------------------------------
gi|Strongylocentrotus_purpurat       --------------------------------------------------
gi|Drosophila_melanogaster_wdb       --------------------------------------------------
jgi|Branchiostoma_floridae_237       --------------------------------------------------
gi|Hydra_vulgaris_alpha              --------------------------------------------------
jgi|Branchiostoma_floridae_112       --------------------------------------------------
jgi|Branchiostoma_floridae_284       --------------------------------------------------
gi|Caenorhabditis_elegans_PPTR       --------------------------------------------------
gi|Amphimedon_queenslandica_be       --------------------------------------------------
gi|Chrysemys_picta_bellii_gamm       --------------------------------------------------
gi|Chrysemys_picta_bellii_delt       --------------------------------------------------
gi|Falco_peregrinus_gamma            --------------------------------------------------
gi|Falco_peregrinus_delta/gamm       --------------------------------------------------
gi|Gallus_gallus_gamma               --------------------------------------------------
gi|Alligator_mississippiensis_       --------------------------------------------------
gi|Alligator_mississippiensis_       --------------------------------------------------
gi|Ambystoma_mexicanum_gamma         --------------------------------------------------
gi|Mus_musculus_gamma                --------------------------------------------------
gi|Rattus_norvegicus_gamma           --------------------------------------------------
gi|Ovis_aries_gamma                  --------------------------------------------------
gi|Bos_taurus_gamma                  --------------------------------------------------
gi|Canis_lupus_familiaris_gamm       --------------------------------------------------
gi|Macaca_mulatta_gamma              --------------------------------------------------
gi|Homo_sapiens_gamma                --------------------------------------------------
gi|Felis_catus_gamma                 --------------------------------------------------
gi|Canis_lupus_familiaris_delt       --------------------------------------------------
gi|Macaca_mulatta_delta/gamma        TLLESYYLMHFRNPGRLSLASPIVLGTRWTAEVLGHQVFEAWPRWAGPRP 150
gi|Bos_taurus_delta/gamma            --------------------------------------------------
gi|Mus_musculus_delta/gamma          --------------------------------------------------
gi|Rattus_norvegicus_delta/gam       --------------------------------------------------
gi|Danio_rerio_gamma                 --------------------------------------------------
gi|Danio_rerio_delta/gamma           --------------------------------------------------
gi|Xenopus_laevis_delta/gamma        --------------------------------------------------
gi|Xenopus_laevis_gamma              --------------------------------------------------
gi|Xenopus_tropicalis_delta/ga       --------------------------------------------------
gi|Mus_musculus_delta                --------------------------------------------------
gi|Rattus_norvegicus_delta           --------------------------------------------------
gi|Felis_catus_delta                 --------------------------------------------------
gi|Bos_taurus_delta                  --------------------------------------------------
gi|Canis_lupus_familiaris_delt       --------------------------------------------------
gi|Macaca_mulatta_delta              --------------------------------------------------
gi|Homo_sapiens_delta                --------------------------------------------------
gi|Ovis_aries_delta                  --------------------------------------------------
gi|Falco_peregrinus_delta            --------------------------------------------------
gi|Gallus_gallus_delta               --------------------------------------------------
gi|Alligator_mississippiensis_       --------------------------------------------------
gi|Chrysemys_picta_bellii_delt       --------------------------------------------------
```

Figure S1. *Cont.*

```
gi|Danio_rerio_delta              --------------------------------------------------
gi|Ambystoma_mexicanum_delta      --------------------------------------------------
gi|Petromyzon_marinus_S4RN43      --------------------------------------------------
jgi|Branchiostoma_floridae_237    --------------------------------------------------
jgi|Branchiostoma_floridae_252    --------------------------------------------------
gi|Strongylocentrotus_purpurat    --------------------------------------------------
gi|Caenorhabditis_elegans_PPTR    --------------------------------------------------
gi|Hydra_vulgaris_delta           --------------------------------------------------
gi|Amphimedon_queenslandica_de    --------------------------------------------------
gi|Drosophila_melanogaster_B56    --------------------------------------------------
gi|Dictyostelium_discoideum_ps    --------------------------------------------------
gi|Dictyostelium_purpureum_N/A    --------------------------------------------------
gi|Dictyostelium_fasciculatum_    --------------------------------------------------
gi|Polysphondylium_pallidum_N/    --------------------------------------------------
gi|Arabidopsis_thaliana_theta     --------------------------------------------------
gi|Arabidopsis_thaliana_eta       --------------------------------------------------
gi|Arabidopsis_thaliana_zeta      --------------------------------------------------
gi|Arabidopsis_thaliana_gamma     --------------------------------------------------
gi|Oryza                          --------------------------------------------------
gi|Oryza_sativa_zeta              --------------------------------------------------
gi|Oryza_sativa_theta             --------------------------------------------------
gi|Oryza_sativa_eta               --------------------------------------------------
gi|Arabidopsis_thaliana_delta     --------------------------------------------------
gi|Arabidopsis_thaliana_N/A       --------------------------------------------------
gi|Oryza_sativa_kappa             --------------------------------------------------
gi|Oryza_sativa_N/A               --------------------------------------------------
gi|Arabidopsis_thaliana_alpha     --------------------------------------------------
gi|Arabidopsis_thaliana_beta      --------------------------------------------------
gi|Oryza_sativa_N/A_2             --------------------------------------------------
gi|Arabidopsis_thaliana_epsilo    --------------------------------------------------
gi|Chlamydomonas_reinhardtii_w    --------------------------------------------------
gi|Schizosaccharomyces_pombe_P    --------------------------------------------------
gi|Schizosaccharomyces_pombe_P    --------------------------------------------------
gi|Ashbya_gossypii_RTS1           ---------------------MMRGFKQKLIKKTTGSSSSTQ-KKKDKE- 27
gi|Saccharomyces_cerevisiae_RT    ---------------------MMRGFKQRLIKKTTGSSSSSSSKKKDKEK 29

gi|Aspergillus_niger_N/A          ------------------------------MKGFRQRVHEQLSRAKDANK 20
gi|Aspergillus_nidulans_parA      ------------------------------MKGFRQRVHEQLSRAKDNKS 20
gi|Mus_musculus_alpha             --------------------------------------------------
gi|Rattus_norvegicus_alpha        --------------------------------------------------
gi|Macaca_mulatta_alpha           --------------------------------------------------
gi|Homo_sapiens_alpha             --------------------------------------------------
gi|Ovis_aries_alpha               --------------------------------------------------
gi|Bos_taurus_alpha               --------------------------------------------------
gi|Felis_catus_alpha              --------------------------------------------------
gi|Canis_lupus_familiaris_alph    --------------------------------------------------
gi|Falco_peregrinus_alpha         --------------------------------------------------
gi|Gallus_gallus_alpha            --------------------------------------------------
gi|Alligator_mississippiensis_    --------------------------------------------------
gi|Chrysemys_picta_bellii_alph    --------------------------------------------------
gi|Xenopus_laevis_alpha           --------------------------------------------------
gi|Xenopus_tropicalis_alpha       --------------------------------------------------
gi|Ambystoma_mexicanum_alpha      --------------------------------------------------
gi|Danio_rerio_alpha              --------------------------------------------------
gi|Petromyzon_marinus_S4RHV1      --------------------------------------------------
gi|Mus_musculus_epsilon           --------------------------------------------------
gi|Rattus_norvegicus_epsilon      --------------------------------------------------
gi|Macaca_mulatta_epsilon         --------------------------------------------------
gi|Homo_sapiens_epsilon           --------------------------------------------------
gi|Ovis_arie_epsilon              --------------------------------------------------
```

Figure S1. *Cont.*

```
gi|Bos_taurus_epsilon               --------------------------------------------------
gi|Canis_lupus_familiaris_epsi      --------------------------------------------------
gi|Falco_peregrinus_epsilon         --------------------------------------------------
gi|Gallus_gallus_epsilon            --------------------------------------------------
gi|Felis_catus_epsilon              --------------------------------------------------
gi|Chrysemys_picta_bellii_epsi      --------------------------------------------------
gi|Xenopus_laevis_epsilon           --------------------------------------------------
gi|Xenopus_tropicalis_epsilon       --------------------------------------------------
gi|Alligator_mississippiensis_      --------------------------------------------------
gi|Ambystoma_mexicanum_epsilon      --------------------------------------------------
gi|Danio_rerio_epsilon              --------------------------------------------------
gi|Mus_musculus_beta                --------------------------------------------------
gi|Rattus_norvegicus_beta           --------------------------------------------------
gi|Felis_catus_beta                 --------------------------------------------------
gi|Canis_lupus_familiaris_beta      --------------------------------------------------
gi|Homo_sapiens_beta                --------------------------------------------------
gi|Bos_taurus_beta                  --------------------------------------------------
gi|Ovis_aries_beta                  --------------------------------------------------
gi|Macaca_mulatta_beta              --------------------------------------------------
gi|Xenopus_laevis_beta              --------------------------------------------------
gi|Alligator_mississippiensis_      --------------------------------------------------
gi|Chrysemys_picta_bellii_beta      --------------------------------------------------
gi|Xenopus_tropicalis_beta          --------------------------------------------------
gi|Danio_rerio_beta                 --------------------------------------------------
gi|Ambystoma_mexicanum_beta         --------------------------------------------------
gi|Petromyzon_marinus_S4RGA7        --------------------------------------------------
gi|Strongylocentrotus_purpurat      --------------------------------------------------
gi|Drosophila_melanogaster_wdb      --------------------------------------------------
jgi|Branchiostoma_floridae_237      --------------------------------------------------
gi|Hydra_vulgaris_alpha             --------------------------------------------------
jgi|Branchiostoma_floridae_112      --------------------------------------------------
jgi|Branchiostoma_floridae_284      --------------------------------------------------
gi|Caenorhabditis_elegans_PPTR      --------------------------------------------------
gi|Amphimedon_queenslandica_be      --------------------------------------------------
gi|Chrysemys_picta_bellii_gamm      --------------------------------------------------
gi|Chrysemys_picta_bellii_delt      --------------------------------------------------
gi|Falco_peregrinus_gamma           --------------------------------------------------
gi|Falco_peregrinus_delta/gamm      --------------------------------------------------
gi|Gallus_gallus_gamma              --------------------------------------------------
gi|Alligator_mississippiensis_      --------------------------------------------------
gi|Alligator_mississippiensis_      --------------------------------------------------
gi|Ambystoma_mexicanum_gamma        --------------------------------------------------
gi|Mus_musculus_gamma               --------------------------------------------------
gi|Rattus_norvegicus_gamma          --------------------------------------------------
gi|Ovis_aries_gamma                 --------------------------------------------------
gi|Bos_taurus_gamma                 --------------------------------------------------
gi|Canis_lupus_familiaris_gamm      --------------------------------------------------
gi|Macaca_mulatta_gamma             --------------------------------------------------
gi|Homo_sapiens_gamma               --------------------------------------------------
gi|Felis_catus_gamma                --------------------------------------------------
gi|Canis_lupus_familiaris_delt      --------------------------------------------------
gi|Macaca_mulatta_delta/gamma       EERGCTSGRDLAEGAAEGGANGERRGCRVCGTSPEELRRAGPTRKGRGCR 200
gi|Bos_taurus_delta/gamma           --------------------------------------------------
gi|Mus_musculus_delta/gamma         --------------------------------------------------
gi|Rattus_norvegicus_delta/gam      --------MPASSGRGRRQVVQARGRAQGAGRIAWSWGGRPGSEVALRCR 42
gi|Danio_rerio_gamma                --------------------------------------------------
gi|Danio_rerio_delta/gamma          --------------------------------------------------
gi|Xenopus_laevis_delta/gamma       --------------------------------------------------
gi|Xenopus_laevis_gamma             --------------------------------------------------
gi|Xenopus_tropicalis_delta/ga      --------------------------------------------------
gi|Mus_musculus_delta               --------------------------------------------------
```

Figure S1. *Cont.*

```
gi|Rattus_norvegicus_delta          --------------------------------------------------
gi|Felis_catus_delta                --------------------------------------------------
gi|Bos_taurus_delta                 --------------------------------------------------
gi|Canis_lupus_familiaris_delt      --------------------------------------------------
gi|Macaca_mulatta_delta             --------------------------------------------------
gi|Homo_sapiens_delta               --------------------------------------------------
gi|Ovis_aries_delta                 --------------------------------------------------
gi|Falco_peregrinus_delta           --------------------------------------------------
gi|Gallus_gallus_delta              --------------------------------------------------
gi|Alligator_mississippiensis_      --------------------------------------------------
gi|Chrysemys_picta_bellii_delt      --------------------------------------------------
gi|Danio_rerio_delta                --------------------------------------------------
gi|Ambystoma_mexicanum_delta        --------------------------------------------------
gi|Petromyzon_marinus_S4RN43        --------------------------------------------------
jgi|Branchiostoma_floridae_237      --------------------------------------------------
jgi|Branchiostoma_floridae_252      --------------------------------------------------
gi|Strongylocentrotus_purpurat      --------------------------------------------------
gi|Caenorhabditis_elegans_PPTR      --------------------------------------------------
gi|Hydra_vulgaris_delta             ----------------------------------------------MPV- 3
gi|Amphimedon_queenslandica_de      ---------------------------------------------MMPIG 5
gi|Drosophila_melanogaster_B56      --MDNEALDPTIKSSTSAATPTAAASETTTTAASSVVETTTTIAAATASA 48
gi|Dictyostelium_discoideum_ps      --------------------------------------------------
gi|Dictyostelium_purpureum_N/A      --------------------------------------------------
gi|Dictyostelium_fasciculatum_      --------------------------------------------------
gi|Polysphondylium_pallidum_N/      --------------------------------------------------
gi|Arabidopsis_thaliana_theta       --------------------------------------------------
gi|Arabidopsis_thaliana_eta         --------------------------------------------------
gi|Arabidopsis_thaliana_zeta        --------------------------------------------------
gi|Arabidopsis_thaliana_gamma       --------------------------------------------------
gi|Oryza                            --------------------------------------------------
gi|Oryza_sativa_zeta                --------------------------------------------------
gi|Oryza_sativa_theta               --------------------------------------------------
gi|Oryza_sativa_eta                 --------------------------------------------------
gi|Arabidopsis_thaliana_delta       --------------------------------------------------
gi|Arabidopsis_thaliana_N/A         --------------------------------------------------
gi|Oryza_sativa_kappa               --------------------------------------------------
gi|Oryza_sativa_N/A                 --------------------------------------------------
gi|Arabidopsis_thaliana_alpha       --------------------------------------------------
gi|Arabidopsis_thaliana_beta        --------------------------------------------------
gi|Oryza_sativa_N/A_2               --------------------------------------------------
gi|Arabidopsis_thaliana_epsilo      --------------------------------------------------
gi|Chlamydomonas_reinhardtii_w      --------------------------------------------------
gi|Schizosaccharomyces_pombe_P      ---------MKGIKSKMLSRGKSQDTQKSSKKKESK-------------- 27
gi|Schizosaccharomyces_pombe_P      ---------MKGLRSKFVKALSLKDEQGSHKNGHSKSHYISKNGSYVETD 41
gi|Ashbya_gossypii_RTS1             ----------TGKKPGSANGAAG-----AVGGSGGRATVDKKEQKGSGAK 62
gi|Saccharomyces_cerevisiae_RT      EKEKSSTTSSTSKKPASASSSSHGTTHSSASSTGSKSTTEKGKQSGSVPS 79

gi|Aspergillus_niger_N/A            SSKKKDSSHSSQ-NNAALGVHHGQQSSSPNQGTPTSSTTSVNDTRGKSPD 69
gi|Aspergillus_nidulans_parA        SKKKESASQASQQNSANLGIHHGQQSASPNQVTPTSSTTSVNDIRGKAPE 70
gi|Mus_musculus_alpha               --------------------------------------------------
gi|Rattus_norvegicus_alpha          --------------------------------------------------
gi|Macaca_mulatta_alpha             --------------------------------------------------
gi|Homo_sapiens_alpha               --------------------------------------------------
gi|Ovis_aries_alpha                 --------------------------------------------------
gi|Bos_taurus_alpha                 --------------------------------------------------
gi|Felis_catus_alpha                --------------------------------------------------
gi|Canis_lupus_familiaris_alph      --------------------------------------------------
gi|Falco_peregrinus_alpha           --------------------------------------------------
gi|Gallus_gallus_alpha              --------------------------------------------------
gi|Alligator_mississippiensis_      --------------------------------------------------
```

Figure S1. *Cont.*

```
gi|Chrysemys_picta_bellii_alph      --------------------------------------------------
gi|Xenopus_laevis_alpha             --------------------------------------------------
gi|Xenopus_tropicalis_alpha         --------------------------------------------------
gi|Ambystoma_mexicanum_alpha        --------------------------------------------------
gi|Danio_rerio_alpha                --------------------------------------------------
gi|Petromyzon_marinus_S4RHV1        --------------------------------------------------
gi|Mus_musculus_epsilon             --------------------------------------------------
gi|Rattus_norvegicus_epsilon        --------------------------------------------------
gi|Macaca_mulatta_epsilon           --------------------------------------------------
gi|Homo_sapiens_epsilon             --------------------------------------------------
gi|Ovis_arie_epsilon                --------------------------------------------------
gi|Bos_taurus_epsilon               --------------------------------------------------
gi|Canis_lupus_familiaris_epsi      --------------------------------------------------
gi|Falco_peregrinus_epsilon         --------------------------------------------------
gi|Gallus_gallus_epsilon            --------------------------------------------------
gi|Felis_catus_epsilon              --------------------------------------------------
gi|Chrysemys_picta_bellii_epsi      --------------------------------------------------
gi|Xenopus_laevis_epsilon           --------------------------------------------------
gi|Xenopus_tropicalis_epsilon       --------------------------------------------------
gi|Alligator_mississippiensis_      --------------------------------------------------
gi|Ambystoma_mexicanum_epsilon      --------------------------------------------------
gi|Danio_rerio_epsilon              --------------------------------------------------
gi|Mus_musculus_beta                --------------------------------------------------
gi|Rattus_norvegicus_beta           --------------------------------------------------
gi|Felis_catus_beta                 --------------------------------------------------
gi|Canis_lupus_familiaris_beta      --------------------------------------------------
gi|Homo_sapiens_beta                --------------------------------------------------
gi|Bos_taurus_beta                  --------------------------------------------------
gi|Ovis_aries_beta                  --------------------------------------------------
gi|Macaca_mulatta_beta              --------------------------------------------------
gi|Xenopus_laevis_beta              --------------------------------------------------
gi|Alligator_mississippiensis_      --------------------------------------------------
gi|Chrysemys_picta_bellii_beta      --------------------------------------------------
gi|Xenopus_tropicalis_beta          --------------------------------------------------
gi|Danio_rerio_beta                 --------------------------------------------------
gi|Ambystoma_mexicanum_beta         --------------------------------------------------
gi|Petromyzon_marinus_S4RGA7        --------------------------------------------------
gi|Strongylocentrotus_purpurat      --------------------------------------------------
gi|Drosophila_melanogaster_wdb      --------------------------------------------------
jgi|Branchiostoma_floridae_237      --------------------------------------------------
gi|Hydra_vulgaris_alpha             --------------------------------------------------
jgi|Branchiostoma_floridae_112      --------------------------------------------------
jgi|Branchiostoma_floridae_284      --------------------------------------------------
gi|Caenorhabditis_elegans_PPTR      --------------------------------------------------
gi|Amphimedon_queenslandica_be      --------------------------------------------------
gi|Chrysemys_picta_bellii_gamm      -------------------------------------------MLTCNKA 7
gi|Chrysemys_picta_bellii_delt      --------------------------------------------------
gi|Falco_peregrinus_gamma           -------------------------------------------MLTCNRA 7
gi|Falco_peregrinus_delta/gamm      --------------------------------------------------
gi|Gallus_gallus_gamma              -------------------------------------------MLTCNRA 7
gi|Alligator_mississippiensis_      -------------------------------------------MLTCNKA 7
gi|Alligator_mississippiensis_      --------------------------------------------------
gi|Ambystoma_mexicanum_gamma        -------------------------------------------MLTCNQV 7
gi|Mus_musculus_gamma               -------------------------------------------MLTCNKA 7
gi|Rattus_norvegicus_gamma          -------------------------------------------MLTCNKA 7
gi|Ovis_aries_gamma                 -------------------------------------------MLTCNKA 7
gi|Bos_taurus_gamma                 -------------------------------------------MLTCNKA 7
gi|Canis_lupus_familiaris_gamm      -------------------------------------------MWTCNKA 7
gi|Macaca_mulatta_gamma             -------------------------------------------MLTCNKA 7
gi|Homo_sapiens_gamma               --------------------------------------------------
gi|Felis_catus_gamma                ----------------------------------MAESPKAGKSGKSSKE 16
```

Figure S1. *Cont.*

```
gi|Canis_lupus_familiaris_delt      ----------------------------------MAESPKAGKSGKSSKE 16
gi|Macaca_mulatta_delta/gamma       VGGTLREKLRRAGPTREGRACRMGGTSREDLRTVELESPKAGKSGKSSKE 250
gi|Bos_taurus_delta/gamma           ---------------------------MPNKNKKEKESPKAGKSGKSSKE 23
gi|Mus_musculus_delta/gamma         ---------------------------MPNKNKKEKEPPKPVKSGKGPKE 23
gi|Rattus_norvegicus_delta/gam      GRSGRRRMLRQCSAWRGLGCWSVQDSPQKPSLGGLREPPKPVKSGKGSKE 92
gi|Danio_rerio_gamma                ---------------------------MLTCSKDE------ARMVLDAPS 17
gi|Danio_rerio_delta/gamma          ---------------------------MPNKTKKDKDSPKSAKVGKTAGQ 23
gi|Xenopus_laevis_delta/gamma       ---------------------------MPNKNKKDKEPPKAGKSGKSGKE 23
gi|Xenopus_laevis_gamma             ---------------------------MPNKNKKDKEPPKAGKSGKSGKE 23
gi|Xenopus_tropicalis_delta/ga      ---------------------------MPNKNKKDKEPPKAGKSGKTGKE 23
gi|Mus_musculus_delta               ---------------------------MSYKLKKDKE-PSKLAKGTAKPS 22
gi|Rattus_norvegicus_delta          AERAECGRAEPEPERGRRSRAGCGPAEMPYKLKKDKE-PPKLAKGTAKPS 49
gi|Felis_catus_delta                ---------------------GSGRAEMPYKLKKEKE-TPKLAKGTTKPS 28
gi|Bos_taurus_delta                 ---------------------------MPYKLKKEKE-PPKLAKGTAKPS 22
gi|Canis_lupus_familiaris_delt      ---------------------------MPYKLKKEKVSPPKLAKGTAKPS 23
gi|Macaca_mulatta_delta             ---------------------------MPYKLKKEKE-PPKVAKCTAKPT 22
gi|Homo_sapiens_delta               ---------------------------MPYKLKKEKE-PPKVAKCTAKPS 22
gi|Ovis_aries_delta                 -----------------------MAEDNAHQTPKFKE-PPKLAKGTAKPS 26
gi|Falco_peregrinus_delta           --------------------------------------------------
gi|Gallus_gallus_delta              ---------------------------MP---SVGEE-SPKSAKSTTKPG 19
gi|Alligator_mississippiensis_      ---------------------------MPYKLKKEKE-PPKSAKSTTKSS 22
gi|Chrysemys_picta_bellii_delt      ---------------------------MPYKLKKEKE-PPKSAKSTTKPS 22
gi|Danio_rerio_delta                ---------------------------MPNKTKKEKE-PTKSAKSSTKNS 22
gi|Ambystoma_mexicanum_delta        --------------------------------------------------
gi|Petromyzon_marinus_S4RN43        ---------------------------MPNKNKKDKEPAKAAKSAKNSPK 23
jgi|Branchiostoma_floridae_237      --------------------------------------------------
jgi|Branchiostoma_floridae_252      -------------------------------------QANRNRAATNN-- 11
gi|Strongylocentrotus_purpurat      -------------------MSRKKPGEWISALIVSKHRPGNVPPPTQLTR 31
gi|Caenorhabditis_elegans_PPTR      ------------------------------------MIPADAPPPTNIGR 14
gi|Hydra_vulgaris_delta             ---KLKKDKEKNSEKKDKGKEE--------ENKGKANLPPQVPVVKKAGP 42
gi|Amphimedon_queenslandica_de      KGKKAEKEKEKAQNKNTSAKPSSESPQNVPATTGDNNVPAPTPVTTGVGV 55
gi|Drosophila_melanogaster_B56      AESKNETTATNNNSNTSGSISSSSSNNIVIPASATNGIKESNSNLSTTTT 98
gi|Dictyostelium_discoideum_ps      ------------------------------MKNDHINYQQNLSQSPILNS 20
gi|Dictyostelium_purpureum_N/A      ------------------------------MKNDHIN------------- 7
gi|Dictyostelium_fasciculatum_      ------------------------------MTSPHIK------------- 7
gi|Polysphondylium_pallidum_N/      ------------------------------MKSPKES------------- 7
gi|Arabidopsis_thaliana_theta       ------------------------------MWKQILSKLPKK-------- 12
gi|Arabidopsis_thaliana_eta         ------------------------------MWKQILSKLPNK-------- 12
gi|Arabidopsis_thaliana_zeta        ------------------------------MIKQIFGKLPR--------- 11
gi|Arabidopsis_thaliana_gamma       ------------------------------MIKQIFGKLPR--------- 11
gi|Oryza                            ------------------------------MMKQIFGR--R--------- 9
gi|Oryza_sativa_zeta                ------------------------------MIKQILGRFPK--------- 11
gi|Oryza_sativa_theta               ------------------------------MIKQILGRLPKK-------- 12
gi|Oryza_sativa_eta                 ------------------------------MIKQILGRLPR--------- 11
gi|Arabidopsis_thaliana_delta       ------------------------------MFKQILGKLPK--------- 11
gi|Arabidopsis_thaliana_N/A         ------------------------------MFKQFLSKLPR--------- 11
gi|Oryza_sativa_kappa               ------------------------------MWKGFLSKLPR--------- 11
gi|Oryza_sativa_N/A                 ------------------------------MWKQFLGKISW--------- 11
gi|Arabidopsis_thaliana_alpha       ------------------------------MFKKIMKGANR--------- 11
gi|Arabidopsis_thaliana_beta        ------------------------------MFKKIMKGGHR--------- 11
gi|Oryza_sativa_N/A_2               ------------------------------MFNKIIKRGGR--------- 11
gi|Arabidopsis_thaliana_epsilo      ------------------------------MFNKIIKLGQK--------- 11
gi|Chlamydomonas_reinhardtii_w      ------------------------------MLKSMKQKFSG--------- 11
gi|Schizosaccharomyces_pombe_P      -----------------------------KSNSHDSSKAPKESPS----- 43
gi|Schizosaccharomyces_pombe_P      DVKHTDTHHSSKHELKKLKSHFLKDTLKHKRNHHANSNNEKHENSDKKIH 91
gi|Ashbya_gossypii_RTS1             Q--------PKAKDAGK-------------SGAGAAPKVKVAAKK----- 86
gi|Saccharomyces_cerevisiae_RT      QGKHHSSSTSKTKTATTPSSSSSSSRSSSVSRSGSSSTKKTSSRKGQEQS 129

gi|Aspergillus_niger_N/A            NGSQAG---------------------------MYPGTPTKQGQQMAPSV 92
gi|Aspergillus_nidulans_parA        DASQAGG--------------------------FPPATPTKQGQPMAPSV 94
```

Figure S1. *Cont.*

```
gi|Mus_musculus_alpha              --------------------------------------------------
gi|Rattus_norvegicus_alpha         --------------------------------------------------
gi|Macaca_mulatta_alpha            --------------------------------------------------
gi|Homo_sapiens_alpha              --------------------------------------------------
gi|Ovis_aries_alpha                --------------------------------------------------
gi|Bos_taurus_alpha                --------------------------------------------------
gi|Felis_catus_alpha               --------------------------------------------------
gi|Canis_lupus_familiaris_alph     --------------------------------------------------
gi|Falco_peregrinus_alpha          --------------------------------------------------
gi|Gallus_gallus_alpha             --------------------------------------------------
gi|Alligator_mississippiensis_     --------------------------------------------------
gi|Chrysemys_picta_bellii_alph     --------------------------------------------------
gi|Xenopus_laevis_alpha            --------------------------------------------------
gi|Xenopus_tropicalis_alpha        --------------------------------------------------
gi|Ambystoma_mexicanum_alpha       --------------------------------------------------
gi|Danio_rerio_alpha               --------------------------------------------------
gi|Petromyzon_marinus_S4RHV1       --------------------------------------------------
gi|Mus_musculus_epsilon            --------------------------------------------------
gi|Rattus_norvegicus_epsilon       --------------------------------------------------
gi|Macaca_mulatta_epsilon          --------------------------------------------------
gi|Homo_sapiens_epsilon            --------------------------------------------------
gi|Ovis_arie_epsilon               --------------------------------------------------
gi|Bos_taurus_epsilon              --------------------------------------------------
gi|Canis_lupus_familiaris_epsi     --------------------------------------------------
gi|Falco_peregrinus_epsilon        --------------------------------------------------
gi|Gallus_gallus_epsilon           --------------------------------------------------
gi|Felis_catus_epsilon             --------------------------------------------------
gi|Chrysemys_picta_bellii_epsi     --------------------------------------------------
gi|Xenopus_laevis_epsilon          --------------------------------------------------
gi|Xenopus_tropicalis_epsilon      --------------------------------------------------
gi|Alligator_mississippiensis_     --------------------------------------------MESAQE 6
gi|Ambystoma_mexicanum_epsilon     --------------------------------------------------
gi|Danio_rerio_epsilon             --------------------------------------------------
gi|Mus_musculus_beta               --------------------------------------------------
gi|Rattus_norvegicus_beta          --------------------------------------------------
gi|Felis_catus_beta                --------------------------------------------------
gi|Canis_lupus_familiaris_beta     --------------------------------------------------
gi|Homo_sapiens_beta               --------------------------------------------------
gi|Bos_taurus_beta                 --------------------------------------------------
gi|Ovis_aries_beta                 --------------------------------------------------
gi|Macaca_mulatta_beta             --------------------------------------------------
gi|Xenopus_laevis_beta             --------------------------------------------------
gi|Alligator_mississippiensis_     --------------------------------------------------
gi|Chrysemys_picta_bellii_beta     --------------------------------------------------
gi|Xenopus_tropicalis_beta         --------------------------------------------------
gi|Danio_rerio_beta                --------------------------------------------------
gi|Ambystoma_mexicanum_beta        --------------------------------------------------
gi|Petromyzon_marinus_S4RGA7       --------------------------------------------------
gi|Strongylocentrotus_purpurat     --------------------------------------------------
gi|Drosophila_melanogaster_wdb     --------------------------------------------------
jgi|Branchiostoma_floridae_237     --------------------------------------------------
gi|Hydra_vulgaris_alpha            --------------------------------------------------
jgi|Branchiostoma_floridae_112     --------------------------------------------------
jgi|Branchiostoma_floridae_284     --------------------------------------------------
gi|Caenorhabditis_elegans_PPTR     ------------------------------------------MHGSGHSL 8
gi|Amphimedon_queenslandica_be     --------------------------------------------------
gi|Chrysemys_picta_bellii_gamm     G------------------------------------------------- 8
gi|Chrysemys_picta_bellii_delt     --------------------------------------------------
gi|Falco_peregrinus_gamma          G------------------------------------------------- 8
gi|Falco_peregrinus_delta/gamm     --------------------------------------------------
gi|Gallus_gallus_gamma             G------------------------------------------------- 8
```

Figure S1. *Cont.*

```
gi|Alligator_mississippiensis_      G------------------------------------------------- 8
gi|Alligator_mississippiensis_      --------------------------------------------------
gi|Ambystoma_mexicanum_gamma        G------------------------------------------------- 8
gi|Mus_musculus_gamma               G------------------------------------------------- 8
gi|Rattus_norvegicus_gamma          G------------------------------------------------- 8
gi|Ovis_aries_gamma                 G------------------------------------------------- 8
gi|Bos_taurus_gamma                 G------------------------------------------------- 8
gi|Canis_lupus_familiaris_gamm      G------------------------------------------------- 8
gi|Macaca_mulatta_gamma             G------------------------------------------------- 8
gi|Homo_sapiens_gamma               --------------------------------------------------
gi|Felis_catus_gamma                GQDII--------------------------------------------- 21
gi|Canis_lupus_familiaris_delt      GQDII--------------------------------------------- 21
gi|Macaca_mulatta_delta/gamma       GQDTV--------------------------------------------- 255
gi|Bos_taurus_delta/gamma           GQDII--------------------------------------------- 28
gi|Mus_musculus_delta/gamma         GQDTA--------------------------------------------- 28
gi|Rattus_norvegicus_delta/gam      GQDTA--------------------------------------------- 97
gi|Danio_rerio_gamma                AN------------------------------------------------ 19
gi|Danio_rerio_delta/gamma          ENSEH--------------------------------------------- 28
gi|Xenopus_laevis_delta/gamma       GQDNP--------------------------------------------- 28
gi|Xenopus_laevis_gamma             GQDNP--------------------------------------------- 28
gi|Xenopus_tropicalis_delta/ga      GQDNP--------------------------------------------- 28
gi|Mus_musculus_delta               SSSK------------------------------DGGGENTD-EAQPQPQ 41
gi|Rattus_norvegicus_delta          SSSK------------------------------DGGGENTDTEAQPQPQ 69
gi|Felis_catus_delta                SSGK------------------------------DGGGESAE-EAQPQPQ 47
gi|Bos_taurus_delta                 SSSK------------------------------DGGGESTE-EAQPQPQ 41
gi|Canis_lupus_familiaris_delt      SSGK------------------------------DGGGESTD-EPQPQPQ 42
gi|Macaca_mulatta_delta             SSGK------------------------------DGGGENTE-EAQPQPQ 41
gi|Homo_sapiens_delta               SSGK------------------------------DGGGENTE-EAQPQPQ 41
gi|Ovis_aries_delta                 SSSK------------------------------DGGGESTE-EPQPPPQ 45
gi|Falco_peregrinus_delta           --------------------------------------------------
gi|Gallus_gallus_delta              GGSG-----------------------------KDGGAENSEEAQQPQQQ 40
gi|Alligator_mississippiensis_      GGSX--G--------------------------KDGGAENSEEVQQ---- 40
gi|Chrysemys_picta_bellii_delt      SGSSGGG--------------------------KDGGAENSEEVQQ---- 42
gi|Danio_rerio_delta                NASAG----------------------------KDVGTENSEEAQQ---- 40
gi|Ambystoma_mexicanum_delta        --------------------------------------------------
gi|Petromyzon_marinus_S4RN43        DAADT--------------------------------------------- 28
jgi|Branchiostoma_floridae_237      --------------------------------------------------
jgi|Branchiostoma_floridae_252      --------------------------------------------------
gi|Strongylocentrotus_purpurat      T------------------------------------------------- 32
gi|Caenorhabditis_elegans_PPTR      TNT----------------------------------------------- 17
gi|Hydra_vulgaris_delta             --------------------------------------------------
gi|Amphimedon_queenslandica_de      GLPAK--------------------------------------------- 60
gi|Drosophila_melanogaster_B56      AAAVAAA--------------------------TTVEGVAPAITSTIVVT 122
gi|Dictyostelium_discoideum_ps      NKNQTQQ--------------------------NQQQQQQQQQQNPQQQQ 44
gi|Dictyostelium_purpureum_N/A      --------------------------------------AQQVSPNLKHQQ 19
gi|Dictyostelium_fasciculatum_      --------------------------------------PKQGSDDPISPS 19
gi|Polysphondylium_pallidum_N/      --------------------------------------ASGVAGSPTSPS 19
gi|Arabidopsis_thaliana_theta       ----------------------------------------SSSK------ 16
gi|Arabidopsis_thaliana_eta         ----------------------------------------KSSKHEHRGR 22
gi|Arabidopsis_thaliana_zeta        -----------------------------------------KPSKSLQND 20
gi|Arabidopsis_thaliana_gamma       -----------------------------------------KPSKSSHND 20
gi|Oryza                            -----------------------------------------KNAKSADKD 18
gi|Oryza_sativa_zeta                -----------------------------------------KPSKSGDKD 20
gi|Oryza_sativa_theta               ----------------------------------------EKPGKSGE-K 21
gi|Oryza_sativa_eta                 -----------------------------------------KPGKAGDSR 20
gi|Arabidopsis_thaliana_delta       --------------------------------------------------
gi|Arabidopsis_thaliana_N/A         -----------------------------------------KSSKSDSG- 19
gi|Oryza_sativa_kappa               -----------------------------------------KTSASGRGA 20
gi|Oryza_sativa_N/A                 -----------------------------------------KAPKSGGG- 19
gi|Arabidopsis_thaliana_alpha       -----------------------------------------KASKAEAND 20
gi|Arabidopsis_thaliana_beta        -----------------------------------------KPSKSEANE 20
```

Figure S1. *Cont.*

```
gi|Oryza_sativa_N/A_2                -----------------------------------------KGQRAADGG 20
gi|Arabidopsis_thaliana_epsilo       -----------------------------------------KFNKSDQHH 20
gi|Chlamydomonas_reinhardtii_w       -----------------------------------------KKDASKNKD 20
gi|Schizosaccharomyces_pombe_P       -----------------------------------TDP-NGSVIG----- 52
gi|Schizosaccharomyces_pombe_P       TTVLASGHEDSDYSTFLPVIETSKVKDANHFPPNYPEPKNDSVSSNIDEF 141
gi|Ashbya_gossypii_RTS1              ---------------------------------------EERSYPTMIAA 97
gi|Saccharomyces_cerevisiae_RT       KQSQQPSQSQKQGSSSSSAAIMNPTPVLTVTKDDKSTSGEDHAHPTLLGA 179


gi|Aspergillus_niger_N/A             VISPSA-----------------------------PHVPPPGAAETMPGD 113
gi|Aspergillus_nidulans_parA         VISPSG-----------------------------PHAPPPGAAETMPGD 115
gi|Mus_musculus_alpha                --------------------------------------MSSPSPP---AP 9
gi|Rattus_norvegicus_alpha           --------------------------------------MSSPSPP---AP 9
gi|Macaca_mulatta_alpha              --------------------------------------MSSSSPP---AG 9
gi|Homo_sapiens_alpha                --------------------------------------MSSSSPP---AG 9
gi|Ovis_aries_alpha                  --------------------------------------MSSSSSPPPPAG 12
gi|Bos_taurus_alpha                  --------------------------------------MSSSSSPPPPAG 12
gi|Felis_catus_alpha                 --------------------------------------------------
gi|Canis_lupus_familiaris_alph       --------------------------------------------------
gi|Falco_peregrinus_alpha            --------------------------------------------------
gi|Gallus_gallus_alpha               --------------------------------------------------
gi|Alligator_mississippiensis_       -----------------------------------------AAAAPLAAM 9
gi|Chrysemys_picta_bellii_alph       --------------------------------------------------
gi|Xenopus_laevis_alpha              --------------------------------------------------
gi|Xenopus_tropicalis_alpha          --------------------------------------------------
gi|Ambystoma_mexicanum_alpha         --------------------------------------------------
gi|Danio_rerio_alpha                 --------------------------------------------------
gi|Petromyzon_marinus_S4RHV1         --------------------------------------------------
gi|Mus_musculus_epsilon              ------------------------------------------------MS 2
gi|Rattus_norvegicus_epsilon         ------------------------------------------------MS 2
gi|Macaca_mulatta_epsilon            ------------------------------------------------MS 2
gi|Homo_sapiens_epsilon              ------------------------------------------------MS 2
gi|Ovis_arie_epsilon                 ------------------------------------------------MS 2
gi|Bos_taurus_epsilon                ------------------------------------------------MS 2
gi|Canis_lupus_familiaris_epsi       ------------------------------------------------MS 2
gi|Falco_peregrinus_epsilon          ------------------------------------------------MS 2
gi|Gallus_gallus_epsilon             ------------------------------------------------MS 2
gi|Felis_catus_epsilon               ------------------------------------------------MS 2
gi|Chrysemys_picta_bellii_epsi       ------------------------------------------------MS 2
gi|Xenopus_laevis_epsilon            ------------------------------------------------MS 2
gi|Xenopus_tropicalis_epsilon        ------------------------------------------------MS 2
gi|Alligator_mississippiensis_       YWNVKL-----------------------------EIRKEIHSVKAGDMS 27
gi|Ambystoma_mexicanum_epsilon       --------------------------------------------------
gi|Danio_rerio_epsilon               ------------------------------------------------MS 2
gi|Mus_musculus_beta                 -----M-----------------------------ETKLPPASTPTSPSS 16
gi|Rattus_norvegicus_beta            -----M-----------------------------ETKLPPASTPTSPSS 16
gi|Felis_catus_beta                  -----M-----------------------------ETKLPPASTPTSPSS 16
gi|Canis_lupus_familiaris_beta       -----M-----------------------------ETKLPPASTPTSPSS 16
gi|Homo_sapiens_beta                 -----M-----------------------------ETKLPPASTPTSPSS 16
gi|Bos_taurus_beta                   -----M-----------------------------ETKLPPASTPTSPSS 16
gi|Ovis_aries_beta                   -----M-----------------------------ETKLPPASTPTSPSS 16
gi|Macaca_mulatta_beta               -----M-----------------------------AQEHGGWGQQRLPWR 16
gi|Xenopus_laevis_beta               --------------------------------------------------
gi|Alligator_mississippiensis_       -----M-----------------------------EMKLTPAGTPPSPPS 16
gi|Chrysemys_picta_bellii_beta       -----M-----------------------------ETKLPPAGTPTSPSS 16
gi|Xenopus_tropicalis_beta           -----M-----------------------------ETKLP--VTPTSPSS 14
gi|Danio_rerio_beta                  -----M-----------------------------ETPTKLPPSSPSSPT 16
gi|Ambystoma_mexicanum_beta          --------------------------------------------------
gi|Petromyzon_marinus_S4RGA7         --------------------------------------------------
gi|Strongylocentrotus_purpurat       --------------------------------------------------
gi|Drosophila_melanogaster_wdb       --------------------------------------------------
```

Figure S1. *Cont.*

```
jgi|Branchiostoma_floridae_237       --------------------------------------------------
gi|Hydra_vulgaris_alpha              ----------------------------------------------MPHG 4
jgi|Branchiostoma_floridae_112       --------------------------------------------------
jgi|Branchiostoma_floridae_284       --------------------------------------------------
gi|Caenorhabditis_elegans_PPTR       TGAPHQ-----------------------------IPPPRTQGAATGGQQ 29
gi|Amphimedon_queenslandica_be       --MTSK-----------------------------ASKGLGKTPPKAGTP 19
gi|Chrysemys_picta_bellii_gamm       --------------------------------------------------
gi|Chrysemys_picta_bellii_delt       --------------------------------------------------
gi|Falco_peregrinus_gamma            --------------------------------------------------
gi|Falco_peregrinus_delta/gamm       --------------------------------------------------
gi|Gallus_gallus_gamma               --------------------------------------------------
gi|Alligator_mississippiensis_       --------------------------------------------------
gi|Alligator_mississippiensis_       --------------------------------------------------
gi|Ambystoma_mexicanum_gamma         --------------------------------------------------
gi|Mus_musculus_gamma                --------------------------------------------------
gi|Rattus_norvegicus_gamma           --------------------------------------------------
gi|Ovis_aries_gamma                  --------------------------------------------------
gi|Bos_taurus_gamma                  --------------------------------------------------
gi|Canis_lupus_familiaris_gamm       --------------------------------------------------
gi|Macaca_mulatta_gamma              --------------------------------------------------
gi|Homo_sapiens_gamma                --------------------------------------------------
gi|Felis_catus_gamma                 ---------------------------------------ES-EIPGRKNN 31
gi|Canis_lupus_familiaris_delt       ---------------------------------------ES-ETSSRKNN 31
gi|Macaca_mulatta_delta/gamma        ---------------------------------------ESEQISVRKNS 266
gi|Bos_taurus_delta/gamma            ---------------------------------------DS-EIPSRKNS 38
gi|Mus_musculus_delta/gamma          ---------------------------------------ET-EIASRKNS 38
gi|Rattus_norvegicus_delta/gam       ---------------------------------------EA-EIASRKNS 107
gi|Danio_rerio_gamma                 --------------------------------------------------
gi|Danio_rerio_delta/gamma           ---------------------------------------EQASNKKGGNS 39
gi|Xenopus_laevis_delta/gamma        ---------------------------------------EHEVSNKKSNS 39
gi|Xenopus_laevis_gamma              ---------------------------------------EHEVSNKKSNS 39
gi|Xenopus_tropicalis_delta/ga       ---------------------------------------EHEVSNKKSNN 39
gi|Mus_musculus_delta                SQ-------------------------------------SPSSNKRPSNS 54
gi|Rattus_norvegicus_delta           PQ-------------------------------------PPSSNKRPSNS 82
gi|Felis_catus_delta                 PQPQPQ-----------------------------PQPQPPSSNKRPSNS 68
gi|Bos_taurus_delta                  PQPQPQ-----------------------------PQ--PPSSNKRPSNS 60
gi|Canis_lupus_familiaris_delt       PQ-------------------------------------PPSSNKRPSNS 55
gi|Macaca_mulatta_delta              PQPQPQ-----------------------------AQSQPPSSNKRPSNS 62
gi|Homo_sapiens_delta                PQPQPQ-----------------------------AQSQPPSSNKRPSNS 62
gi|Ovis_aries_delta                  PQ-------------------------------------PPSSNKRPSNS 58
gi|Falco_peregrinus_delta            --------------------------------------------------
gi|Gallus_gallus_delta               PQQQQQ---------------------------------QTPSNKRPSNS 57
gi|Alligator_mississippiensis_       -QQQQQ---------------------------------QTTSNKRPSNS 56
gi|Chrysemys_picta_bellii_delt       -QQQPP---------------------------------PPTSNKRPSNS 58
gi|Danio_rerio_delta                 -----------------------------------------TASKRPSNS 49
gi|Ambystoma_mexicanum_delta         --------------------------------------------------
gi|Petromyzon_marinus_S4RN43         ---------------------------------------DESVSERGGNK 39
jgi|Branchiostoma_floridae_237       --------------------------------------------------
jgi|Branchiostoma_floridae_252       --------------------------------------------------
gi|Strongylocentrotus_purpurat       --------------------------------------------------
gi|Caenorhabditis_elegans_PPTR       --------------------------------------------------
gi|Hydra_vulgaris_delta              --------------------------------------------------
gi|Amphimedon_queenslandica_de       -----------------------------------------DIDGINSDT 69
gi|Drosophila_melanogaster_B56       GGTPPL-----------------------------SSLANKLKDNTPPYD 143
gi|Dictyostelium_discoideum_ps       QFQHQQ-----------------------------VPQLSPQQIPFSEPL 65
gi|Dictyostelium_purpureum_N/A       TNTNQT-----------------------------VP-LSPQQIPFSEPL 39
gi|Dictyostelium_fasciculatum_       SPNMYL-----------------------------GNGVQQQQQQAPTSP 40
gi|Polysphondylium_pallidum_N/       -----------------------------------GKGIS---------- 24
gi|Arabidopsis_thaliana_theta        ---NHS-----------------------------SSSS-----STSKSS 29
gi|Arabidopsis_thaliana_eta          EHGGHS-----------------------------SSSSHTSGASTSKST 43
gi|Arabidopsis_thaliana_zeta         SN--GE-----------------------------GGVNN-SYYASNSST 38
```

Figure S1. *Cont.*

```
gi|Arabidopsis_thaliana_gamma       SNPNGE-----------------------------GGVN--SYYIPNSGI 39
gi|Oryza                            FFSGTS-----------------------------PSVLD-QVSGLGVAD 38
gi|Oryza_sativa_zeta                PIGRSS-----------------------------PSVPN-PPLGPRGAE 40
gi|Oryza_sativa_theta               DLAGVG-----------------------------SSLPDARTTTDLTMS 42
gi|Oryza_sativa_eta                 DAAGPN-----------------------------GNEP----SNSYSVA 37
gi|Arabidopsis_thaliana_delta       -------------------------------------------KTSAKFW 18
gi|Arabidopsis_thaliana_N/A         ELN--R-----------------------------SSSGP-VSSPVQRSG 37
gi|Oryza_sativa_kappa               DLDSGQ-----------------------------CSNGAGNGNPIQRTS 41
gi|Oryza_sativa_N/A                 -----------------------------------GGGSPTAKSPLSKGA 34
gi|Arabidopsis_thaliana_alpha       SSMYG---------------------------------FDPP-GRSGPGS 36
gi|Arabidopsis_thaliana_beta        PSSYG---------------------------------IGLPDNRSGPGS 37
gi|Oryza_sativa_N/A_2               DAAHGR-----------------------------PAAAAPSSSSGGAQP 41
gi|Arabidopsis_thaliana_epsilo      QDNNNN-------------------------------NNNTSTNTVVRGS 39
gi|Chlamydomonas_reinhardtii_w      D------------------------------------------SQPSTSA 28
gi|Schizosaccharomyces_pombe_P      -----A-----------------------------QNDFLTVPKHSGKKV 68
gi|Schizosaccharomyces_pombe_P      PNDSIS-----------------------------SASFLSVPQSTPPYL 162
gi|Ashbya_gossypii_RTS1             AVSPGS-----TSG--------------------PPSPKEAEAASPNGID 122
gi|Saccharomyces_cerevisiae_RT      VSAVPSSPISNASGTAVSSDVENGNSNNNNMNINTSNTQDANHASSQSID 229


gi|Aspergillus_niger_N/A            LAPPRKSHVFDRLQTTPKDMSEGIRTPKRQHSSRFDISDQRQ-------- 155
gi|Aspergillus_nidulans_parA        LAPPRKSHVFDRLQTTPKDMSEGIRTPKRQHSSRFDISDQRQ-------- 157
gi|Mus_musculus_alpha               VACAAISASEKVDGFTRKSVRKAQRQKRSQGSSQFRSQGSQ--------- 50
gi|Rattus_norvegicus_alpha          VACAAISASEKVDGFTRKSVRKAQRQKRSQGSSQFRSQGSQ--------- 50
gi|Macaca_mulatta_alpha             AASAAISASEKVDGFTRKSVRKAQRQKRSQGSSQFRSQGSQ--------- 50
gi|Homo_sapiens_alpha               AASAAISASEKVDGFTRKSVRKAQRQKRSQGSSQFRSQGSQ--------- 50
gi|Ovis_aries_alpha                 AASAAISAAEKVDGFTRKSVRKAQRQKRSQGSSQFRSQCSQ--------- 53
gi|Bos_taurus_alpha                 AASAAISAAEKVDGFTRKSVRKAQRQKRSQGSSQFRSQCSQ--------- 53
gi|Felis_catus_alpha                --------------------------------------------------
gi|Canis_lupus_familiaris_alph      --------------------------------------------------
gi|Falco_peregrinus_alpha           -MSAAISAAEKVDGFTRKSVRKAQRQKRSQGSSQFRSQSSQ--------- 40
gi|Gallus_gallus_alpha              -MSAAISAAEKVDGFTRKSVRKAQRQKRSQGSSQFRSQSSQ--------- 40
gi|Alligator_mississippiensis_      SAAAAISAAEKVDGFTRKSVRKAQRQKRSQGSSQFRSQSSQ--------- 50
gi|Chrysemys_picta_bellii_alph      -MSAAISASEKVDGFTRKSVRKAQRQKRSQGSSQFRSQSSQ--------- 40
gi|Xenopus_laevis_alpha             --MSAISAAEKVDGFTRKSVRKAQRQRRSQGSSQFLGQGPP--------- 39
gi|Xenopus_tropicalis_alpha         --MSAISAAEKVDGFTRKSVRKAQRRGRSQGSSQFRGQGPP--------- 39
gi|Ambystoma_mexicanum_alpha        --------------------------------------------------
gi|Danio_rerio_alpha                --MSAISASEKVDGFTRKSVRKAQRQRVCQGSSQFAVNSSRP-------- 40
gi|Petromyzon_marinus_S4RHV1        MMSATATAAEKVDGFSRRSLRRAGRARRSQGSSQFRTRAHL--------- 41
gi|Mus_musculus_epsilon             SAPTTPPSVDKVDGFSRKSVRKA-RQKRSQSSSQFRSQGKP--------- 42
gi|Rattus_norvegicus_epsilon        SAPTTPPSVDKVDGFSRKSVRKA-RQKRSQSSSQFRSQGKP--------- 42
gi|Macaca_mulatta_epsilon           SAPTTPPSVDKVDGFSRKSVRKA-RQKRSQSSSQFRSQGKP--------- 42
gi|Homo_sapiens_epsilon             SAPTTPPSVDKVDGFSRKSVRKA-RQKRSQSSSQFRSQGKP--------- 42
gi|Ovis_arie_epsilon                SAPTTPPSVDKVDGFSRKSVRKA-RQKRSQSSSQFRSQGKP--------- 42
gi|Bos_taurus_epsilon               SAPTTPPSVDKVDGFSRKSVRKA-RQKRSQSSSQFRSQGKP--------- 42
gi|Canis_lupus_familiaris_epsi      SAPTTPPSVDKVDGFSRKSVRKA-RQKRSQSSSQFRSQGKP--------- 42
gi|Falco_peregrinus_epsilon         SAPTTPPSVDKVDGFSRKSVRKA-RQKRSQSSSQFRSQGKP--------- 42
gi|Gallus_gallus_epsilon            SAPTTPPSVDKVDGFSRKSVRKA-RQKRSQSSSQFRSQGKP--------- 42
gi|Felis_catus_epsilon              SAPTTPPSVDKVDGFSRKSVRKA-RQKRSQSSSQFRSQGKP--------- 42
gi|Chrysemys_picta_bellii_epsi      SAPTTPPSVDKVDGFSRKSVRKA-RQKRSQSSSQFRSQGKP--------- 42
gi|Xenopus_laevis_epsilon           SAPTTPPSVDKVDGFSRKSVRKA-RQKRSQSSSQFRSQGKP--------- 42
gi|Xenopus_tropicalis_epsilon       SAPTTPPSVDKVDGFSRKSVRKA-RQKRSQSSSQFRSQGKP--------- 42
gi|Alligator_mississippiensis_      SAPTTPPSVDKVDGFSRKSVRKA-RQKRSQSSSQFRSQGKP--------- 67
gi|Ambystoma_mexicanum_epsilon      --QTTPPSVDKVDGFSRKSVRKA-RQKRSQSSSQFRSQGKP--------- 38
gi|Danio_rerio_epsilon              SAATTAPSVDKVDGFSRKSVRKA-RQKRAQSSSQFRSQDKP--------- 42
gi|Mus_musculus_beta                PGLSPVPPPDKVDGFSRRSLRRA-RPRRSHSSSQFRYQSNQ--------- 56
gi|Rattus_norvegicus_beta           PGLSPVPPPDKVDGFSRRSLRRA-RPRRSHSSSQFRYQSNQ--------- 56
gi|Felis_catus_beta                 PGLSPVPPPDKVDGFSRRSLRRA-RPRRSHSSSQFRYQSNQ--------- 56
gi|Canis_lupus_familiaris_beta      PGLSPVPPPDKVDGFSRRSLRRA-RPRRSHSSSQFRYQSNQ--------- 56
gi|Homo_sapiens_beta                PGLSPVPPPDKVDGFSRRSLRRA-RPRRSHSSSQFRYQSNQ--------- 56
gi|Bos_taurus_beta                  PGLSPVPPPDKVDGFSRRSLRRA-RPRRSHSSSQFRYQSNQ--------- 56
```

Figure S1. *Cont.*

```
gi|Ovis_aries_beta                 PGLSPVPPPDKVDGFSRRSLRRA-RPRDXEGSPLPRAAAAG--------- 56
gi|Macaca_mulatta_beta             LGGS-VPPRAGAAG------------------------GAG--------- 32
gi|Xenopus_laevis_beta             --------------------------------------------------
gi|Alligator_mississippiensis_     PGLSPVPPPEKVDGFSRRSLRRT-RPRRSHSSSQFRYQSSQ--------- 56
gi|Chrysemys_picta_bellii_beta     PGMSPVPPPDKVDGFSRRSLRRS-RQRRSHSSSQFRYQSNQ--------- 56
gi|Xenopus_tropicalis_beta         PGLSPVLAADKVDGFSRRSLRRS-RQRRSHSSSQFRYQSKQ--------- 54
gi|Danio_rerio_beta                TGFS-VPSAEKVDGFPRRSMRRA-RQRRSHSSSQFRYQSSQ--------- 55
gi|Ambystoma_mexicanum_beta        --------------------------------------------------
gi|Petromyzon_marinus_S4RGA7       --------------------------------------------------
gi|Strongylocentrotus_purpurat     -MSSGSTAVDKIDPFAKK-SLRKG--KQKKSQGSSQYRLS---------- 36
gi|Drosophila_melanogaster_wdb     -MSSG-TFVDRIDPFAKR-SLKK---KGKKSQGSSRYRNS---------- 34
jgi|Branchiostoma_floridae_237     --------------------------------------------------
gi|Hydra_vulgaris_alpha            ASAAQPSTKENVDPFTRKSSVKKIVKSQKKQQGSSRFKTT---------- 44
jgi|Branchiostoma_floridae_112     MSAATGPVTDKIDPFSKKQSVKKP--KQKRSQASSRYKAK---------- 38
jgi|Branchiostoma_floridae_284     --------------------MITP--KP---------------------- 6
gi|Caenorhabditis_elegans_PPTR     LSATANQFVDKIDPFHNKRGTSRR--LRINN--SSRYNVD---------- 65
gi|Amphimedon_queenslandica_be     PLGVQSSSLPVPDPFRKMSHINRK---NQQTQSSSRYNLKK--------- 57
gi|Chrysemys_picta_bellii_gamm     ---------------------------SRMVVDAAN-SNGP--------- 21
gi|Chrysemys_picta_bellii_delt     --------------------------------------------------
gi|Falco_peregrinus_gamma          ---------------------------SRMVVDAAN-SNGP--------- 21
gi|Falco_peregrinus_delta/gamm     --------------------------------------------------
gi|Gallus_gallus_gamma             ---------------------------SRMVVDAAN-SNGP--------- 21
gi|Alligator_mississippiensis_     ---------------------------SRMVVDAAN-SNGP--------- 21
gi|Alligator_mississippiensis_     --------------------------------------------------
gi|Ambystoma_mexicanum_gamma       ---------------------------SRMVVDAAGGSNGP--------- 22
gi|Mus_musculus_gamma              ---------------------------SGMVVDAAS-SNGP--------- 21
gi|Rattus_norvegicus_gamma         ---------------------------SGMVVDAAS-SNGP--------- 21
gi|Ovis_aries_gamma                ---------------------------SRMVVDAAN-SNGP--------- 21
gi|Bos_taurus_gamma                ---------------------------SRMVVDAAS-SNGP--------- 21
gi|Canis_lupus_familiaris_gamm     ---------------------------SRMVVDAAN-SNGP--------- 21
gi|Macaca_mulatta_gamma            ---------------------------SRMVVDAAN-SNGP--------- 21
gi|Homo_sapiens_gamma              -----------------------------MVVDAAN-SNGP--------- 11
gi|Felis_catus_gamma               LPATPSTVSSKIKVPAAQPIVR---RDKRQNSSRFS-ASNN--------- 68
gi|Canis_lupus_familiaris_delt     LAAPPSTVSSKIKVPVPQPIVK---KEKRQNSSRFN-ASNN--------- 68
gi|Macaca_mulatta_delta/gamma      LVAVPSTVSAKIKVPVSQPIVK---KDKRQNSSRFS-ASNN--------- 303
gi|Bos_taurus_delta/gamma          LIAAPSAASSKIKVPAPQPIVK---RDRRQNSSRLS-ASNN--------- 75
gi|Mus_musculus_delta/gamma        LTVVQSSTSTKIKVPIPQPVVVPVKKDKRQNSSRFN-ASNN--------- 78
gi|Rattus_norvegicus_delta/gam     LASVQSSTS-KIKVPVPQPVPVK--KDKRQNSSRFN-ASNN--------- 144
gi|Danio_rerio_gamma               -------------GPFQPVA------------------------------ 26
gi|Danio_rerio_delta/gamma         VPPATQLLKGKQSGSQTPVKKE-----KRPNSSRFS-LSNN--------- 74
gi|Xenopus_laevis_delta/gamma      TPPPAQVSKIKVPTPQAVVKKE-----KRQSSSRYN-VSNN--------- 74
gi|Xenopus_laevis_gamma            TPPPAQVSKIKVPTPQAVVKKE-----KRQSSSRYN-VSNN--------- 74
gi|Xenopus_tropicalis_delta/ga     TPPPAQVSKIKVPTPQAVVKKE-----KRQSSSRFN-VSNN--------- 74
gi|Mus_musculus_delta              TPPPTQLSKIKYSGGPQIVKKE-----RRQSSFPFN-LNKN--------- 89
gi|Rattus_norvegicus_delta         TPPPTQLSKIKYSGGPQIVKKE-----RRQSSSRFN-LSKN--------- 117
gi|Felis_catus_delta               TPPPTQLSKIKYSGGPQIVKKE-----RRQSSSRFN-LSKN--------- 103
gi|Bos_taurus_delta                TPPPTQLSKIKYSGGPQIVKKE-----RRQSSSRFN-LSKN--------- 95
gi|Canis_lupus_familiaris_delt     TPPPTQLSKIKYSGGPQIVKKE-----RRQSSSRFN-LSKN--------- 90
gi|Macaca_mulatta_delta            TPPPTQLSKIKYSGGPQIVKKE-----RRQSSSRFN-LSKN--------- 97
gi|Homo_sapiens_delta              TPPPTQLSKIKYSGGPQIVKKE-----RRQSSSRFN-LSKN--------- 97
gi|Ovis_aries_delta                TPPPTQLSKIKYSGGPQIVKKE-----RRQSSSRFN-LSKN--------- 93
gi|Falco_peregrinus_delta          --------------------------------------------------
gi|Gallus_gallus_delta             APPPTQLNKIKYSGGPQIVKKE-----RRHSSSRFN-LSKN--------- 92
gi|Alligator_mississippiensis_     APPPTQLNKIKYSGGPQIVKKE-----RRHSSSRFN-LSKN--------- 91
gi|Chrysemys_picta_bellii_delt     TPPPTQLNKIKYSGGPQIVKKE-----RRHSSSRFN-LTKN--------- 93
gi|Danio_rerio_delta               TPPPTQLNKIKYSGGPQIVKKE-----RRQSSSRFN-LSKN--------- 84
gi|Ambystoma_mexicanum_delta       --------------------------------------------------
gi|Petromyzon_marinus_S4RN43       PPPQTQLNKVKYGSPANIVKKE-----KRQSSSRFN-VTKN--------- 74
jgi|Branchiostoma_floridae_237     --------------------------------------------------
jgi|Branchiostoma_floridae_252     VPPPTQLTKIKLTGGQQHVVKK----DKRQSSSRFN-ISKN--------- 47
gi|Strongylocentrotus_purpurat     ------------KGTGPLVKK-----DKRQGSSRFN-ISKN--------- 55
```

Figure S1. *Cont.*

```
gi|Caenorhabditis_elegans_PPTR      ------------YGGGPVIPRR----ERRQSSSMFN-ISQN--------- 41
gi|Hydra_vulgaris_delta             --------------ASVQVLKR----DNRQSSSSFN-ISKN--------- 64
gi|Amphimedon_queenslandica_de      VPIAYNSIVSRLQGSGSYTHRT----NSRQGSSRFN-ISKN--------- 105
gi|Drosophila_melanogaster_B56      APPPTPISKVLNITGTPIVRKE-----KRQTSARYN-ASKN--------- 178
gi|Dictyostelium_discoideum_ps      KNNLTLQHQQQQQHQQLAGGQHGSLLRKSYSSRFHEKPQGE-------- 107
gi|Dictyostelium_purpureum_N/A      K-----------------AGQQHGSLLRKSYSSRFHEKPQGE-------- 64
gi|Dictyostelium_fasciculatum_      SKLLGQTPKSEA------IPLTKNTSLRRSASSRFHEKSKVE-------- 76
gi|Polysphondylium_pallidum_N/      -----LTPKSE-------IPIKNTINLRRSASSRFHEKSSSE-------- 54
gi|Arabidopsis_thaliana_theta       DNGASKSGNSQTQNAPPVKPSADSGFKEGNLKGNGNG------------- 66
gi|Arabidopsis_thaliana_eta         DNGAAKS-HAKNASPAGKSAASDSGFKDGNLKSSGNNNNNNNNGV----- 87
gi|Arabidopsis_thaliana_zeta        TSISKPSSTSSKSSSASGSRVANG-TLAPNSMSSNRNTNQGKKPLGGDAV 87
gi|Arabidopsis_thaliana_gamma       SSISKPSSK----SSASNSNGANGTVIAPSSTSSNR-TNQVN-------- 76
gi|Oryza                            RATSNLGSQPPIISSTGLSYGSGNRVENPNTRTNGNLYSSSF-------- 80
gi|Oryza_sativa_zeta                RS-SNLSSQTPVISSSGLSYGSGMHVGNANSRVNGNSVQPTV-------- 81
gi|Oryza_sativa_theta               SRIANPNNYTAAVTNPGQNYTVKNAHHGGAGVSNGFLAPPVF-------- 84
gi|Oryza_sativa_eta                 RSVEQGN------KRSGNGDYVVPAGLTPNPMMNGTVVYHSN-------- 73
gi|Arabidopsis_thaliana_delta       DNGESQT------LDNNNNQGGGDEVLSQRTSSNGDTSLDCVS------- 55
gi|Arabidopsis_thaliana_N/A         TSGGGSGPV------RSNSGKRMSSAVFPASVVAGIEP------------ 69
gi|Oryza_sativa_kappa               SCGSIPSGR------STSTIKRMSSAIFPSSVVAGIEP------------ 73
gi|Oryza_sativa_N/A                 GNGEPPAP--------------------PAAAAGGGG------------- 51
gi|Arabidopsis_thaliana_alpha       NMIVNHASRGSLVPSSPNSMAAATTQPPPMYS------------------ 68
gi|Arabidopsis_thaliana_beta        NVVVSHASRGALVNSSP-SPVTATPPPPPLGS------------------ 68
gi|Oryza_sativa_N/A_2               PVTVNHASRAAAPSSPPSPHAAAAASAPSAGAAASNQASSHHHQQ----- 86
gi|Arabidopsis_thaliana_epsilo      RTTTPAPSSVSNGESQTTAQSPSQTPNHPMFTTTP--------------- 74
gi|Chlamydomonas_reinhardtii_w      KAGAPAASS----KAPAPRPISAIREKPPLPAINEQTLQQYY-------- 66
gi|Schizosaccharomyces_pombe_P      PIDTTPTPRDEILLENVRT-------VRKQRSSLYHISENR--------- 102
gi|Schizosaccharomyces_pombe_P      VSQPTPLNKFLAGAENVDIPHSLRPVPRREHSSQFQVSEKR--------- 203
gi|Ashbya_gossypii_RTS1             IPRSSHSFERLPTPTKLNPDTDLELIKTPQRHSSSRFEPSR--------- 163
gi|Saccharomyces_cerevisiae_RT      IPRSSHSFERLPTPTKLNPDTDLELIKTPQRHSSSRFEPSR--------- 270
```

```
gi|Aspergillus_niger_N/A            ----------RELEKLQGFHEVPPN-RRQELFMQKIDQCNIIFDFNDP-- 192
gi|Aspergillus_nidulans_parA        ----------RELEKLPGFHEVPPN-RRQELFMQKIDQCNIIFDFNDP-- 194
gi|Mus_musculus_alpha               ----------AELHPLPQLKDATSN-EQQELFCQKLQQCCVLFDFMDS-- 87
gi|Rattus_norvegicus_alpha          ----------AELHPLPQLKDATSN-EQQELFCQKLQQCCVLFDFMDS-- 87
gi|Macaca_mulatta_alpha             ----------AELHPLPQLKDATSN-EQQDLFCQKLQQCCILFDFMDS-- 87
gi|Homo_sapiens_alpha               ----------AELHPLPQLKDATSN-EQQELFCQKLQQCCILFDFMDS-- 87
gi|Ovis_aries_alpha                 ----------PELHPLPQLKDATSN-EQQELFCQKLQQCCILFDFMDS-- 90
gi|Bos_taurus_alpha                 ----------PELHPLPQLKDATSN-EQQELFCQKLQQCCILFDFMDS-- 90
gi|Felis_catus_alpha                ---------------------------------------------MDS-- 3
gi|Canis_lupus_familiaris_alph      ---------------------------------------------MDS-- 3
gi|Falco_peregrinus_alpha           ----------VELSPLPQLKDATFN-EQQDLFCQKLQQCCVLFDFMDS-- 77
gi|Gallus_gallus_alpha              ----------VELSPLPQLKDATSN-EQQDLFCQKLQQCCVLFDFMDS-- 77
gi|Alligator_mississippiensis_      ----------VELSPLPQLKDATSN-EQQELFCQKLQQCCILFDFIDS-- 87
gi|Chrysemys_picta_bellii_alph      ----------VELSPLPQLKDATSN-EQQDLFCQKLQQCCILFDFMDS-- 77
gi|Xenopus_laevis_alpha             ----------VELSPLPALKDATSN-EQQDLFCQKLEQCCVLFDFMDS-- 76
gi|Xenopus_tropicalis_alpha         ----------VELSPLPPLKDATSN-DQQDLFCQKLQQCCVLFDFMDS-- 76
gi|Ambystoma_mexicanum_alpha        ---------------------------------------------MDS-- 3
gi|Danio_rerio_alpha                --------AAPEISALPQLKDASST-EQHELFMQKLQQCCKLFDFYDT-- 79
gi|Petromyzon_marinus_S4RHV1        ------------ELAPLPALADTPAS-EQEELFCKKLQQCCVIFDFLDS-- 77
gi|Mus_musculus_epsilon             ----------IELTPLPLLKDVPTS-EQPELFLKKLQQCCVIFDFMDT-- 79
gi|Rattus_norvegicus_epsilon        ----------IELTPLPLLKDVPTS-EQPELFLKKLQQCCVIFDFMDT-- 79
gi|Macaca_mulatta_epsilon           ----------IELTPLPLLKDVPSS-EQPELFLKKLQQCCVIFDFMDT-- 79
gi|Homo_sapiens_epsilon             ----------IELTPLPLLKDVPSS-EQPELFLKKLQQCCVIFDFMDT-- 79
gi|Ovis_arie_epsilon                ----------IELTPLPLLKDVPSS-EQPELFLKKLQQCCVIFDFMDT-- 79
gi|Bos_taurus_epsilon               ----------IELTPLPLLKDVPSS-EQPELFLKKLQQCCVIFDFMDT-- 79
gi|Canis_lupus_familiaris_epsi      ----------IELTPLPLLKDVPSS-EQPELFLKKLQQCCVIFDFMDT-- 79
gi|Falco_peregrinus_epsilon         ----------IELTPLPLLKDVPSS-EQPELFLKKLQQCCVIFDFMDT-- 79
gi|Gallus_gallus_epsilon            ----------IELTPLPLLKDVPSS-EQPELFLKKLQQCCVIFDFMDT-- 79
gi|Felis_catus_epsilon              ----------IELTPLPLLKDVPSS-EQPELFLKKLQQCCVIFDFMDT-- 79
gi|Chrysemys_picta_bellii_epsi      ----------IELTPLPLLKDVPSS-EQPELFLKKLQQCCVIFDFMDT-- 79
```

Figure S1. *Cont.*

```
gi|Xenopus_laevis_epsilon              ----------IELTPLPLLKDVPTL-EQPELFLKKLQQCGVIFDFMDT-- 79
gi|Xenopus_tropicalis_epsilon          ----------IELTPLPLLKDVPTS-EQPELFLKKLQQCCVIFDFMDT-- 79
gi|Alligator_mississippiensis_         ----------IELTPLPLLK------------------ACKALTFTES-- 87
gi|Ambystoma_mexicanum_epsilon         ----------IELTPLPLLKDVQNS-EQSELFLKKLQQCCVLFDFMET-- 75
gi|Danio_rerio_epsilon                 ----------IELVALPLLKDVSAQ-EQPELFLKKLQQCCTLFDFMDT-- 79
gi|Mus_musculus_beta                   ----------QELTPLPLLKDVPAS-ELHELLSRKLAQCGVMFDFLDC-- 93
gi|Rattus_norvegicus_beta              ----------QELTPLPLLKDVPAS-ELHELLSRKLAQCGVMFDFLDC-- 93
gi|Felis_catus_beta                    ----------QELTPLPLLKDVPAS-ELHELLSRKLAQCGVMFDFLDC-- 93
gi|Canis_lupus_familiaris_beta         ----------QELTPLPLLKDVPAS-ELHELLSRKLAQCGVMFDFLDC-- 93
gi|Homo_sapiens_beta                   ----------QELTPLPLLKDVPAS-ELHELLSRKLAQCGVMFDFLDC-- 93
gi|Bos_taurus_beta                     ----------QELTPLPLLKDVPAS-ELHELLSRKLAQCGVMFDFLDC-- 93
gi|Ovis_aries_beta                     ----------AQAQTPPPSPDVPAS-ELHELLSRKLAQCGVMFDFLDC-- 93
gi|Macaca_mulatta_beta                 ----------VQAQDALPSPDVPAS-ELHELLSRKLAQCGVMFDFLDC-- 69
gi|Xenopus_laevis_beta                 --------------------------------------------------
gi|Alligator_mississippiensis_         ----------QELTPLPLLKDVAVS-ELHELLGRKLQQCCVLFDFLDP-- 93
gi|Chrysemys_picta_bellii_beta         ----------QELTPLPLLKDVAVA-ELHELLCKKLQQCCVLFDFLDC-- 93
gi|Xenopus_tropicalis_beta             ----------QELTPLPPLKDAPVS-ELHDLFCKKLQQCSVIFQFMDC-- 91
gi|Danio_rerio_beta                    ----------VELTPLPLLKDAPVA-ELHDLFCKKLQQCCVLFDFLDC-- 92
gi|Ambystoma_mexicanum_beta            --------------------------------------------------
gi|Petromyzon_marinus_S4RGA7           --------------------------------------------------
gi|Strongylocentrotus_purpurat         --------NNTEIQQLN-LLKDTPPPEQQELVVKKLEQCCMVFDFMDA-- 75
gi|Drosophila_melanogaster_wdb         --------QDVELQQLPPLKADCSSLEQEELFIRKLRQCCVSFDFMDP-- 74
jgi|Branchiostoma_floridae_237         ------------------LPVDAPPAEQHELFIKKLQQCSVVFDFMDS-- 30
gi|Hydra_vulgaris_alpha                --------PVAELQPLT-LLKDAPAQERQELFIKKLQQCCTVFDFMDP-- 83
jgi|Branchiostoma_floridae_112         --------GGTELVPLPQLKDATPA-EQQELFLKKLQQCSVLFDFADDC- 78
jgi|Branchiostoma_floridae_284         ---------GSLLRPFPS-ADATPA-EQQELFLKKLQQCSVLFDFADDC- 44
gi|Caenorhabditis_elegans_PPTR         --------SAQELVQLALIKDTAAN-EQPALVIEKLVQCQHVFDFYDP-- 104
gi|Amphimedon_queenslandica_be         --------PPPDYKPLPLLKDSIQG-DRESLMLQKIAQCCYVFDFSDP-- 96
gi|Chrysemys_picta_bellii_gamm         ----------FQPVALLHIRDVPPA-EQEKLFIQKLRQCCVLFDFVSDP- 59
gi|Chrysemys_picta_bellii_delt         --------------------------------------------------
gi|Falco_peregrinus_gamma              ----------FQPVALLHIRDVPPA-DQEKLFIQKLRQCCVLFDFVSDP- 59
gi|Falco_peregrinus_delta/gamm         --------------------------------------------------
gi|Gallus_gallus_gamma                 ----------FQPVALLHIRDVPPA-DQEKLFIQKLRQCCVLFDFVSDP- 59
gi|Alligator_mississippiensis_         ----------FQPVALLHIRDVPPA-DQEKLFIQKLRQCCVLFDFVSDP- 59
gi|Alligator_mississippiensis_         --------------------------------------------------
gi|Ambystoma_mexicanum_gamma           ----------FQPVALSHIRDVPAA-EQEKLFIQKLRQCCVLFDFVSDP- 60
gi|Mus_musculus_gamma                  ----------FQPVALLHIRDVPPA-DQEKLFIQKLRQCCVLFDFVSDP- 59
gi|Rattus_norvegicus_gamma             ----------FQPVALLHIRDVPPA-DQEKLFIQKLRQCCVLFDFVSDP- 59
gi|Ovis_aries_gamma                    ----------FQPVALLHIRDVPPA-DQEKLFIQKLRQCCVLFDFVSDP- 59
gi|Bos_taurus_gamma                    ----------FQPVALLHIRDVPPA-DQEKLFIQKLRQCCVLFDFVSDP- 59
gi|Canis_lupus_familiaris_gamm         ----------FQPVALLHIRDVPPA-DQEKLFIQKLRQCCVLFDFVSDP- 59
gi|Macaca_mulatta_gamma                ----------FQPVALLHIRDVPPA-DQEKLFIQKLRQCCVLFDFVSDP- 59
gi|Homo_sapiens_gamma                  ----------FQPVVLLHIRDVPPA-DQEKLFIQKLRQCCVLFDFVSDP- 49
gi|Felis_catus_gamma                   ----------RELQKLPSLKDVPPA-DQEKLFIQKLRQCCVLFDFVSDP- 106
gi|Canis_lupus_familiaris_delt         ----------RELQKLPSLKDVPPA-DQEKLFIQKLRQCCVLFDFVSDP- 106
gi|Macaca_mulatta_delta/gamma          ----------RELQKLPSLKDVPPA-DQEKLFIQKLRQCCVLFDFVSDP- 341
gi|Bos_taurus_delta/gamma              ----------RELQKLPSLKDVPPA-DQEKLFIQKLRQCCVLFDFVSDP- 113
gi|Mus_musculus_delta/gamma            ----------RELQKLPSLKDVPPA-DQEKLFIQKLRQCCVLFDFVSDP- 116
gi|Rattus_norvegicus_delta/gam         ----------RELQKLPSLKDVPPA-DQEKLFIQKLRQCCVLFDFVSDP- 182
gi|Danio_rerio_gamma                   ---------------LMHFRDVPPA-EQEKLFVQKLRQCCVLFDFVSDP- 59
gi|Danio_rerio_delta/gamma             ----------RELQKLPVFTDVPPA-EQEKLFVQKLRQCCVLFDFVSDP- 112
gi|Xenopus_laevis_delta/gamma          ----------RELQKLPALKDVPPA-EQEKLFVQKLRQCCVLFDFVSDP- 112
gi|Xenopus_laevis_gamma                ----------RELQKLPALKDVPPA-EQEKLFVQKLRQCCVLFDFVSDP- 112
gi|Xenopus_tropicalis_delta/ga         ----------RELQKLPALKDVPPA-EQEKLFVQKLRQCCVLFDFVSDP- 112
gi|Mus_musculus_delta                  ----------RELQKLPALKDSPTQ-EREELFIQKLRQCCVLFDFVSDP- 127
gi|Rattus_norvegicus_delta             ----------RELQKLPALKDSPTQ-EREELFIQKLRQCCVLFDFVSDP- 155
gi|Felis_catus_delta                   ----------RELQKLPALKDSPTQ-EREELFIQKLRQCCVLFDFVSDP- 141
gi|Bos_taurus_delta                    ----------RELQKLPALKDSPTQ-EREELFIQKLRQCCVLFDFVSDP- 133
gi|Canis_lupus_familiaris_delt         ----------RELQKLPALKDSPTQ-EREELFIQKLRQCCVLFDFVSDP- 128
gi|Macaca_mulatta_delta                ----------RELQKLPALKDSPTQ-EREELFIQKLRQCCVLFDFVSDP- 135
gi|Homo_sapiens_delta                  ----------RELQKLPALKDSPTQ-EREELFIQKLRQCCVLFDFVSDP- 135
```

Figure S1. *Cont.*

```
gi|Ovis_aries_delta                  ----------RELQKLPALKDSPTQ-EREELFIQKLRQCCVLFDFVSDP- 131
gi|Falco_peregrinus_delta            -------------MSLLSLTDAPPH-EREELFIQKLRQCCVLFDFISDP- 35
gi|Gallus_gallus_delta               ----------RELQKLPALKDAPPH-EREELFIQKLRQCCVLFDFISDP- 130
gi|Alligator_mississippiensis_       ----------RELQKLPALKDAPPH-EREELFIQKLRQCCVLFDFISDP- 129
gi|Chrysemys_picta_bellii_delt       ----------RELQKLPALKDAPAH-EREELFIQKLRQCCVLFDFISDP- 131
gi|Danio_rerio_delta                 ----------RELQKLPALKDAPPI-DREELFVQKLRQCCVLFDFVTDP- 122
gi|Ambystoma_mexicanum_delta         --------------------------------------------------
gi|Petromyzon_marinus_S4RN43         ----------RELQKLPALKDAPPG-EREELFVQKLRQCCVLFDFVSDP- 112
jgi|Branchiostoma_floridae_237       ----------------PAHVDARPE-EREDLFVQKLRQCCVVFDFVNDP- 32
jgi|Branchiostoma_floridae_252       ----------RELQKLAALKDARPE-EREDLFVQKLRQCCVVFDFVNDP- 85
gi|Strongylocentrotus_purpurat       ----------RELQKLPPLKDATPG-EREDLFSKKLLQCCVVFDFISDP- 93
gi|Caenorhabditis_elegans_PPTR       ----------RELQRLPAIKDADPS-ERETLFIQKLRQCCVVFDFANDA- 79
gi|Hydra_vulgaris_delta              ----------RELQKLPLIKDTNNS-EKESLFIQKLQQCCVLFDFVVDP- 102
gi|Amphimedon_queenslandica_de       ----------REISKLPPLKDATPN-AREDLFLQKLHQCSIIFDFNRDP- 143
gi|Drosophila_melanogaster_B56       ----------CELTALIPLNEKTAASEREELFIQKIQQCCTLFDFS-EP- 216
gi|Dictyostelium_discoideum_ps       ------------LTKIANFQDVSPE-ERPSLFLLKLKQCCYVYDFSDN-- 142
gi|Dictyostelium_purpureum_N/A       ------------LTKIPGFQEVPPE-ERPSLFLLKLKQCCYVYDFSEN-- 99
gi|Dictyostelium_fasciculatum_       ------------LQQIPGFHDVPPE-ERQSLLIKKLKQCCYVFDFSDTE- 112
gi|Polysphondylium_pallidum_N/       ------------LQQIPAFNVVPPE-ERPNLLLLKLKQCCALFDFSDSE- 90
gi|Arabidopsis_thaliana_theta        ---------FTPYEALPGFKDVPNA-EKQNLFVRKLSLCCVVFDFSD--- 103
gi|Arabidopsis_thaliana_eta          ---------FTPYEALPSFKDVPNT-EKQNLFIKKLNLCRVVFDFTD--- 124
gi|Arabidopsis_thaliana_zeta         VQAGPFPSSGGVYEALPSFRDVPIS-EKPNLFIGKLSMCCVVFDFSD--- 133
gi|Arabidopsis_thaliana_gamma        ----------GVYEALPSFRDVPTS-EKPNLFIKKLSMCCVVFDFND--- 112
gi|Oryza                             -------------QPLPSFKDVPNS-EKQNLLIRKLKLCCIVFDFTD--- 113
gi|Oryza_sativa_zeta                 -------------ELLPSFKDVPNT-EKNNLFVKKLNLCCATFDFTD--- 114
gi|Oryza_sativa_theta                -------------EALPSLRDAPAP-EKPSLFLRKVVMCYVVFDFTD--- 117
gi|Oryza_sativa_eta                  -------------EPLPAFKDVPAS-EKQNLFVKKVNLCCAVYDFAD--- 106
gi|Arabidopsis_thaliana_delta        -----------SFDVLPRLRDVSIS-EKQELFLKKLRLCCLVFDFVAE-- 91
gi|Arabidopsis_thaliana_N/A          ---------------LVPFKDVPSS-EKLNLFVSKVSLCCVTFDFS---- 99
gi|Oryza_sativa_kappa                ---------------LVSFKDVPNS-EKQNLFVSKLNLCCAVFDFS---- 103
gi|Oryza_sativa_N/A                  --------------------GAEGE-TREDVFLRKLNVCCVVFDFSSAAA 80
gi|Arabidopsis_thaliana_alpha        ------------VEPLPLFRDVSVS-ERQSLFLRKLQICCFQFDFTD--- 102
gi|Arabidopsis_thaliana_beta         ------------VEPLPLFRDVPVS-ERQTLFLRKLQNCCFLFDFTD--- 102
gi|Oryza_sativa_N/A_2                -QQPAAAPQPPLLEPLPLLRDVAAA-DRPGLLVRKLRLVAALFDFTD--- 131
gi|Arabidopsis_thaliana_epsilo       -----------ILEVLPLLKDVSSS-DRPLLFMKKAHMCSCHCDFSD--- 109
gi|Chlamydomonas_reinhardtii_w       ------------AEPLPSFRDVSPA-EKQYLFVQKLHLCSFGFDFAD--- 100
gi|Schizosaccharomyces_pombe_P       -----------NLVRLPSFTDVPVN-KWHSLALEKLEQCCVVFDFNDPS- 139
gi|Schizosaccharomyces_pombe_P       -----------TLVRLPSFDDVHTS-EREELFIKKLEQCNIIFDFNDPS- 240
gi|Ashbya_gossypii_RTS1              ---------YTQISKLPGFDDVPPE-EQISLFIAKVDQCNIMFDFSDPS- 202
gi|Saccharomyces_cerevisiae_RT       ---------YTPLTKLPNFNEVSPE-ERIPLFIAKVDQCNTMFDFNDPS- 309

gi|Aspergillus_niger_N/A             ------TADMKSKEIKRLALHELLDYIANNRS--------VITEPMY-PR 227
gi|Aspergillus_nidulans_parA         ------TADMKSKEIKRLALHELLDYIANNRS--------VITEPMY-PR 229
gi|Mus_musculus_alpha                ------VSDLKSKEIKRATLNELVEYVS-TNR-------GVIVESAY-SD 122
gi|Rattus_norvegicus_alpha           ------VSDLKSKEIKRATLNELVEYVS-TNR-------GVIVESAY-SD 122
gi|Macaca_mulatta_alpha              ------VSDLKSKEIKRATLNELVEYVS-TNR-------GVIVESAY-SD 122
gi|Homo_sapiens_alpha                ------VSDLKSKEIKRATLNELVEYVS-TNR-------GVIVESAY-SD 122
gi|Ovis_aries_alpha                  ------VSDLKSKEIKRATLNELVEYVS-TNR-------GVIVESAY-SD 125
gi|Bos_taurus_alpha                  ------VSDLKSKEIKRATLNELVEYVS-TNR-------GVIVESAY-SD 125
gi|Felis_catus_alpha                 ------VSDLKSKEIKRATLNELVEYVS-TNR-------GVIVESAY-SD 38
gi|Canis_lupus_familiaris_alph       ------VSDLKSKEIKRATLNELVEYVS-TNR-------GVIVESAY-SD 38
gi|Falco_peregrinus_alpha            ------VSDLKSKEIKRATLNELVEYVS-TNR-------GVIVESAY-AD 112
gi|Gallus_gallus_alpha               ------VSDLKGKEIKRATLNELVEYVS-TNR-------GVIVESAY-AD 112
gi|Alligator_mississippiensis_       ------VSDLKSKEIKRATLNELVEYVS-TNR-------GVLVESAY-AD 122
gi|Chrysemys_picta_bellii_alph       ------VSDLKSKEIKRATLNELVEYVS-TTR-------GVIVESAY-AD 112
gi|Xenopus_laevis_alpha              ------ISDLKSKEIKRATLNELVEYVA-TNR-------GVLVETAY-PE 111
gi|Xenopus_tropicalis_alpha          ------ISDLKSKEIKRATLNELVEYVS-INR-------GVLVESAY-PD 111
gi|Ambystoma_mexicanum_alpha         ------VSDLKSKEIKRATLNELVEYVS-TNR-------GVIVETAY-PD 38
gi|Danio_rerio_alpha                 ------VTDLKSKETKRATLSELVDYVS-TNR-------GVLVEPVY-PE 114
gi|Petromyzon_marinus_S4RHV1         ------VSDLKGKEIKRAALNELVDYIT-AGR-------GVLTESVY-PD 112
```

Figure S1. *Cont.*

```
gi|Mus_musculus_epsilon              ------LSDLKMKEYKRSTLNELVDYIT-ISR-------GCLTEQTY-PE 114
gi|Rattus_norvegicus_epsilon         ------LSDLKMKEYKRSTLNELVDYIT-ISR-------GCLTEQTY-PE 114
gi|Macaca_mulatta_epsilon            ------LSDLKMKEYKRSTLNELVDYIT-ISR-------GCLTEQTY-PE 114
gi|Homo_sapiens_epsilon              ------LSDLKMKEYKRSTLNELVDYIT-ISR-------GCLTEQTY-PE 114
gi|Ovis_arie_epsilon                 ------LSDLKMKEYKRSTLNELVDYIT-ISR-------GCLTEQTY-PE 114
gi|Bos_taurus_epsilon                ------LSDLKMKEYKRSTLNELVDYIT-ISR-------GCLTEQTY-PE 114
gi|Canis_lupus_familiaris_epsi       ------LSDLKMKEYKRSTLNELVDYIT-ISR-------GCLTEQTY-PE 114
gi|Falco_peregrinus_epsilon          ------LSDLKMKEYKRSTLNELVDYIT-ISR-------GCLTEQTY-PE 114
gi|Gallus_gallus_epsilon             ------LSDLKMKEYKRSTLNELVDYIT-ISR-------GCLTEQTY-PE 114
gi|Felis_catus_epsilon               ------LSDLKMKEYKRSTLNELVDYIT-ISR-------GCLTEQTY-PE 114
gi|Chrysemys_picta_bellii_epsi       ------LSDLKMKEYKRSTLNELVDYIT-ISR-------GCLTEQTY-PE 114
gi|Xenopus_laevis_epsilon            ------LSDLKMKEYKRSTLNELVDYVA-ISR-------GCLTEQTY-PE 114
gi|Xenopus_tropicalis_epsilon        ------LSDLKMKEYKRSTLNELVDYIT-ISR-------GCLTEQTY-PE 114
gi|Alligator_mississippiensis_       ------LFS------KNTLLISLKTFLN-YKN-------G---------- 107
gi|Ambystoma_mexicanum_epsilon       ------LSDLKMKEYKRSTLNELVDYIT-ISR-------GCLTEQTY-PE 110
gi|Danio_rerio_epsilon               ------LSDLKMKEYKRSTLNELVDYVT-LSR-------GYLTEQTY-PE 114
gi|Mus_musculus_beta                 ------VADLKGKEVKRAALNELVECVG-CTR-------GVLIEPVY-PD 128
gi|Rattus_norvegicus_beta            ------VADLKGKEVKRAALNELVECVG-STR-------GVLIEPVY-PD 128
gi|Felis_catus_beta                  ------VADLKGKEVKRAALNELVECVG-STR-------GVLIEPVY-PD 128
gi|Canis_lupus_familiaris_beta       ------VADLKGKEVKRAALNELVECVG-STR-------GVLIEPVY-PD 128
gi|Homo_sapiens_beta                 ------VADLKGKEVKRAALNELVECVG-STR-------GVLIEPVY-PD 128
gi|Bos_taurus_beta                   ------VADLKGKEVKRAALNELVECVG-STR-------GVLIEPVY-PD 128
gi|Ovis_aries_beta                   ------VADLKGKEVKRAALNELVECVG-STR-------GVLIEPVY-PD 128
gi|Macaca_mulatta_beta               ------VADLKGKEVKRAALNELVECVG-STR-------GVLIEPVY-PD 104
gi|Xenopus_laevis_beta               --------------------------------------------------
gi|Alligator_mississippiensis_       ------VADLKGKEVKRAALAELVECVA-TAR-------GLLIEPTY-PE 128
gi|Chrysemys_picta_bellii_beta       ------VADLKGKEIKRAALNELVECVA-TNR-------GVLIEPVY-PE 128
gi|Xenopus_tropicalis_beta           ------VADLKGKEIKRAALNELVECIA-TNR-------GVLIEPVY-PE 126
gi|Danio_rerio_beta                  ------VADLKGKEIKRAALNELVESVA-TSR-------GVLIEPLY-PE 127
gi|Ambystoma_mexicanum_beta          --------------------------------------------------
gi|Petromyzon_marinus_S4RGA7         --------------------------------------------------
gi|Strongylocentrotus_purpurat       ------VSELRGKEIKRASLNELVDYIT-TGR-------GVLTEPLY-PE 110
gi|Drosophila_melanogaster_wdb       ------VTDLKGKEIKRAALNDLSTYIT-HGR-------GVLTEPVY-PE 109
jgi|Branchiostoma_floridae_237       ------VSDLKGKEIKRASLNELVDYIT-AGR-------GVLTEQVY-PE 65
gi|Hydra_vulgaris_alpha              ------VGDLRGKEVKRGTLNELVDYIT-SER-------GVLTEPVY-PE 118
jgi|Branchiostoma_floridae_112       ------VSELRSMEVKRFSLIEVLDHIG-KT--------GVLLEQTY-PE 112
jgi|Branchiostoma_floridae_284       ------VSELRSMEVKRFSLIEVLDHIG-KT--------GVLLEQTY-PE 78
gi|Caenorhabditis_elegans_PPTR       ------VAQLKCKEIKRAALNELIDHIT-STK-------GAIVETIY-PA 139
gi|Amphimedon_queenslandica_be       ------MADLRAKEIKRAALNEILDYIT-NYK-------EVLSEPLY-PE 131
gi|Chrysemys_picta_bellii_gamm       ------LSDLKWKEVKRAALSEMVEYIT-HNR-------NVITEPIY-PE 94
gi|Chrysemys_picta_bellii_delt       ----------------------MVEYIT-HNR-------NVITEPIY-PE 19
gi|Falco_peregrinus_gamma            ------LSDLKWKEVKRAALSEMVEYIT-HNR-------NVITEPIY-PE 94
gi|Falco_peregrinus_delta/gamm       ----------------------MVEYIT-HNR-------NVITEPIY-PE 19
gi|Gallus_gallus_gamma               ------LSDLKWKEVKRAALSEMVEYIT-HNR-------NVITEPIY-PE 94
gi|Alligator_mississippiensis_       ------LSDLKWKEVKRAALSEMVEYIT-HNR-------NVITEPIY-PE 94
gi|Alligator_mississippiensis_       ----------------------MVEYIT-HNR-------NVITEPIY-PE 19
gi|Ambystoma_mexicanum_gamma         ------LSDLKWKEVKRAALSEMVEYIT-HNR-------NVITEPIY-PE 95
gi|Mus_musculus_gamma                ------LSDLKWKEVKRAALSEMVEYIT-HNR-------NVITEPIY-PE 94
gi|Rattus_norvegicus_gamma           ------LSDLKWKEVKRAALSEMVEYIT-HNR-------NVITEPIY-PE 94
gi|Ovis_aries_gamma                  ------LSDLKWKEVKRAALSEMVEYIT-HNR-------NVITEPIY-PE 94
gi|Bos_taurus_gamma                  ------LSDLKWKEVKRAALSEMVEYIT-HNR-------NVITEPIY-PE 94
gi|Canis_lupus_familiaris_gamm       ------LSDLKWKEVKRAALSEMVEYIT-HNR-------NVITEPIY-PE 94
gi|Macaca_mulatta_gamma              ------LSDLKWKEVKRAALSEMVEYIT-HNR-------NVITEPIY-PE 94
gi|Homo_sapiens_gamma                ------LSDLKWKEVKRAALSEMVEYIT-HNR-------NVITEPIY-PE 84
gi|Felis_catus_gamma                 ------LSDLKWKEVKRAALSEMVEYIT-HNR-------NVITEPIY-PE 141
gi|Canis_lupus_familiaris_delt       ------LSDLKWKEVKRAALSEMVEYIT-HNR-------NVITEPIY-PE 141
gi|Macaca_mulatta_delta/gamma        ------LSDLKWKEVKRAALSEMVEYIT-HNR-------NVITEPIY-PE 376
gi|Bos_taurus_delta/gamma            ------LSDLKWKEVKRAALSEMVEYIT-HNR-------NVITEPIY-PE 148
gi|Mus_musculus_delta/gamma          ------LSDLKWKEVKRAALSEMVEYIT-HNR-------NVITEPIY-PE 151
gi|Rattus_norvegicus_delta/gam       ------LSDLKWKEVKRAALSEMVEYIT-HNR-------NVITEPIY-PE 217
gi|Danio_rerio_gamma                 ------LSDLKWKEVKRAALSEMVEYIT-HNR-------NVITEPIY-PE 94
```

Figure S1. *Cont.*

```
gi|Danio_rerio_delta/gamma          ------LSDLKWKEVKRAALSEMVEYIT-HNR-------NVITEPIY-PE 147
gi|Xenopus_laevis_delta/gamma       ------LSDLKWKEVKRAALSEMVEYIT-HNR-------NVITEPIY-PE 147
gi|Xenopus_laevis_gamma             ------LSDLKWKEVKRAALSEMVEYIT-HNR-------NVITEPIY-PE 147
gi|Xenopus_tropicalis_delta/ga      ------LSDLKWKEVKRAALSEMVEYIT-HNR-------NVITEPIY-PE 147
gi|Mus_musculus_delta               ------LSDLKCKEVKRAGLNEMVEYIT-HSR-------DVVTEAIY-PE 162
gi|Rattus_norvegicus_delta          ------LSDLKFKEVKRAGLNEMVEYIT-HSR-------DVVTEAIY-PE 190
gi|Felis_catus_delta                ------LSDLKFKEVKRAGLNEMVEYIT-HSR-------DVVTEAIY-PE 176
gi|Bos_taurus_delta                 ------LSDLKFKEVKRAGLNEMVEYIT-HSR-------DVVTEAIY-PE 168
gi|Canis_lupus_familiaris_delt      ------LSDLKFKEVKRAGLNEMVEYIT-HSR-------DVVTEAIY-PE 163
gi|Macaca_mulatta_delta             ------LSDLKFKEVKRAGLNEMVEYIT-HSR-------DVVTEAIY-PE 170
gi|Homo_sapiens_delta               ------LSDLKFKEVKRAGLNEMVEYIT-HSR-------DVVTEAIY-PE 170
gi|Ovis_aries_delta                 ------LSDLKFKEVKRAGLNEMVEYIT-HSR-------DVVTEAIYGPG 167
gi|Falco_peregrinus_delta           ------LSDLKFKEVKRAGLNEMVEYIT-HNR-------DVVTEAIY-PE 70
gi|Gallus_gallus_delta              ------LSDLKFKEVKRAGLNEMVEYIT-HNR-------DVVTEAIY-PE 165
gi|Alligator_mississippiensis_      ------LSDLKFKEVKRAGLNEMVEYIT-HNR-------DVITEAIY-PE 164
gi|Chrysemys_picta_bellii_delt      ------LSDLKFKEVKRAGLNEMVEYIT-HNR-------DVVTEAIY-PE 166
gi|Danio_rerio_delta                ------LSDLKYKEVKRAGLNEMVEYIT-HNR-------DVVTESIY-PE 157
gi|Ambystoma_mexicanum_delta        ---------------------MVEYIT-HNR-------DVVTESIY-PE 19
gi|Petromyzon_marinus_S4RN43        ------LSDLKWKEVKRAALNEMVEYIT-HSR-------GVITEPIY-PE 147
jgi|Branchiostoma_floridae_237      ------LSDLKWKEVKRAALNEMVEYVT-HNR-------GVITEAVY-PE 67
jgi|Branchiostoma_floridae_252      ------LSDLKWKEVKRAALNEMVEYVT-HNR-------GVITEAVY-PE 120
gi|Strongylocentrotus_purpurat      ------LSDLKWKEVKRAALNELVEYVT-HQR-------GIITEAIY-PE 128
gi|Caenorhabditis_elegans_PPTR      ------LSDLKFKEVKRAALNELVDHVSGAPK-------GSLSDAVY-PE 115
gi|Hydra_vulgaris_delta             ------LSDLKWKEVKRAALNEIVDYLT-HNK-------NVITEPVY-PE 137
gi|Amphimedon_queenslandica_de      ------LSDLKFKEVKRAALNELVEFIT-HNR-------GVITESIY-PE 178
gi|Drosophila_melanogaster_B56      ------LSDLKFKEVKRAALHEMVDFLT-NQN-------GVITEVIY-PE 251
gi|Dictyostelium_discoideum_ps      -------TYMVSKGVKQEALLQCVNFLSTN--------DQPLHESIY-KM 176
gi|Dictyostelium_purpureum_N/A      -------NYMVSKSIKQEALLQCVQFLSTN--------DQPLHESVY-KM 133
gi|Dictyostelium_fasciculatum_      -------SDKSSKAIKLEALLQCVEFLSTN--------KEVISEPIY-EA 146
gi|Polysphondylium_pallidum_N/      -------ADSKSKSIKREALLQCVDYLSSS--------KDAYTEAVY-EA 124
gi|Arabidopsis_thaliana_theta       -----PTKNVKEKDIKRQTLLELVDYVASP--------NGKFSETVI-QE 139
gi|Arabidopsis_thaliana_eta         -----PTKNIKEKDIKRQTLLELVDYVNSP--------NGKFSEVGI-QE 160
gi|Arabidopsis_thaliana_zeta        -----PSKNLKEKEIKRQTLLELVDYVASV--------GFKFNDVSM-QE 169
gi|Arabidopsis_thaliana_gamma       -----PSKNLREKEIKRQTLLELVDYIATV--------STKLSDAAM-QE 148
gi|Oryza                            -----PTKNIQEKEMKSQTLLEIVDYVVSA--------TVKFPEIVM-LE 149
gi|Oryza_sativa_zeta                -----PTKSVKEKEVKRQTLLELVDYIASA--------NGKFPEIIM-QE 150
gi|Oryza_sativa_theta               -----PTKDVKEKEIKRQTLLELVDYVTSA--------TGKFPEPAV-QE 153
gi|Oryza_sativa_eta                 -----PTKNLKEKETKRQTLMELVDYVTSA--------NGKFSEVVM-SE 142
gi|Arabidopsis_thaliana_delta       -----PQQNFKEKEIKRQTLLEVVDYVISSG-------NGKFPESVI-QE 128
gi|Arabidopsis_thaliana_N/A         ----DPGKNSIEKDVKRQTLLELLDFVASG--------SVKFTEPAI-LA 136
gi|Oryza_sativa_kappa               ----DPNKSSAEKDIKRQTLLDLIDYVDSS--------SSRFSEAVI-AA 140
gi|Oryza_sativa_N/A                 AAAAERGRDSPERERKRQVLVSLVDCVGAA--------EEPLTEAMI-SG 121
gi|Arabidopsis_thaliana_alpha       -----TLKNAREKEIKRQTLLELVDFIQSGA-------GKLTEVCQ-EE 138
gi|Arabidopsis_thaliana_beta        -----TIKNARDKEIKRQTLLELVDFIQSGS--------SKISESCQ-EE 138
gi|Oryza_sativa_N/A_2               -----SLKHPREKEAKRQALLELVDYVQAPSPAANANAPARLPENVQ-EA 175
gi|Arabidopsis_thaliana_epsilo      -----TLIMPREKEIKRQTLLELVDFLHSSS--------GKVNETMQ-SE 145
gi|Chlamydomonas_reinhardtii_w      -----PTKHVREKEIKRQTLLELVDYANSG--------AGKFTEGVS-ED 136
gi|Schizosaccharomyces_pombe_P      -------TDLYGKEVKREALQDLIDLIS-VRK-------EAIDESLY-PS 173
gi|Schizosaccharomyces_pombe_P      -------SDLASKEIKREALLQMIDYVS-ENR-------GISSASLF-PY 274
gi|Ashbya_gossypii_RTS1             -------FDIHGKEIKRITLQELIEFIVTNRF--------TYTEEMY-GH 236
gi|Saccharomyces_cerevisiae_RT      -------FDIQGKEIKRSTLDELIEFLVTNRF--------TYTNEMY-AH 343


gi|Aspergillus_niger_N/A            VVEMFAKNLFRPIPPPVTPQ-----GEAFDPEEDEPVLEVAWPHIQV--- 269
gi|Aspergillus_nidulans_parA        VVEMFAKNLFRPIPPPITPQ-----GEAFDPEEDEPVLEVAWPHIQV--- 271
gi|Mus_musculus_alpha               IVKMISANIFRTLPPSDN--------PDFDPEEDEPTLEASWPHIQL--- 161
gi|Rattus_norvegicus_alpha          IVKMISANIFRTLPPSDN--------PDFDPEEDEPTLEASWPHIQL--- 161
gi|Macaca_mulatta_alpha             IVKMISANIFRTLPPSDN--------PDFDPEEDEPTLEASWPHIQL--- 161
gi|Homo_sapiens_alpha               IVKMISANIFRTLPPSDN--------PDFDPEEDEPTLEASWPHIQL--- 161
gi|Ovis_aries_alpha                 IVKMISANIFRTLPPSDN--------PDFDPEEDEPTLEASWPHIQL--- 164
gi|Bos_taurus_alpha                 IVKMISANIFRTLPPSDN--------PDFDPEEDEPTLEASWPHIQL--- 164
```

Figure S1. *Cont.*

```
gi|Felis_catus_alpha                IVKMISANIFRTLPPSDN--------PDFDPEEDEPTLEASWPHIQL--- 77
gi|Canis_lupus_familiaris_alph      IVKMISANIFRTLPPSDN--------PDFDPEEDEPTLEASWPHIQL--- 77
gi|Falco_peregrinus_alpha           IVKMISSNIFRTLPPSDN--------PDFDPEEDEPTLEASWPHIQL--- 151
gi|Gallus_gallus_alpha              IVKMISSNIFRTLPPSDN--------PDFDPEEDEPTLEASWPHIQL--- 151
gi|Alligator_mississippiensis_      IVKMISSNIFRTLPPSDN--------PDFDPEEDEPTLEASWPHIQL--- 161
gi|Chrysemys_picta_bellii_alph      IVKMISSNIFRTLPPSDN--------PDFDPEEDEPTLEASWPHIQL--- 151
gi|Xenopus_laevis_alpha             IIKMICSNIFRTLPPSDN--------PDFDPEEDEPTLEASWPHIQL--- 150
gi|Xenopus_tropicalis_alpha         IIKMICSNIFRTLPPSDN--------PDFDPEEDEPTLEASWPHIQL--- 150
gi|Ambystoma_mexicanum_alpha        IVRMISSNIFRTLPPSDN--------PEFDPEEDEPTLEASWPHIQL--- 77
gi|Danio_rerio_alpha                ITTMVSTNIFRTLPPSEN--------PDFDPEEDEPTLEASWPHMQL--- 153
gi|Petromyzon_marinus_S4RHV1        IIRMVAYNIFRTLPPSEN--------PEFDPEEDEPTLEASWPHLQI--- 151
gi|Mus_musculus_epsilon             VVRMVSCNIFRTLPPSDS--------NEFDPEEDEPTLEASWPHLQL--- 153
gi|Rattus_norvegicus_epsilon        VVRMVSCNIFRTLPPSDS--------NEFDPEEDEPTLEASWPHLQL--- 153
gi|Macaca_mulatta_epsilon           VVRMVSCNIFRTLPPSDS--------NEFDPEEDEPTLEASWPHLQL--- 153
gi|Homo_sapiens_epsilon             VVRMVSCNIFRTLPPSDS--------NEFDPEEDEPTLEASWPHLQL--- 153
gi|Ovis_arie_epsilon                VVRMVSCNIFRTLPPSDS--------NEFDPEEDEPTLEASWPHLQL--- 153
gi|Bos_taurus_epsilon               VVRMVSCNIFRTLPPSDS--------NEFDPEEDEPTLEASWPHLQL--- 153
gi|Canis_lupus_familiaris_epsi      VVRMVSCNIFRTLPPSDS--------NEFDPEEDEPTLEASWPHLQL--- 153
gi|Falco_peregrinus_epsilon         VVRMVSCNIFRTLPPSDS--------NEFDPEEDEPTLEASWPHLQL--- 153
gi|Gallus_gallus_epsilon            VVRMVSCNIFRTLPPSDS--------NEFDPEEDEPTLEASWPHLQL--- 153
gi|Felis_catus_epsilon              VVRMVSCNIFRTLPPSDS--------NEFDPEEDEPTLEASWPHLQL--- 153
gi|Chrysemys_picta_bellii_epsi      VVRMVSCNIFRTLPPSDS--------NEFDPEEDEPTLEASWPHLQL--- 153
gi|Xenopus_laevis_epsilon           AVRMVSCNIFRTLPPSDS--------NEFDPEEDEPTLEASWPHLQL--- 153
gi|Xenopus_tropicalis_epsilon       VVRMVSCNIFRTLPPSDS--------NEFDPEEDEPTLEASWPHLQL--- 153
gi|Alligator_mississippiensis_      ----VSCNIFRTLPPSDS--------NEFDPEEDEPTLEASWPHLQL--- 142
gi|Ambystoma_mexicanum_epsilon      VVRMVSYNIFRTLPPSDS--------NEFDPEEDEPTLEASWPHLQL--- 149
gi|Danio_rerio_epsilon              VVKMVSYNIFRTLPPSDS--------NEFDPEEDEPTLEASWPHLQL--- 153
gi|Mus_musculus_beta                IIRMISVNIFRTLPPSEN--------PEFDPEEDEPNLEPSWPHLQL--- 167
gi|Rattus_norvegicus_beta           IIRMISVNIFRTLPPSEN--------PEFDPEEDEPNLEPSWPHLQL--- 167
gi|Felis_catus_beta                 IIRMISVNIFRTLPPSEN--------PEFDPEEDEPNLEPSWPHLQL--- 167
gi|Canis_lupus_familiaris_beta      IIRMISVNIFRTLPPSEN--------PEFDPEEDEPNLEPSWPHLQL--- 167
gi|Homo_sapiens_beta                IIRMISVNIFRTLPPSEN--------PEFDPEEDEPNLEPSWPHLQL--- 167
gi|Bos_taurus_beta                  IIRMISVNIFRTLPPSEN--------PEFDPEEDEPNLEPSWPHLQL--- 167
gi|Ovis_aries_beta                  IIRMISVNIFRTLPPSEN--------PEFDPEEDEPNLEPSWPHLQL--- 167
gi|Macaca_mulatta_beta              IIRMISVNIFRTLPPSEN--------PEFDPEEDEPNLEPSWPHCSL--- 143
gi|Xenopus_laevis_beta              --------------------------------------------------
gi|Alligator_mississippiensis_      AVRMISVNIFRTLPPSEN--------PEFDPEEDEPNLEPSWPHLQL--- 167
gi|Chrysemys_picta_bellii_beta      IIKMISVNIFRTLPPTEN--------PEFDPEEDEPNLEPSWPHLQL--- 167
gi|Xenopus_tropicalis_beta          VIKMISVNIFRTLPPTDN--------PEFDPEEDEPNFEPSWPHLQL--- 165
gi|Danio_rerio_beta                 AIKMISVNIFRTLPPSEN--------PEFDPEEDEPALEASWPHLQL--- 166
gi|Ambystoma_mexicanum_beta         ---MISGNIFRTLPPSEN--------PEFDPEEDEPNLEPSWPHLQL--- 36
gi|Petromyzon_marinus_S4RGA7        --------------------------------------------------
gi|Strongylocentrotus_purpurat      CIQMISCNIFRTLPPSEN--------SDFDPEEDDPTLEASWPHLQL--- 149
gi|Drosophila_melanogaster_wdb      IIRMISCNLFRTLPPSEN--------PDFDPEEDDPTLEASWPHLQL--- 148
jgi|Branchiostoma_floridae_237      VVKMIASNIFRTLPPSEN--------TEFDPEEDDPTLEASWPHLQI--- 104
gi|Hydra_vulgaris_alpha             IVRMVANNCFKTLPPSNN--------PDFDPEKNEPTLEASWPHLQV--- 157
jgi|Branchiostoma_floridae_112      VVKMIAANIFRPLPPAEN--------AEFDPDEDEPTFEASWPHLQ---- 150
jgi|Branchiostoma_floridae_284      VVKMVSTNIFRPLPPAEN--------AEFDPDEDEPTFEASWPHLQI--- 117
gi|Caenorhabditis_elegans_PPTR      VIKMVAKNIFRVLPPSEN--------CEFDPEEDEPTLEVSWPHLQL--- 178
gi|Amphimedon_queenslandica_be      IVRMMTTNAFRTLPPLEQG-------TEYDPDEDEPPTELTWPHLEL--- 171
gi|Chrysemys_picta_bellii_gamm      VVHMFAVNMFRTLPPSSNPT-----GAEFDPEEDEPTLEAAWPHLQL--- 136
gi|Chrysemys_picta_bellii_delt      VVHMFAVNMFRTLPPSSNPT-----GAEFDPEEDEPTLEAAWPHLQL--- 61
gi|Falco_peregrinus_gamma           VVHMFAVNMFRTLPPSSNPT-----GAEFDPEEDEPTLEAAWPHLQL--- 136
gi|Falco_peregrinus_delta/gamm      VVHMFAVNMFRTLPPSSNPT-----GAEFDPEEDEPTLEAAWPHLQL--- 61
gi|Gallus_gallus_gamma              VVHMFAVNMFRTLPPSSNPT-----GAEFDPEEDEPTLEAAWPHLQL--- 136
gi|Alligator_mississippiensis_      VVHMFAVNMFRTLPPSSNPT-----GAEFDPEEDEPTLEAAWPHLQL--- 136
gi|Alligator_mississippiensis_      VVHMFAVNMFRTLPPSSNPT-----GAEFDPEEDEPTLEAAWPHLQL--- 61
gi|Ambystoma_mexicanum_gamma        VVHMFAVNMFRTLPPSSNPT-----GAEFDPEEDEPTLEAAWPHLQL--- 137
gi|Mus_musculus_gamma               AVHMFAVNMFRTLPPSSNPT-----GAEFDPEEDEPTLEAAWPHLQL--- 136
gi|Rattus_norvegicus_gamma          AVHMFAVNMFRTLPPSSNPT-----GAEFDPEEDEPTLEAAWPHLQL--- 136
gi|Ovis_aries_gamma                 VVHMFAVNMFRTLPPSSNPT-----GAEFDPEEDEPTLEAAWPHLQL--- 136
```

Figure S1. *Cont.*

```
gi|Bos_taurus_gamma                VVHMFAVNMFRTLPPSSNPT-----GAEFDPEEDEPTLEAAWPHLQL--- 136
gi|Canis_lupus_familiaris_gamm     VVHMFAVNMFRTLPPSSNPT-----GAEFDPEEDEPTLEAAWPHLQL--- 136
gi|Macaca_mulatta_gamma            VVHMFAVNMFRTLPPSSNPT-----GAEFDPEEDETTLEAAWPHLQL--- 136
gi|Homo_sapiens_gamma              VVHMFAVNMFRTLPPSSNPT-----GAEFDPEEDEPTLEAAWPHLQL--- 126
gi|Felis_catus_gamma               VVHMFAVNMFRTLPPSSNPT-----GAEFDPEEDEPTLEAAWPHLQL--- 183
gi|Canis_lupus_familiaris_delt     VVHMFAVNMFRTLPPSSNPT-----GAEFDPEEDEPTLEAAWPHLQL--- 183
gi|Macaca_mulatta_delta/gamma      VVHMFAVNMFRTLPPSSNPT-----GAEFDPEEDETTLEAAWPHLQL--- 418
gi|Bos_taurus_delta/gamma          VVHMFAVNMFRTLPPSSNPT-----GAEFDPEEDEPTLEAAWPHLQL--- 190
gi|Mus_musculus_delta/gamma        AVHMFAVNMFRTLPPSSNPT-----GAEFDPEEDEPTLEAAWPHLQL--- 193
gi|Rattus_norvegicus_delta/gam     AVHMFAVNMFRTLPPSSNPT-----GAEFDPEEDEPTLEAAWPHLQL--- 259
gi|Danio_rerio_gamma               VVHMFAVNMFRTLPPSSNPT-----GAEFDPEEDEPTLEAAWPHLQL--- 136
gi|Danio_rerio_delta/gamma         VVHMFAVNMFRTLPPSSNPT-----GAEFDPEEDEPTLEAAWPHLQL--- 189
gi|Xenopus_laevis_delta/gamma      VVHMFAVNMFRTLPPSSNPT-----GAEFDPEEDEPTLEAAWPHLQL--- 189
gi|Xenopus_laevis_gamma            VVHMFAVNMFRTLPPSSNPT-----GAEFDPEEDEPTLEAAWPHLQL--- 189
gi|Xenopus_tropicalis_delta/ga     VVHMFAVNMFRTLPPSSNPT-----GAEFDPEEDEPTLEAAWPHLQL--- 189
gi|Mus_musculus_delta              AVTMFSVNLFRTLPPSSNPT-----GAEFDPEEDEPTLEAAWPHLQL--- 204
gi|Rattus_norvegicus_delta         AVTMFSVNLFRTLPPSSNPT-----GAEFDPEEDEPTLEAAWPHLQL--- 232
gi|Felis_catus_delta               AVTMFSVNLFRTLPPSSNPT-----GAEFDPEEDEPTLEAAWPHLQL--- 218
gi|Bos_taurus_delta                AVTMFSVNLFRTLPPSSNPT-----GAEFDPEEDEPTLEAAWPHLQL--- 210
gi|Canis_lupus_familiaris_delt     AVTMFSVNLFRTLPPSSNPT-----GAEFDPEEDEPTLEAAWPHLQL--- 205
gi|Macaca_mulatta_delta            AVTMFSVNLFRTLPPSSNPT-----GAEFDPEEDEPTLEAAWPHLQVPGQ 215
gi|Homo_sapiens_delta              AVTMFSVNLFRTLPPSSNPT-----GAEFDPEEDEPTLEAAWPHLQL--- 212
gi|Ovis_aries_delta                CTPQFSVNLFRTLPPSSNPT-----GAEFDPEEDEPTLEAAWPHLQL--- 209
gi|Falco_peregrinus_delta          AVIMFSVNLFRTLPPSSNPT-----GAEFDPEEDEPTLEAAWPHLQL--- 112
gi|Gallus_gallus_delta             AVIMFSVNLFRTLPPSSNPT-----GAEFDPEEDEPTLEAAWPHLQL--- 207
gi|Alligator_mississippiensis_     AVIMFSVNLFRTLPPSSNPT-----GAEFDPEEDEPTLEAAWPHLQL--- 206
gi|Chrysemys_picta_bellii_delt     AVIMFSVNLFRTLPPSSNPT-----GAEFDPEEDEPTLEAAWPHLQL--- 208
gi|Danio_rerio_delta               AVIMFSVNLFRTLPPSSNPT-----GAEFDPEEDEPTLEAAWPHLQL--- 199
gi|Ambystoma_mexicanum_delta       AVLMFSVNLFRTLPPSSNPT-----GAEFDPEEDEPTLEAAWPHLQL--- 61
gi|Petromyzon_marinus_S4RN43       VVHVFAVNMFRTLPPSSNPT-----GAEFDPEEDEPTLEAAWPHLQL--- 189
jgi|Branchiostoma_floridae_237     AVNMFSINVFRTLPPTSNPN-----GAEFDPEEDEPTLEAAWPHLQI--- 109
jgi|Branchiostoma_floridae_252     AVNMFSINVFRTLPPTSNPN-----GAEFDPEEDEPTLEAAWPHLQI--- 162
gi|Strongylocentrotus_purpurat     AVQMFAINMFRTLPPPSNPH-----GAEFDPEEDEPTLEAAWPHLQL--- 170
gi|Caenorhabditis_elegans_PPTR     AIGMFSTNLFRPLSPPTNPI-----GAEFDPDEDEPTLEAAWPHLQL--- 157
gi|Hydra_vulgaris_delta            ACNMFSVNVFRALPPSMNPA-----GAEFDPEEDEPTLEAAWPHLQI--- 179
gi|Amphimedon_queenslandica_de     AVRMFGINAFRALPPSANPS-----GAEFDPEEDEPALEPSWPHLQI--- 220
gi|Drosophila_melanogaster_B56     AINMFAVNLFRTLPPSSNPN-----GAEFDPEEDEPTLESSWPHLQL--- 293
gi|Dictyostelium_discoideum_ps     VFEMVAVNLFRPLPPRINPY-----GVMYDPEEDEPILEAAWPHIQV--- 218
gi|Dictyostelium_purpureum_N/A     LFEMVAVNLFRPLPPRINPY-----GVMYDPEEDEPILEAAWPHIQV--- 175
gi|Dictyostelium_fasciculatum_     VFEMVASNLFRPLPPRINPY-----GVMYDPEEDEPILEPAWMHIQI--- 188
gi|Polysphondylium_pallidum_N/     LFEMISINLFRPLPPRMNPY-----GVMYDPEEDEPILEPAWLHIQI--- 166
gi|Arabidopsis_thaliana_theta      VVRMVSVNIFRTLNPQPREN---KVIDALDLEEEEPSMDPTWPHLQL--- 183
gi|Arabidopsis_thaliana_eta        VVRMVSANIFRTLNPQPREN---KVIDALDLEEEEPSMDLAWPHLQL--- 204
gi|Arabidopsis_thaliana_zeta       LTKMVAVNLFRTFPSANHES---KILEIHDMDDEEPSLEPAWPHVQV--- 213
gi|Arabidopsis_thaliana_gamma      IAKVAVVNLFRTFPSANHES---KILETLDVDDEEPALEPAWPHLQV--- 192
gi|Oryza                           ITRMISANLFRTLISPPREK---KVLQAFDLEEDEAVMDPAWSHLQI--- 193
gi|Oryza_sativa_zeta               ITRMVSVNLFRTLTTPPREN---K-IEAFDVDDEEPVMDPAWSHLQI--- 193
gi|Oryza_sativa_theta              VIKMVSTNLFRVPNPAPREN---KPLESFDMEEEEPVMDPAWPHLQI--- 197
gi|Oryza_sativa_eta                ITKMVSINLFRSSSPTPREN---KAIEGVDLEEEEPLMDPAWSHLQI--- 186
gi|Arabidopsis_thaliana_delta      ATKMISANLFSN-PHRQWKN---KTPEALDLEEEEGSLNPSWPHLQI--- 171
gi|Arabidopsis_thaliana_N/A        MCRMCAVNLFRVFPPNYRSS---S--GGENDDD-EPMFDPAWPHLQI--- 177
gi|Oryza_sativa_kappa              SSRMFAVNLFRVFPPNYRSS---SSGGGEGEE-EPMFEPAWCHLQL--- 183
gi|Oryza_sativa_N/A                CVRMFAINLFRVFPPKVRSG---AAGAAEADED-EPFFDPSWYHLQA--- 164
gi|Arabidopsis_thaliana_alpha      MVKMISVNIFRCLPPASHEN-TGQEP--ADLEEEEPYLEPSWPHLQL--- 182
gi|Arabidopsis_thaliana_beta       MIKMISVNIFRSLPPASHEN-TGQEP--ADPEEEEPYLEPSWPHLQL--- 182
gi|Oryza_sativa_N/A_2              LVAAISVNVFRPLPPALHESAAAIDPGAAPDDEEEPYLDPAWPHLQL--- 222
gi|Arabidopsis_thaliana_epsilo     LIRMVSANIFRCLPPAYHEN-TGAPPEGNDPEEEEPYLEPWWPHLQL--- 191
gi|Chlamydomonas_reinhardtii_w     IIYMLSNNLFRTLPPVRSHG---DAEGNYDAEEEEPSLDPAWPHLQI--- 180
gi|Schizosaccharomyces_pombe_P     IVHMFAVNVFRPLPPPSNPP-----GEIMDLEEDEPALEVAWPHLHL--- 215
gi|Schizosaccharomyces_pombe_P     VVNTFSLNVFRPISPALNDY-----SSDMFALDDEPFLEPAWPHLEE--- 316
gi|Ashbya_gossypii_RTS1            VVNMFKVNLFRPIPPPVNPIG-----DVYDPDEDEPVNELAWPHMQC--- 278
```

Figure S1. *Cont.*

```
gi|Saccharomyces_cerevisiae_RT      VVNMFKINLFRPIPPPVNPVG-----DIYDPDEDEPVNELAWPHMQA--- 385


gi|Aspergillus_niger_N/A            -------VYEFFLRFIESQDFNTNIAKQYIDHHFVLQLLELFDSEDPRER 312
gi|Aspergillus_nidulans_parA        -------VYEFFLRFIESQDFNTNIAKAYIDHHFVLQLLELFDSEDPRER 314
gi|Mus_musculus_alpha               -------VYEFFLRFLESPDFQPSIAKRYIDQKFVQQLLELFDSEDPRER 204
gi|Rattus_norvegicus_alpha          -------VYEFFLRFLESPDFQPSIAKRYIDQKFVQQLLELFDSEDPRER 204
gi|Macaca_mulatta_alpha             -------VYEFFLRFLESPDFQPSIAKRYIDQKFVQQLLELFDSEDPRER 204
gi|Homo_sapiens_alpha               -------VYEFFLRFLESPDFQPSIAKRYIDQKFVQQLLELFDSEDPRER 204
gi|Ovis_aries_alpha                 -------VYEFFLRFLESPDFQPSIAKRYIDQKFVQQLLELFDSEDPRER 207
gi|Bos_taurus_alpha                 -------VYEFFLRFLESPDFQPSIAKRYIDQKFVQQLLELFDSEDPRER 207
gi|Felis_catus_alpha                -------VYEFFLRFLESPDFQPSIAKRYIDQKFVQQLLELFDSEDPRER 120
gi|Canis_lupus_familiaris_alph      -------VYEFFLRFLESPDFQPSIAKRYIDQKFVQQLLELFDSEDPRER 120
gi|Falco_peregrinus_alpha           -------VYEFFLRFLESPDFQPSIAKRYIDQKFVQQLLELFDSEDPRER 194
gi|Gallus_gallus_alpha              -------VYEFFLRFLESPDFQPSIAKRFIDQKFVQQLLELFDSEDPRER 194
gi|Alligator_mississippiensis_      -------VYEFFLRFLESLDFQPSIAKRYIDQKFVQQLLELFDSEDPRER 204
gi|Chrysemys_picta_bellii_alph      -------VYEFFLRFLESPDFQPSIAKRYIDQKFVQQLLELFDSEDPRER 194
gi|Xenopus_laevis_alpha             -------VYEFLLRFLESPDFQPSIAKRFVDQKFVQQLLDLFDSEDPRER 193
gi|Xenopus_tropicalis_alpha         -------VYEFFLRFLESPDFQPSIAKRFVDQKFVQQLLELFDSEDPRER 193
gi|Ambystoma_mexicanum_alpha        -------VYEFFLRFLESQDFQPSIAKRYVDQKFVQQLLELFDSEDPRER 120
gi|Danio_rerio_alpha                -------VYEFFLRFLENPDFQPSTAKRYIDQKFVLQLLELFDSEDPRER 196
gi|Petromyzon_marinus_S4RHV1        -------VYEFFLRFLEQQEFQPSIAKKYIDQKFVLQLLELFDSEDPRER 194
gi|Mus_musculus_epsilon             -------VYEFFIRFLESQEFQPSIAKKYIDQKFVLQLLELFDSEDPRER 196
gi|Rattus_norvegicus_epsilon        -------VYEFFIRFLESQEFQPSIAKKYIDQKFVLQLLELFDSEDPRER 196
gi|Macaca_mulatta_epsilon           -------VYEFFIRFLESQEFQPSIAKKYIDQKFVLQLLELFDSEDPRER 196
gi|Homo_sapiens_epsilon             -------VYEFFIRFLESQEFQPSIAKKYIDQKFVLQLLELFDSEDPRER 196
gi|Ovis_arie_epsilon                -------VYEFFIRFLESQEFQPSIAKKYIDQKFVLQLLELFDSEDPRER 196
gi|Bos_taurus_epsilon               -------VYEFFIRFLESQEFQPSIAKKYIDQKFVLQLLELFDSEDPRER 196
gi|Canis_lupus_familiaris_epsi      -------VYEFFIRFLESQEFQPSIAKKYIDQKFVLQLLELFDSEDPRER 196
gi|Falco_peregrinus_epsilon         -------VYEFFIRFLESQEFQPSIAKKYIDQKFVLQLLELFDSEDPRER 196
gi|Gallus_gallus_epsilon            -------VYEFFIRFLESQEFQPSIAKKYIDQKFVLQLLELFDSEDPRER 196
gi|Felis_catus_epsilon              -------VYEFFIRFLESQEFQPSIAKKYIDQKFVLQLLELFDSEDPRER 196
gi|Chrysemys_picta_bellii_epsi      -------VYEFFIRFLESQEFQPSIAKKYIDQKFVLQLLELFDSEDPRER 196
gi|Xenopus_laevis_epsilon           -------IYEFFIRFLESQEFQPSIAKKYIDQKFVLQLLELFDSEDPRER 196
gi|Xenopus_tropicalis_epsilon       -------VYEFFIRFLESQEFQPSIAKKYIDQKFVLQLLELFDSEDPRER 196
gi|Alligator_mississippiensis_      -------VYEFFIRFLESQEFQPSIAKKYIDQKFVLQLLELFDSEDPRER 185
gi|Ambystoma_mexicanum_epsilon      -------VYEFFIRFLESQEFQPSIAKKYIDQKFVLQLLELFDSEDPRER 192
gi|Danio_rerio_epsilon              -------VYEFFIRFLESQEFQPSAAKKYIDQKFVLQLLELFDSEDPRER 196
gi|Mus_musculus_beta                -------VYEFFLRFLESPDFQPSVAKRYVDQKFVLMLLELFDSEDPRER 210
gi|Rattus_norvegicus_beta           -------VYEFFLRFLESPDFQPSVAKRYVDQKFVLMLLELFDSEDPRER 210
gi|Felis_catus_beta                 -------VYEFFLRFLESPDFQPSVAKRYVDQKFVLMLLELFDSEDPRER 210
gi|Canis_lupus_familiaris_beta      -------VYEFFLRFLESPDFQPSVAKRYVDQKFVLMLLELFDSEDPRER 210
gi|Homo_sapiens_beta                -------VYEFFLRFLESPDFQPSVAKRYVDQKFVLMLLELFDSEDPRER 210
gi|Bos_taurus_beta                  -------VYEFFLRFLESPDFQPSVAKRYVDQKFVLMLLELFDSEDPRER 210
gi|Ovis_aries_beta                  -------VYEFFLRFLESPDFQPSVAKRYVDQKFVLMLLELFDSEDPRER 210
gi|Macaca_mulatta_beta              -------GFFIRVMDFISP-----------------ISLDNFETYVAQSR 169
gi|Xenopus_laevis_beta              --------------------------------------------------
gi|Alligator_mississippiensis_      -------VYEFFLRFLESPDFQPSVAKRYVDQKFVLMLLELFDSEDPRER 210
gi|Chrysemys_picta_bellii_beta      -------VYEFFLRFLESPDFQPSVAKRYVDQKFVLLLLELFDSEDPRER 210
gi|Xenopus_tropicalis_beta          -------VYEFFLRFLESQDFQPSLAKRYVDQKFVLMLLELFDSEDPRER 208
gi|Danio_rerio_beta                 -------VYEFFLRFLESPDFQPSLAKRYVDQKFVLQLLELFDSEDPRER 209
gi|Ambystoma_mexicanum_beta         -------VYEFFLRFLESPDFQPSVAKRYVDQRFVMTLLDLFDSEDPRER 79
gi|Petromyzon_marinus_S4RGA7        --------------------------------------------------
gi|Strongylocentrotus_purpurat      -------VYEFFLRFLESPEFQPSIAKKYIDQKFVMHLLELFDSEDPRER 192
gi|Drosophila_melanogaster_wdb      -------VYEVFLRFLESQDFQATIGKRVIDQKFVLQLLELFDSEDPRER 191
jgi|Branchiostoma_floridae_237      -------VYEFFLRFLESPDFQPSIAKKYIDQKFVLQLLELFDSEDPRER 147
gi|Hydra_vulgaris_alpha             -------VFEFFLRVLESPEFQPTIAKKYIDQKFVLQLLELFDSEDPRER 200
jgi|Branchiostoma_floridae_112      --------------------------------------------------
jgi|Branchiostoma_floridae_284      -------VYEIVLRFLESPEFQPTIAKKYVDQRFVMQLLELFDSEDPRER 160
gi|Caenorhabditis_elegans_PPTR      -------VYELFLRFLESPDFQASIGKKYIDQRFVLKLLDLFDSEDPRER 221
gi|Amphimedon_queenslandica_be      -------VYAFFLRFLESPEMQASIAKKYIDQQFVIQLLELFDSLDPRER 214
```

Figure S1. *Cont.*

```
gi|Chrysemys_picta_bellii_gamm       -------VYEFFLRFLESPDFQPNVAKKYIDQKFVLQLLELFDSEDPRER 179
gi|Chrysemys_picta_bellii_delt       -------VYEFFLRFLESPDFQPNVAKKYIDQKFVLQLLELFDSEDPRER 104
gi|Falco_peregrinus_gamma            -------VYEFFLRFLESPDFQPNIAKKYIDQKFVLQLLELFDSEDPRER 179
gi|Falco_peregrinus_delta/gamm       -------VYEFFLRFLESPDFQPNIAKKYIDQKFVLQLLELFDSEDPRER 104
gi|Gallus_gallus_gamma               -------VYEFFLRFLESPDFQPNIAKKYIDQKFVLQLLELFDSEDPRER 179
gi|Alligator_mississippiensis_       -------VYEFFLRFLESPDFQPNIAKKYIDQKFVLQLLELFDSEDPRER 179
gi|Alligator_mississippiensis_       -------VYEFFLRFLESPDFQPNIAKKYIDQKFVLQLLELFDSEDPRER 104
gi|Ambystoma_mexicanum_gamma         -------VYEFFLRFLESPDFQPNVAKKYIDQKFVLQLLELFDSEDPRER 180
gi|Mus_musculus_gamma                -------VYEFFLRFLESPDFQPNIAKKYIDQKFVLQLLELFDSEDPRER 179
gi|Rattus_norvegicus_gamma           -------VYEFFLRFLESPDFQPNIAKKYIDQKFVLQLLELFDSEDPRER 179
gi|Ovis_aries_gamma                  -------VYEFFLRLLESPDFQPNIAKKYIDQKFVLQLLELFDSEDPRER 179
gi|Bos_taurus_gamma                  -------VYEFFLRLLESPDFQPNIAKKYIDQKFVLQLLELFDSEDPRER 179
gi|Canis_lupus_familiaris_gamm       -------VYEFFLRFLESPDFQPNIAKKYIDQKFVLQLLELFDSEDPRER 179
gi|Macaca_mulatta_gamma              -------VYEFFLRFLESPDFQPNIAKKYIDQKFVLQLLELFDSEDPRER 179
gi|Homo_sapiens_gamma                -------VYEFFLRFLESPDFQPNIAKKYIDQKFVLQLLELFDSEDPRER 169
gi|Felis_catus_gamma                 -------VYEFFLRFLESPDFQPNIAKKYIDQKFVLQLLELFDSEDPRER 226
gi|Canis_lupus_familiaris_delt       -------VYEFFLRFLESPDFQPNIAKKYIDQKFVLQLLELFDSEDPRER 226
gi|Macaca_mulatta_delta/gamma        -------VYEFFLRFLESPDFQPNIAKKYIDQKFVLQLLELFDSEDPRER 461
gi|Bos_taurus_delta/gamma            -------VYEFFLRLLESPDFQPNIAKKYIDQKFVLQLLELFDSEDPRER 233
gi|Mus_musculus_delta/gamma          -------VYEFFLRFLESPDFQPNIAKKYIDQKFVLQLLELFDSEDPRER 236
gi|Rattus_norvegicus_delta/gam       -------VYEFFLRFLESPDFQPNIAKKYIDQKFVLQLLELFDSEDPRER 302
gi|Danio_rerio_gamma                 -------VYEFFLRFLESPDFQPNIAKKYIDQKFVLQLLELFDSEDPRER 179
gi|Danio_rerio_delta/gamma           -------VYEFFLRFLESPDFQPNIAKKYIDQKFVLQLLELFDSEDPRER 232
gi|Xenopus_laevis_delta/gamma        -------VYEFFLRFLESPDFQPNVAKKYIDQKFVLQLLELFDSEDPRER 232
gi|Xenopus_laevis_gamma              -------VYEFFLRFLESPDFQPNVAKKYIDQKFVLQLLELFDSEDPRER 232
gi|Xenopus_tropicalis_delta/ga       -------VYEFFLRFLESPDFQPNVAKKYIDQKFVLQLLELFDSEDPRER 232
gi|Mus_musculus_delta                -------VYEFFLRFLESPDFQPNIAKKYIDQKFVLALLDLFDSEDPRER 247
gi|Rattus_norvegicus_delta           -------VYEFFLRFLESPDFQPNIAKKYIDQKFVLALLDLFDSEDPRER 275
gi|Felis_catus_delta                 -------VYEFFLRFLESPDFQPNIAKKYIDQKFVLALLDLFDSEDPRER 261
gi|Bos_taurus_delta                  -------VYEFFLRFLESPDFQPNIAKKYIDQKFVLALLDLFDSEDPRER 253
gi|Canis_lupus_familiaris_delt       -------VYEFFLRFLESPDFQPNIAKKYIDQKFVLALLDLFDSEDPRER 248
gi|Macaca_mulatta_delta              GGQVGPGCREWGSRGXXXXXXXX-XXKKYIDQKFVLALLDLFDSEDPRER 264
gi|Homo_sapiens_delta                -------VYEFFLRFLESPDFQPNIAKKYIDQKFVLALLDLFDSEDPRER 255
gi|Ovis_aries_delta                  -------VYEFFLRFLESPDFQPNIAKKYIDQKFVLALLDLFDSEDPRER 252
gi|Falco_peregrinus_delta            -------VYEFFLRFLESPDFQPNVAKKYIDQKFVLSLLDLFDSEDPRER 155
gi|Gallus_gallus_delta               -------VYEFFLRFLESPDFQPNIAKKYIDQKFVLSLLDLFDSEDPRER 250
gi|Alligator_mississippiensis_       -------VYEFFLRFLESPDFQPNVAKKYIDQKFVLSLLDLFDSEDPRER 249
gi|Chrysemys_picta_bellii_delt       -------VYEFFLRFLESPDFQPNIAKKYIDQKFVLSLLDLFDSEDPRER 251
gi|Danio_rerio_delta                 -------VYEFFLRFLESPDFQPNIAKKYIDQKFVLSLLELFDSEDPRER 242
gi|Ambystoma_mexicanum_delta         -------VYEFFLRFLESPDFQPNVAKKYIDQKFVMSLLDLFDSEDPRER 104
gi|Petromyzon_marinus_S4RN43         -------VYEFFLRFLESPDFQPNVAKKYIDQKFVLQLLELFDSEDPRER 232
jgi|Branchiostoma_floridae_237       -------VYEFFLRFLESPDFQPNTAKKYIDQKFVLQLLDLFDSEDPRER 152
jgi|Branchiostoma_floridae_252       -------VYEFFLRFLESPDFQPNTAKKYIDQKFVLQLLDLFDSEDPRER 205
gi|Strongylocentrotus_purpurat       -------VYEYFLRFLESPDFQPNVAKKYIDQKFVLALLELFDSEDPRER 213
gi|Caenorhabditis_elegans_PPTR       -------VYEFFLRFLECPDFQSQVAKRYIDQNFILRLLMIMDSEDPRER 200
gi|Hydra_vulgaris_delta              -------VYEFFLRFLESTDFQPNIAKKYIDQRFLINILNLFDSEDPRER 222
gi|Amphimedon_queenslandica_de       -------VYEFFLRFLESQDFQPGIAKKFIDQKFVGQLLELFDSEDPRER 263
gi|Drosophila_melanogaster_B56       -------VYELFLRFLESPDFQPSMAKRFIDHQFVLQLLDLFDSEDPRER 336
gi|Dictyostelium_discoideum_ps       -------VYEVLLRFIDSPTFNTHIAKNYVDDRFVLQMLDLFDSEDPRER 261
gi|Dictyostelium_purpureum_N/A       -------VYEVLLRFIDSPTFNTHIAKNYVDDKFVLQMLELFDSEDPRER 218
gi|Dictyostelium_fasciculatum_       -------VYELLLRFVDSTNFNTHIAKNYVDQKFVLQLLDLFDSEDPRER 231
gi|Polysphondylium_pallidum_N/       -------VYEILLRFIDSPIFNTHIAKAFVDQKFVLQLLDLFDSEDPRER 209
gi|Arabidopsis_thaliana_theta        -------VYEILLRLIASPETDTKLAKKYIDQSFVSRLLDLFDSEDPRER 226
gi|Arabidopsis_thaliana_eta          -------VYELFLRFVASPETDTKLAKRYIDQSFVLRLLDLFDSEDPRER 247
gi|Arabidopsis_thaliana_zeta         -------VYEILLRFVASPMTDAKLAKRYIDHSFVLKLLDLFDSEDQRER 256
gi|Arabidopsis_thaliana_gamma        -------VYELLLRFVASPMTDAKLAKRYIDHSFVLKLLDLFDSEDQRER 235
gi|Oryza                             -------VYELLLKFIQSPETDAKLAKRYIDHSFILRLLDIFDSEDPRER 236
gi|Oryza_sativa_zeta                 -------VYELFLRFIQSPETDAKLAKRYIDHSFVLRLLDLFDSEDPRER 236
gi|Oryza_sativa_theta                -------VYELFLRFVQSPETDAKLAKRYVDHGFIIKLLDLFDSEDPRER 240
gi|Oryza_sativa_eta                  -------VYEVFLRFVASQETDAKLAKRYIDHSFILRLLDLFDSEDPRER 229
gi|Arabidopsis_thaliana_delta        -------VYEFLLRIVASPNTDPKISKKYIDHTFVLKLLDLFDSEDPRER 214
```

Figure S1. *Cont.*

```
gi|Arabidopsis_thaliana_N/A          -------VYDLLLKFITSPCLDAKVAKKYLDHAFIVRLLDLFDSEDPRER 220
gi|Oryza_sativa_kappa                -------VYELLLKFIGSSSLDAKVGKKYFDHSFIVKLLNLLDSEDPRER 226
gi|Oryza_sativa_N/A                  -------VYELLLRFVMSPVVDVKIARKYMDNSFVSRLLDLFDSDDPRER 207
gi|Arabidopsis_thaliana_alpha        -------IYELLLRYIVPSDTDTKVAKRYIDHSFVLRLLELFETEDPRER 225
gi|Arabidopsis_thaliana_beta         -------VYELLLRYVVSTDTDTKVAKRYIDHSFVLKLLDLFDSEDPRER 225
gi|Oryza_sativa_N/A_2                -------VYELLLRYVVSPDTDTKVAKRYVDHAFVLRLLDLFDSEDPRER 265
gi|Arabidopsis_thaliana_epsilo       -------VYELLLRYVVSSEIEPKTAKKFINHTFVSRLLDLFDSEDPRER 234
gi|Chlamydomonas_reinhardtii_w       -------VYEFLLRYVVSNDTDAKIAKKYIDQQFVLKLLELFDSEDPRER 223
gi|Schizosaccharomyces_pombe_P       -------VYDFFLRFFESPSLNTSVAKVYINQKFIRKLLVLFDSEDPRER 258
gi|Schizosaccharomyces_pombe_P       -------VYLLFIKFLESPDFRASKAKSLVDRRFFNRLLALFDTEDPRER 359
gi|Ashbya_gossypii_RTS1              -------VYEFFLRFVESPDFNHQIAKQFIDQEFILKLLELFDSEDIRER 321
gi|Saccharomyces_cerevisiae_RT       -------VYEFFLRFVESPDFNHQIAKQYIDQDFILKLLELFDSEDIRER 428


gi|Aspergillus_niger_N/A             DFLKTTLHRIYGKFLNLR-----SYIRRSINNVFFQFTYETERFNGIAEL 357
gi|Aspergillus_nidulans_parA         DFLKTTLHRIYGKFLNLR-----SYIRRSINNVFFQFSYETERFNGIAEL 359
gi|Mus_musculus_alpha                DFLKTVLHRIYGKFLGLR-----AFIRKQINNIFLRFIYETEHFNGVAEL 249
gi|Rattus_norvegicus_alpha           DFLKTVLHRIYGKFLGMR-----AFIRKQTNNIFLRFIYETEHFNGVAEL 249
gi|Macaca_mulatta_alpha              DFLKTVLHRIYGKFLGLR-----AFIRKQINNIFLRFIYETEHFNGVAEL 249
gi|Homo_sapiens_alpha                DFLKTVLHRIYGKFLGLR-----AFIRKQINNIFLRFIYETEHFNGVAEL 249
gi|Ovis_aries_alpha                  DFLKTVLHRIYGKFLGLR-----AFIRKQINNIFLRFIYETEHFNGVAEL 252
gi|Bos_taurus_alpha                  DFLKTVLHRIYGKFLGLR-----AFIRKQINNIFLRFIYETEHFNGVAEL 252
gi|Felis_catus_alpha                 DFLKTVLHRIYGKFLGLR-----AFIRKQINNIFLRFIYETEHFNGVAEL 165
gi|Canis_lupus_familiaris_alph       DFLKTVLHRIYGKFLGLR-----AFIRKQINNIFLRFIYETEHFNGVAEL 165
gi|Falco_peregrinus_alpha            DFLKTVLHRIYGKFLGLR-----AFIRKQINNIFLRFIYETEHFNGVAEL 239
gi|Gallus_gallus_alpha               DFLKTVLHRIYGKFLGLR-----AFIRKQINNIFLRFIYETEHFNGVAEL 239
gi|Alligator_mississippiensis_       DFLKTVLHRIYGKFLGLR-----AFIRKQINNIFLRFIYETEHFNGVAEL 249
gi|Chrysemys_picta_bellii_alph       DFLKTVLHRIYGKFLGLR-----AFIRKQINNIFLRFIYETEHFNGVAEL 239
gi|Xenopus_laevis_alpha              DFLKTVLHRIYGKFLGLR-----AFIRKQINNIFLRFIYETDHFNGVAEL 238
gi|Xenopus_tropicalis_alpha          DFLKTVLHRIYGKFLGLR-----AFIRKQINNIFLRFIYETEHFNGVAEL 238
gi|Ambystoma_mexicanum_alpha         DFLKTVLHRIYGKFLGLR-----AFIRKQINNIFLRFIYETDHFNGVAEL 165
gi|Danio_rerio_alpha                 EFLKTILHRIYGKFLGLR-----AFIRKQINNIFLRFIYETEHFNGVAEL 241
gi|Petromyzon_marinus_S4RHV1         DFLRTVLHRIYGKLLGLR-----AFIRTQINNILLRYIYETERFNGVGEM 239
gi|Mus_musculus_epsilon              DYLKTVLHRIYGKFLGLR-----AFIRKQINNIFLRFVYETEHFNGVAEL 241
gi|Rattus_norvegicus_epsilon         DYLKTVLHRIYGKFLGLR-----AFIRKQINNIFLRFVYETEHFNGVAEL 241
gi|Macaca_mulatta_epsilon            DYLKTVLHRIYGKFLGLR-----AFIRKQINNIFLRFVYETEHFNGVAEL 241
gi|Homo_sapiens_epsilon              DYLKTVLHRIYGKFLGLR-----AFIRKQINNIFLRFVYETEHFNGVAEL 241
gi|Ovis_arie_epsilon                 DYLKTVLHRIYGKFLGLR-----AFIRKQINNIFLRFVYETEHFNGVAEL 241
gi|Bos_taurus_epsilon                DYLKTVLHRIYGKFLGLR-----AFIRKQINNIFLRFVYETEHFNGVAEL 241
gi|Canis_lupus_familiaris_epsi       DYLKTVLHRIYGKFLGLR-----AFIRKQINNIFLRFVYETEHFNGVAEL 241
gi|Falco_peregrinus_epsilon          DYLKTVLHRIYGKFLGLR-----AFIRKQINNIFLRFVYETEHFNGVAEL 241
gi|Gallus_gallus_epsilon             DYLKTVLHRIYGKFLGLR-----AFIRKQINNIFLRFVYETEHFNGVAEL 241
gi|Felis_catus_epsilon               DYLKTVLHRIYGKFLGLR-----AFIRKQINNIFLRFVYETEHFNGVAEL 241
gi|Chrysemys_picta_bellii_epsi       DYLKTVLHRIYGKFLGLR-----AFIRKQINNIFLRFVYETEHFNGVAEL 241
gi|Xenopus_laevis_epsilon            DYLKTVLHRIYGKFLGLR-----AFIRKQINNIFLRFVYETEHFNGVAEL 241
gi|Xenopus_tropicalis_epsilon        DYLKTVLHRIYGKFLGLR-----AFIRKQINNIFLRFVYETEHFNGVAEL 241
gi|Alligator_mississippiensis_       DYLKTVLHRIYGKFLGLR-----AFIRKQINNIFLRFVYETEHFNGVAEL 230
gi|Ambystoma_mexicanum_epsilon       DYLKTVLHRIYGKFLGLR-----AFIRKQINNIFLRFVYETEHFNGVAEL 237
gi|Danio_rerio_epsilon               DCLKTVLHRIYGKFLGLR-----AFIRKQINNIFLCFVYETERFNGVAEL 241
gi|Mus_musculus_beta                 EYLKTILHRVYGKFLGLR-----AYIRKQCNHIFLRFIYELEHFNGVAEL 255
gi|Rattus_norvegicus_beta            EYLKTILHRVYGKFLGLR-----AYIRKQCNHIFLRFIYELEHFNGVAEL 255
gi|Felis_catus_beta                  EYLKTILHRVYGKFLGLR-----AYIRKQCSHIFLRFIYELEHFNGVAEL 255
gi|Canis_lupus_familiaris_beta       EYLKTILHRVYGKFLGLR-----AYIRKQCSHIFLRFIYEFEHFNGVAEL 255
gi|Homo_sapiens_beta                 EYLKTILHRVYGKFLGLR-----AYIRKQCNHIFLRFIYEFEHFNGVAEL 255
gi|Bos_taurus_beta                   EYLKTILHRVYGKFLGLR-----AYIRKQCSHIFLRFIYEFEHFNGVAEL 255
gi|Ovis_aries_beta                   EYLKTILHRVYGKFLGLR-----AYIRKQCSHIFLRFIYEFEHFNGIAEL 255
gi|Macaca_mulatta_beta               CAKCLSFSWLTGKSLGLRGTGPTLLALSPPAFLFCRFIYEFEHFNGVAEL 219
gi|Xenopus_laevis_beta               --------------------------------------------------
gi|Alligator_mississippiensis_       EYLKTILHRVYGKFLGLR-----AYIRKQCNNIFLRFIYETEHFNGVAEL 255
gi|Chrysemys_picta_bellii_beta       EYLKTILHRVYGKFLGLR-----AYIRKQCNNIFLRFIYETEHFNGVAEL 255
gi|Xenopus_tropicalis_beta           EYLKTILHRVYGKFLGLR-----AYIRKQCNNIFLRFIYETERFNGVAEL 253
```

Figure S1. *Cont.*

gi|Danio_rerio_beta	EYLKTILHRVYGKLLGLR-----AYIRKQINNIFLRFIYETERFNGVAEL	254
gi|Ambystoma_mexicanum_beta	EYLKTILHRVYGKFLGLR-----AYIRKQCNNIFLRFVYETEHFNGVAEL	124
gi|Petromyzon_marinus_S4RGA7	---------------------------------VCCRFIYETEHFNGVAEL	18
gi|Strongylocentrotus_purpurat	DFLKTVLHRIYGKFLGLR-----AFIRKQINHIFLRFIYETEHFNGVGEL	237
gi|Drosophila_melanogaster_wdb	DFLKTVLHRIYGKFLGLR-----AFIRKQINNIFLRFIYETEHFNGVGEL	236
jgi|Branchiostoma_floridae_237	DFLKTVLHRIYGKFLGLR-----AFIRKQINHIFLKFIYETEHFNGVGEL	192
gi|Hydra_vulgaris_alpha	EFLKTVLHRIYGKFLGLR-----AYIRKQINHMFLKFIYETEHFSGVGEL	245
jgi|Branchiostoma_floridae_112	--	
jgi|Branchiostoma_floridae_284	DLLKTMLHRIYGKFLGLR-----AFIRKQINHIFLKFIYETEHFNGIGEL	205
gi|Caenorhabditis_elegans_PPTR	DFLKTVLHRIYGKFLGLR-----AFIRKHINNMFLRFVYETDSFNGVGEL	266
gi|Amphimedon_queenslandica_be	DYLKTVLHRIYGKFLGLR-----AFIRKHINYIFLRFVYETERFAGIAEL	259
gi|Chrysemys_picta_bellii_gamm	DFLKTTLHRIYGKFLGLR-----AYIRKQINNIFYRFIYETEHHNGIAEL	224
gi|Chrysemys_picta_bellii_delt	DFLKTTLHRIYGKFLGLR-----AYIRKQINNIFYRFIYETEHHNGIAEL	149
gi|Falco_peregrinus_gamma	DFLKTTLHRIYGKFLGLR-----AYIRKQINNIFYRFIYETEHHNGIAEL	224
gi|Falco_peregrinus_delta/gamm	DFLKTTLHRIYGKFLGLR-----AYIRKQINNIFYRFIYETEHHNGIAEL	149
gi|Gallus_gallus_gamma	DFLKTTLHRIYGKFLGLR-----AYIRKQINNIFYRFIYETEHHNGIAEL	224
gi|Alligator_mississippiensis_	DFLKTTLHRIYGKFLGLR-----AYIRKQINNIFYRFIYETEHHNGIAEL	224
gi|Alligator_mississippiensis_	DFLKTTLHRIYGKFLGLR-----AYIRKQINNIFYRFIYETEHHNGIAEL	149
gi|Ambystoma_mexicanum_gamma	DFLKTTLHRIYGKFLGLR-----AYIRKQINNIFYRFIYETEHHNGIAEL	225
gi|Mus_musculus_gamma	DFLKTTLHRIYGKFLGLR-----AYIRKQINNIFYRFIYETEHHNGIAEL	224
gi|Rattus_norvegicus_gamma	DFLKTTLHRIYGKFLGLR-----AYIRKQINNIFYRFIYETEHHNGIAEL	224
gi|Ovis_aries_gamma	DFLKTTLHRIYGKFLGLR-----AYIRKQINNIFYRFIYETEHHNGIAEL	224
gi|Bos_taurus_gamma	DFLKTTLHRIYGKFLGLR-----AYIRKQINNIFYRFIYETEHHNGIAEL	224
gi|Canis_lupus_familiaris_gamm	DFLKTTLHRIYGKFLGLR-----AYIRKQINNIFYRFIYETEHHNGIAEL	224
gi|Macaca_mulatta_gamma	DFLKTTLHRIYGKFLGLR-----AYIRKQINNIFYRFIYETEHHNGIAEL	224
gi|Homo_sapiens_gamma	DFLKTTLHRIYGKFLGLR-----AYIRKQINNIFYRFIYETEHHNGIAEL	214
gi|Felis_catus_gamma	DFLKTTLHRIYGKFLGLR-----AYIRKQINNIFYRFIYETEHHNGIAEL	271
gi|Canis_lupus_familiaris_delt	DFLKTTLHRIYGKFLGLR-----AYIRKQINNIFYRFIYETEHHNGIAEL	271
gi|Macaca_mulatta_delta/gamma	DFLKTTLHRIYGKFLGLR-----AYIRKQINNIFYRFIYETEHHNGIAEL	506
gi|Bos_taurus_delta/gamma	DFLKTTLHRIYGKFLGLR-----AYIRKQINNIFYRFIYETEHHNGIAEL	278
gi|Mus_musculus_delta/gamma	DFLKTTLHRIYGKFLGLR-----AYIRKQINNIFYRFIYETEHHNGIAEL	281
gi|Rattus_norvegicus_delta/gam	DFLKTTLHRIYGKFLGLR-----AYIRKQINNIFYRFIYETEHHNGIAEL	347
gi|Danio_rerio_gamma	DFLKTTLHRIYGKFLGLR-----AYIRKQINNIFYRFIYETEHHNGIAEL	224
gi|Danio_rerio_delta/gamma	DFLKTTLHRIYGKFLGLR-----AYIRKQINNIFYRFIYETEHHNGIAEL	277
gi|Xenopus_laevis_delta/gamma	DFLKTTLHRIYGKFLGLR-----AYIRKQINNIFYRFIYETEHHNGIAEL	277
gi|Xenopus_laevis_gamma	DFLKTTLHRIYGKFLGLR-----AYIRKQINNIFYRFIYETEHHNGIAEL	277
gi|Xenopus_tropicalis_delta/ga	DFLKTTLHRIYGKFLGLR-----AYIRKQINNIFYRFIYETEHHNGIAEL	277
gi|Mus_musculus_delta	DFLKTILHRIYGKFLGLR-----AYIRRQINHIFYRFIYETEHHNGIAEL	292
gi|Rattus_norvegicus_delta	DFLKTILHRIYGKFLGLR-----AYIRRQINHIFYRFIYETEHHNGIAEL	320
gi|Felis_catus_delta	DFLKTILHRIYGKFLGLR-----AYIRRQINHIFYRFIYETEHHNGIAEL	306
gi|Bos_taurus_delta	DFLKTILHRIYGKFLGLR-----AYIRRQINHIFYRFIYETEHHNGIAEL	298
gi|Canis_lupus_familiaris_delt	DFLKTILHRIYGKFLGLR-----AYIRRQINHIFYRFIYETEHHNGIAEL	293
gi|Macaca_mulatta_delta	DFLKTILHRIYGKFLGLR-----AYIRRQINHIFYRFIYETEHHNGIAEL	309
gi|Homo_sapiens_delta	DFLKTILHRIYGKFLGLR-----AYIRRQINHIFYRFIYETEHHNGIAEL	300
gi|Ovis_aries_delta	DFLKTILHRIYGKFLGLR-----AYIRRQINHIFYRFIYETEHHNGIAEL	297
gi|Falco_peregrinus_delta	DFLKTILHRIYGKFLGLR-----AYVRRQINNIFYRFIYETEHHNGIAEL	200
gi|Gallus_gallus_delta	DFLKTILHRIYGKFLGLR-----AYVRRQINNIFYRFIYETEHHNGIAEL	295
gi|Alligator_mississippiensis_	DFLKTILHRIYGKFLGLR-----AYVRRQINNIFYRFIYETEHHNGIAEL	294
gi|Chrysemys_picta_bellii_delt	DFLKTILHRIYGKFLGLR-----AYVRRQINNIFYRFIYETEHHNGIAEL	296
gi|Danio_rerio_delta	DFLKTILHRIYGKFLGLR-----AYIRRQINNIFYRFIYETEHHNGIAEL	287
gi|Ambystoma_mexicanum_delta	DFLKTILHRIYGKFLGLR-----AYIRRQINNIFYRFIYETEHHNGIAEL	149
gi|Petromyzon_marinus_S4RN43	DFLKTTLHRIYGKFLGLR-----AYIRKQINNIFYRFIYETEHHNGIAEL	277
jgi|Branchiostoma_floridae_237	DFLKTTLHRIYGKFLGLR-----AYIRKQINNIFYRFIYETEHHNGIAEL	197
jgi|Branchiostoma_floridae_252	DFLKTTLHRIYGKFLGLR-----AYIRKQINNIFYRFIYETEHHNGIAEL	250
gi|Strongylocentrotus_purpurat	DFLKTTLHRIYGKFLGLR-----AYIRKQINNIFYRFVYETEHHNGIAEL	258
gi|Caenorhabditis_elegans_PPTR	DFLKTTLHRIYGKFLGHR-----AYIRKQINNIFYSFIYETERHNGIAEL	245
gi|Hydra_vulgaris_delta	DFLKTILHRIYGKFLNLR-----AYIRKQINNLFYRFIYETEHHNGIAEL	267
gi|Amphimedon_queenslandica_de	DFLKTTLHRIYGKFLGLR-----AHIRKQISNIFYRFIYETEKHNGVAEL	308
gi|Drosophila_melanogaster_B56	DFLKTVLHRIYGKFLGLR-----AFIRKQINNVFYRFIYETEHHNGIAEL	381
gi|Dictyostelium_discoideum_ps	DYLKTTLHRIYGKFLGLR-----GFIRTAIRNLFCTFVYESHQHNGISEI	306
gi|Dictyostelium_purpureum_N/A	DYLKTTLHRIYGKFLGLR-----SFIRTAIRNLFCTFVYESHQHNGISEI	263

Figure S1. *Cont.*

```
gi|Dictyostelium_fasciculatum_      DYLKTTLHRIYGKFIALR-----GYIRTAIRDLFCTFVYESSQHNGVSEI 276
gi|Polysphondylium_pallidum_N/      DYLKTTLHRIYGKFIALR-----GFIRTAIKDLFCTFVYESYQHNGVSEI 254
gi|Arabidopsis_thaliana_theta       DCLKTVLHRIYGKFMVHR-----PFIRKSINNIFYRFVFETEKHNGIAEF 271
gi|Arabidopsis_thaliana_eta         DCLKTILHRIYGKFMVHR-----PFIRKSINNIFYRFVFETEKHNGIAEF 292
gi|Arabidopsis_thaliana_zeta        EYLKTILHRVYGKFMVHR-----PYIRKAINNIFYRFISETEKHNGIAEL 301
gi|Arabidopsis_thaliana_gamma       EYLKTILHRIYGKFMVHR-----PFIRKAINNIFYRFIFETEKHNGIAEL 280
gi|Oryza                            EYLKMTLHRIYGKFMVYR-----PFIRKAINNIFYQFIYETEKHNGIAEL 281
gi|Oryza_sativa_zeta                EYLKTILHRVYGKFMVHR-----PFIRKAINNIFYQFIYETEKHNGIAEL 281
gi|Oryza_sativa_theta               EYLKTILHRIYGKFMVHR-----PFIRKAINNIFYRFIFETEKHNGIAEL 285
gi|Oryza_sativa_eta                 DYLKTILHRIYGKFMVHR-----PFIRKAINNIFYRFIFETEKHNGVAEL 274
gi|Arabidopsis_thaliana_delta       EYLKTILHRIYGRFMVHR-----PFIRKTMNNILYDFIFETGKHSGIAEF 259
gi|Arabidopsis_thaliana_N/A         ECLKTILHRVYGKFMVHR-----PFVRKSMSNIFYRFVFETEKHSGIAEL 265
gi|Oryza_sativa_kappa               DCLKTILHRIYGKFMVHR-----PFIRKAVSNIFYHFVFETDRHNGIAEL 271
gi|Oryza_sativa_N/A                 ECLKTVLHRIYGKFMGNR-----PFIRKAVSNIFYRFVFETDRHNGIAEL 252
gi|Arabidopsis_thaliana_alpha       EYLKTILHRIYGKFMVHR-----PFIRKAMNHIFYRFIYETERHSGIGEL 270
gi|Arabidopsis_thaliana_beta        EYLKTILHRIYGKFMVHR-----PFIRKAINNIFYRFIYETERHSGIGEL 270
gi|Oryza_sativa_N/A_2               EYLKTVLHRIYGKFMVHR-----PFIRKAINNVFYRFIFETERHNGIGEL 310
gi|Arabidopsis_thaliana_epsilo      EYLKTVLHRIYGKFIFHR-----PFIRCSIYNIFYKFLYETERCIGIGEL 279
gi|Chlamydomonas_reinhardtii_w      DYLKTILHRIYGKFMVHR-----PFIRKAINNVFYRFIFETERHNGVAEL 268
gi|Schizosaccharomyces_pombe_P      DFLKTTLHRIYGKFLSLR-----AFIRRSINNLFLQFVYENEQFNGIAEL 303
gi|Schizosaccharomyces_pombe_P      ELLKTTLHRIYGKFLNLR-----SYIRKSMNNVFLQFIYEREKFHGIAEL 404
gi|Ashbya_gossypii_RTS1             DCLKTTLHRIYGKFLSLR-----SFIRRSINNIFLQFVYETERFNGIAEL 366
gi|Saccharomyces_cerevisiae_RT      DCLKTTLHRIYGKFLSLR-----SFIRRSMNNIFLQFIYETEKFNGVAEL 473

gi|Aspergillus_niger_N/A            LEILGSIINGFALPLKEEHKLFLTRVLLPLHKVKSLSMYHPQLAYCIVQF 407
gi|Aspergillus_nidulans_parA        LEILGSIINGFALPLKEEHKLFLTRVLLPLHKVKSLSMYHPQLAYCIVQF 409
gi|Mus_musculus_alpha               LEILGSIINGFALPLKAEHKQFLMKVLIPMHTAKGLALFHAQLAYCVVQF 299
gi|Rattus_norvegicus_alpha          LEILGSIINGFALPLKAEHKQFLMKVLIPMHTAKGLALFHAQLAYCVVQF 299
gi|Macaca_mulatta_alpha             LEILGSIINGFALPLKAEHKQFLMKVLIPMHTAKGLALFHAQLAYCVVQF 299
gi|Homo_sapiens_alpha               LEILGSIINGFALPLKAEHKQFLMKVLIPMHTAKGLALFHAQLAYCVVQF 299
gi|Ovis_aries_alpha                 LEILGSIINGFALPLKAEHKQFLMKVLIPMHTAKGLALFHAQLAYCVVQF 302
gi|Bos_taurus_alpha                 LEILGSIINGFALPLKAEHKQFLMKVLIPMHTAKGLALFHAQLAYCVVQF 302
gi|Felis_catus_alpha                LEILGSIINGFALPLKAEHKQFLMKVLIPMHTAKGLALFHAQLAYCVVQF 215
gi|Canis_lupus_familiaris_alph      LEILGSIINGFALPLKAEHKQFLMKVLIPMHTAKGLALFHAQLAYCVVQF 215
gi|Falco_peregrinus_alpha           LEILGSIINGFALPLKAEHKQFLMKVLIPMHTAKGLALFHAQLAYCVVQF 289
gi|Gallus_gallus_alpha              LEILGSIINGFALPLKAEHKQFLMKVLIPMHTAKGLALFHAQLAYCVVQF 289
gi|Alligator_mississippiensis_      LEILGSIINGFALPLKAEHKQFLMKVLIPMHTAKGLALFHAQLAYCVVQF 299
gi|Chrysemys_picta_bellii_alph      LEILGSIINGFALPLKAEHKQFLMKVLIPMHTAKGLALFHAQLAYCVVQF 289
gi|Xenopus_laevis_alpha             LEILGSIINGFALPLKSEHKQFLMKVLIPMHTAKGLALFHAQLAYCVVQF 288
gi|Xenopus_tropicalis_alpha         LEILGSIINGFALPLKAEHKQFLMKVLIPMHTAKGLALFHAQLAYCVVQF 288
gi|Ambystoma_mexicanum_alpha        LEILGSIINGFALPLKAEHKQFLMKVLIPMHTAKGLALFHAQLAYCVVQF 215
gi|Danio_rerio_alpha                LEILGSIINGFALPLKAEHKQFLMKILIPLHTAKPLALFHAQLAYCVVQF 291
gi|Petromyzon_marinus_S4RHV1        LEILGSIIDGFALPLKTEHKQFLVRVLLPLHTARGLSIFHAQLAYCVMLF 289
gi|Mus_musculus_epsilon             LEILGSIINGFALPLKAEHKQFLVKVLIPLHTVRSLSLFHAQLAYCIVQF 291
gi|Rattus_norvegicus_epsilon        LEILGSIINGFALPLKAEHKQFLVKVLIPLHTVRSLSLFHAQLAYCIVQF 291
gi|Macaca_mulatta_epsilon           LEILGSIINGFALPLKAEHKQFLVKVLIPLHTVRSLSLFHAQLAYCIVQF 291
gi|Homo_sapiens_epsilon             LEILGSIINGFALPLKAEHKQFLVKVLIPLHTVRSLSLFHAQLAYCIVQF 291
gi|Ovis_arie_epsilon                LEILGSIINGFALPLKAEHKQFLVKVLIPLHTVRSLSLFHAQLAYCIVQF 291
gi|Bos_taurus_epsilon               LEILGSIINGFALPLKAEHKQFLVKVLIPLHTVRSLSLFHAQLAYCIVQF 291
gi|Canis_lupus_familiaris_epsi      LEILGSIINGFALPLKAEHKQFLVKVLIPLHTVRSLSLFHAQLAYCIVQF 291
gi|Falco_peregrinus_epsilon         LEILGSIINGFALPLKAEHKQFLVKVLIPLHTVRSLSLFHAQLAYCIVQF 291
gi|Gallus_gallus_epsilon            LEILGSIINGFALPLKAEHKQFLVKVLIPLHTVRSLSLFHAQLAYCIVQF 291
gi|Felis_catus_epsilon              LEILGSIINGFALPLKAEHKQFLVKVLIPLHTVRSLSLFHAQLAYCIVQF 291
gi|Chrysemys_picta_bellii_epsi      LEILGSIINGFALPLKAEHKQFLVKVLIPLHTVRSLSLFHAQLAYCIVQF 291
gi|Xenopus_laevis_epsilon           LEILGSIINGFALPLKAEHKQFLVKVLIPLHTVRSLSLFHAQLAYCIVQF 291
gi|Xenopus_tropicalis_epsilon       LEILGSIINGFALPLKAEHKQFLVKVLIPLHTVRSLSLFHAQLAYCIVQF 291
gi|Alligator_mississippiensis_      LEILGSIINGFALPLKAEHKQFLVKVLIPLHTVRSLSLFHAQLAYCIVQF 280
gi|Ambystoma_mexicanum_epsilon      LEILGSIINGFALPLKAEHKQFLVKVLIPLHTVRSLSLFHAQLAYCIVQF 287
gi|Danio_rerio_epsilon              LEILGSIINGFALPLKAEHKQFLVKVLLPLHTVRSLSLFHAQLAYCIVQF 291
gi|Mus_musculus_beta                LEILGSIINGFALPLKTEHKQFLVRVLIPLHSVKSLSVFHAQLAYCVVQF 305
```

Figure S1. *Cont.*

```
gi|Rattus_norvegicus_beta          LEILGSIINGFALPLKTEHKQFLVRVLIPLHSVKSLSVFHAQLAYCVVQF 305
gi|Felis_catus_beta                LEILGSIINGFALPLKTEHKQFLVRVLIPLHSVKSLSVFHAQLAYCVVQF 305
gi|Canis_lupus_familiaris_beta     LEILGSIINGFALPLKTEHKQFLVRVLIPLHSVKSLSVFHAQLAYCVVQF 305
gi|Homo_sapiens_beta               LEILGSIINGFALPLKTEHKQFLVRVLIPLHSVKSLSVFHAQLAYCVVQF 305
gi|Bos_taurus_beta                 LEILGSIINGFALPLKTEHKQFLVRVLIPLHSVKSLSVFHAQLAYCVVQF 305
gi|Ovis_aries_beta                 LEILGSIINGFALPLKTEHKQFLVRVLIPLHSVKSLSVFHAQLAYCVVQF 305
gi|Macaca_mulatta_beta             LEILGSIINGFALPLKTEHKQFLVRVLIPLHSVKSLSVFHAQLAYCVVQF 269
gi|Xenopus_laevis_beta             --------------------------------------------------
gi|Alligator_mississippiensis_     LEILGSIINGFALPLXAEHKQFLVRVLIPLHSVKALSVFHAQLAYCVVQF 305
gi|Chrysemys_picta_bellii_beta     LEILGSIINGFALPLKTEHKHFLVRVLIPLHSVKSLSIFHAQLAYCVVQF 305
gi|Xenopus_tropicalis_beta         LEILGSIINGFALPLKSEHKQFLVRVLIPLHSAKSLSIFHAQLAYCAVQF 303
gi|Danio_rerio_beta                LEILGSIINGFALPLKSEHKQFLVRVLIPLHTAKSLSIFHAQLAYCVVQF 304
gi|Ambystoma_mexicanum_beta        LEILGSIINGFALPLKAEHKQFLVRVLLPLHSVKSLSIFHAQLAYCVVQF 174
gi|Petromyzon_marinus_S4RGA7       LEILGSIINGFAMPLKSEHKQFLVKVLLPLHTVKVLSLFHAQLAYCVVQF 68
gi|Strongylocentrotus_purpurat     LEILGSIINGFALPLKTEHKQFLIKVLIPLHKVKCLGLYHAQLAYCVVQF 287
gi|Drosophila_melanogaster_wdb     LEILGSIINGFALPLKAEHKQFLVKVLLPLHKVKCLSLYHAQLAYCIVQF 286
jgi|Branchiostoma_floridae_237     LEILGSIINGFALPLKAEHKQFLIKVLIPLHKAKSLSLYHAQLAYCVVQF 242
gi|Hydra_vulgaris_alpha            LEILGSIINGFALPLKAEHKQFLLKVLIPLHKARCLSLYHAQLAYCVVQF 295
jgi|Branchiostoma_floridae_112     --------------------------------------------------
jgi|Branchiostoma_floridae_284     LEILGSIINGFALPLKAEHKQFLLKVLIPLHKAKSLAQYHAQLAYCVVQF 255
gi|Caenorhabditis_elegans_PPTR     LEILGSIINGFALPLKQEHKVFLVKVLLPLHKPKCLSLYHAQLAYCVVQF 316
gi|Amphimedon_queenslandica_be     LEILGSIINGFALPLKVEHKQFLIRVLLPLHKAKSLAQYQAQLAYCVVQF 309
gi|Chrysemys_picta_bellii_gamm     LEILGSIINGFALPLKEEHKIFLLKVLLPLHKVKSLSVYHPQLAYCVVQF 274
gi|Chrysemys_picta_bellii_delt     LEILGSIINGFALPLKEEHKIFLLKVLLPLHKVKSLSVYHPQLAYCVVQF 199
gi|Falco_peregrinus_gamma          LEILGSIINGFALPLKEEHKIFLLKVLLPLHKVKSLSVYHPQLAYCVVQF 274
gi|Falco_peregrinus_delta/gamm     LEILGSIINGFALPLKEEHKIFLLKVLLPLHKVKSLSVYHPQLAYCVVQF 199
gi|Gallus_gallus_gamma             LEILGSIINGFALPLKEEHKIFLLKVLLPLHKVKSLSVYHPQLAYCVVQF 274
gi|Alligator_mississippiensis_     LEILGSIINGFALPLKEEHKIFLLKVLLPLHKVKSLSVYHPQLAYCVVQF 274
gi|Alligator_mississippiensis_     LEILGSIINGFALPLKEEHKIFLLKVLLPLHKVKSLSVYHPQLAYCVVQF 199
gi|Ambystoma_mexicanum_gamma       LEILGSIINGFALPLKEEHKIFLLKVLLPLHKVKSLSVYHPQLAYCVVQF 275
gi|Mus_musculus_gamma              LEILGSIINGFALPLKEEHKIFLLKVLLPLHKVKSLSVYHPQLAYCVVQF 274
gi|Rattus_norvegicus_gamma         LEILGSIINGFALPLKEEHKIFLLKVLLPLHKVKSLSVYHPQLAYCVVQF 274
gi|Ovis_aries_gamma                LEILGSIINGFALPLKEEHKIFLLKVLLPLHKVKSLSVYHPQLAYCVVQF 274
gi|Bos_taurus_gamma                LEILGSIINGFALPLKEEHKIFLLKVLLPLHKVKSLSVYHPQLAYCVVQF 274
gi|Canis_lupus_familiaris_gamm     LEILGSIINGFALPLKEEHKIFLLKVLLPLHKVKSLSVYHPQLAYCVVQF 274
gi|Macaca_mulatta_gamma            LEILGSIINGFALPLKEEHKIFLLKVLLPLHKVKSLSVYHPQLAYCVVQF 274
gi|Homo_sapiens_gamma              LEILGSIINGFALPLKEEHKIFLLKVLLPLHKVKSLSVYHPQLAYCVVQF 264
gi|Felis_catus_gamma               LEILGSIINGFALPLKEEHKIFLLKVLLPLHKVKSLSVYHPQLAYCVVQF 321
gi|Canis_lupus_familiaris_delt     LEILGSIINGFALPLKEEHKIFLLKVLLPLHKVKSLSVYHPQLAYCVVQF 321
gi|Macaca_mulatta_delta/gamma      LEILGSIINGFALPLKEEHKIFLLKVLLPLHKVKSLSVYHPQLAYCVVQF 556
gi|Bos_taurus_delta/gamma          LEILGSIINGFALPLKEEHKIFLLKVLLPLHKVKSLSVYHPQLAYCVVQF 328
gi|Mus_musculus_delta/gamma        LEILGSIINGFALPLKEEHKIFLLKVLLPLHKVKSLSVYHPQLAYCVVQF 331
gi|Rattus_norvegicus_delta/gam     LEILGSIINGFALPLKEEHKIFLLKVLLPLHKVKSLSVYHPQLAYCVVQF 397
gi|Danio_rerio_gamma               LEILGSIINGFALPLKEEHKIFLLKVLLPLHKVKSLSVYHPQLAYCVVQF 274
gi|Danio_rerio_delta/gamma         LEILGSIINGFALPLKEEHKIFLLKVLLPLHKVKSLSVYHPQLAYCVVQF 327
gi|Xenopus_laevis_delta/gamma      LEILGSIINGFALPLKEEHKIFLLKVLLPLHKVKSLSVYHPQLAYCVVQF 327
gi|Xenopus_laevis_gamma            LEILGSIINGFALPLKEEHKIFLLKVLLPLHKVKSLSVYHPQLAYCVVQF 327
gi|Xenopus_tropicalis_delta/ga     LEILGSIINGFALPLKEEHKIFLLKVLLPLHKVKSLSVYHPQLAYCVVQF 327
gi|Mus_musculus_delta              LEILGSIINGFALPLKEEHKVFLVRVLLPLHKVKSLSVYHPQLAYCVVQF 342
gi|Rattus_norvegicus_delta         LEILGSIINGFALPLKEEHKMFLIRVLLPLHKVKSLSVYHPQLAYCVVQF 370
gi|Felis_catus_delta               LEILGSIINGFALPLKEEHKMFLIRVLLPLHKVKSLSVYHPQLAYCVVQF 356
gi|Bos_taurus_delta                LEILGSIINGFALPLKEEHKMFLIRVLLPLHKVKSLSVYHPQLAYCVVQF 348
gi|Canis_lupus_familiaris_delt     LEILGSIINGFALPLKEEHKMFLIRVLLPLHKVKSLSVYHPQLAYCVVQF 343
gi|Macaca_mulatta_delta            LEILGSIINGFALPLKEEHKMFLIRVLLPLHKVKSLSVYHPQLAYCVVQF 359
gi|Homo_sapiens_delta              LEILGSIINGFALPLKEEHKMFLIRVLLPLHKVKSLSVYHPQLAYCVVQF 350
gi|Ovis_aries_delta                LEILGSIINGFALPLKEEHKMFLIRVLLPLHKVKSLSVYHPQLAYCVVQF 347
gi|Falco_peregrinus_delta          LEILGSIINGFALPLKEEHKMFLIRVLLPLHKVKSLSVYHPQLAYCVVQF 250
gi|Gallus_gallus_delta             LEILGSIINGFALPLKEEHKMFLIRVLLPLHKVKSLSVYHPQLAYCVVQF 345
gi|Alligator_mississippiensis_     LEILGSIINGFALPLKEEHKMFLIRVLLPLHKVKSLSVYHPQLAYCVVQF 344
gi|Chrysemys_picta_bellii_delt     LEILGSIINGFALPLKEEHKMFLIRVLLPLHKVKSLSVYHPQLAYCVVQF 346
gi|Danio_rerio_delta               LEILGSIINGFALPLKEEHKMFLIRVLLPLHKVKSLSVYHPQLAYCVVQF 337
```

Figure S1. *Cont.*

```
gi|Ambystoma_mexicanum_delta          LEILGSIINGFALPLKEEHKMFLIRVLLPLHKVKSLSVYHPQLAYCVVQF 199
gi|Petromyzon_marinus_S4RN43          LEILGSIINGFALPLKEEHKVFLLKVLLPLHKVKSLSVYHPQLAYCVVQF 327
jgi|Branchiostoma_floridae_237        LEVLGSIINGFALPLKEEHKVFLLKVLLPLHKVKSLSVYHPQLAYCVVQF 247
jgi|Branchiostoma_floridae_252        LEVLGSIINGFALPLKEEHKVFLLKVLLPLHKVKSLSVYHPQLAYCVVQF 300
gi|Strongylocentrotus_purpurat        LEILGSIINGFALPLKEEHKTFLLKVLMPLHKAKSLSVYHPQLAYCVVQF 308
gi|Caenorhabditis_elegans_PPTR        LEILGSIINGFALPLKEEHKTFLLRVLLPLHKVKSLSVYHPQLAYCVVQF 295
gi|Hydra_vulgaris_delta               LEVLGSIINGFALPLKEEHKMFLLKVLLPLHKVKALSMHHPQLAYCVVQF 317
gi|Amphimedon_queenslandica_de        LEILGSIINGFALPLKEEHKMFLLRVLIPLHKVKSLSCYHPQLAYCVVQF 358
gi|Drosophila_melanogaster_B56        LEILGSIINGFALPLKEEHKQFLLKVLLPLHKAKSLSVYHPQLTYCVVQF 431
gi|Dictyostelium_discoideum_ps        LEVLGSIINGFLVPLKDEHKQFLIKVLIPLHKPKSYSVYCSHLGYCMSQF 356
gi|Dictyostelium_purpureum_N/A        LEVLGSIINGFVVPLKDEHKQFLIKVLIPLHKPKSYSVYCSHLGYCMTQF 313
gi|Dictyostelium_fasciculatum_        LEVLGSIINGFAVPLKDEHKQFLTKVLLPLHKPKSYSIYCSQLGYCISQF 326
gi|Polysphondylium_pallidum_N/        LEVLGSIINGFAVPLKDEHKQFLVKVLLPLHKPKSYSVYCSHLGYCITQF 304
gi|Arabidopsis_thaliana_theta         LEILGSIINGFALPLKDEHKVFLVRALVPLHKPKSLQMYHQQLSYCITQF 321
gi|Arabidopsis_thaliana_eta           LEILGSIINGFALPLKDEHK-----------------------------F 313
gi|Arabidopsis_thaliana_zeta          LEILGSIINGFALPLKEEHKLFLLRALIPLHKPKCSSVYHQQLSYCIVQF 351
gi|Arabidopsis_thaliana_gamma         LEILGSIINGFALPLKEEHKLFLIRALIPLHRPKCASAYHQQLSYCIVQF 330
gi|Oryza                              LEILGSIINGFALPLKEEHKLFLVRTLIPLHKPKCIALYHQQLSYSITQF 331
gi|Oryza_sativa_zeta                  LEILGSIINGFALPLKEEHKLFLVRALIPLHKPKCIGMYHQQLSYCITQF 331
gi|Oryza_sativa_theta                 LEILGSIINGFALPLKEEHKLFLVRALIPLHKPKCVSMYHQQLSYCVTQF 335
gi|Oryza_sativa_eta                   LEILGSIINGFALPLKEEHKLFLVRALIPLHKPKCVSMYHQQLSYCITQF 324
gi|Arabidopsis_thaliana_delta         LEVLGSIINGFALPLKEEHKLFLTRVLIPLHKLKCLPNYHQQLSYCVIQF 309
gi|Arabidopsis_thaliana_N/A           LEIFGSIVSGFALPLKEEHKIFLWRVLIPLHKPKSVGNYFQQLSYCITQF 315
gi|Oryza_sativa_kappa                 LEVFGSVISGFALPLKEEHKIFLWRVLVPLHKPKSVGVYLQQLTYCVTQF 321
gi|Oryza_sativa_N/A                   LEVFGSVISGFAKPLKEEHKLFLWKALVPLHKPKTVGVYLPQLTYCIIQF 302
gi|Arabidopsis_thaliana_alpha         LEILGSIINGFALPMKEEHKLFLIRALIPLHKPKPIAMYHQQLSYCIVQF 320
gi|Arabidopsis_thaliana_beta          LEILGSIINGFALPMKEEHKLFLIRVLIPLHKPKPIVVYHQQLSYCIVQF 320
gi|Oryza_sativa_N/A_2                 LEILGSIINGFALPMKEEHKLFLSRALIPLHKPKSVAIYHQQLSYCIVQF 360
gi|Arabidopsis_thaliana_epsilo        LEILGSVINGFTVPMREEHRLYLVKAILPLHKSKGISIYHQQLAYCVTQF 329
gi|Chlamydomonas_reinhardtii_w        LEILGSIINGFALPLKEEHKLFLQRALMPLHKPKCVAMYHQQLAYCVTQF 318
gi|Schizosaccharomyces_pombe_P        LEILGSIINGFALPLKEEHKIFLSRVLIPLHKAKSLPLYYPQIAYGIVQF 353
gi|Schizosaccharomyces_pombe_P        LEILGSIINGFAVPLKEEHKIFLSKVLIPLHQTKSVFLYHPQLTYCIVQF 454
gi|Ashbya_gossypii_RTS1               LEILGSIINGFALPLKEEHKVFLVRILIPLHKVRCLSLYHPQLAYCIVQF 416
gi|Saccharomyces_cerevisiae_RT        LEILGSIINGFALPLKEEHKVFLVRILIPLHKVRCLSLYHPQLAYCIVQF 523


gi|Aspergillus_niger_N/A              LEKDSSLTEDVVLGLLRYWPKTNSTKEVMYLNEVEDIFEVMDPAE-FAKV 456
gi|Aspergillus_nidulans_parA          LEKDSTLTEDVVLGLLRYWPKTNSTKEVMFLNEVEDIFEVMDPAE-FAKV 458
gi|Mus_musculus_alpha                 LEKDTTLTEPVIRGLLKFWPKTCSQKEVMFLGEIEEILDVIEPTQ-FKKI 348
gi|Rattus_norvegicus_alpha            LEKDTTLTEPVIRGLLKFWPKTCSQKEVMFLGEIEEILDVIEPTQ-FKKI 348
gi|Macaca_mulatta_alpha               LEKDTTLTEPVIRGLLKFWPKTCSQKEVMFLGEIEEILDVIEPTQ-FKKI 348
gi|Homo_sapiens_alpha                 LEKDTTLTEPVIRGLLKFWPKTCSQKEVMFLGEIEEILDVIEPTQ-FKKI 348
gi|Ovis_aries_alpha                   LEKDTTLTEPVIRGLLKFWPKTCSQKEVMFLGEIEEILDVIEPTQ-FKKI 351
gi|Bos_taurus_alpha                   LEKDTTLTEPVIRGLLKFWPKTCSQKEVMFLGEIEEILDVIEPTQ-FKKI 351
gi|Felis_catus_alpha                  LEKDTTLTEPVIRGLLKFWPKTCSQKEVMFLGEIEEILDVIEPTQ-FKKI 264
gi|Canis_lupus_familiaris_alph        LEKDTTLTEPVIRGLLKFWPKTCSQKEVMFLGEIEEILDVIEPTQ-FKKI 264
gi|Falco_peregrinus_alpha             LEKDTTLTEPVIRGLLKFWPKTCSQKEVMFLGEIEEILDVIEPTQ-FKKI 338
gi|Gallus_gallus_alpha                LEKDTTLTEPVIRGLLKFWPKTCSQKEVMFLGEIEEILDVIEPTQ-FKKI 338
gi|Alligator_mississippiensis_        LEKETTLTEPVIRGLLKFWPKTCSQKEVMFLGEIEEILDVIEPTQ-FKKI 348
gi|Chrysemys_picta_bellii_alph        LEKDTTLTEPVIRGLLKFWPKTCSQKEVMFLGEIEEILDVIEPTQ-FKKI 338
gi|Xenopus_laevis_alpha               MEKDTTLTEPVIRGLLKFWPKTCSQKEVMFLGEIEEILDVIEPTQ-FKKI 337
gi|Xenopus_tropicalis_alpha           LEKDTTLTDPVIRGLLKFWPKTCSQKEVMYLGEIEEILDVIEPTQ-FKKI 337
gi|Ambystoma_mexicanum_alpha          LEKDTTLTEPVIRGLLKFWPKTCSQKEVMYLGEIEEILDVIEPTQ-FKKI 264
gi|Danio_rerio_alpha                  LEKDPTLTEVVVRGLLKFWPKTCSQKEVMFLGEIEEILDVIEPTQ-FKKI 340
gi|Petromyzon_marinus_S4RHV1          LEKDATLAEIVVRGLLKYWPKTCSQKEVMFLGEVEEVLDVIEPTQ-FVKI 338
gi|Mus_musculus_epsilon               LEKDPSLTEPVIRGLMKFWPKTCSQKEVMFLGELEEILDVIEPSQ-FVKI 340
gi|Rattus_norvegicus_epsilon          LEKDPSLTEPVIRGLMKFWPKTCSQKEVMFLGELEEILDVIEPSQ-FVKI 340
gi|Macaca_mulatta_epsilon             LEKDPSLTEPVIRGLMKFWPKTCSQKEVMFLGELEEILDVIEPSQ-FVKI 340
gi|Homo_sapiens_epsilon               LEKDPSLTEPVIRGLMKFWPKTCSQKEVMFLGELEEILDVIEPSQ-FVKI 340
gi|Ovis_arie_epsilon                  LEKDPSLTEPVIRGLMKFWPKTCSQKEVMFLGELEEILDVIEPSQ-FVKI 340
gi|Bos_taurus_epsilon                 LEKDPSLTEPVIRGLMKFWPKTCSQKEVMFLGELEEILDVIEPSQ-FVKI 340
```

Figure S1. *Cont.*

```
gi|Canis_lupus_familiaris_epsi      LEKDPSLTEPVIRGLMKFWPKTCSQKEVMFLGELEEILDVIEPSQ-FVKI 340
gi|Falco_peregrinus_epsilon         LEKDPSLTEPVIRGLMKFWPKTCSQKEVMFLGELEEILDVIEPSQ-FVKI 340
gi|Gallus_gallus_epsilon            LEKDPSLTEPVIRGLMKFWPKTCSQKEVMFLGELEEILDVIEPSQ-FVKI 340
gi|Felis_catus_epsilon              LEKDPSLTEPVIRGLMKFWPKTCSQKEVMFLGELEEILDVIEPSQ-FVKI 340
gi|Chrysemys_picta_bellii_epsi      LEKDPSLTEPVIRGLMKFWPKTCSQKEVMFLGELEEILDVIEPSQ-FVKI 340
gi|Xenopus_laevis_epsilon           LEKDPTLTEPVIRGLMKFWPKTCSQKEVMFLGELEEILDVIEPSQ-FVKI 340
gi|Xenopus_tropicalis_epsilon       LEKDPTLTEPVIRGLMKFWPKTCSQKEVMFLGELEEILDVIEPSQ-FVKI 340
gi|Alligator_mississippiensis_      LEKDPSLTEPVIRGLMKFWPKTCSQKEVMFLGELEEILDVIEPSQ-FVKI 329
gi|Ambystoma_mexicanum_epsilon      LEKDPSLTEPVIRGLMKFWPKTCSQKEVMFLGELEEILDVIEPTQ-FVKI 336
gi|Danio_rerio_epsilon              LEKDPTLTEPVIRGLLKFWPKTCSQKEVMFLGELEEILDVIEPTQ-FVKI 340
gi|Mus_musculus_beta                LEKDATLTEHVIRGLLKYWPKTCTQKEVMFLGEMEEILDVIEPSQ-FVKI 354
gi|Rattus_norvegicus_beta           LEKDATLTEHVIRGLLKYWPKTCTQKEVMFLGEMEEILDVIEPSQ-FVKI 354
gi|Felis_catus_beta                 LEKDATLTEHVIRGLLKYWPKTCTQKEVMFLGEMEEILDVIEPSQ-FVKI 354
gi|Canis_lupus_familiaris_beta      LEKDATLTEHVIRGLLKYWPKTCTQKEVMFLGEMEEILDVIEPSQ-FVKI 354
gi|Homo_sapiens_beta                LEKDATLTEHVIRGLLKYWPKTCTQKEVMFLGEMEEILDVIEPSQ-FVKI 354
gi|Bos_taurus_beta                  LEKDATLTEHVIRGLLKYWPKTCTQKEVMFLGEMEEILDVIEPSQ-FVKI 354
gi|Ovis_aries_beta                  LEKDATLTEHVIRGLLKYWPKTCTQKEVMFLGEMEEILDVIEPSQ-FVKI 354
gi|Macaca_mulatta_beta              LEKDATLTEHVIRGLLKYWPKTCTQKEVMFLGEMEEILDVIEPSQ-FVKI 318
gi|Xenopus_laevis_beta              --------------------------------------------------
gi|Alligator_mississippiensis_      LEKDATLTEHVIRGLLKYWPKTCTQKEVMFLGEIEEILDVIEPSQ-FVKV 354
gi|Chrysemys_picta_bellii_beta      LEKDATLTEHVIRGLLKYWPKTCTQKEVMFLGEIEEILDVIEPSQ-FVKV 354
gi|Xenopus_tropicalis_beta          LEKDSSLTEHVIRGLLKYWPKTCTQKEVMFLGELEEILDVIEPSQ-FVKI 352
gi|Danio_rerio_beta                 MEKDATVTEHIIRGLLRYWPKTCTQKEVMFLGEIEEILDVIEPSQ-FIRV 353
gi|Ambystoma_mexicanum_beta         LEKDATLTEHVIRGLLKYWPKTCTQKEVMFLGEIEEILDVIEPSQ-FIKI 223
gi|Petromyzon_marinus_S4RGA7        LEKDPQLTEPVIRALLKFWPKTCSQKEVMFLGELEEILDVIESNQ-FVKI 117
gi|Strongylocentrotus_purpurat      LEKDAALTEQVVGGLLKFWPKTCSAKEVMFLGEIEEILDVIEPSQ-FVKI 336
gi|Drosophila_melanogaster_wdb      LEKDPFLTEPVVRGLLKFWPKTCSQKEVMFLGEIEEILDVIDPPQ-FVKI 335
jgi|Branchiostoma_floridae_237      LEKDATLTEPVVRGLLKFWPKTCSQKEVMFLGEIEEILDVIEPSQ-FVRI 291
gi|Hydra_vulgaris_alpha             LEKDSTLTEPVIMGLLKYWPKTCSQKEVMFLGEIEEILDAIEASQ-FQKV 344
jgi|Branchiostoma_floridae_112      --------------------------------------------------
jgi|Branchiostoma_floridae_284      LEKDATLTEPVVRGLLKFWPKTCSQKEVMFLGEIEEILDVIEPSQ-FVRI 304
gi|Caenorhabditis_elegans_PPTR      IEKDSSLTPQVFEALLKFWPRTCSSKEVMFLGEVEEILDIIEPEQ-FKKI 365
gi|Amphimedon_queenslandica_be      LEKDAALTEQVLHGLFKFWPKTNSSKEVMYLGEIEEILDIIDAPQ-FVTI 358
gi|Chrysemys_picta_bellii_gamm      LEKDSTLTEPVVMALLKYWPKTHSPKEVMFLNELEEILDVIEPSE-FVKV 323
gi|Chrysemys_picta_bellii_delt      LEKDSTLTEPVVMALLKYWPKTHSPKEVMFLNELEEILDVIEPSE-FVKV 248
gi|Falco_peregrinus_gamma           LEKDSTLTEPVVMALLKYWPKTHSPKEVMFLNELEEILDVIEPSE-FVKV 323
gi|Falco_peregrinus_delta/gamm      LEKDSTLTEPVVMALLKYWPKTHSPKEVMFLNELEEILDVIEPSE-FVKV 248
gi|Gallus_gallus_gamma              LEKDSTLTEPVVMALLKYWPKTHSPKEVMFLNELEEILDVIEPSE-FVKV 323
gi|Alligator_mississippiensis_      LEKDSTLTEPVVMALLKYWPKTHSPKEVMFLNELEEILDVIEPSE-FVKV 323
gi|Alligator_mississippiensis_      LEKDSTLTEPVVMALLKYWPKTHSPKEVMFLNELEEILDVIEPSE-FVKV 248
gi|Ambystoma_mexicanum_gamma        LEKDSTLTEPVVMALLKYWPKTHSPKEVMFLNELEEILDVIEPSE-FVKV 324
gi|Mus_musculus_gamma               LEKDSTLTEPVVMALLKYWPKTHSPKEVMFLNELEEILDVIEPSE-FVKI 323
gi|Rattus_norvegicus_gamma          LEKDSTLTEPVVMALLKYWPKTHSPKEVMFLNELEEILDVIEPSE-FVKI 323
gi|Ovis_aries_gamma                 LEKDSTLTEPVVMALLKYWPKTHSPKEVMFLNELEEILDVIEPSE-FVKI 323
gi|Bos_taurus_gamma                 LEKDSTLTEPVVMALLKYWPKTHSPKEVMFLNELEEILDVIEPSE-FVKI 323
gi|Canis_lupus_familiaris_gamm      LEKDSTLTEPVVMALLKYWPKTHSPKEVMFLNELEEILDVIEPSE-FVKI 323
gi|Macaca_mulatta_gamma             LEKDSTLTEPVVMALLKYWPKTHSPKEVMFLNELEEILDVIEPSE-FVKI 323
gi|Homo_sapiens_gamma               LEKDSTLTEPVVMALLKYWPKTHSPKEVMFLNELEEILDVIEPSE-FVKI 313
gi|Felis_catus_gamma                LEKDSTLTEPVVMALLKYWPKTHSPKEVMFLNELEEILDVIEPSE-FVKV 370
gi|Canis_lupus_familiaris_delt      LEKDSTLTEPVVMALLKYWPKTHSPKEVMFLNELEEILDVIEPSE-FVKI 370
gi|Macaca_mulatta_delta/gamma       LEKDSTLTEPVVMALLKYWPKTHSPKEVMFLNELEEILDVIEPSE-FVKI 605
gi|Bos_taurus_delta/gamma           LEKDSTLTEPVVMALLKYWPKTHSPKEVMFLNELEEILDVIEPSE-FVKI 377
gi|Mus_musculus_delta/gamma         LEKDSTLTEPVVMALLKYWPKTHSPKEVMFLNELEEILDVIEPSE-FVKI 380
gi|Rattus_norvegicus_delta/gam      LEKDSTLTEPVVMALLKYWPKTHSPKEVMFLNELEEILDVIEPSE-FVKI 446
gi|Danio_rerio_gamma                LEKDSTLTEPVVMALLKYWPKTHSPKEVMFLNELEEILDVIEPSE-FVKV 323
gi|Danio_rerio_delta/gamma          LEKDSTLTEPVVMALLKYWPKTHSPKEVMFLNELEEILDVIEPSE-FVKV 376
gi|Xenopus_laevis_delta/gamma       LEKDSTLTEPVVMALLKYWPKTHSPKEVMFLNELEEILDVIEPSE-FVKV 376
gi|Xenopus_laevis_gamma             LEKDSTLTEPVVMALLKYWPKTHSPKEVMFLNELEEILDVIEPSE-FVKV 376
gi|Xenopus_tropicalis_delta/ga      LEKDSTLTEPVVMALLKYWPKTHSPKEVMFLNELEEILDVIEPSE-FVKV 376
gi|Mus_musculus_delta               LEKESSLTEPVIVGLLKFWPKTHSPKEVMFLNELEEILDVIEPSE-FSKV 391
gi|Rattus_norvegicus_delta          LEKESSLTEPVIVGLLKFWPKTHSPKEVMFLNELEEILDVIEPSE-FSKV 419
```

Figure S1. *Cont.*

```
gi|Felis_catus_delta                LEKESSLTEPVIVGLLKFWPKTHSPKEVMFLNELEEILDVIEPSE-FSKV 405
gi|Bos_taurus_delta                 LEKESSLTEPVIVGLLKFWPKTHSPKEVMFLNELEEILDVIEPSE-FSKV 397
gi|Canis_lupus_familiaris_delt      LEKESSLTEPVIVGLLKFWPKTHSPKEVMFLNELEEILDVIEPSE-FSKV 392
gi|Macaca_mulatta_delta             LEKESSLTEPVIVGLLKFWPKTHSPKEVMFLNELEEILDVIEPSE-FSKV 408
gi|Homo_sapiens_delta               LEKESSLTEPVIVGLLKFWPKTHSPKEVMFLNELEEILDVIEPSE-FSKV 399
gi|Ovis_aries_delta                 LEKESSLTEPVIVGLLKFWPKTHSPKEVMFLNELEEILDVIEPSE-FSKV 396
gi|Falco_peregrinus_delta           LEKDSSLTEPVIVGLLKFWPKTHSPKEVMFLNELEEILDVIEPSE-FVKV 299
gi|Gallus_gallus_delta              LEKDSSLTEPVIVGLLKFWPKTHSPKEVMFLNELEEILDVIEPSE-FVKV 394
gi|Alligator_mississippiensis_      LEKDSSLTEPVIMGLLKFWPKTHSPKEVMFLNELEEILDVIEPSE-FVKV 393
gi|Chrysemys_picta_bellii_delt      LEKESSLTEPVIVGLLKFWPKTHSPKEVMFLNELEEILDVIEPSE-FVKV 395
gi|Danio_rerio_delta                LEKDSSLTEPVIMGLLKFWPKTHSPKEVMFLNELEEILDVIEPSE-FVKV 386
gi|Ambystoma_mexicanum_delta        LEKDSSLTEPVIVGLLKFWPKTHSPKEVMFLNELEEILDVIEPSE-FVKV 248
gi|Petromyzon_marinus_S4RN43        LEKDSTLTEPVVMGLLKYWPKTHSPKEVMFLNELEEILDVIEPSE-FVKV 376
jgi|Branchiostoma_floridae_237      LEKDASLTEPVIMGLLKFWPKVHSPKEVMFLNELEEILDVIEPSE-FQKV 296
jgi|Branchiostoma_floridae_252      LEKDASLTEPVIMGLLKFWPKVHSPKEVS--NKLGNLLVIHRRS------ 342
gi|Strongylocentrotus_purpurat      LEKDPSLTEQVLRLLLKFWPKVHSPKEVMFLNELEEILDVIEPLE-FQKV 357
gi|Caenorhabditis_elegans_PPTR      IEKDSSLTEPVISGMLRFWPKQHSPKEVMFLNELEEVLDVIEPNE-FQKI 344
gi|Hydra_vulgaris_delta             LEKDPTLTEPCIMGLIKFWPKVHSTKEVMFLNELEEILDVIEPNE-FVKI 366
gi|Amphimedon_queenslandica_de      LEKDPSLTEPVVTGLLKFWPKVHSPKEVMFLNELEEILDVMEPVE-FVKV 407
gi|Drosophila_melanogaster_B56      LEKDPSLSEAVIKSLLKFWPKTHSPKEVMFLNELEELLDVIEPAE-FQKV 480
gi|Dictyostelium_discoideum_ps      IEKEPSLAEPIFKSILRLWPCGNSQKEVLFLSEMEDLLGLVSDEQ-FAKF 405
gi|Dictyostelium_purpureum_N/A      IEKDPSLAEPIFKSILRLWPCGNSQKEVLFLSEMEDLLSSVSEDQ-FLKF 362
gi|Dictyostelium_fasciculatum_      LEKDPMLALTIFKSILRQWPIGNSQKEVLFLYEIEDLLGSIRDEQQFTEI 376
gi|Polysphondylium_pallidum_N/      IEKDPTLSDVVFKTVLRMWPVGNSQKEVLFLYEIEDLLNSIGEEQ-FMRI 353
gi|Arabidopsis_thaliana_theta       VEKDCKLADTVIRGLLKSWPVTNSSKEVMFLNELEEVLEATQPPE-FQRC 370
gi|Arabidopsis_thaliana_eta         VEKDCKLADTVIRGLLKYWPVTNSSKEVMFLNELEEVLEATQPPE-FQRC 362
gi|Arabidopsis_thaliana_zeta        VEKDFKLADTVIRGLLKYWPVTNSSKEVMFLGELEEVLEATQAAE-FQRC 400
gi|Arabidopsis_thaliana_gamma       VEKDFKLADTVIRGLLKYWPVTNSSKEVMFLGELEEVLEATQAAE-FQRC 379
gi|Oryza                            VEKDCKLADTVIRGLIKYWPITNSTKEVMFLGELEEILDATQPAE-FQKC 380
gi|Oryza_sativa_zeta                VEKDCKLADTVIRGLLKYWPITNSSKEVLFLGELEEILEATQPAE-FQKC 380
gi|Oryza_sativa_theta               VEKDCKLADTVIRGLLKYWPITNSAKEVMFLGELEEVLEATQPAE-FQRC 384
gi|Oryza_sativa_eta                 VEKDCKLADTVIRGLLKYWPVTNSSKEVMFLGELEEVLEATQPAE-FQRC 373
gi|Arabidopsis_thaliana_delta       VEKDCKLADTVIRGMLKYWPVTNSAKEIMFLNELEEILEATQLTE-FERC 358
gi|Arabidopsis_thaliana_N/A         IDKEPKLGSVVIKGLLKFWPITNSQKEVMFLGEVEEIVEAMSVME-FQKI 364
gi|Oryza_sativa_kappa               IEKDPKLASSVIIGLLRYWPITNSQKEVMFLSEIEEILETISTAE-FQKC 370
gi|Oryza_sativa_N/A                 IEKEPKLAGTVIRGLLKYWPVTNSQKEMMFLGELEEVLELTEMAE-FQKC 351
gi|Arabidopsis_thaliana_alpha       VEKDYKLADTVIRGLLKFWPVTNCTKEVLFLGELEEVLEATQTVE-FQRC 369
gi|Arabidopsis_thaliana_beta        VEKDYKLADTVIRGLLKYWPVTNCSKENLFLGELEEVLEATQPVE-FQRC 369
gi|Oryza_sativa_N/A_2               VEKDYKLADTVIRGLLKYWPVINCQKEVLFLGELEEVLEATQPAE-FQRC 409
gi|Arabidopsis_thaliana_epsilo      VEKDYKLADTVIRGLLKFWPLTNCQKEVLFLGELEEVLDATEPSE-FQQC 378
gi|Chlamydomonas_reinhardtii_w      VEKDSKLAEPVLTALLKYWPVTNSQKEVLFLGELEEILELTQAPE-FQKV 367
gi|Schizosaccharomyces_pombe_P      VEKDSSVTEEVVLGLLRYWPKVNSSKEVLFLNEIEDIIEVMEPSE-FLKI 402
gi|Schizosaccharomyces_pombe_P      IDKDPSLTKAVLTGILKYWPRINSFKELLFLNEIEDIFEVLEPSE-FVNI 503
gi|Ashbya_gossypii_RTS1             LEKEPMLTEEVIMGLLRYWPKVNSTKEIMFLNEIEDIFEVIEPLE-FIKV 465
gi|Saccharomyces_cerevisiae_RT      LEKDPFLTEEVVMGLLRYWPKINSTKEIMFLNEIEDIFEVIEPLE-FIKV 572

gi|Aspergillus_niger_N/A            QVPLFQQLAKSVASPHFQ-------------------------------- 474
gi|Aspergillus_nidulans_parA        QEPLFQQLAKSVASPHFQ-------------------------------- 476
gi|Mus_musculus_alpha               EEPLFKQISKCVSSSHFQ-------------------------------- 366
gi|Rattus_norvegicus_alpha          EEPLFKQISKCVSSSHFQ-------------------------------- 366
gi|Macaca_mulatta_alpha             EEPLFKQISKCVSSSHFQ-------------------------------- 366
gi|Homo_sapiens_alpha               EEPLFKQISKCVSSSHFQ-------------------------------- 366
gi|Ovis_aries_alpha                 EEPLFKQISKCVSSSHFQ-------------------------------- 369
gi|Bos_taurus_alpha                 EEPLFKQISKCVSSSHFQ-------------------------------- 369
gi|Felis_catus_alpha                EEPLFKQISKCVSSSHFQ-------------------------------- 282
gi|Canis_lupus_familiaris_alph      EEPLFKQISKCVSSSHFQ-------------------------------- 282
gi|Falco_peregrinus_alpha           EEPLFKQISKCVSSSHFQ-------------------------------- 356
gi|Gallus_gallus_alpha              EEPLFKQISKCVSSSHFQ-------------------------------- 356
gi|Alligator_mississippiensis_      EEPLFKQISKCVSSSHFQ-------------------------------- 366
gi|Chrysemys_picta_bellii_alph      EEPLFKQISKCVSSSHFQ-------------------------------- 356
```

Figure S1. *Cont.*

```
gi|Xenopus_laevis_alpha            QEPLFKQISKCVSSSHFQ-------------------------------- 355
gi|Xenopus_tropicalis_alpha        QEPLFKQISKCVSSSHFQ-------------------------------- 355
gi|Ambystoma_mexicanum_alpha       QEPLFKQISKCVSSSHFQ-------------------------------- 282
gi|Danio_rerio_alpha               QEPLFKQIAKCVASPHFQ-------------------------------- 358
gi|Petromyzon_marinus_S4RHV1       QEPLFKQISRCVASPHLH-------------------------------- 356
gi|Mus_musculus_epsilon            QEPLFKQIAKCVSSPHFQ-------------------------------- 358
gi|Rattus_norvegicus_epsilon       QEPLFKQIAKCVSSPHFQ-------------------------------- 358
gi|Macaca_mulatta_epsilon          QEPLFKQIAKCVSSPHFQ-------------------------------- 358
gi|Homo_sapiens_epsilon            QEPLFKQIAKCVSSPHFQ-------------------------------- 358
gi|Ovis_arie_epsilon               QEPLFKQIAKCVSSPHFQ-------------------------------- 358
gi|Bos_taurus_epsilon              QEPLFKQIAKCVSSPHFQ-------------------------------- 358
gi|Canis_lupus_familiaris_epsi     QEPLFKQIAKCVSSPHFQ-------------------------------- 358
gi|Falco_peregrinus_epsilon        QEPLFKQIAKCVSSPHFQ-------------------------------- 358
gi|Gallus_gallus_epsilon           QEPLFKQIAKCVSSPHFQ-------------------------------- 358
gi|Felis_catus_epsilon             QEPLFKQIAKCVSSPHFQ-------------------------------- 358
gi|Chrysemys_picta_bellii_epsi     QEPLFKQIAKCVSSPHFQ-------------------------------- 358
gi|Xenopus_laevis_epsilon          QEPLFKQISKCVSSPHFQ-------------------------------- 358
gi|Xenopus_tropicalis_epsilon      QEPLFKQISKCVSSPHFQ-------------------------------- 358
gi|Alligator_mississippiensis_     QEPLFKQIAKCVSSPHFQ-------------------------------- 347
gi|Ambystoma_mexicanum_epsilon     QEPLFKQISKCVSSPHFQ-------------------------------- 354
gi|Danio_rerio_epsilon             QELLFKQIARCVSSPHFQ-------------------------------- 358
gi|Mus_musculus_beta               QEPLFKQVARCVSSPHFQ-------------------------------- 372
gi|Rattus_norvegicus_beta          QEPLFKQVARCVSSPHFQ-------------------------------- 372
gi|Felis_catus_beta                QEPLFKQVARCVSSPHFQ-------------------------------- 372
gi|Canis_lupus_familiaris_beta     QEPLFKQVARCVSSPHFQ-------------------------------- 372
gi|Homo_sapiens_beta               QEPLFKQVARCVSSPHFQ-------------------------------- 372
gi|Bos_taurus_beta                 QEPLFKQVARCVSSPHFQ-------------------------------- 372
gi|Ovis_aries_beta                 QEPLFKQVARCVSSPHFQ-------------------------------- 372
gi|Macaca_mulatta_beta             QEPLFKQVARCVSSPHFQ-------------------------------- 336
gi|Xenopus_laevis_beta             ------SVFTCC-------------------------------------- 6
gi|Alligator_mississippiensis_     QQPLFKQVAQCIASPHFQ-------------------------------- 372
gi|Chrysemys_picta_bellii_beta     QEPLFKQIARCISSPHFQ-------------------------------- 372
gi|Xenopus_tropicalis_beta         QEPLFKQIARCVSSPHFQ-------------------------------- 370
gi|Danio_rerio_beta                QEPLFKQIAACISSPHFQ-------------------------------- 371
gi|Ambystoma_mexicanum_beta        QEPLFKQIARCIASPHFQ-------------------------------- 241
gi|Petromyzon_marinus_S4RGA7       QEPLFRQIAKCVSSPHFQ-------------------------------- 135
gi|Strongylocentrotus_purpurat     QEMLFRQLAKCVASPHFQ-------------------------------- 354
gi|Drosophila_melanogaster_wdb     QEPLFRQIAKCVSSPHFQ-------------------------------- 353
jgi|Branchiostoma_floridae_237     QEPLFKQISKCVSSPHFQ-------------------------------- 309
gi|Hydra_vulgaris_alpha            MEPLFKKISKCVSSPHFQ-------------------------------- 362
jgi|Branchiostoma_floridae_112     --------------------------------------------------
jgi|Branchiostoma_floridae_284     QEQLFKQMSKCVSSPHFQ-------------------------------- 322
gi|Caenorhabditis_elegans_PPTR     IDPLFRQLAKCVSSPHFQ-------------------------------- 383
gi|Amphimedon_queenslandica_be     QEPLFRQLAKCVSSPHFQ-------------------------------- 376
gi|Chrysemys_picta_bellii_gamm     MEPLFRQLAKCVSSPHFQ-------------------------------- 341
gi|Chrysemys_picta_bellii_delt     MEPLFRQLAKCVSSPHFQ-------------------------------- 266
gi|Falco_peregrinus_gamma          MEPLFRQLAKCVSSPHFQ-------------------------------- 341
gi|Falco_peregrinus_delta/gamm     MEPLFRQLAKCVSSPHFQ-------------------------------- 266
gi|Gallus_gallus_gamma             MEPLFRQLAKCVSSPHFQ-------------------------------- 341
gi|Alligator_mississippiensis_     MEPLFRQLAKCVSSPHFQ-------------------------------- 341
gi|Alligator_mississippiensis_     MEPLFRQLAKCVSSPHFQ-------------------------------- 266
gi|Ambystoma_mexicanum_gamma       MEPLFRQLAKCVSSPHFQ-------------------------------- 342
gi|Mus_musculus_gamma              MEPLFRQLAKCVSSPHFQ-------------------------------- 341
gi|Rattus_norvegicus_gamma         MEPLFRQLAKCVSSPHFQ-------------------------------- 341
gi|Ovis_aries_gamma                MEPLFRQLAKCVSSPHFQ-------------------------------- 341
gi|Bos_taurus_gamma                MEPLFRQLAKCVSSPHFQ-------------------------------- 341
gi|Canis_lupus_familiaris_gamm     MEPLFRQLAKCVSSPHFQ-------------------------------- 341
gi|Macaca_mulatta_gamma            MEPLFRQLAKCVSSPHFQ-------------------------------- 341
gi|Homo_sapiens_gamma              MEPLFRQLAKCVSSPHFQ-------------------------------- 331
gi|Felis_catus_gamma               MEPLFRQLAKCVSSPHFQ-------------------------------- 388
gi|Canis_lupus_familiaris_delt     MEPLFRQLAKCVSSPHFQ-------------------------------- 388
```

Figure S1. *Cont.*

```
gi|Macaca_mulatta_delta/gamma        MEPLFRQLAKCVSSPHFQ-------------------------------- 623
gi|Bos_taurus_delta/gamma            MEPLFRQLAKCVSSPHFQ-------------------------------- 395
gi|Mus_musculus_delta/gamma          MEPLFRQLAKCVSSPHFQ-------------------------------- 398
gi|Rattus_norvegicus_delta/gam       MEPLFRQLAKCVSSPHFQ-------------------------------- 464
gi|Danio_rerio_gamma                 MEPLFRQLAKCVSSPHFQ-------------------------------- 341
gi|Danio_rerio_delta/gamma           MEPLFRQLAKCVSSPHFQ-------------------------------- 394
gi|Xenopus_laevis_delta/gamma        MEPLFRQLAKCVSSPHFQ-------------------------------- 394
gi|Xenopus_laevis_gamma              MEPLFRQLAKCVSSPHFQ-------------------------------- 394
gi|Xenopus_tropicalis_delta/ga       MEPLFRQLAKCVSSPHFQ-------------------------------- 394
gi|Mus_musculus_delta                MEPLFRQLAKCVSSPHFQ-------------------------------- 409
gi|Rattus_norvegicus_delta           MEPLFRQLAKCVSSPHFQ-------------------------------- 437
gi|Felis_catus_delta                 MEPLFRQLAKCVSSPHFQ-------------------------------- 423
gi|Bos_taurus_delta                  MEPLFRQLAKCVSSPHFQ-------------------------------- 415
gi|Canis_lupus_familiaris_delt       MEPLFRQLAKCVSSPHFQ-------------------------------- 410
gi|Macaca_mulatta_delta              MEPLFRQLAKCVSSPHFQ-------------------------------- 426
gi|Homo_sapiens_delta                MEPLFRQLAKCVSSPHFQ-------------------------------- 417
gi|Ovis_aries_delta                  MEPLFRQLAKCVSSPHFQ-------------------------------- 414
gi|Falco_peregrinus_delta            MEPLFRQLAKCVSSPHFQ-------------------------------- 317
gi|Gallus_gallus_delta               MEPLFRQLAKCVSSPHFQ-------------------------------- 412
gi|Alligator_mississippiensis_       MEPLFRQLAKCVSSPHFQ-------------------------------- 411
gi|Chrysemys_picta_bellii_delt       MEPLFRQLAKCVSSPHFQ-------------------------------- 413
gi|Danio_rerio_delta                 MEPLFRQLAKCVSSPHFQ-------------------------------- 404
gi|Ambystoma_mexicanum_delta         MEPLFRQLAKCVSSPHFQ-------------------------------- 266
gi|Petromyzon_marinus_S4RN43         MEPLFRQLAKCVSSPHFQ-------------------------------- 394
jgi|Branchiostoma_floridae_237       MVPLFRQLAQCVSSPHFQ-------------------------------- 314
jgi|Branchiostoma_floridae_252       -IVLNEDFFFGYSN-ISQ-------------------------------- 358
gi|Strongylocentrotus_purpurat       MTPLFQQLANCVSSPHFQ-------------------------------- 375
gi|Caenorhabditis_elegans_PPTR       MTPLFSQIARCVSSPHFQ-------------------------------- 362
gi|Hydra_vulgaris_delta              VEPLFKKLGQCVSSQHFQ-------------------------------- 384
gi|Amphimedon_queenslandica_de       MQPLFKQISKCISSQHFQ-------------------------------- 425
gi|Drosophila_melanogaster_B56       MVPLFRQIAKCVSSPHFQ-------------------------------- 498
gi|Dictyostelium_discoideum_ps       RNQFFRQMTKCFQSEHFQ-------------------------------- 423
gi|Dictyostelium_purpureum_N/A       RTQFFRQMTKCFQSEHFQ-------------------------------- 380
gi|Dictyostelium_fasciculatum_       QQILFKQLARCFQSEHFQ-------------------------------- 394
gi|Polysphondylium_pallidum_N/       YIPLFKQITKCFNSEHFQ-------------------------------- 371
gi|Arabidopsis_thaliana_theta        MVPLFRQVARCLNSLHFQ-------------------------------- 388
gi|Arabidopsis_thaliana_eta          MVPLFRQIARCLNSLHFQAISFRHTLTALLMRSRYSLPVISNEQMEELIL 412
gi|Arabidopsis_thaliana_zeta         MVPLSRQIARCLNSSHFQ-------------------------------- 418
gi|Arabidopsis_thaliana_gamma        MVPLFRQIARCLNSSHFQ-------------------------------- 397
gi|Oryza                             MVPLFRQIACCLNSSHFQ-------------------------------- 398
gi|Oryza_sativa_zeta                 MVPLFRQIAHCLNSSHFQ-------------------------------- 398
gi|Oryza_sativa_theta                MVSLFCQIARCLNSSHFQ-------------------------------- 402
gi|Oryza_sativa_eta                  MVPLFRQIACSMNSSHFQ-------------------------------- 391
gi|Arabidopsis_thaliana_delta        MVPLSRQIAQCLSSSHFQ-------------------------------- 376
gi|Arabidopsis_thaliana_N/A          MVPLFLRIACCVTSSHFQ-------------------------------- 382
gi|Oryza_sativa_kappa                MVPLFRRIAQCIKSSHFQ-------------------------------- 388
gi|Oryza_sativa_N/A                  MVPLFRRIAHCLNSSHFQ-------------------------------- 369
gi|Arabidopsis_thaliana_alpha        MVPLFQQIARCLSSSNFQ-------------------------------- 387
gi|Arabidopsis_thaliana_beta         MVPLFQQIGRCLTSSHFQ-------------------------------- 387
gi|Oryza_sativa_N/A_2                MVPLFKQIGRCLNSSHFQ-------------------------------- 427
gi|Arabidopsis_thaliana_epsilo       VVPLFTQIGKCLNSAHFQ-------------------------------- 396
gi|Chlamydomonas_reinhardtii_w       MIPLFRQLAKCLNSQHFQ-------------------------------- 385
gi|Schizosaccharomyces_pombe_P       QVPLFHKLATSISSQNFQ-------------------------------- 420
gi|Schizosaccharomyces_pombe_P       MSPLFQQLARSISSMHFQ-------------------------------- 521
gi|Ashbya_gossypii_RTS1              EVPLFVQLAKCISSPHFQ-------------------------------- 483
gi|Saccharomyces_cerevisiae_RT       EVPLFVQLRKCISSPHFQ-------------------------------- 590

gi|Aspergillus_niger_N/A             ----------------VAERALYFWNNEYFCNLVSDNVEIILP--IMFPP 506
gi|Aspergillus_nidulans_parA         ----------------VAERALYFWNNEYFCNLVSDNVEVILP--IMFPP 508
gi|Mus_musculus_alpha                ----------------VAERALYFWNNEYILSLIEENIDKILP--IMFAS 398
```

Figure S1. *Cont.*

```
gi|Rattus_norvegicus_alpha            ----------------VAERALYFWNNEYILSLIEENIDKILP--IMFAS 398
gi|Macaca_mulatta_alpha               ----------------VAERALYFWNNEYILSLIEENIDKILP--IMFAS 398
gi|Homo_sapiens_alpha                 ----------------VAERALYFWNNEYILSLIEENIDKILP--IMFAS 398
gi|Ovis_aries_alpha                   ----------------VAERALYFWNNEYILSLIEENIDKILP--IMFGS 401
gi|Bos_taurus_alpha                   ----------------VAERALYFWNNEYILSLIEENIDKILP--IMFGS 401
gi|Felis_catus_alpha                  ----------------VAERALYFWNNEYILSLIEENIDKILP--IMFGS 314
gi|Canis_lupus_familiaris_alph        ----------------VAERALYFWNNEYILSLIEENIDKILP--IMFGS 314
gi|Falco_peregrinus_alpha             ----------------VAERALYFWNNEYILSLIEENIDKILP--IMFAS 388
gi|Gallus_gallus_alpha                ----------------VAERALYFWNNEYILSLIEENIDKILP--IMFGS 388
gi|Alligator_mississippiensis_        ----------------VAERALYFWNNEYILSLIEENIDKILP--IMFGS 398
gi|Chrysemys_picta_bellii_alph        ----------------VAERALYFWNNEYILSLIEENINKILP--IMFGS 388
gi|Xenopus_laevis_alpha               ----------------VAERALYFWNNEYILSLIEENIDKILP--IMFGS 387
gi|Xenopus_tropicalis_alpha           ----------------VAERALYFWNNEYILSLIEENIDKILP--IMFGS 387
gi|Ambystoma_mexicanum_alpha          ----------------VAERALYFWNNEYILSLIEENIDKILP--IMFGS 314
gi|Danio_rerio_alpha                  ----------------VAERALYYWNNEYILSLIEENNAKIFP--IMFGN 390
gi|Petromyzon_marinus_S4RHV1          ----------------VAEHALYYWNNKYILSLIEENSNVILP--IMFGN 388
gi|Mus_musculus_epsilon               ----------------VAERALYYWNNEYIMSLIEENSNVILP--IMFSS 390
gi|Rattus_norvegicus_epsilon          ----------------VAERALYYWNNEYIMSLIEENSNVILP--IMFSS 390
gi|Macaca_mulatta_epsilon             ----------------VAERALYYWNNEYIMSLIEENSNVILP--IMFSS 390
gi|Homo_sapiens_epsilon               ----------------VAERALYYWNNEYIMSLIEENSNVILP--IMFSS 390
gi|Ovis_arie_epsilon                  ----------------VAERALYYWNNEYIMSLIEENSNVILP--IMFSS 390
gi|Bos_taurus_epsilon                 ----------------VAERALYYWNNEYIMSLIEENSNVILP--IMFSS 390
gi|Canis_lupus_familiaris_epsi        ----------------VAERALYYWNNEYIMSLIEENSNVILP--IMFSS 390
gi|Falco_peregrinus_epsilon           ----------------VAERALYYWNNEYIMSLIEENSNVILP--IMFSS 390
gi|Gallus_gallus_epsilon              ----------------VAERALYYWNNEYIMSLIEENSNVILP--IMFSS 390
gi|Felis_catus_epsilon                ----------------VAERALYYWNNEYIMSLIEENSNVILP--IMFSS 390
gi|Chrysemys_picta_bellii_epsi        ----------------VAERALYYWNNEYIMSLIEENSNVILP--IMFSS 390
gi|Xenopus_laevis_epsilon             ----------------VAERALYYWNNEYIMSLIEENSNVVLP--IMFAS 390
gi|Xenopus_tropicalis_epsilon         ----------------VAERALYYWNNEYIMSLIEENSNVILP--IMFAS 390
gi|Alligator_mississippiensis_        ----------------VAERALYYWNNEYIMSLIEENSNVILP--IMFSS 379
gi|Ambystoma_mexicanum_epsilon        ----------------VAERALYYWNNEYIMSLIEENSNVILP--IMFAS 386
gi|Danio_rerio_epsilon                ----------------VAERALYYWNNEYIMSLIEENCSAILP--IMFAS 390
gi|Mus_musculus_beta                  ----------------VAERALYFWNNEYILSLIEDNCHTVLP--AVFGT 404
gi|Rattus_norvegicus_beta             ----------------VAERALYFWNNEYILSLIEDNCHTVLP--AVFGT 404
gi|Felis_catus_beta                   ----------------VAERALYFWNNEYILSLIEDNCHTVLP--AVFGT 404
gi|Canis_lupus_familiaris_beta        ----------------VAERALYFWNNEYILSLIEDNCHTVLP--AVFGT 404
gi|Homo_sapiens_beta                  ----------------VAERALYFWNNEYILSLIEDNCHTVLP--AVFGT 404
gi|Bos_taurus_beta                    ----------------VAERALYFWNNEYILSLIEDNCHTVLP--AVFGT 404
gi|Ovis_aries_beta                    ----------------VAERALYFWNNEYILSLIEDNCHTVLP--AVFGT 404
gi|Macaca_mulatta_beta                ----------------VAERALYFWNNEYILSLIEDNCHTVLP--AVFGT 368
gi|Xenopus_laevis_beta                ----------------------YYWD-------------GFLP--TSFS- 18
gi|Alligator_mississippiensis_        ----------------VAERALYFWNNEYILSLIEDNCQAVLP--AIFGT 404
gi|Chrysemys_picta_bellii_beta        ----------------VAERALYFWNNEYILSLIEDNCHTVLP--AIFGT 404
gi|Xenopus_tropicalis_beta            ----------------VAERALYFWNNEYILSLIEENCHTILP--LIFGT 402
gi|Danio_rerio_beta                   ----------------VAERALYFWNNEYILSLIEENCQVILP--LVFGT 403
gi|Ambystoma_mexicanum_beta           ----------------VAERALYFWNNEYILSLIEDNCHSILP--IIFST 273
gi|Petromyzon_marinus_S4RGA7          ----------------VAERAMYYWNNEYILGLMEENISTILP--IVFGS 167
gi|Strongylocentrotus_purpurat        ----------------VAERALYFWNNEYVVSLIEENIQAIMP--IMFTN 386
gi|Drosophila_melanogaster_wdb        ----------------VAERALYLWNNEYAMSLIEENNAVIMP--IMFPA 385
jgi|Branchiostoma_floridae_237        ----------------VAERALYFWNNEYVMSLIEENSNVIMP--IMFPN 341
gi|Hydra_vulgaris_alpha               ----------------VAERALYFWNNEYIMSLIDENSAVILP--IMFSC 394
jgi|Branchiostoma_floridae_112        --------------------------------------------------
jgi|Branchiostoma_floridae_284        ----------------VAERALYFWNNEYVMSLIEENSNVILP--IMFSC 354
gi|Caenorhabditis_elegans_PPTR        ----------------VAERALYFWNNEYILSLIEDTSSLVMP--IMFPA 415
gi|Amphimedon_queenslandica_be        ----------------VAERALYFWNNEYIVGLIQENSEVIMP--IMFDA 408
gi|Chrysemys_picta_bellii_gamm        ----------------VAERALYYWNNEYIMSLISDNAAKILP--IMFPS 373
gi|Chrysemys_picta_bellii_delt        ----------------VAERALYYWNNEYIMSLISDNAAKILP--IMFPS 298
gi|Falco_peregrinus_gamma             ----------------VAERALYYWNNEYIMSLISDNAAKILP--IMFPS 373
gi|Falco_peregrinus_delta/gamm        ----------------VAERALYYWNNEYIMSLISDNAAKILP--IMFPS 298
gi|Gallus_gallus_gamma                ----------------VAERALYYWNNEYIMSLISDNAAKILP--IMFPS 373
gi|Alligator_mississippiensis_        ----------------VAERALYYWNNEYIMSLISDNAAKILP--IMFPS 373
```

Figure S1. *Cont.*

```
gi|Alligator_mississippiensis_      ----------------VAERALYYWNNEYIMSLISDNAAKILP--IMFPS 298
gi|Ambystoma_mexicanum_gamma        ----------------VAERALYYWNNEYIMSLISDNAAKILP--IMFPS 374
gi|Mus_musculus_gamma               ----------------VAERALYYWNNEYIMSLISDNAAKILP--IMFPS 373
gi|Rattus_norvegicus_gamma          ----------------VAERALYYWNNEYIMSLISDNAAKILP--IMFPS 373
gi|Ovis_aries_gamma                 ----------------VAERALYYWNNEYIMSLISDNAAKILP--IMFPS 373
gi|Bos_taurus_gamma                 ----------------VAERALYYWNNEYIMSLISDNAAKILP--IMFPS 373
gi|Canis_lupus_familiaris_gamm      ----------------VAERALYYWNNEYIMSLISDNAAKILP--IMFPS 373
gi|Macaca_mulatta_gamma             ----------------VAERALYYWNNEYIMSLISDNAAKILP--IMFPS 373
gi|Homo_sapiens_gamma               ----------------VAERALYYWNNEYIMSLISDNAAKILP--IMFPS 363
gi|Felis_catus_gamma                ----------------VAERALYYWNNEYIMSLISDNAAKILP--IMFPS 420
gi|Canis_lupus_familiaris_delt      ----------------VAERALYYWNNEYIMSLISDNAAKILP--IMFPS 420
gi|Macaca_mulatta_delta/gamma       ----------------VAERALYYWNNEYIMSLISDNAAKILP--IMFPS 655
gi|Bos_taurus_delta/gamma           ----------------VAERALYYWNNEYIMSLISDNAAKILP--IMFPS 427
gi|Mus_musculus_delta/gamma         ----------------VAERALYYWNNEYIMSLISDNAAKILP--IMFPS 430
gi|Rattus_norvegicus_delta/gam      ----------------VAERALYYWNNEYIMSLISDNAAKILP--IMFPS 496
gi|Danio_rerio_gamma                ----------------VAERALYYWNNEYIMSLISDNAAKILP--IMFPS 373
gi|Danio_rerio_delta/gamma          ----------------VAERALYYWNNEYIMSLISDNAAKILP--IMFPS 426
gi|Xenopus_laevis_delta/gamma       ----------------VAERALYYWNNEYIMSLISDNAAKILP--IMFPS 426
gi|Xenopus_laevis_gamma             ----------------VAERALYYWNNEYIMSLISDNAAKILP--IMFPS 426
gi|Xenopus_tropicalis_delta/ga      ----------------VAERALYYWNNEYIMSLISDNAAKILP--IMFPS 426
gi|Mus_musculus_delta               ----------------VAERALYYWNNEYIMSLISDNAARILP--IMFPA 441
gi|Rattus_norvegicus_delta          ----------------VAERALYYWNNEYIMSLISDNAARVLP--IMFPA 469
gi|Felis_catus_delta                ----------------VAERALYYWNNEYIMSLISDNAARVLP--IMFPA 455
gi|Bos_taurus_delta                 ----------------VAERALYYWNNEYIMSLISDNAARVLP--IMFPA 447
gi|Canis_lupus_familiaris_delt      ----------------VAERALYYWNNEYIMSLISDNAARVLP--IMFPA 442
gi|Macaca_mulatta_delta             ----------------VAERALYYWNNEYIMSLISDNAARVLP--IMFPA 458
gi|Homo_sapiens_delta               ----------------VAERALYYWNNEYIMSLISDNAARVLP--IMFPA 449
gi|Ovis_aries_delta                 ----------------VAERALYYWNNEYIMSLISDNAARVLP--IMFPA 446
gi|Falco_peregrinus_delta           ----------------VAERALYYWNNEYIMSLISDNAAKILP--IMFPA 349
gi|Gallus_gallus_delta              ----------------VAERALYYWNNEYIMSLISDNAAKILP--IMFPA 444
gi|Alligator_mississippiensis_      ----------------VAERALYYWNNEYIMSLISDNAAKILP--IMFPA 443
gi|Chrysemys_picta_bellii_delt      ----------------VAERALYYWNNEYIMSLISDNAAKILP--IMFPA 445
gi|Danio_rerio_delta                ----------------VAERALYYWNNEYIMSLISDNAAKILP--IMFPA 436
gi|Ambystoma_mexicanum_delta        ----------------VAERALYYWNNEYIMSLISDNAARILP--IMFPA 298
gi|Petromyzon_marinus_S4RN43        ----------------VAERALYYWNNEYIMSLVSDNAAKILP--IMFPA 426
jgi|Branchiostoma_floridae_237      ----------------VAERALYYWNNEYIMSLISDNAQVILP--IMFPA 346
jgi|Branchiostoma_floridae_252      ----------------VAERALYYWNNEYIMSLISDNAQVILP--IMFPA 390
gi|Strongylocentrotus_purpurat      ----------------VAERALYYWNNEYIMSLISDNDETILP--IMFPA 407
gi|Caenorhabditis_elegans_PPTR      ----------------VAERALYYWNNEYVMSLVADNARVIIP--IMFPV 394
gi|Hydra_vulgaris_delta             ----------------VAERALYYWNNEYIMSLVSDNATKAIP--IIFPY 416
gi|Amphimedon_queenslandica_de      ----------------VAERALYYWSNDYIMSLVGENPQALFP--IILPA 457
gi|Drosophila_melanogaster_B56      ----------------VAERALYYWNNEYIMSLITDNSAVILP--IMFPA 530
gi|Dictyostelium_discoideum_ps      ----------------VAERALYLFSNENIVLLIASKNNFTLALETFYKP 457
gi|Dictyostelium_purpureum_N/A      ----------------VAERALYLFSNENIVLLIASKNNFTLALETFYKP 414
gi|Dictyostelium_fasciculatum_      ----------------VAERSLLLLSNEHIVGLLSSKRSVPLALNYFYKI 428
gi|Polysphondylium_pallidum_N/      ----------------VAERALLLWSNDHVVSLVSSKTCLP-------NV 398
gi|Arabidopsis_thaliana_theta       ----------------VAERALFLWNNDHIENLIMQNRKVILP--IIFPA 420
gi|Arabidopsis_thaliana_eta         RYGKYQCLGLLYSALGVAERALFLWNNNHIENLIMQNRKVILP--IIFPA 460
gi|Arabidopsis_thaliana_zeta        ----------------VAERALFLWNNDHIRNLITQNHKVIMP--IVFPA 450
gi|Arabidopsis_thaliana_gamma       ----------------VAERALFLWNNDHIRNLITQNHKVIMP--IVFPA 429
gi|Oryza                            ----------------VAERALFLWNNDHIENLIRQNSKVILP--IIFSA 430
gi|Oryza_sativa_zeta                ----------------VAERALFLWNNDHIENLIKQNSRVILP--IIFPA 430
gi|Oryza_sativa_theta               ----------------VAERALFLWNNDHIEVLIKQNSKVILP--IILPA 434
gi|Oryza_sativa_eta                 ----------------VAERALFLWNNDHIENLIKQNYKVILP--IIFPA 423
gi|Arabidopsis_thaliana_delta       ----------------VAERALYLWNNDHVTNLVRQNSRIILP--IVFPA 408
gi|Arabidopsis_thaliana_N/A         ----------------VSERALFLWNNDQIVNLIGHNRQAILP--IMFTA 414
gi|Oryza_sativa_kappa               ----------------VAERALFIWNNDNVISLIAQNRQMIMP--IIVPA 420
gi|Oryza_sativa_N/A                 ----------------VAERALFLWNNEHLFDLISQNRQTIMP--IIYPA 401
gi|Arabidopsis_thaliana_alpha       ----------------VAERALFLWNNEHVVGLIAQNRGVILP--IIFAS 419
gi|Arabidopsis_thaliana_beta        ----------------VAERALFLWNNEHIVGLIAQNRSVILP--IIYPT 419
gi|Oryza_sativa_N/A_2               ----------------VAERALFLWNNDHIVSLIAQNRSVIFP--IIFEA 459
```

Figure S1. *Cont.*

```
gi|Arabidopsis_thaliana_epsilo      ----------------VAERALFLWNNEHIVGLIAQNKDVIFP--IIFEA 428
gi|Chlamydomonas_reinhardtii_w      ----------------VAERSLFLWNNEYIVNLVAQHRHQLLP--ILFPA 417
gi|Schizosaccharomyces_pombe_P      ----------------VAERALYFFNNDYFVHLVEENVDIILP--IIYPA 452
gi|Schizosaccharomyces_pombe_P      ----------------VAERALCLWSNEYFTSLVSQNVVTLLP--IIYPS 553
gi|Ashbya_gossypii_RTS1             ----------------VAEKVLSYWNNEYFLNLCIENAEVILP--IIFPA 515
gi|Saccharomyces_cerevisiae_RT      ----------------VAEKVLSYWNNEYFLNLCIENAEVILP--IIFPA 622

gi|Aspergillus_niger_N/A            LYENS----------------------------KGHWNRTIHSMVYNAMK 528
gi|Aspergillus_nidulans_parA        LFENS----------------------------KGHWNRTIHSMVYNAMK 530
gi|Mus_musculus_alpha               LYKIS----------------------------KEHWNQTIVALVYNVLK 420
gi|Rattus_norvegicus_alpha          LYKIS----------------------------KEHWNQTIVALVYNVLK 420
gi|Macaca_mulatta_alpha             LYKIS----------------------------KEHWNPTIVALVYNVLK 420
gi|Homo_sapiens_alpha               LYKIS----------------------------KEHWNPTIVALVYNVLK 420
gi|Ovis_aries_alpha                 LYKIS----------------------------KEHWNPTIVALVYNVLK 423
gi|Bos_taurus_alpha                 LYKIS----------------------------KEHWNPTIVALVYNVLK 423
gi|Felis_catus_alpha                LYKIS----------------------------KEHWNPTIVALVYNVLK 336
gi|Canis_lupus_familiaris_alph      LYKIS----------------------------KEHWNPTIVALVYNVLK 336
gi|Falco_peregrinus_alpha           LYKIS----------------------------KEHWNPTIVALVYNVLK 410
gi|Gallus_gallus_alpha              LYKIS----------------------------KEHWNPTIVALVYNVLK 410
gi|Alligator_mississippiensis_      LYKIS----------------------------KEHWNPTIVALVYNVLK 420
gi|Chrysemys_picta_bellii_alph      LYKIS----------------------------KEHWNPTIVALVYNVLK 410
gi|Xenopus_laevis_alpha             LYKIS----------------------------KEHWNPTIVALVYNVLK 409
gi|Xenopus_tropicalis_alpha         LYKIS----------------------------KEHWNPTIVALVYNVLK 409
gi|Ambystoma_mexicanum_alpha        LYKIS----------------------------KEHWNPTIVALVYNVLK 336
gi|Danio_rerio_alpha                LYRIS----------------------------KEHWNPTIVALVYNVLK 412
gi|Petromyzon_marinus_S4RHV1        LYKMS----------------------------EEHWNQTILGLNFNVMK 410
gi|Mus_musculus_epsilon             LYRIS----------------------------KEHWNPAIVALVYNVLK 412
gi|Rattus_norvegicus_epsilon        LYRIS----------------------------KEHWNPAIVALVYNVLK 412
gi|Macaca_mulatta_epsilon           LYRIS----------------------------KEHWNPAIVALVYNVLK 412
gi|Homo_sapiens_epsilon             LYRIS----------------------------KEHWNPAIVALVYNVLK 412
gi|Ovis_arie_epsilon                LYRIS----------------------------KEHWNPAIVALVYNVLK 412
gi|Bos_taurus_epsilon               LYRIS----------------------------KEHWNPAIVALVYNVLK 412
gi|Canis_lupus_familiaris_epsi      LYRIS----------------------------KEHWNPAIVALVYNVLK 412
gi|Falco_peregrinus_epsilon         LYRIS----------------------------KEHWNPAIVALVYNVLK 412
gi|Gallus_gallus_epsilon            LYRIS----------------------------KEHWNPAIVALVYNVLK 412
gi|Felis_catus_epsilon              LYRIX----------------------------KEHWNPAIVALVYNVLK 412
gi|Chrysemys_picta_bellii_epsi      LYRIS----------------------------KEHWNPAIVALVYNVLK 412
gi|Xenopus_laevis_epsilon           LYRIS----------------------------KEHWNPAIVALVYNVLK 412
gi|Xenopus_tropicalis_epsilon       LYRIS----------------------------KEHWNPAIVALVYNVLK 412
gi|Alligator_mississippiensis_      LYRIS----------------------------KEHWNPAIVALVYNVLK 401
gi|Ambystoma_mexicanum_epsilon      LYRIS----------------------------KEHWNPAIVALVYNVLK 408
gi|Danio_rerio_epsilon              LYRIS----------------------------KEHWNPAISALIYNVLK 412
gi|Mus_musculus_beta                LYQVS----------------------------KEHWNQTIVSLIYNVLK 426
gi|Rattus_norvegicus_beta           LYQVS----------------------------KEHWNQTIVSLIYNVLK 426
gi|Felis_catus_beta                 LYQVS----------------------------KEHWNQTIVSLIYNVLK 426
gi|Canis_lupus_familiaris_beta      LYQVS----------------------------KEHWNQTIVSLIYNVLK 426
gi|Homo_sapiens_beta                LYQVS----------------------------KEHWNQTIVSLIYNVLK 426
gi|Bos_taurus_beta                  LYQVS----------------------------KEHWNQTIVSLIYNVLK 426
gi|Ovis_aries_beta                  LYQVS----------------------------KEHWNQTIVSLIYNVLK 426
gi|Macaca_mulatta_beta              LYQVS----------------------------KEHWNQTIVSLIYNVLK 390
gi|Xenopus_laevis_beta              ----------------------------------AHR--TIVSLIYNVLK 32
gi|Alligator_mississippiensis_      LYRVS----------------------------KEHWNQTIVSLVYNVLK 426
gi|Chrysemys_picta_bellii_beta      LYRVS----------------------------KEHWNQTIVSLVYNVLK 426
gi|Xenopus_tropicalis_beta          LYQVS----------------------------KEHWNQTIVSLIYNVLK 424
gi|Danio_rerio_beta                 LYRVS----------------------------KEHWNQTIISLIYNVLK 425
gi|Ambystoma_mexicanum_beta         LYRVS----------------------------KEHWNQTIVSLIYNVLK 295
gi|Petromyzon_marinus_S4RGA7        LYRVS----------------------------KEHWNQTIVALVYNILK 189
gi|Strongylocentrotus_purpurat      LYRIS----------------------------KDHWNQTIVALVYNVLR 408
gi|Drosophila_melanogaster_wdb      LYRIS----------------------------KEHWNQTIVALVYNVLK 407
jgi|Branchiostoma_floridae_237      LYRIS----------------------------KEHWNQTIVALVYNVLK 363
```

Figure S1. *Cont.*

```
gi|Hydra_vulgaris_alpha              LYRIS----------------------------KDHWNQTIVALVYNVLK 416
jgi|Branchiostoma_floridae_112       --------------------------------------------------
jgi|Branchiostoma_floridae_284       LYRIS----------------------------KDHWNQTIVALVYNVLK 376
gi|Caenorhabditis_elegans_PPTR       LYRIS----------------------------KEHWNQTIVALVYNVLK 437
gi|Amphimedon_queenslandica_be       LYRIS----------------------------KEHWNKAIVSLVYNVLK 430
gi|Chrysemys_picta_bellii_gamm       LYRNS----------------------------KTHWNKTIHGLIYNALK 395
gi|Chrysemys_picta_bellii_delt       LYRNS----------------------------KTHWNKTIHGLIYNALK 320
gi|Falco_peregrinus_gamma            LYRNS----------------------------KTHWNKTIHGLIYNALK 395
gi|Falco_peregrinus_delta/gamm       LYRNS----------------------------KTHWNKTIHGLIYNALK 320
gi|Gallus_gallus_gamma               LYRNS----------------------------KTHWNKTIHGLIYNALK 395
gi|Alligator_mississippiensis_       LYRNS----------------------------KTHWNKTIHGLIYNALK 395
gi|Alligator_mississippiensis_       LYRNS----------------------------KTHWNKTIHGLIYNALK 320
gi|Ambystoma_mexicanum_gamma         LYRNS----------------------------KTHWNKTIHGLIYNALK 396
gi|Mus_musculus_gamma                LYRNS----------------------------KTHWNKTIHGLIYNALK 395
gi|Rattus_norvegicus_gamma           LYRNS----------------------------KTHWNKTIHGLIYNALK 395
gi|Ovis_aries_gamma                  LYRNS----------------------------KTHWNKTIHGLIYNALK 395
gi|Bos_taurus_gamma                  LYRNS----------------------------KTHWNKTIHGLIYNALK 395
gi|Canis_lupus_familiaris_gamm       LYRNS----------------------------KTHWNKTIHGLIYNALK 395
gi|Macaca_mulatta_gamma              LYRNS----------------------------KTHWNKTIHGLIYNALK 395
gi|Homo_sapiens_gamma                LYRNS----------------------------KTHWNKTIHGLIYNALK 385
gi|Felis_catus_gamma                 LYRNS----------------------------KTHWNKTIHGLIYNALK 442
gi|Canis_lupus_familiaris_delt       LYRNS----------------------------KTHWNKTIHGLIYNALK 442
gi|Macaca_mulatta_delta/gamma        LYRNS----------------------------KTHWNKTIHGLIYNALK 677
gi|Bos_taurus_delta/gamma            LYRNS----------------------------KTHWNKTIHGLIYNALK 449
gi|Mus_musculus_delta/gamma          LYRNS----------------------------KTHWNKTIHGLIYNALK 452
gi|Rattus_norvegicus_delta/gam       LYRNS----------------------------KTHWNKTIHGLIYNALK 518
gi|Danio_rerio_gamma                 LYRNS----------------------------KTHWNKTIHGLIYNALK 395
gi|Danio_rerio_delta/gamma           LYRNS----------------------------KTHWNKTIHGLIYNALK 448
gi|Xenopus_laevis_delta/gamma        LYRNS----------------------------KTHWNKTIHGLIYNALK 448
gi|Xenopus_laevis_gamma              LYRNS----------------------------KTHWNKTIHGLIYNALK 448
gi|Xenopus_tropicalis_delta/ga       LYRNS----------------------------KTHWNKTIHGLIYNALK 448
gi|Mus_musculus_delta                LYRNS----------------------------KSHWNKTIHGLIYNALK 463
gi|Rattus_norvegicus_delta           LYRNS----------------------------KSHWNKTIHGLIYNALK 491
gi|Felis_catus_delta                 LYRNS----------------------------KSHWNKTIHGLIYNALK 477
gi|Bos_taurus_delta                  LYRNS----------------------------KSHWNKTIHGLIYNALK 469
gi|Canis_lupus_familiaris_delt       LYRNS----------------------------KSHWNKTIHGLIYNALK 464
gi|Macaca_mulatta_delta              LYRNS----------------------------KSHWNKTIHGLIYNALK 480
gi|Homo_sapiens_delta                LYRNS----------------------------KSHWNKTIHGLIYNALK 471
gi|Ovis_aries_delta                  LYRNS----------------------------KSHWNKTIHGLIYNALK 468
gi|Falco_peregrinus_delta            LYKNS----------------------------KSHWNKTIHGLIYNALK 371
gi|Gallus_gallus_delta               LYKNS----------------------------KSHWNKTIHGLIYNALK 466
gi|Alligator_mississippiensis_       LYKNS----------------------------KSHWNKTIHGLIYNALK 465
gi|Chrysemys_picta_bellii_delt       LYKNS----------------------------KSHWNKTIHGLIYNALK 467
gi|Danio_rerio_delta                 LYKNS----------------------------KSHWNKTIHGLIYNALK 458
gi|Ambystoma_mexicanum_delta         LYKNS----------------------------KSHWNKTIHGLIYNALK 320
gi|Petromyzon_marinus_S4RN43         LYRNS----------------------------KSHWNKTIHGLIYNALK 448
jgi|Branchiostoma_floridae_237       LYKNS----------------------------KTHWNKTIHGLIYNALK 368
jgi|Branchiostoma_floridae_252       LYKNS----------------------------KTHWNKTIHGLIYNALK 412
gi|Strongylocentrotus_purpurat       LYKNS----------------------------KAHWNKTINGLIYNALK 429
gi|Caenorhabditis_elegans_PPTR       LFKNS----------------------------KSHWNKTIHGLIYNALK 416
gi|Hydra_vulgaris_delta              LYRNS----------------------------KTHWNKTIHGLIYNALK 438
gi|Amphimedon_queenslandica_de       LLRHS----------------------------KQHWNKTILGLIYNALK 479
gi|Drosophila_melanogaster_B56       LNRNS----------------------------KTHWNKNIHGLIYNALK 552
gi|Dictyostelium_discoideum_ps       LHENS----------------------------ISHWNRSIRNLSISSLK 479
gi|Dictyostelium_purpureum_N/A       LYENS----------------------------IGHWNKSIRNLSISSLK 436
gi|Dictyostelium_fasciculatum_       LHDNA----------------------------NNHWNKTIRSLSFNSLK 450
gi|Polysphondylium_pallidum_N/       LYENS----------------------------NNHWNKSIRSLSFSSLK 420
gi|Arabidopsis_thaliana_theta        LERNT----------------------------QKHWNQAVHSLTLNVQK 442
gi|Arabidopsis_thaliana_eta          LERNA----------------------------QKHWNQAVHSLTLNVRK 482
gi|Arabidopsis_thaliana_zeta         LERNT----------------------------RGHWNQAVQSLTINVRK 472
gi|Arabidopsis_thaliana_gamma        MERNT----------------------------RGHWNQAVQSLTLNVRK 451
```

Figure S1. *Cont.*

```
gi|Oryza                             LEKNV----------------------------IEHWNQAVKSLSLNVQK 452
gi|Oryza_sativa_zeta                 LERNA----------------------------NGHWNQAVQSLTLNVRK 452
gi|Oryza_sativa_theta                IERNT----------------------------KEHWNQAVQSLSLNVRK 456
gi|Oryza_sativa_eta                  LERNA----------------------------RGHWNQAVRSLTLNVRK 445
gi|Arabidopsis_thaliana_delta        LEKNG----------------------------SSHWNQAVKNLTENVLK 430
gi|Arabidopsis_thaliana_N/A          LEKNA----------------------------QNHWNQSVLNLTLNVRK 436
gi|Oryza_sativa_kappa                LEHNS----------------------------QNHWNQAVLNLTDNVKK 442
gi|Oryza_sativa_N/A                  LERNT----------------------------RWHWNQSVLNVTMNVRK 423
gi|Arabidopsis_thaliana_alpha        LEKNI----------------------------ESHWNQAVHGLSANIKR 441
gi|Arabidopsis_thaliana_beta         LEKNI----------------------------QSHWNQAVHGLTTNIKK 441
gi|Oryza_sativa_N/A_2                LERNI----------------------------TSHWNQAVHGLTANVRK 481
gi|Arabidopsis_thaliana_epsilo       LERNM----------------------------KGHWNQAVHGLSENVRR 450
gi|Chlamydomonas_reinhardtii_w       LEENT----------------------------NSHWNPAVHGLTCNVRK 439
gi|Schizosaccharomyces_pombe_P       LFEIS----------------------------KSHWNRVIHSMVCNVLK 474
gi|Schizosaccharomyces_pombe_P       LYKTA----------------------------NEHWNSTIQAIACNVLQ 575
gi|Ashbya_gossypii_RTS1              LYELTSQLDLDSQTDEEGNPNQDPYMLVEQAINSGSWNRAIHAMAFKALK 565
gi|Saccharomyces_cerevisiae_RT       LYELTSQLELDTANGED--SISDPYMLVEQAINSGSWNRAIHAMAFKALK 670

gi|Aspergillus_niger_N/A             MFMEINPQLFDECSHEYNERQNS--------------------------- 551
gi|Aspergillus_nidulans_parA         MFMEINPQLFDECSHEYNERQNS--------------------------- 553
gi|Mus_musculus_alpha                TLMEMNGKLFDDLTSSYKAERQR--------------------------- 443
gi|Rattus_norvegicus_alpha           TLMEMNGKLFDDLTSSYKAERQR--------------------------- 443
gi|Macaca_mulatta_alpha              TLMEMNGKLFDDLTSSYKAERQR--------------------------- 443
gi|Homo_sapiens_alpha                TLMEMNGKLFDDLTSSYKAERQR--------------------------- 443
gi|Ovis_aries_alpha                  TLMEMNGKLFDDLTSSYKAERQR--------------------------- 446
gi|Bos_taurus_alpha                  TLMEMNGKLFDDLTSSYKAERQR--------------------------- 446
gi|Felis_catus_alpha                 TLMEMNGKLFDDLTSSYKAERQR--------------------------- 359
gi|Canis_lupus_familiaris_alph       TLMEMNGKLFDDLTSSYKAERQR--------------------------- 359
gi|Falco_peregrinus_alpha            TLMEMNGKLFDELTSSYKAERQR--------------------------- 433
gi|Gallus_gallus_alpha               TLMEMNGKLFDELTSSYKAERQR--------------------------- 433
gi|Alligator_mississippiensis_       TLMEMNGKLFDELTSTYKAERQR--------------------------- 443
gi|Chrysemys_picta_bellii_alph       TLMEMNGKLFDELTSSYKAERQR--------------------------- 433
gi|Xenopus_laevis_alpha              TLMEMNGKLFDELTGSYKAERQR--------------------------- 432
gi|Xenopus_tropicalis_alpha          TLMEMNGKLFDELTGSYKAERQR--------------------------- 432
gi|Ambystoma_mexicanum_alpha         TLMEMNGKLFDELTGSYKAERQR--------------------------- 359
gi|Danio_rerio_alpha                 TMMEMNSKLFDELTTSYKSDRQR--------------------------- 435
gi|Petromyzon_marinus_S4RHV1         TMMDMNCELFGELTESYNNERQR--------------------------- 433
gi|Mus_musculus_epsilon              AFMEMNSTMFDELTATYKSDRQR--------------------------- 435
gi|Rattus_norvegicus_epsilon         AFMEMNSTMFDELTATYKSDRQR--------------------------- 435
gi|Macaca_mulatta_epsilon            AFMEMNSTMFDELTATYKSDRQR--------------------------- 435
gi|Homo_sapiens_epsilon              AFMEMNSTMFDELTATYKSDRQR--------------------------- 435
gi|Ovis_arie_epsilon                 AFMEMNSTMFDELTATYKSDRQR--------------------------- 435
gi|Bos_taurus_epsilon                AFMEMNSTMFDELTATYKSDRQR--------------------------- 435
gi|Canis_lupus_familiaris_epsi       AFMEMNSTMFDELTATYKSDRQR--------------------------- 435
gi|Falco_peregrinus_epsilon          AFMEMNSTMFDELTATYKSDRQR--------------------------- 435
gi|Gallus_gallus_epsilon             AFMEMNSTMFDELTATYKSDRQR--------------------------- 435
gi|Felis_catus_epsilon               AFMEMNSTMFDELTATYKSDRQR--------------------------- 435
gi|Chrysemys_picta_bellii_epsi       AFMEMNSTMFDELTATYN-------------------------------- 430
gi|Xenopus_laevis_epsilon            AFMEMNSTLFDELTATYKSDRQR--------------------------- 435
gi|Xenopus_tropicalis_epsilon        AFMEMNSTLFDELTATYN-------------------------------- 430
gi|Alligator_mississippiensis_       AFMEMNSTMFDELTATYKSDRQRLSKAAGEKNEETSGTPTRLSRAFCVTQ 451
gi|Ambystoma_mexicanum_epsilon       AFMEMNSTLFDELTATYKSDRQR--------------------------- 431
gi|Danio_rerio_epsilon               AFMEMNSALFDELAASYKSDRQR--------------------------- 435
gi|Mus_musculus_beta                 TFMEMNGKLFDELTASYKLEKQQ--------------------------- 449
gi|Rattus_norvegicus_beta            TFMEMNGKLFDELTASYKLEKQQ--------------------------- 449
gi|Felis_catus_beta                  TFMEMNGKLFDELTASYKLEKQQ--------------------------- 449
gi|Canis_lupus_familiaris_beta       TFMEMNGKLFDELTASYKLEKQQ--------------------------- 449
gi|Homo_sapiens_beta                 TFMEMNGKLFDELTASYKLEKQQ--------------------------- 449
gi|Bos_taurus_beta                   TFMEMNGKLFDELTASYKLEKQQ--------------------------- 449
gi|Ovis_aries_beta                   TFMEMNGKLFDELTASYKLEKQQ--------------------------- 449
```

Figure S1. *Cont.*

```
gi|Macaca_mulatta_beta              TFMEMNGKLFDELTASYKLEKQQ--------------------------- 413
gi|Xenopus_laevis_beta              TFMEMNGKLFDELTASYKLDRQQ--------------------------- 55
gi|Alligator_mississippiensis_      TFMEMNGKLFDELTASYKLEKQQ--------------------------- 449
gi|Chrysemys_picta_bellii_beta      TFMEMNGKLFDELTASYKVEKQQ--------------------------- 449
gi|Xenopus_tropicalis_beta          TFMEMNGKLFDELTASYKLDRQQ--------------------------- 447
gi|Danio_rerio_beta                 TFMEMNSKLFDDLTASYKVEKQK--------------------------- 448
gi|Ambystoma_mexicanum_beta         TFMEMNSTLFDELTASYKVEKQQ--------------------------- 318
gi|Petromyzon_marinus_S4RGA7        TFMEMNSKLFDELTASYKAERQR--------------------------- 212
gi|Strongylocentrotus_purpurat      TLMEMNSKLFDELTTSYKADRQK--------------------------- 431
gi|Drosophila_melanogaster_wdb      TFMEMNSKLFDELTSSYKAERQK--------------------------- 430
jgi|Branchiostoma_floridae_237      TFMEMNSKLFDELTANYKAERQK--------------------------- 386
gi|Hydra_vulgaris_alpha             SFMEMNSKLFDDLTASYKADRQK--------------------------- 439
jgi|Branchiostoma_floridae_112      --------------------------------------------------
jgi|Branchiostoma_floridae_284      TFMEMNSKLFDELTASYKADRQK--------------------------- 399
gi|Caenorhabditis_elegans_PPTR      TFMEMNGKLFDELTSTYKGERLR--------------------------- 460
gi|Amphimedon_queenslandica_be      AMTAMNPKLFDQLLDSYKAEKQK--------------------------- 453
gi|Chrysemys_picta_bellii_gamm      LFMEMNQKLFDDCTQQFKAEKLK--------------------------- 418
gi|Chrysemys_picta_bellii_delt      LFMEMNQKLFDDCTQQFKAEKLK--------------------------- 343
gi|Falco_peregrinus_gamma           LFMEMNQKLFDDCTQQFKAEKLK--------------------------- 418
gi|Falco_peregrinus_delta/gamm      LFMEMNQKLFDDCTQQFKAEKLK--------------------------- 343
gi|Gallus_gallus_gamma              LFMEMNQKLFDDCTQQFKAEKLK--------------------------- 418
gi|Alligator_mississippiensis_      LFMEMNQKLFDDCTQQFKAEKLK--------------------------- 418
gi|Alligator_mississippiensis_      LFMEMNQKLFDDCTQQFKAEKLK--------------------------- 343
gi|Ambystoma_mexicanum_gamma        LFMEMNQKLFDDCTQQFKAEKQK--------------------------- 419
gi|Mus_musculus_gamma               LFMEMNQKLFDDCTQQFKAEKLK--------------------------- 418
gi|Rattus_norvegicus_gamma          LFMEMNQKLFDDCTQQFKAEKLK--------------------------- 418
gi|Ovis_aries_gamma                 LFMEMNQKLFDDCTQQFKAEKLK--------------------------- 418
gi|Bos_taurus_gamma                 LFMEMNQKLFDDCTQQFKAEKLK--------------------------- 418
gi|Canis_lupus_familiaris_gamm      LFMEMNQKLFDDCTQQFKAEKLK--------------------------- 418
gi|Macaca_mulatta_gamma             LFMEMNQKLFDDCTQQFKAEKLK--------------------------- 418
gi|Homo_sapiens_gamma               LFMEMNQKLFDDCTQQFKAEKLK--------------------------- 408
gi|Felis_catus_gamma                LFMEMNQKLFDDCTQQFKAEKLK--------------------------- 465
gi|Canis_lupus_familiaris_delt      LFMEMNQKLFDDCTQQFKAEKLK--------------------------- 465
gi|Macaca_mulatta_delta/gamma       LFMEMNQKLFDDCTQQFKAEKLK--------------------------- 700
gi|Bos_taurus_delta/gamma           LFMEMNQKLFDDCTQQFKAEKLK--------------------------- 472
gi|Mus_musculus_delta/gamma         LFMEMNQKLFDDCTQQFKAEKLK--------------------------- 475
gi|Rattus_norvegicus_delta/gam      LFMEMNQKLFDDCTQQFKAEKLK--------------------------- 541
gi|Danio_rerio_gamma                LFMEMNQKLFDDCTQQFRAEKNK--------------------------- 418
gi|Danio_rerio_delta/gamma          LFMEMNQKLFDDCTQQFRAEKNK--------------------------- 471
gi|Xenopus_laevis_delta/gamma       LFMEMNQKLFDDCTQQFKAEKQK--------------------------- 471
gi|Xenopus_laevis_gamma             LFMEMNQKLFDDCTQQFKAEKQK--------------------------- 471
gi|Xenopus_tropicalis_delta/ga      LFMEMNQKLFDDCTQQFKAEKQK--------------------------- 471
gi|Mus_musculus_delta               LFMEMNQKLFDDCTQQYKAEKQK--------------------------- 486
gi|Rattus_norvegicus_delta          LFMEMNQKLFDDCTQQYKAEKQK--------------------------- 514
gi|Felis_catus_delta                LFMEMNQKLFDDCTQQYKAEKQK--------------------------- 500
gi|Bos_taurus_delta                 LFMEMNQKLFDDCTQQYKAEKQK--------------------------- 492
gi|Canis_lupus_familiaris_delt      LFMEMNQKLFDDCTQQYKAEKQK--------------------------- 487
gi|Macaca_mulatta_delta             LFMEMNQKLFDDCTQQYKAEKQK--------------------------- 503
gi|Homo_sapiens_delta               LFMEMNQKLFDDCTQQYKAEKQK--------------------------- 494
gi|Ovis_aries_delta                 LFMEMNQKLFDDCTQQYKAEKQK--------------------------- 491
gi|Falco_peregrinus_delta           LFMEMNQKLFDDCTQQYKAEKQK--------------------------- 394
gi|Gallus_gallus_delta              LFMEMNQKLFDDCTQQYKAEKQK--------------------------- 489
gi|Alligator_mississippiensis_      LFMEMNQKLFDDCTQQYKAEKQK--------------------------- 488
gi|Chrysemys_picta_bellii_delt      LFMEMNQKLFDDCTQQYKAEKQK--------------------------- 490
gi|Danio_rerio_delta                LFMEMNQKLFDDCTQQYKAEKQK--------------------------- 481
gi|Ambystoma_mexicanum_delta        LFMEMNQKLFDDCTQQYKAEKQK--------------------------- 343
gi|Petromyzon_marinus_S4RN43        LFMEMNQKLFDDCTQQYKAERQK--------------------------- 471
jgi|Branchiostoma_floridae_237      LFMEMNQKLFDDCTQRYKAERQK--------------------------- 391
jgi|Branchiostoma_floridae_252      LFMEMNQKLFDDCTQRYKAERQK--------------------------- 435
gi|Strongylocentrotus_purpurat      LFMEINQKRFDDCMQKYKDERQQ--------------------------- 452
gi|Caenorhabditis_elegans_PPTR      MFMEMNQKLFDECSQAYQKDRVQ--------------------------- 439
```

Figure S1. *Cont.*

```
gi|Hydra_vulgaris_delta             LLMEMNQKLFDQCAQQYNFQIE--------------------------- 460
gi|Amphimedon_queenslandica_de      IFAEMNQKLFDECSDKFKEELEQ-------------------------- 502
gi|Drosophila_melanogaster_B56      LFMEIDQRLFDECSKNYKQEKQM-------------------------- 575
gi|Dictyostelium_discoideum_ps      LFMEIDMDLFNKISEKYKESKKK-------------------------- 502
gi|Dictyostelium_purpureum_N/A      LFMEIDIDLFNKVSEKYKETKKK-------------------------- 459
gi|Dictyostelium_fasciculatum_      LFMEIDLETFNSISCKVMEEERA-------------------------- 473
gi|Polysphondylium_pallidum_N/      LFMDIDVETFNRISDQYKE------------------------------ 439
gi|Arabidopsis_thaliana_theta       IFNDIDAELFKDCLAKFREDESK-------------------------- 465
gi|Arabidopsis_thaliana_eta         IFHDLDPELFKECLAKFKEDESK-------------------------- 505
gi|Arabidopsis_thaliana_zeta        VLCEIDQVLFDECLAKFQVEEVN-------------------------- 495
gi|Arabidopsis_thaliana_gamma       VMAETDQILFDECLAKFQEDEAN-------------------------- 474
gi|Oryza                            LFSDRDPELYKECLRKYEENKAK-------------------------- 475
gi|Oryza_sativa_zeta                LFSDHDVGVYDECQRKYEDEKAK-------------------------- 475
gi|Oryza_sativa_theta               IFMDHDPVLFEECLKKFEEDEAK-------------------------- 479
gi|Oryza_sativa_eta                 IFSDHDSAFFGECTQKFNDDELK-------------------------- 468
gi|Arabidopsis_thaliana_delta       VLSDTNPDLFEECLHKFQEDQQK-------------------------- 453
gi|Arabidopsis_thaliana_N/A         MFCEMDEALFMSCHARFKEDEAK-------------------------- 459
gi|Oryza_sativa_kappa               MFSEMDDVLFSACLVKYKEDEER-------------------------- 465
gi|Oryza_sativa_N/A                 MFLEMDEGLLLTCQRNFQEEEEK-------------------------- 446
gi|Arabidopsis_thaliana_alpha       MFMEMDPELFEECQQQYEEKQAK-------------------------- 464
gi|Arabidopsis_thaliana_beta        MFMEMDPELFEECQRQYEEKQAK-------------------------- 464
gi|Oryza_sativa_N/A_2               MFLDMDSELFEECQQQYIEKQAR-------------------------- 504
gi|Arabidopsis_thaliana_epsilo      MFLEMDTELFEECEKQYLENEAK-------------------------- 473
gi|Chlamydomonas_reinhardtii_w      MFQELDEALYEECKRKYEEEQTS-------------------------- 462
gi|Schizosaccharomyces_pombe_P      LFMDINPSLFDEVDAEYSESRRK-------------------------- 497
gi|Schizosaccharomyces_pombe_P      IFVDMDADFFNGLVEDYKQAIIK-------------------------- 598
gi|Ashbya_gossypii_RTS1             IFLETNPVLYENCNSLYLSSLKE-------------------------- 588
gi|Saccharomyces_cerevisiae_RT      IFLETNPVLYENCNALYLSSVKE-------------------------- 693

gi|Aspergillus_niger_N/A            -----------------AEERELGRQQRWEKVLERAKDRKNGVTVPP--- 581
gi|Aspergillus_nidulans_parA        -----------------AEMREKARQNRWEKVAERAMQRQNGVNLPR--- 583
gi|Mus_musculus_alpha               -----------------EKKKELEREELWKKLEELQLK------------ 464
gi|Rattus_norvegicus_alpha          -----------------EKKKELEREELWKKLEELKLK------------ 464
gi|Macaca_mulatta_alpha             -----------------EKKKELEREELWKKLEELKLK------------ 464
gi|Homo_sapiens_alpha               -----------------EKKKELEREELWKKLEELKLK------------ 464
gi|Ovis_aries_alpha                 -----------------EKKKELEREELWKKLEELKLK------------ 467
gi|Bos_taurus_alpha                 -----------------EKKKELEREELWKKLEELKLK------------ 467
gi|Felis_catus_alpha                -----------------EKKKELEREELWKKLEELKLK------------ 380
gi|Canis_lupus_familiaris_alph      -----------------EKKKELEREELWKKLEELKLK------------ 380
gi|Falco_peregrinus_alpha           -----------------EKKKELEREELWRKLEELKLK------------ 454
gi|Gallus_gallus_alpha              -----------------EKKKELEREELWRKLEELKLK------------ 454
gi|Alligator_mississippiensis_      -----------------EKKKELEREELWRKLEELKLK------------ 464
gi|Chrysemys_picta_bellii_alph      -----------------EKKKELEREELWRKLEELKLK------------ 454
gi|Xenopus_laevis_alpha             -----------------EKKKELEREELWRRLDDLLLK------------ 453
gi|Xenopus_tropicalis_alpha         -----------------EKKKELEREELWRRLDDLLLK------------ 453
gi|Ambystoma_mexicanum_alpha        -----------------ERKKELEREELWRKLDDLKLK------------ 380
gi|Danio_rerio_alpha                -----------------EKKKEQEREELWRRLDELRMK------------ 456
gi|Petromyzon_marinus_S4RHV1        -----------------EKKKEEEREELWKKLEDLELK------------ 454
gi|Mus_musculus_epsilon             -----------------EKKKEKEREELWKKLEDLELK------------ 456
gi|Rattus_norvegicus_epsilon        -----------------EKKKEKEREELWKKLEDLELK------------ 456
gi|Macaca_mulatta_epsilon           -----------------EKKKEKEREELWKKLEDLELK------------ 456
gi|Homo_sapiens_epsilon             -----------------EKKKEKEREELWKKLEDLELK------------ 456
gi|Ovis_arie_epsilon                -----------------EKKKEKEREELWKKLEDLELK------------ 456
gi|Bos_taurus_epsilon               -----------------EKKKEKEREELWKKLEDLELK------------ 456
gi|Canis_lupus_familiaris_epsi      -----------------EKKKEKEREELWKKLEDLELK------------ 456
gi|Falco_peregrinus_epsilon         -----------------EKKKEKEREELWKKLEDLELK------------ 456
gi|Gallus_gallus_epsilon            -----------------EKKKEKEREELWKKLEDLELK------------ 456
gi|Felis_catus_epsilon              -----------------EKKKEKEREELWKKLEDLELK------------ 456
gi|Chrysemys_picta_bellii_epsi      -----------------EKKKEKEREELWKKLEDLELK------------ 451
gi|Xenopus_laevis_epsilon           -----------------EKKKEKEREELWKKLEDLELK------------ 456
```

Figure S1. *Cont.*

```
gi|Xenopus_tropicalis_epsilon        -----------------EKKKEKEREELWKKLEDLELK------------ 451
gi|Alligator_mississippiensis_       GSHKQSEGYCMSSNLVSEKKKEKEREELWKKLEDLELK------------ 489
gi|Ambystoma_mexicanum_epsilon       --------------------------------------------------
gi|Danio_rerio_epsilon               -----------------ERKKDKEREDLWRKLEDLELR------------ 456
gi|Mus_musculus_beta                 -----------------EQQKAQERQELWRGLEELRLR------------ 470
gi|Rattus_norvegicus_beta            -----------------EQQKAQERQELWRGLEELRLR------------ 470
gi|Felis_catus_beta                  -----------------EQQKARERQELWQGLEELRLR------------ 470
gi|Canis_lupus_familiaris_beta       -----------------EQQKARERQELWQGLEELRLR------------ 470
gi|Homo_sapiens_beta                 -----------------EQQKAQERQELWQGLEELRLR------------ 470
gi|Bos_taurus_beta                   -----------------EQQKARERQELWQGLEELRLR------------ 470
gi|Ovis_aries_beta                   -----------------EQQKARERQELWQGLEELRLR------------ 470
gi|Macaca_mulatta_beta               -----------------EQQKAQERQELWQGLEELRLR------------ 434
gi|Xenopus_laevis_beta               --------------------------------------------------
gi|Alligator_mississippiensis_       -----------------ELKKEKERQELWRQLDELRLR------------ 470
gi|Chrysemys_picta_bellii_beta       -----------------ELKKEKERQELWKQLDELQLK------------ 470
gi|Xenopus_tropicalis_beta           -----------------EMKREKERQELWRKLDDLRLK------------ 468
gi|Danio_rerio_beta                  -----------------ELKKERERVELWRNLEDLRER------------ 469
gi|Ambystoma_mexicanum_beta          -----------------ELKKEKERQELWKKLDDLQLR------------ 339
gi|Petromyzon_marinus_S4RGA7         -----------------ERKREKEREDLWQRLEELQLQ------------ 233
gi|Strongylocentrotus_purpurat       -----------------EKKKEKERDELWKRLSKLEMN------------ 452
gi|Drosophila_melanogaster_wdb       -----------------EKKRERDREELWKKLHELESNRSSGRTAGGSAT 463
jgi|Branchiostoma_floridae_237       -----------------ERKKEKEREELWKKLDGLEIN------------ 407
gi|Hydra_vulgaris_alpha              -----------------EKKKERDRDDLWKRLDKLKLE------------ 460
jgi|Branchiostoma_floridae_112       --------------------------------------------------
jgi|Branchiostoma_floridae_284       -----------------EKKKEKEREELWKQLDKLEIS------------ 420
gi|Caenorhabditis_elegans_PPTR       -----------------EKQREKDRDAFWKKMEALELN------------ 481
gi|Amphimedon_queenslandica_be       -----------------ELKKGKDRNELWKQLDRLKLE------------ 474
gi|Chrysemys_picta_bellii_gamm       -----------------EKLKSKEREEEAWVKIENLAKSNPQYPTYSDTSV 451
gi|Chrysemys_picta_bellii_delt       -----------------EKLKSKEREEEAWVKIENLAKSNPQYPTYSDTSV 376
gi|Falco_peregrinus_gamma            -----------------EKLKSKEREEEAWVKIENLAKSNPQYPTYSDTSL 451
gi|Falco_peregrinus_delta/gamm       -----------------EKLKSKEREEEAWVKIENLAKSNPQYPTYSDTSL 376
gi|Gallus_gallus_gamma               -----------------EKLKLKEREEEAWVKIENLAKSNPQYPTYSDTSL 451
gi|Alligator_mississippiensis_       -----------------EKLKLKEREEEAWVKIENLAKSNPQYPVYSDTSV 451
gi|Alligator_mississippiensis_       -----------------EKLKLKEREEEAWVKIENLAKSNPQYPVYSDTSV 376
gi|Ambystoma_mexicanum_gamma         -----------------EKLKLKEREEEAWVKIENLAKSNPKYSMRPDNST 452
gi|Mus_musculus_gamma                -----------------EKLKMKEREEEAWVKIENLAKANPQYAVYSQASA 451
gi|Rattus_norvegicus_gamma           -----------------EKLKMKEREEEAWVKIENLAKANPQYAVYSQASA 451
gi|Ovis_aries_gamma                  -----------------EKLKMKEREEEAWVKIENLAKANP---------- 441
gi|Bos_taurus_gamma                  -----------------EKLKMKEREEEAWVKIENLAKANPQYAVYSQAST 451
gi|Canis_lupus_familiaris_gamm       -----------------EKLKMKEREEEAWVKIENLAKANPQYAVYSQAST 451
gi|Macaca_mulatta_gamma              -----------------EKLKMKEREEEAWVKIENLAKANPQYTVYSQAST 451
gi|Homo_sapiens_gamma                -----------------EKLKMKEREEEAWVKIENLAKANPQYTVYSQAST 441
gi|Felis_catus_gamma                 -----------------EKLKMKEREEEAWVKIENLAKANP---------- 488
gi|Canis_lupus_familiaris_delt       -----------------EKLKMKEREEEAWVKIENLAKANPQYAVYSQAST 498
gi|Macaca_mulatta_delta/gamma        -----------------EKLKMKEREEEAWVKIENLAKANPQYTVYSQAST 733
gi|Bos_taurus_delta/gamma            -----------------EKLKMKEREEEAWVKIENLAKANPQYAVYSQAST 505
gi|Mus_musculus_delta/gamma          -----------------EKLKMKEREEEAWVKIENLAKANPQYAVYSQASA 508
gi|Rattus_norvegicus_delta/gam       -----------------EKLKMKEREEEAWVKIENLAKANPQYAVYSQASA 574
gi|Danio_rerio_gamma                 -----------------EKAKWKEREEEAWIKIENLAKSNPQFLMYIDANS 451
gi|Danio_rerio_delta/gamma           -----------------EKAKWKEREEEAWIKIENLAKSNPQFLMYIDANS 504
gi|Xenopus_laevis_delta/gamma        -----------------EKLKLKEREEEAWVKIENLAKSNAQSCCNGNRWA 504
gi|Xenopus_laevis_gamma              -----------------EKLKLKEREEEAWVKIENLAKSNAQSCCNGNRWA 504
gi|Xenopus_tropicalis_delta/ga       -----------------EKLKLKEREEEAWIKIENLAKSNPQYPMYTDTSA 504
gi|Mus_musculus_delta                -----------------GRFRMKEREEMWQKIEELARLNPQYPMFRAPPP 519
gi|Rattus_norvegicus_delta           -----------------GRFRMKEREEMWQKIEELARLNPQYPMFRAPPP 547
gi|Felis_catus_delta                 -----------------GRFRMKEREEMWQKIEELARLNPQYPMFRAPPP 533
gi|Bos_taurus_delta                  -----------------GRFRMKEREEMWQKIEELARLNPQYPMFRAPPP 525
gi|Canis_lupus_familiaris_delt       -----------------GRFRMKEREEMWQKIEELARLNPQYPMFRAPPP 520
gi|Macaca_mulatta_delta              -----------------GRFRMKEREEMWQKIEELARLNPQYPMFRAPPP 536
gi|Homo_sapiens_delta                -----------------GRFRMKEREEMWQKIEELARLNPQYPMFRAPPP 527
gi|Ovis_aries_delta                  -----------------GRFRMKEREEMWQKIEELARLNPQYPMFRAPPP 524
```

Figure S1. *Cont.*

```
gi|Falco_peregrinus_delta           -----------------GRFRMKEREEMWQKIEELARLNPQYPMYYAPPP 427
gi|Gallus_gallus_delta              -----------------GRFRMKEREEMWQKIEELARLNPQYPMYYAPPS 522
gi|Alligator_mississippiensis_      -----------------GRFRMKERVEMWQKIEELARLNPQYPMYYAPPP 521
gi|Chrysemys_picta_bellii_delt      -----------------GRFKLREREEMWHKVEELARQNPQYPMYYAPPP 523
gi|Danio_rerio_delta                -----------------EKYKVKEREEMWHKIEALAKQNPQSTKVQLRPG 514
gi|Ambystoma_mexicanum_delta        -----------------GKFKVKEREEMWHKIEELARLNP---------- 366
gi|Petromyzon_marinus_S4RN43        -----------------QARGQVRGGSCRTQMSTLARCDPAQDLNG---- 500
jgi|Branchiostoma_floridae_237      -----------------EKEKVREREEAWDRIEHIAQTNPMVSTANLGTC 424
jgi|Branchiostoma_floridae_252      -----------------EKEKVREREEAWDRIEHIAQTNPMVSTVNLGTC 468
gi|Strongylocentrotus_purpurat      -----------------EKDKLLKMDEIWNKLEYKARSNP---------- 475
gi|Caenorhabditis_elegans_PPTR      -----------------EKTLNEEKERIWNNIEKQAMGNPQYVEVKALFA 472
gi|Hydra_vulgaris_delta             --------------------------------------------------
gi|Amphimedon_queenslandica_de      -----------------EPKVVKKREDTWTALEKLALMSPHVSKLSSPQN 535
gi|Drosophila_melanogaster_B56      -----------------EREKLSQREELWQQVESLAKTNPEWTKARRFND 608
gi|Dictyostelium_discoideum_ps      -----------------QQQIQQ-REKFKQN--------------APETQ 520
gi|Dictyostelium_purpureum_N/A      -----------------QQQQQQQREKTRQN--------------VDVS- 477
gi|Dictyostelium_fasciculatum_      -----------------KKRQQSIMEDQRARLAHQQQQLQMQQHMQQQMQ 506
gi|Polysphondylium_pallidum_N/      ------------------RKTNKITEEKKP-------------------Q 452
gi|Arabidopsis_thaliana_theta       -----------------EAEIGAKREATWKR------------------- 479
gi|Arabidopsis_thaliana_eta         -----------------AAETEAKREATWKR------------------- 519
gi|Arabidopsis_thaliana_zeta        -----------------KTEVKAKRERTWQR------------------- 509
gi|Arabidopsis_thaliana_gamma       -----------------ETEVVAKREATWKL------------------- 488
gi|Oryza                            -----------------EKDHKLKQESVWKR------------------- 489
gi|Oryza_sativa_zeta                -----------------EKETKLKQEVAWKR------------------- 489
gi|Oryza_sativa_theta               -----------------ETALRSKREATWKR------------------- 493
gi|Oryza_sativa_eta                 -----------------QEESNSKREALWKR------------------- 482
gi|Arabidopsis_thaliana_delta       -----------------AEDTKKKNGETWRQ------------------- 467
gi|Arabidopsis_thaliana_N/A         -----------------QCSAAEKRKEVWAR------------------- 473
gi|Oryza_sativa_kappa               -----------------QASLESKRRLTWEK------------------- 479
gi|Oryza_sativa_N/A                 -----------------RAASEERRKLIWEN------------------- 460
gi|Arabidopsis_thaliana_alpha       -----------------SKQVEEQRQNRWRR------------------- 478
gi|Arabidopsis_thaliana_beta        -----------------SKEVEEQRQYTWKR------------------- 478
gi|Oryza_sativa_N/A_2               -----------------AKDMEEQRLSAWRQ------------------- 518
gi|Arabidopsis_thaliana_epsilo      -----------------ACELLEQRELTWKR------------------- 487
gi|Chlamydomonas_reinhardtii_w      -----------------ERAAMETRDRKWEY------------------- 476
gi|Schizosaccharomyces_pombe_P      -----------------KEDEEIIREERWTILENIAKEN----------- 519
gi|Schizosaccharomyces_pombe_P      -----------------QEEVMIIRKQQWCQIEALAAE------------ 619
gi|Ashbya_gossypii_RTS1             -----------------SQKRREKREENWNKLQEYVRN------------ 609
gi|Saccharomyces_cerevisiae_RT      -----------------TQQRKVQREENWSKLEEYVKN------------ 714

gi|Aspergillus_niger_N/A            --------------------------------------------------
gi|Aspergillus_nidulans_parA        --------------------------------------------------
gi|Mus_musculus_alpha               --------------------------------------------------
gi|Rattus_norvegicus_alpha          --------------------------------------------------
gi|Macaca_mulatta_alpha             --------------------------------------------------
gi|Homo_sapiens_alpha               --------------------------------------------------
gi|Ovis_aries_alpha                 --------------------------------------------------
gi|Bos_taurus_alpha                 --------------------------------------------------
gi|Felis_catus_alpha                --------------------------------------------------
gi|Canis_lupus_familiaris_alph      --------------------------------------------------
gi|Falco_peregrinus_alpha           --------------------------------------------------
gi|Gallus_gallus_alpha              --------------------------------------------------
gi|Alligator_mississippiensis_      --------------------------------------------------
gi|Chrysemys_picta_bellii_alph      --------------------------------------------------
gi|Xenopus_laevis_alpha             --------------------------------------------------
gi|Xenopus_tropicalis_alpha         --------------------------------------------------
gi|Ambystoma_mexicanum_alpha        --------------------------------------------------
gi|Danio_rerio_alpha                --------------------------------------------------
gi|Petromyzon_marinus_S4RHV1        --------------------------------------------------
gi|Mus_musculus_epsilon             --------------------------------------------------
```

Figure S1. *Cont.*

```
gi|Rattus_norvegicus_epsilon       --------------------------------------------------
gi|Macaca_mulatta_epsilon          --------------------------------------------------
gi|Homo_sapiens_epsilon            --------------------------------------------------
gi|Ovis_arie_epsilon               --------------------------------------------------
gi|Bos_taurus_epsilon              --------------------------------------------------
gi|Canis_lupus_familiaris_epsi     --------------------------------------------------
gi|Falco_peregrinus_epsilon        --------------------------------------------------
gi|Gallus_gallus_epsilon           --------------------------------------------------
gi|Felis_catus_epsilon             --------------------------------------------------
gi|Chrysemys_picta_bellii_epsi     --------------------------------------------------
gi|Xenopus_laevis_epsilon          --------------------------------------------------
gi|Xenopus_tropicalis_epsilon      --------------------------------------------------
gi|Alligator_mississippiensis_     --------------------------------------------------
gi|Ambystoma_mexicanum_epsilon     --------------------------------------------------
gi|Danio_rerio_epsilon             --------------------------------------------------
gi|Mus_musculus_beta               --------------------------------------------------
gi|Rattus_norvegicus_beta          --------------------------------------------------
gi|Felis_catus_beta                --------------------------------------------------
gi|Canis_lupus_familiaris_beta     --------------------------------------------------
gi|Homo_sapiens_beta               --------------------------------------------------
gi|Bos_taurus_beta                 --------------------------------------------------
gi|Ovis_aries_beta                 --------------------------------------------------
gi|Macaca_mulatta_beta             --------------------------------------------------
gi|Xenopus_laevis_beta             --------------------------------------------------
gi|Alligator_mississippiensis_     --------------------------------------------------
gi|Chrysemys_picta_bellii_beta     --------------------------------------------------
gi|Xenopus_tropicalis_beta         --------------------------------------------------
gi|Danio_rerio_beta                --------------------------------------------------
gi|Ambystoma_mexicanum_beta        --------------------------------------------------
gi|Petromyzon_marinus_S4RGA7       --------------------------------------------------
gi|Strongylocentrotus_purpurat     --------------------------------------------------
gi|Drosophila_melanogaster_wdb     TSNSAASAASTSLQ------------------------------------ 477
jgi|Branchiostoma_floridae_237     --------------------------------------------------
gi|Hydra_vulgaris_alpha            --------------------------------------------------
jgi|Branchiostoma_floridae_112     --------------------------------------------------
jgi|Branchiostoma_floridae_284     ---STANSKTVKAV------------------------------------ 431
gi|Caenorhabditis_elegans_PPTR     ---PPAEGKEVTPS------------------------------------ 492
gi|Amphimedon_queenslandica_be     --------------------------------------------------
gi|Chrysemys_picta_bellii_gamm     LN---------------SPVAMETDGPLIEDLQMLKKTVKEEAC------ 480
gi|Chrysemys_picta_bellii_delt     LN---------------SPVAMETDGPLIEDLQMLKKTVKEEAC------ 405
gi|Falco_peregrinus_gamma          LN---------------SPVAMETDGPLIEDLQMLKKTVKEEAC------ 480
gi|Falco_peregrinus_delta/gamm     LN---------------SPVAMETDGPLIEDLQMLKKTVKEEAC------ 405
gi|Gallus_gallus_gamma             LN---------------SPVAMETDGPLIEDLQMLKKTVKEEAC------ 480
gi|Alligator_mississippiensis_     LN---------------SPVAMETDGPLIEDLQMLKKTVKEEAC------ 480
gi|Alligator_mississippiensis_     LN---------------SPVAMETDGPLIEDLQMLKKTVKEEAC------ 405
gi|Ambystoma_mexicanum_gamma       FN---------------SQIAMETDGPLIEDVQMLKKIVKEEAS------ 481
gi|Mus_musculus_gamma              VS---------------IPVAMETDGPQFEDVQMLKKTVSDEARQVKVGR 486
gi|Rattus_norvegicus_gamma         MS---------------IPVAMETDGPQFEDVQMLKKTVSDETR------ 480
gi|Ovis_aries_gamma                --------------------------------QVLKKRVT---------- 449
gi|Bos_taurus_gamma                IS---------------IPVAMETDGPLFEDVQMLRKTVNEEAR------ 480
gi|Canis_lupus_familiaris_gamm     MS---------------IPVAMETDGPLFEDVQMLRKAVNEEAR------ 480
gi|Macaca_mulatta_gamma            VS---------------IPVAMETDGPLFEDVQMLRKTVKDEAH------ 480
gi|Homo_sapiens_gamma              MS---------------IPVAMETDGPLFEDVQMLRKTVKDEAH------ 470
gi|Felis_catus_gamma               --------------------------------------------------
gi|Canis_lupus_familiaris_delt     MS---------------IPVAMETDGPLFEDVQMLRKAVNEEA------- 526
gi|Macaca_mulatta_delta/gamma      VS---------------IPVAMETDGPLFEDVQMLRKTVKDEA------- 761
gi|Bos_taurus_delta/gamma          IS---------------IPVAMETDGPLFEDVQMLRKTVNEEA------- 533
gi|Mus_musculus_delta/gamma        VS---------------IPVAMETDGPQFEDVQMLKKTVSDEARQVKVGR 543
gi|Rattus_norvegicus_delta/gam     MS---------------IPVAMETDGPQFEDVQMLKKTVSDET------- 602
gi|Danio_rerio_gamma               LC---------------SPMDMETDGPMLEDVLMLKKTVEEEAT------ 480
gi|Danio_rerio_delta/gamma         LC---------------SPMDMETDGPMLEDVLMLKKTVEEEAT------ 533
```

Figure S1. *Cont.*

```
gi|Xenopus_laevis_delta/gamma      LT------------------------------------------------ 506
gi|Xenopus_laevis_gamma            LT------------------------------------------------ 506
gi|Xenopus_tropicalis_delta/ga     LN---------------SPVAMETDGPLLEDVQMLKKTVKEEAS------ 533
gi|Mus_musculus_delta              LP---------------PVYSMETETPTAEDIQLLKRTVETEAV------ 548
gi|Rattus_norvegicus_delta         LP---------------PVYSMETETPTAEDIQLLKRTVETEAV------ 576
gi|Felis_catus_delta               LP---------------PVYSMETETPTAEDIQLLKRTVETEAV------ 562
gi|Bos_taurus_delta                LP---------------PVYSMETETPTAEDIQLLKRTVETEAV------ 554
gi|Canis_lupus_familiaris_delt     LP---------------PVYSMETETPTAEDIQLLKRTVETEAV------ 549
gi|Macaca_mulatta_delta            LP---------------PVYSMETETPTAEDIQLLKRTVETEAV------ 565
gi|Homo_sapiens_delta              LP---------------PVYSMETETPTAEDIQLLKRTVETEAV------ 556
gi|Ovis_aries_delta                LP---------------PVYSMETETPTAEDIQLLKRTVETEAV------ 553
gi|Falco_peregrinus_delta          LP---------------SVCCMETETPTAEDIQLLKKTVETEAV------ 456
gi|Gallus_gallus_delta             LP---------------LVCCMETETPTAEDIQLLKKTVETEAV------ 551
gi|Alligator_mississippiensis_     LP---------------PVYCMETETPTAEDIQLLKKTMETEAV------ 550
gi|Chrysemys_picta_bellii_delt     LP---------------PVYCMETETPTAEDIQLLKKTVETEAV------ 552
gi|Danio_rerio_delta               LAQEEYMMYNEGGM---PIYSMETETPTVEDIQLLKKTVESEAS------ 555
gi|Ambystoma_mexicanum_delta       --------------------------------------------------
gi|Petromyzon_marinus_S4RN43       -----------------AVVSMETGTPSSEDIMLLKKSMETEAV------ 527
jgi|Branchiostoma_floridae_237     SSCVGG------------SVAMETDANDDAELSLDKMNERG--------- 453
jgi|Branchiostoma_floridae_252     SSCVGCEW---------LSLPSLVLASLQMELPFLRSCEVLTCCVV---- 505
gi|Strongylocentrotus_purpurat     ------------------LAAEGVPGGRKESKPLLR-------------- 493
gi|Caenorhabditis_elegans_PPTR     RFNPDEII--------SSRQQNGVDENMKTSTVLSKDEILKNAV------ 508
gi|Hydra_vulgaris_delta            --------------------------------------------------
gi|Amphimedon_queenslandica_de     AFRKSMLSSLTSIASEEPTSPDGVPTSEELKSIPQTPPSESGAS------ 579
gi|Drosophila_melanogaster_B56     CL------------------------PVSDSRALCDQYSENSDS------ 628
gi|Dictyostelium_discoideum_ps     KSKQINQNNNNNNNN----------------------------------- 535
gi|Dictyostelium_purpureum_N/A     -FKQINQKE----------------------------------------- 485
gi|Dictyostelium_fasciculatum_     QQQQLQQQMQ---------------------------------------- 516
gi|Polysphondylium_pallidum_N/     QPRPITSNIS---------------------------------------- 462
gi|Arabidopsis_thaliana_theta      --------------------------------------------------
gi|Arabidopsis_thaliana_eta        --------------------------------------------------
gi|Arabidopsis_thaliana_zeta       --------------------------------------------------
gi|Arabidopsis_thaliana_gamma      --------------------------------------------------
gi|Oryza                           --------------------------------------------------
gi|Oryza_sativa_zeta               --------------------------------------------------
gi|Oryza_sativa_theta              --------------------------------------------------
gi|Oryza_sativa_eta                --------------------------------------------------
gi|Arabidopsis_thaliana_delta      --------------------------------------------------
gi|Arabidopsis_thaliana_N/A        --------------------------------------------------
gi|Oryza_sativa_kappa              --------------------------------------------------
gi|Oryza_sativa_N/A                --------------------------------------------------
gi|Arabidopsis_thaliana_alpha      --------------------------------------------------
gi|Arabidopsis_thaliana_beta       --------------------------------------------------
gi|Oryza_sativa_N/A_2              --------------------------------------------------
gi|Arabidopsis_thaliana_epsilo     --------------------------------------------------
gi|Chlamydomonas_reinhardtii_w     --------------------------------------------------
gi|Schizosaccharomyces_pombe_P     --------------------------------------------------
gi|Schizosaccharomyces_pombe_P     --------------------------------------------------
gi|Ashbya_gossypii_RTS1            --------------------------------------------------
gi|Saccharomyces_cerevisiae_RT     --------------------------------------------------


gi|Aspergillus_niger_N/A           ---------------------QCQVAEIPVHLDDIDQVAHESQKRLHALK 610
gi|Aspergillus_nidulans_parA       ---------------------NSTTAEIPLQLDDVDALTQESQRRLQSLK 612
gi|Mus_musculus_alpha              ----------------------KALE-KQNNAYNMHSIRSSTSAK----- 486
gi|Rattus_norvegicus_alpha         ----------------------KALE-KQNNAYNMHSILSNTSAQ----- 486
gi|Macaca_mulatta_alpha            ----------------------KALE-KQNSAYNMHSILSNTSAE----- 486
gi|Homo_sapiens_alpha              ----------------------KALE-KQNSAYNMHSILSNTSAE----- 486
gi|Ovis_aries_alpha                ----------------------KALE-KQNSAYNMHSILSNTNDG----- 489
gi|Bos_taurus_alpha                ----------------------KALE-KQNSAYNMHSILSNTNDG----- 489
gi|Felis_catus_alpha               ----------------------KALE-KQNSAYNMHSILSNTSDE----- 402
```

Figure S1. *Cont.*

```
gi|Canis_lupus_familiaris_alph       ----------------------KALE-KQNSAYNMHSILSNTSDE----- 402
gi|Falco_peregrinus_alpha            ----------------------KAMAEKQNSTHNVLNAHN-TSAK----- 476
gi|Gallus_gallus_alpha               ----------------------KAMAEKQNSTHNVLNAHN-TSAK----- 476
gi|Alligator_mississippiensis_       ----------------------KVLAEKQNSTHNVLNAQNNTSAK----- 487
gi|Chrysemys_picta_bellii_alph       ----------------------KTLVEKQNSTHNVLNAHNSTSAK----- 477
gi|Xenopus_laevis_alpha              ----------------------KALADKQNPTLCVQNIQNNASAK----- 476
gi|Xenopus_tropicalis_alpha          ----------------------KTLADKQNPALCVQNIQNNASAK----- 476
gi|Ambystoma_mexicanum_alpha         ----------------------KALAEKQKFHHNVQNAQKNTIAK----- 403
gi|Danio_rerio_alpha                 ----------------------EVLM-IQNNRHDIHSLTTTTSIKNNEHD 483
gi|Petromyzon_marinus_S4RHV1         ----------------------NFQN----------NILVK--------- 463
gi|Mus_musculus_epsilon              ----------------------RGLR--------RDGIIPT--------- 467
gi|Rattus_norvegicus_epsilon         ----------------------RGLR--------RDGIIPT--------- 467
gi|Macaca_mulatta_epsilon            ----------------------RGLR--------RDGIIPT--------- 467
gi|Homo_sapiens_epsilon              ----------------------RGLR--------RDGIIPT--------- 467
gi|Ovis_arie_epsilon                 ----------------------RGLR--------RDGIIPT--------- 467
gi|Bos_taurus_epsilon                ----------------------RGLR--------RDGIIPT--------- 467
gi|Canis_lupus_familiaris_epsi       ----------------------RGLR--------RDGIIPT--------- 467
gi|Falco_peregrinus_epsilon          ----------------------RGLR--------RDGIIPT--------- 467
gi|Gallus_gallus_epsilon             ----------------------RGLR--------RDGIIPT--------- 467
gi|Felis_catus_epsilon               ----------------------RGLR--------RDGIIPT--------- 467
gi|Chrysemys_picta_bellii_epsi       ----------------------RGLR--------RDGIIPT--------- 462
gi|Xenopus_laevis_epsilon            ----------------------RGLR--------SDGIIPT--------- 467
gi|Xenopus_tropicalis_epsilon        ----------------------RGLR--------SDGIIPT--------- 462
gi|Alligator_mississippiensis_       ----------------------RGLR--------RDGIIPT--------- 500
gi|Ambystoma_mexicanum_epsilon       --------------------------------------------------
gi|Danio_rerio_epsilon               ----------------------RGIQN-------SDGVIPT--------- 468
gi|Mus_musculus_beta                 ----------------------RLQGTQGAKEAPVPRPTPQVAASGGQS- 497
gi|Rattus_norvegicus_beta            ----------------------RLQGTQGAKEAPVPRPTPQVAASGGQS- 497
gi|Felis_catus_beta                  ----------------------RLQGTQGAKEAPLQRLTPQVATSGGQS- 497
gi|Canis_lupus_familiaris_beta       ----------------------RLQGTQGAKETPLQRLTPQVATSGGQS- 497
gi|Homo_sapiens_beta                 ----------------------RLQGTQGAKEAPLQRLTPQVAASGGQS- 497
gi|Bos_taurus_beta                   ----------------------RLQGTQGAKEAPLQRLTPQVTG-GGQS- 496
gi|Ovis_aries_beta                   ----------------------RLQGTQGAKEAPLQRLTPQVTG-GGQS- 496
gi|Macaca_mulatta_beta               ----------------------RLQGTQGAKEAPLQRLTPQVATSGGQS- 461
gi|Xenopus_laevis_beta               --------------------------------------------------
gi|Alligator_mississippiensis_       ----------------------RLRGLEEAQMNRLNLQHGHSPKT----- 493
gi|Chrysemys_picta_bellii_beta       ----------------------KLQGLEEAQMNRLNLQHSLGLQSGNKS- 497
gi|Xenopus_tropicalis_beta           ----------------------KMNGLEEAQMNQLNLQHSRGSKS----- 491
gi|Danio_rerio_beta                  ----------------------RLQSLTEASRNQKNLQERENTAKSQSDT 497
gi|Ambystoma_mexicanum_beta          ----------------------RLRSLEEAQMNRLNLQNSLSMQSSSKS- 366
gi|Petromyzon_marinus_S4RGA7         ----------------------QSPNNNSARAAGTS-------------- 247
gi|Strongylocentrotus_purpurat       --------------------------HLN---NQTNSS------------ 461
gi|Drosophila_melanogaster_wdb       ---------------------PPSSAGLNSHQQQSNSGSSGSLSSGGAGG 506
jgi|Branchiostoma_floridae_237       --------------------------------------------------
gi|Hydra_vulgaris_alpha              --------------------------------KTGNSSEPGT-------- 470
jgi|Branchiostoma_floridae_112       --------------------------------------------------
jgi|Branchiostoma_floridae_284       ---------------------PPMSPNSAPPGDRGANYSPYSPTAHRLMA 460
gi|Caenorhabditis_elegans_PPTR       ---------------------LFPEKLTDYLKKDGPNMTPLPVATAGGGD 521
gi|Amphimedon_queenslandica_be       --------------------------------HDMKLAKK---------- 482
gi|Chrysemys_picta_bellii_gamm       -------------------QAQRDQKK-DRPLMRRKSELPQDIYTMKALE 510
gi|Chrysemys_picta_bellii_delt       -------------------QAQRDQKK-DRPLMRRKSELPQDIYTMKALE 435
gi|Falco_peregrinus_gamma            -------------------QAQKDQKK-DRPLVRRKSELPQDIYTMKALE 510
gi|Falco_peregrinus_delta/gamm       -------------------QAQKDQKK-DRPLVRRKSELPQDIYTMKALE 435
gi|Gallus_gallus_gamma               -------------------QAQKDQKK-DRPLVRRKSELPQDIYTMKALE 510
gi|Alligator_mississippiensis_       -------------------QAQKDQKK-DRPLVRRKSELPQDIYTMKALE 510
gi|Alligator_mississippiensis_       -------------------QAQKDQKK-DRPLVRRKSELPQDIYTMKALE 435
gi|Ambystoma_mexicanum_gamma         -------------------LALKDIKK-ERPIVRRKSELPQDIYTMKALE 511
gi|Mus_musculus_gamma                EKSHDSHASQEGVETDWAAQAQKELKK-DRPLVRRKSELPQDPHTEKALE 535
gi|Rattus_norvegicus_gamma           -------------------QAQKDLKK-DRPLVRRKSELPQDPHTEKALE 510
gi|Ovis_aries_gamma                  --------------------------------------------------
gi|Bos_taurus_gamma                  -------------------QAQKDPKK-ERPLARRKSELPQDLHTKSALE 510
```

Figure S1. *Cont.*

```
gi|Canis_lupus_familiaris_gamm      -------------------QAQKDLKK-DRPGVRRKPELPQDPHTKNALE 510
gi|Macaca_mulatta_gamma             -------------------QAQKDPKK-DRPLARRKSELPQDPHTKKALE 510
gi|Homo_sapiens_gamma               -------------------QAQKDPKK-DRPLALRKSELPQDPHTKKALE 500
gi|Felis_catus_gamma                -------------------QAQKDLKK-DRPLVRRKSELPQDLHTKNALE 518
gi|Canis_lupus_familiaris_delt      ------------------RQAQKDLKK-DRPGVRRKPELPQDPHTKNALE 557
gi|Macaca_mulatta_delta/gamma       ------------------HQAQKDPKK-DRPLARRKSELPQDPHTKKALE 792
gi|Bos_taurus_delta/gamma           ------------------RQAQKDPKK-ERPLARRKSELPQDLHTKSALE 564
gi|Mus_musculus_delta/gamma         EKSHDSHASQEGVETDWAAQAQKELKK-DRPLVRRKSELPQDPHTEKALE 592
gi|Rattus_norvegicus_delta/gam      ------------------RQAQKDLKK-DRPLVRRKSELPQDPHTEKALE 633
gi|Danio_rerio_gamma                -------------------PLHREQRK-ERPLMRRKSELPQDTSTVKALE 510
gi|Danio_rerio_delta/gamma          -------------------PLHREQRK-ERPLMRRKSELPQDTSTVKALE 563
gi|Xenopus_laevis_delta/gamma       --------------------------------------------------
gi|Xenopus_laevis_gamma             --------------------------------------------------
gi|Xenopus_tropicalis_delta/ga      -------------------QAQKDLKK-ERPLVRRKSELPQDIYTMKALE 563
gi|Mus_musculus_delta               -------------------QMLKDIKK-DKVLLRRKSELPQDVYTIKALE 578
gi|Rattus_norvegicus_delta          -------------------QMLKDIKK-DKVLLRRKSELPQDVYTIKALE 606
gi|Felis_catus_delta                -------------------QMLKDIKK-EKVLLRRKSELPQDVYTIKALE 592
gi|Bos_taurus_delta                 -------------------QMLKDIKK-EKVLLRRKSELPQDVYTIKALE 584
gi|Canis_lupus_familiaris_delt      -------------------QMLKDIKK-EKVLLRRKSELPQDVYTIKALE 579
gi|Macaca_mulatta_delta             -------------------QMLKDIKK-EKVLLRRKSELPQDVYTIKALE 595
gi|Homo_sapiens_delta               -------------------QMLKDIKK-EKVLLRRKSELPQDVYTIKALE 586
gi|Ovis_aries_delta                 -------------------QMLKDIKK-EKVLLRRKSELPQDVYTIKALE 583
gi|Falco_peregrinus_delta           -------------------QMLKDIKK-EKALLRRKSELPQDVYTIKALE 486
gi|Gallus_gallus_delta              -------------------QMLKDIKK-EKVLLRRKSELPQDVYTIKALE 581
gi|Alligator_mississippiensis_      -------------------QMLKDIKK-EKVLLHRKSELPQDVYTIKALE 580
gi|Chrysemys_picta_bellii_delt      -------------------QMLKDIKK-EKVLLRRKSELPQDVYTIKALE 582
gi|Danio_rerio_delta                -------------------QGMKEIKK-DKVLMRRKSELPQDVYTIKALE 585
gi|Ambystoma_mexicanum_delta        --------------------------------------------------
gi|Petromyzon_marinus_S4RN43        -------------------QVQNSKGEGKKVLLRSKSELPQEMSTMRALE 558
jgi|Branchiostoma_floridae_237      ------------------QAGVSRGKDLKKMLPRRKSELPHDSLTMRALS 485
jgi|Branchiostoma_floridae_252      ----------------FPQAGVSRGKDLKKMLPRRKSELPHDSLTMRALS 539
gi|Strongylocentrotus_purpurat      ----------------------------------RKSELPRDAFTIKAIG 509
gi|Caenorhabditis_elegans_PPTR      ------------------GVSSMK-NDMDFGPNHKQSDFPPDEQTTRALG 539
gi|Hydra_vulgaris_delta             --------------------------------------------------
gi|Amphimedon_queenslandica_de      ------------------NTGAVKTNNKGHMLIRRKSYLPMDNDTSQALQ 611
gi|Drosophila_melanogaster_B56      -----------------------AYDQSEQRARQPPPPLPPQKQAHQEPR 655
gi|Dictyostelium_discoideum_ps      -INNNNNNNNNNNGSTETKADKP-------SMIRRKSLLPVDPSTIAALS 577
gi|Dictyostelium_purpureum_N/A      ---------------IETKSDKP-------SMIRRKSLLPVDPSTIAALS 513
gi|Dictyostelium_fasciculatum_      --------------QHMQQQQQPQTQPSKASMIRRKSLLPVDPSTIAALS 552
gi|Polysphondylium_pallidum_N/      --------------ALPENGEKP------ISMIRRKSLLPVDPSTIAALS 492
gi|Arabidopsis_thaliana_theta       -------------------------------LEEIGNQKQ--KSSL---- 492
gi|Arabidopsis_thaliana_eta         -------------------------------LEELGVRKA--S------- 529
gi|Arabidopsis_thaliana_zeta        -------------------------------LEDLATSKTVVTNEAVLVP 528
gi|Arabidopsis_thaliana_gamma       -------------------------------LEELAASKS-VSNEAVLVP 506
gi|Oryza                            -------------------------------LEEVASAKA-TSGEAVLIS 507
gi|Oryza_sativa_zeta                -------------------------------LEEMASAKA-TSGAAVLVS 507
gi|Oryza_sativa_theta               -------------------------------LEEIASSKT-ISSEPAVPP 511
gi|Oryza_sativa_eta                 -------------------------------LEEAAVPRS-DNNNPVGTP 500
gi|Arabidopsis_thaliana_delta       -------------------------------LEEIVASMAK--------- 477
gi|Arabidopsis_thaliana_N/A         -------------------------------LENAASMKPITGKTAVLVT 492
gi|Oryza_sativa_kappa               -------------------------------LESAASFQPVTGHTAVLVG 498
gi|Oryza_sativa_N/A                 -------------------------------LERNASFRPVTGDIGFSVL 479
gi|Arabidopsis_thaliana_alpha       -------------------------------LDEAVEERE----REDPMI 493
gi|Arabidopsis_thaliana_beta        -------------------------------LAEAAAERDGGGGEEDHMI 497
gi|Oryza_sativa_N/A_2               -------------------------------LEAAAAKAS----GDDMVL 533
gi|Arabidopsis_thaliana_epsilo      -------------------------------LEEAASLAAN--------- 497
gi|Chlamydomonas_reinhardtii_w      -------------------------------LQKLAVQKSNGRPLPEVKP 495
gi|Schizosaccharomyces_pombe_P      -------------------------------AMKLKSQNPTTVHSTTERL 538
gi|Schizosaccharomyces_pombe_P      -------------------------------------------NKPTDYL 626
gi|Ashbya_gossypii_RTS1             -----------------------LHIS------SVDNPVAVDRIGTGDLH 630
gi|Saccharomyces_cerevisiae_RT      -----------------------LRINNDKDQYTIKNPELRNSFNTASEN 741
```

Figure S1. *Cont.*

```
gi|Aspergillus_niger_N/A            LDDSSSTERRPRESTASSVSTERPE------------------------- 635
gi|Aspergillus_nidulans_parA        LDEAGSKDRRPREGSITSP------------------------------- 631
gi|Mus_musculus_alpha               --------------------------------------------------
gi|Rattus_norvegicus_alpha          --------------------------------------------------
gi|Macaca_mulatta_alpha             --------------------------------------------------
gi|Homo_sapiens_alpha               --------------------------------------------------
gi|Ovis_aries_alpha                 --------------------------------------------------
gi|Bos_taurus_alpha                 --------------------------------------------------
gi|Felis_catus_alpha                --------------------------------------------------
gi|Canis_lupus_familiaris_alph      --------------------------------------------------
gi|Falco_peregrinus_alpha           --------------------------------------------------
gi|Gallus_gallus_alpha              --------------------------------------------------
gi|Alligator_mississippiensis_      --------------------------------------------------
gi|Chrysemys_picta_bellii_alph      --------------------------------------------------
gi|Xenopus_laevis_alpha             --------------------------------------------------
gi|Xenopus_tropicalis_alpha         --------------------------------------------------
gi|Ambystoma_mexicanum_alpha        --------------------------------------------------
gi|Danio_rerio_alpha                I------------------------------------------------- 484
gi|Petromyzon_marinus_S4RHV1        --------------------------------------------------
gi|Mus_musculus_epsilon             --------------------------------------------------
gi|Rattus_norvegicus_epsilon        --------------------------------------------------
gi|Macaca_mulatta_epsilon           --------------------------------------------------
gi|Homo_sapiens_epsilon             --------------------------------------------------
gi|Ovis_arie_epsilon                --------------------------------------------------
gi|Bos_taurus_epsilon               --------------------------------------------------
gi|Canis_lupus_familiaris_epsi      --------------------------------------------------
gi|Falco_peregrinus_epsilon         --------------------------------------------------
gi|Gallus_gallus_epsilon            --------------------------------------------------
gi|Felis_catus_epsilon              --------------------------------------------------
gi|Chrysemys_picta_bellii_epsi      --------------------------------------------------
gi|Xenopus_laevis_epsilon           --------------------------------------------------
gi|Xenopus_tropicalis_epsilon       --------------------------------------------------
gi|Alligator_mississippiensis_      --------------------------------------------------
gi|Ambystoma_mexicanum_epsilon      --------------------------------------------------
gi|Danio_rerio_epsilon              --------------------------------------------------
gi|Mus_musculus_beta                --------------------------------------------------
gi|Rattus_norvegicus_beta           --------------------------------------------------
gi|Felis_catus_beta                 --------------------------------------------------
gi|Canis_lupus_familiaris_beta      --------------------------------------------------
gi|Homo_sapiens_beta                --------------------------------------------------
gi|Bos_taurus_beta                  --------------------------------------------------
gi|Ovis_aries_beta                  --------------------------------------------------
gi|Macaca_mulatta_beta              --------------------------------------------------
gi|Xenopus_laevis_beta              --------------------------------------------------
gi|Alligator_mississippiensis_      --------------------------------------------------
gi|Chrysemys_picta_bellii_beta      --------------------------------------------------
gi|Xenopus_tropicalis_beta          --------------------------------------------------
gi|Danio_rerio_beta                 SAANTT-------------------------------------------- 503
gi|Ambystoma_mexicanum_beta         --------------------------------------------------
gi|Petromyzon_marinus_S4RGA7        --------------------------------------------------
gi|Strongylocentrotus_purpurat      --------------------------------------------------
gi|Drosophila_melanogaster_wdb      DNNPATTNAKIKQDKADN-------------------------------- 524
jgi|Branchiostoma_floridae_237      --------------------------------------------------
gi|Hydra_vulgaris_alpha             --------------------------------------------------
jgi|Branchiostoma_floridae_112      --------------------------------------------------
jgi|Branchiostoma_floridae_284      K------------------------------------------------- 461
gi|Caenorhabditis_elegans_PPTR      KSPSVVKKSSTGSETTTPAKK----------------------------- 542
gi|Amphimedon_queenslandica_be      --------------------------------------------------
gi|Chrysemys_picta_bellii_gamm      SHCRADELI-SHDGH----------------------------------- 524
```

Figure S1. *Cont.*

```
gi|Chrysemys_picta_bellii_delt      SHCRADELI-SHDGH----------------------------------- 449
gi|Falco_peregrinus_gamma           SHCRADELI-SHDGQ----------------------------------- 524
gi|Falco_peregrinus_delta/gamm      SHCRADELI-SHDGQ----------------------------------- 449
gi|Gallus_gallus_gamma              SHCRADELI-SHDGH----------------------------------- 524
gi|Alligator_mississippiensis_      SHCRADELI-SHDGH----------------------------------- 524
gi|Alligator_mississippiensis_      SHCRADELI-SHDGH----------------------------------- 449
gi|Ambystoma_mexicanum_gamma        SHCRADELL-THDAH----------------------------------- 525
gi|Mus_musculus_gamma               AHCRASELL-SQDGR----------------------------------- 549
gi|Rattus_norvegicus_gamma          AHCRASELL-SQDGR----------------------------------- 524
gi|Ovis_aries_gamma                 --------------------------------------------------
gi|Bos_taurus_gamma                 AHGRAEEPA-PQDGR----------------------------------- 524
gi|Canis_lupus_familiaris_gamm      AHCRADELV-SQDGR----------------------------------- 524
gi|Macaca_mulatta_gamma             AHCRADELA-SQDGR----------------------------------- 524
gi|Homo_sapiens_gamma               AHCRADELA-SQDGR----------------------------------- 514
gi|Felis_catus_gamma                AHCRADQLV-SQDGR----------------------------------- 532
gi|Canis_lupus_familiaris_delt      AHCRADELV-SQDGR----------------------------------- 571
gi|Macaca_mulatta_delta/gamma       AHCRADELA-SQDGR----------------------------------- 806
gi|Bos_taurus_delta/gamma           AHGRAEEPA-PQDGR----------------------------------- 578
gi|Mus_musculus_delta/gamma         AHCRASELL-SQDGR----------------------------------- 606
gi|Rattus_norvegicus_delta/gam      AHCRASELL-SQDGR----------------------------------- 647
gi|Danio_rerio_gamma                THRRAEDMIGTQDGH----------------------------------- 525
gi|Danio_rerio_delta/gamma          THRRAEDMIGTQDGH----------------------------------- 578
gi|Xenopus_laevis_delta/gamma       --------------------------------------------------
gi|Xenopus_laevis_gamma             --------------------------------------------------
gi|Xenopus_tropicalis_delta/ga      SHCRADELISHDGH------------------------------------ 577
gi|Mus_musculus_delta               AHKRAEEFLTASQEAL---------------------------------- 594
gi|Rattus_norvegicus_delta          AHKRAEEFLTASQEAL---------------------------------- 622
gi|Felis_catus_delta                AHKRAEEFLTASQEAL---------------------------------- 608
gi|Bos_taurus_delta                 AHKRAEEFLTASQEAL---------------------------------- 600
gi|Canis_lupus_familiaris_delt      AHKRAEEFLTASQEAL---------------------------------- 595
gi|Macaca_mulatta_delta             AHKRAEEFLTASQEAL---------------------------------- 611
gi|Homo_sapiens_delta               AHKRAEEFLTASQEAL---------------------------------- 602
gi|Ovis_aries_delta                 AHKRAEEFLTASQEAL---------------------------------- 599
gi|Falco_peregrinus_delta           AHKRAEEFLTSSQEAL---------------------------------- 502
gi|Gallus_gallus_delta              AHKRAEEFLTSNQEAL---------------------------------- 597
gi|Alligator_mississippiensis_      AHKRAEEFLTSSQEAL---------------------------------- 596
gi|Chrysemys_picta_bellii_delt      AHKRAEEFLTSSQEAL---------------------------------- 598
gi|Danio_rerio_delta                AHKRAEEYLTANQEAL---------------------------------- 601
gi|Ambystoma_mexicanum_delta        --------------------------------------------------
gi|Petromyzon_marinus_S4RN43        VHRRAEEYLSTSHE------------------------------------ 572
jgi|Branchiostoma_floridae_237      DHKRADDYLTTSPETS---------------------------------- 501
jgi|Branchiostoma_floridae_252      DHKRADDYLTTSPETS---------------------------------- 555
gi|Strongylocentrotus_purpurat      THKRHEEFLVASPETNS--------------------------------- 526
gi|Caenorhabditis_elegans_PPTR      EYKRHDPFLKKVTSTDEQ-------------------------------- 557
gi|Hydra_vulgaris_delta             --------------------------------------------------
gi|Amphimedon_queenslandica_de      QYKPHERLGVPNES------------------------------------ 625
gi|Drosophila_melanogaster_B56      EVRQALATLTTLNNY----------------------------------- 670
gi|Dictyostelium_discoideum_ps      SHRSLEDIMSTNSNS----GNDDDDENNHTNHDSEIENEVKEDFRVPVNN 623
gi|Dictyostelium_purpureum_N/A      NHRSLEEIISNKNEN----SNDNNDDE-----------EVKEDSLHYVPY 548
gi|Dictyostelium_fasciculatum_      SHRSLEDIVHHSSDSEMNCTDDNNADQQPNN-------DYNGDHKTSTSK 595
gi|Polysphondylium_pallidum_N/      NHRSLEGLVHSGDG-------DHDAEETEML-------YDSSESKVPVAI 528
gi|Arabidopsis_thaliana_theta       --------------------------------------------------
gi|Arabidopsis_thaliana_eta         --------------------------------------------------
gi|Arabidopsis_thaliana_zeta        RFVSSVNLTTSSSESTGS-------------------------------- 546
gi|Arabidopsis_thaliana_gamma       RFSSSVTLATG--KTSGS-------------------------------- 522
gi|Oryza                            PSLARTSSLV---------------------------------------- 517
gi|Oryza_sativa_zeta                RTLPRQSSAV---------------------------------------- 517
gi|Oryza_sativa_theta               EATVH--------------------------------------------- 516
gi|Oryza_sativa_eta                 NGKFSQAAG----------------------------------------- 509
gi|Arabidopsis_thaliana_delta       --------------------------------------------------
gi|Arabidopsis_thaliana_N/A         --PRATSIAC---------------------------------------- 500
```

Figure S1. *Cont.*

```
gi|Oryza_sativa_kappa               RQPSANLIATLI-------------------------------------- 510
gi|Oryza_sativa_N/A                 P-ASAPLVAPTMT------------------------------------- 491
gi|Arabidopsis_thaliana_alpha       TS------------------------------------------------ 495
gi|Arabidopsis_thaliana_beta        TS------------------------------------------------ 499
gi|Oryza_sativa_N/A_2               VN------------------------------------------------ 535
gi|Arabidopsis_thaliana_epsilo      --------------------------------------------------
gi|Chlamydomonas_reinhardtii_w      LSALLRV------------------------------------------- 502
gi|Schizosaccharomyces_pombe_P      KKLSLDYTNG---------------------------------------- 548
gi|Schizosaccharomyces_pombe_P      R------------------------------------------------- 627
gi|Ashbya_gossypii_RTS1             --------------------------------------------------
gi|Saccharomyces_cerevisiae_RT      NTLNEENENDCDSEIQ---------------------------------- 757


gi|Aspergillus_niger_N/A            -----
gi|Aspergillus_nidulans_parA        -----
gi|Mus_musculus_alpha               -----
gi|Rattus_norvegicus_alpha          -----
gi|Macaca_mulatta_alpha             -----
gi|Homo_sapiens_alpha               -----
gi|Ovis_aries_alpha                 -----
gi|Bos_taurus_alpha                 -----
gi|Felis_catus_alpha                -----
gi|Canis_lupus_familiaris_alph      -----
gi|Falco_peregrinus_alpha           -----
gi|Gallus_gallus_alpha              -----
gi|Alligator_mississippiensis_      -----
gi|Chrysemys_picta_bellii_alph      -----
gi|Xenopus_laevis_alpha             -----
gi|Xenopus_tropicalis_alpha         -----
gi|Ambystoma_mexicanum_alpha        -----
gi|Danio_rerio_alpha                -----
gi|Petromyzon_marinus_S4RHV1        -----
gi|Mus_musculus_epsilon             -----
gi|Rattus_norvegicus_epsilon        -----
gi|Macaca_mulatta_epsilon           -----
gi|Homo_sapiens_epsilon             -----
gi|Ovis_arie_epsilon                -----
gi|Bos_taurus_epsilon               -----
gi|Canis_lupus_familiaris_epsi      -----
gi|Falco_peregrinus_epsilon         -----
gi|Gallus_gallus_epsilon            -----
gi|Felis_catus_epsilon              -----
gi|Chrysemys_picta_bellii_epsi      -----
gi|Xenopus_laevis_epsilon           -----
gi|Xenopus_tropicalis_epsilon       -----
gi|Alligator_mississippiensis_      -----
gi|Ambystoma_mexicanum_epsilon      -----
gi|Danio_rerio_epsilon              -----
gi|Mus_musculus_beta                -----
gi|Rattus_norvegicus_beta           -----
gi|Felis_catus_beta                 -----
gi|Canis_lupus_familiaris_beta      -----
gi|Homo_sapiens_beta                -----
gi|Bos_taurus_beta                  -----
gi|Ovis_aries_beta                  -----
gi|Macaca_mulatta_beta              -----
gi|Xenopus_laevis_beta              -----
gi|Alligator_mississippiensis_      -----
gi|Chrysemys_picta_bellii_beta      -----
gi|Xenopus_tropicalis_beta          -----
gi|Danio_rerio_beta                 -----
```

Figure S1. *Cont.*

```
gi|Ambystoma_mexicanum_beta         -----
gi|Petromyzon_marinus_S4RGA7        -----
gi|Strongylocentrotus_purpurat      -----
gi|Drosophila_melanogaster_wdb      -----
jgi|Branchiostoma_floridae_237      -----
gi|Hydra_vulgaris_alpha             -----
jgi|Branchiostoma_floridae_112      -----
jgi|Branchiostoma_floridae_284      -----
gi|Caenorhabditis_elegans_PPTR      -----
gi|Amphimedon_queenslandica_be      -----
gi|Chrysemys_picta_bellii_gamm      -----
gi|Chrysemys_picta_bellii_delt      -----
gi|Falco_peregrinus_gamma           -----
gi|Falco_peregrinus_delta/gamm      -----
gi|Gallus_gallus_gamma              -----
gi|Alligator_mississippiensis_      -----
gi|Alligator_mississippiensis_      -----
gi|Ambystoma_mexicanum_gamma        -----
gi|Mus_musculus_gamma               -----
gi|Rattus_norvegicus_gamma          -----
gi|Ovis_aries_gamma                 -----
gi|Bos_taurus_gamma                 -----
gi|Canis_lupus_familiaris_gamm      -----
gi|Macaca_mulatta_gamma             -----
gi|Homo_sapiens_gamma               -----
gi|Felis_catus_gamma                -----
gi|Canis_lupus_familiaris_delt      -----
gi|Macaca_mulatta_delta/gamma       -----
gi|Bos_taurus_delta/gamma           -----
gi|Mus_musculus_delta/gamma         -----
gi|Rattus_norvegicus_delta/gam      -----
gi|Danio_rerio_gamma                -----
gi|Danio_rerio_delta/gamma          -----
gi|Xenopus_laevis_delta/gamma       -----
gi|Xenopus_laevis_gamma             -----
gi|Xenopus_tropicalis_delta/ga      -----
gi|Mus_musculus_delta               -----
gi|Rattus_norvegicus_delta          -----
gi|Felis_catus_delta                -----
gi|Bos_taurus_delta                 -----
gi|Canis_lupus_familiaris_delt      -----
gi|Macaca_mulatta_delta             -----
gi|Homo_sapiens_delta               -----
gi|Ovis_aries_delta                 -----
gi|Falco_peregrinus_delta           -----
gi|Gallus_gallus_delta              -----
gi|Alligator_mississippiensis_      -----
gi|Chrysemys_picta_bellii_delt      -----
gi|Danio_rerio_delta                -----
gi|Ambystoma_mexicanum_delta        -----
gi|Petromyzon_marinus_S4RN43        -----
jgi|Branchiostoma_floridae_237      -----
jgi|Branchiostoma_floridae_252      -----
gi|Strongylocentrotus_purpurat      -----
gi|Caenorhabditis_elegans_PPTR      -----
gi|Hydra_vulgaris_delta             -----
gi|Amphimedon_queenslandica_de      -----
gi|Drosophila_melanogaster_B56      -----
gi|Dictyostelium_discoideum_ps      RYTFT 628
gi|Dictyostelium_purpureum_N/A      ISQ-- 551
gi|Dictyostelium_fasciculatum_      MIF-- 598
```

Figure S1. *Cont.*

```
gi|Polysphondylium_pallidum_N/      SQ--- 530
gi|Arabidopsis_thaliana_theta       -----
gi|Arabidopsis_thaliana_eta         -----
gi|Arabidopsis_thaliana_zeta        -----
gi|Arabidopsis_thaliana_gamma       -----
gi|Oryza                            -----
gi|Oryza_sativa_zeta                -----
gi|Oryza_sativa_theta               -----
gi|Oryza_sativa_eta                 -----
gi|Arabidopsis_thaliana_delta       -----
gi|Arabidopsis_thaliana_N/A         -----
gi|Oryza_sativa_kappa               -----
gi|Oryza_sativa_N/A                 -----
gi|Arabidopsis_thaliana_alpha       -----
gi|Arabidopsis_thaliana_beta        -----
gi|Oryza_sativa_N/A_2               -----
gi|Arabidopsis_thaliana_epsilo      -----
gi|Chlamydomonas_reinhardtii_w      -----
gi|Schizosaccharomyces_pombe_P      -----
gi|Schizosaccharomyces_pombe_P      -----
gi|Ashbya_gossypii_RTS1             -----
gi|Saccharomyces_cerevisiae_RT      -----
```

Figure S1. A CLUSTAL 2.1 multiple sequence alignment of all analyzed B56 sequences.
